# Boragerma[5]pyramidanes via a Germole-to-Borole Rearrangement

**DOI:** 10.1021/acs.inorgchem.6c00260

**Published:** 2026-03-24

**Authors:** Lukas Bührmann, Amrit Chandi, Nadeschda Geibel, Lena Albers, Marc Schmidtmann, Thomas Müller

**Affiliations:** † 11233 Institute of Chemistry, Carl von Ossietzky Universität Oldenburg, Carl von Ossietzky-Strasse 9-11, D-26129 Oldenburg, Federal Republic of Germany, European Union

## Abstract

The scope and limitations of a germole-to-borole rearrangement are reported. Double salt metathesis reactions of dipotassium germolediides with amino-, aryl-, and ferrocenyl-substituted boron dihalides allow for the preparation of borole complexes of Ge­(II) in synthetically useful yields and quantities. The analogous silole-to-borole transformation is viable but is less selective and of limited synthetic use. The analysis of the molecular and electronic structure of the obtained Ge­(II) borole complexes classifies them as boragerma[5]­pyramidanes, molecular *nido*-type clusters. The molecular structure can be fine-tuned as strongly electron-donating substituents at the boron atom induce an opening of the cluster. The boragerma[5]­pyramidanes bind via the germanium atom to transition metal complexes and behave as σ-donors with only insignificant π-acceptor abilities. Their reduction with elemental lithium and their reaction with strong nucleophiles such as N-heterocylic carbenes (NHCs) lead to the elimination of germanium and isolation of borole derivatives.

## Graphical abstract

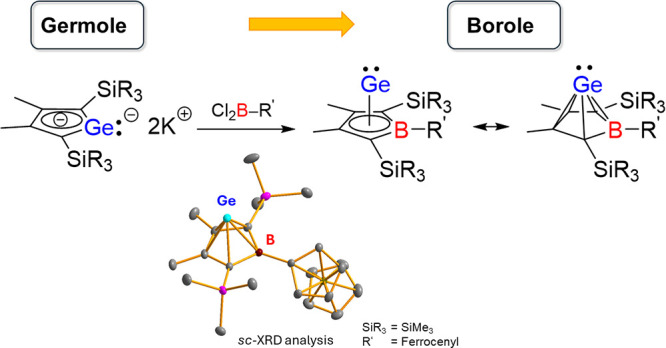

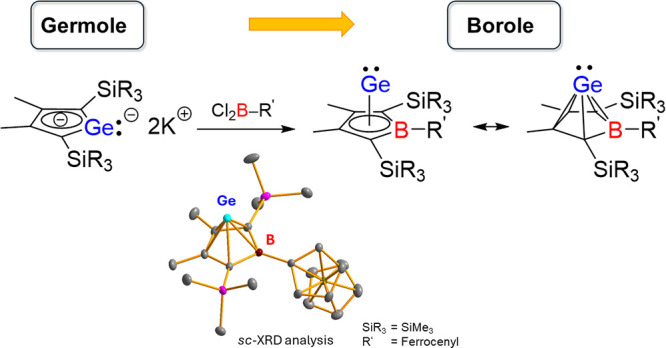

## Introduction

Borolediides, [(R^1^B­(CR)_4_]^2–^ are dianionic heterocyclic analogues of monoanionic cyclopentadienides [(CR)_5_]^−^.^1–3^ Transition metal complexes of borolides are well documented since the pioneering work of Herberich and co-workers starting in the early 1980.^4–7^ Recent investigations by the groups of Braunschweig and Breher indicated that boroles can undergo stepwise reductions to form monoanionic borole radicals and aromatic borole dianions.^1,8^ In principle, this suggests that these five membered bora-heterocycles can stabilize different oxidation states of transition metal ions and can act as redox-active ligands. Reports on complexes of borolides with p-block elements are relatively scarce. Herberich and co-workers reported the formation of a cluster compound **2** of the *nido*-C_4_B_2_H_6_ family by salt metathesis reaction of dilithium borolediide Li_2_[**1**] with an alkyl boron dichloride in 1984 (Scheme 1).^9^ More than 30 years later, the group of Sindlinger used this approach to synthesize borolediide complexes of the group 14 elements E (E = Si, Ge, Sn) **3**-**7** from sterically protected dilithium borolediides and well-selected E­(II) sources.^10,11^ The same group reported also the synthesis of an aluminum borolediide sandwich complex **9** by reaction of the neutral borole **8** with Cp*Al­(I), which is a formal redox complexation.^12^ Interestingly with Cp*Ga­(I) the same borole forms the Lewis-acid/-base complex **10** without a redox event.^12,13^ Parallel to Sindlinger’s work, our group reported a germole-to-borole transformation starting from dipotassium germolediides K_2_[**11**], which provides access to germanium complexes of boroles **12**.^13^ As covalent bonding prevails between the germanium atom and the borole ring, these Ge­(II) complexes **12** can be described as *nido*-clusters or boragerma[5]­pyramidanes.^14,15^ Here, we will discuss the scope and the limitations of the germole-to-borole transformation to give germa[5]­pyramidanes, its extension to silicon complexes and initial reactivity studies.

**Scheme 1:**
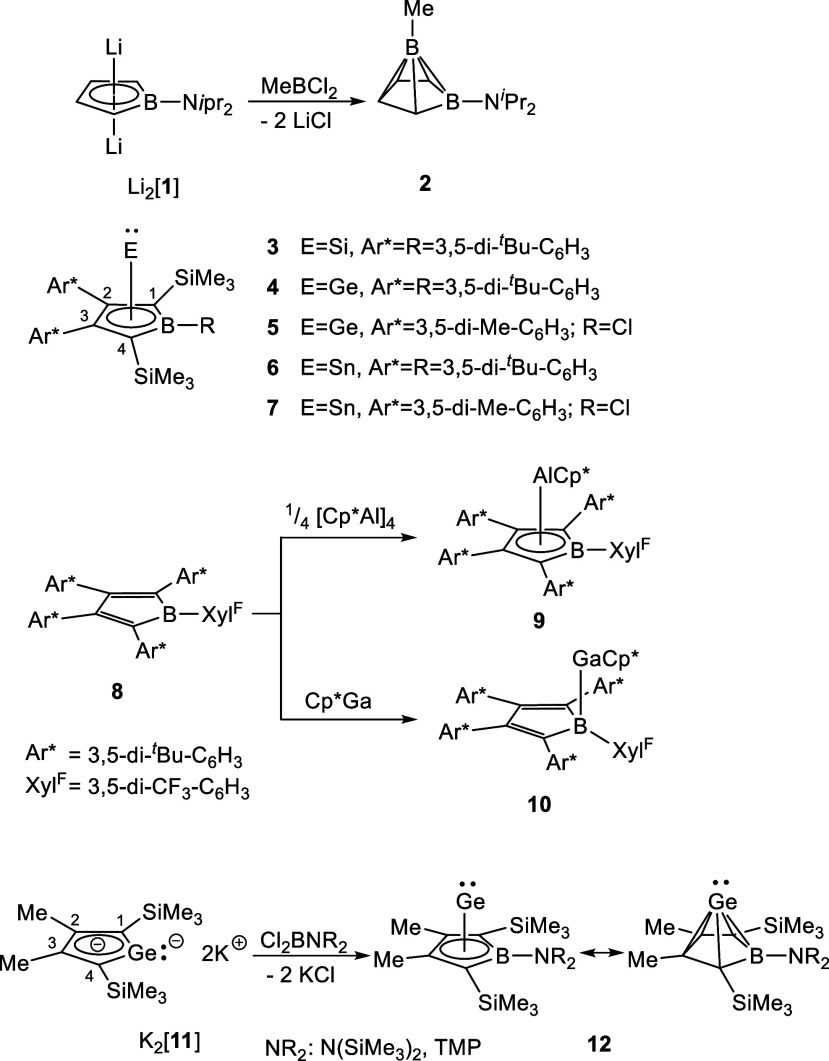
Synthesis of Main Group Element Borole Complexes (TMP = tetramethylpiperidinyl)

## Results and Discussion

The synthesis of boragerma[5]­pyramidanes **12** and **14** was achieved via double salt metathesis reactions, using boron dihalides **15** as reaction partner for dipotassium germolediide salts K_2_[**11**] and K_2_[**13**] as reported previously for **12a**-**c** (Scheme 2).^13,16^ The reaction proceeds via strongly polarized heterofulvenes **16** and bicyclic germylenes **17**. Examples for both types of intermediates, **16** and **17**, are known from reactions of dipotassium germolediides with other element dihalides^17–19^ but neither **16** nor **17** are detected in the reaction with boron dihalides (see ref.^13^ and Supporting Information, Figure S81 for the results of a DFT investigation of the mechanism). Overall, this transformation constitutes a germole-to-borole conversion that proceeds selectively, affording the corresponding boragerma[5]­pyramidanes **12** and **14** in isolated yields ranging from 31% to 75%. Reactions with boron dichlorides with strongly electron-withdrawing or sterically demanding substituents, such as (C_6_F_5_)­BCl_2_ or Mes*BCl_2_ (Mes* = 2,4,6-^*t*^Bu_3_C_6_H_2_), as well as with boron tribromide, were unsuccessful, leading only to complex product mixtures. The new germanium borole complexes **12d**-**f** and **14a**,**d**-**f** were characterized via heteronuclear NMR spectroscopy, high-resolution mass spectrometry (HR-MS) and in the case of **12e**,**g** and **14a**,**e-g** also by single crystal X-ray diffraction (sc-XRD) analysis. The ^11^B NMR resonances of the new borole complexes **12d**-**f** and **14a**,**d**-**f** appear in a characteristic region at δ^11^B = 29.4 (**12d**) - 35.6 (**14a**) (for comparison: **12a**-**c**: δ^11^B = 29.5 – 37.2 and **4**, **5**: δ^11^B = 29.2, 30.2 (Scheme 1))^11,13^ which is almost identical to that of related borole dianions (δ^11^B = 27 – 37 and Li_2_[**18**]: δ^11^B = 37).^6,10,20^ Typical for the germanium borole complexes are also the ^13^C NMR chemical shifts of the sp^2^-hybridized ring carbon atoms. The broad signals (Δω_1/2_ = 27.8 Hz (**12d**) and Δω_1/2_ = 24.3 Hz (**14d**)) of the carbon atoms C^1/4^ appear at low frequencies (δ^13^C^1/4^ = 89.3 (**12e**) – 107.0 (**12d**)) while the carbon atoms C^2/3^ resonate in a region expected for sp^2^-hybridized carbon atoms (δ^13^C^2/3^ = 128.3 (**12e**) – 136.0 (**14a**)). This data is fully consistent with ^13^C NMR chemical shifts obtained for other Ge­(II)­borole complexes such as **12a**-**c**, **4** and **5** ^11,13^ and for Li_2_[**18**] (δ^13^C­(C^1/4^) = 99 – 113, δ^13^C­(C^2/3^) = 128 – 142, see Figure 1 and Table S1).

**Scheme 2:**
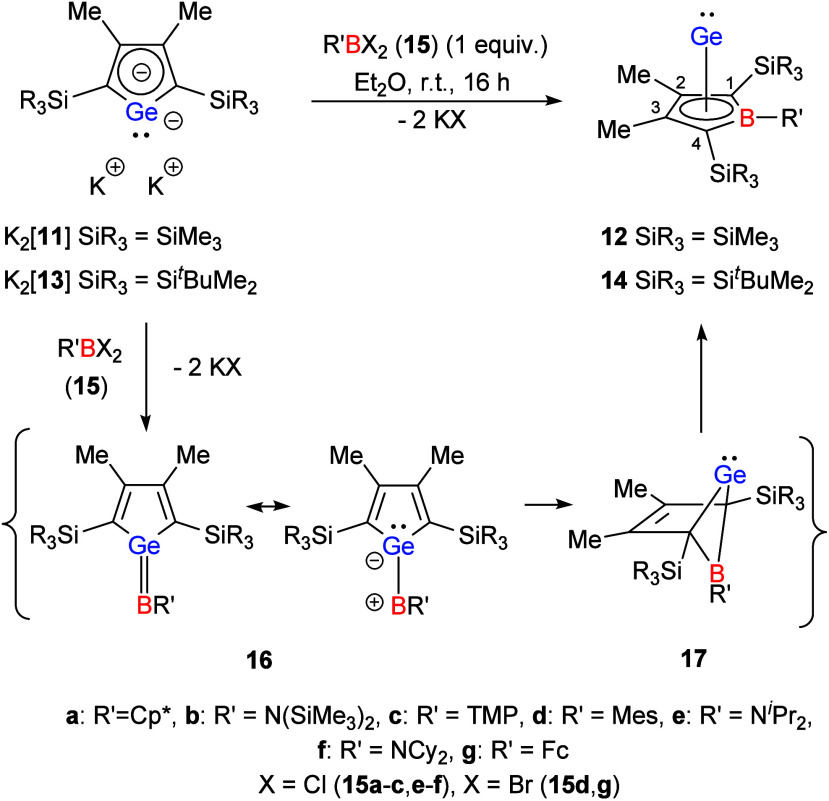
Synthesis of Boragerma[5]­pyramidanes **12** and **14** (TMP = 2,2,4,4-tetramethyl-piperidinyl)

**Figure 1:**
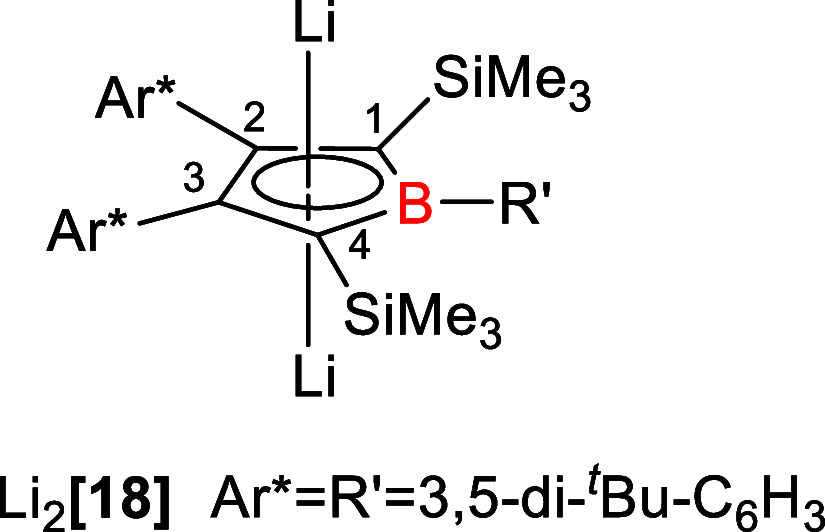
Dilithium borolediide Li_2_[**18**] reported by Sindlinger and co-workers.^10^

It is noteworthy, that our attempts to synthesize related Si­(II) pyramidanes **20** were less successful (Scheme 3). The reaction of dipotassium silolediide K_2_[**19**] with different boron dichlorides **15** resulted in the formation of intractable mixtures. Only in the reactions with the two amino-substituted boron chlorides **15b** and **15f**, we clearly identified as main products the respective Si­(II) complexes **20b** and **20f** by NMR spectroscopy (**20b**: δ^13^C­(C^1/4^) = 100.5, δ^11^B = 33.2, δ^29^Si­(Si­(II)) = −348.3;^21^ **20f**: δ^13^C­(C^2/3^) = 136.8, δ^13^C­(C^1/4^) = 88.7, δ^11^B = 35.0, δ^29^Si­(Si­(II)) = −337.5 (Scheme 2). These NMR data agree with results from the Sindlinger group obtained for the Si­(II)­borole complex **3** (δ^13^C­(C^2/3^) = 139.2, δ^13^C­(C^1/4^) = 110.4, δ^11^B = 31.5, δ^29^Si­(Si­(II)) = −349.4).^10^ Optimization of the reaction conditions for the synthesis of complex **20f** resulted in the isolation of the raw material in 77% yield, which could not further be purified by crystallization, due to its high solubility in all tested solvents.

**Scheme 3:**
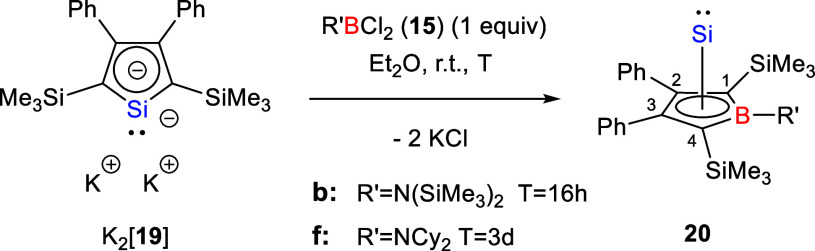
Attempted Synthesis of Borasila[5]­pyramidanes **20**

Crystals suitable for sc-XRD analysis were obtained from germapyramidanes **12e**,**g** and **14a**,**e**-**g**. The obtained molecular structures are very similar (see Table 1) and resemble closely that of the published TMP-substituted complex **12c**.^13^ Figure 2 shows the molecular structure of complexes **12e** and **14e** as examples (see also the Supporting Information for the structures of **12g** and **14a**,**f**,**g**). The borole rings are almost planar as the angles α­(B) between the midpoints of the C2/C3 and C1/C4 atom distances and the boron atom, are almost 180° (α­(B) = 167.2–171.5°, see Figure 2 and Table 1). The inner-cyclic C–C bonds of the borole rings are shorter than regular C–C single bonds (154 pm) and follow a long–short–long pattern (145.9–147.3 pm/142.3–143.2 pm/146.3–147.2 pm). The B–C bonds (156.9–158.5 pm) are short B–C single bonds (standard B–C single bond 160 pm).^22^ The C–Ge distances are all very similar (217.8–221.2 pm) and ca. 10% longer than regular Ge–C single bonds (196 pm)^22,23^ but significantly smaller than the sum of the van der Waals radii of both elements (ΣvdWr­(C/Ge)=381 pm).^24^ Similarly, the B–Ge distance is ca. 10% longer than regular B–Ge single bonds (theoretical value: 206 pm,^22^ mean value from sc-XRD: 214 pm).^23^ These structural features suggest that compounds **12** and **14** can be best regarded as Ge­(II) borole complexes or alternatively as *nido*-type main group cluster ([5]­pyramidanes) (Figure 3). This description is supported by an analysis of the bonding situation of complex **12a**, which indicates multicenter bonding between the germanium atom and the borole ring.^13^

**Figure 2:**
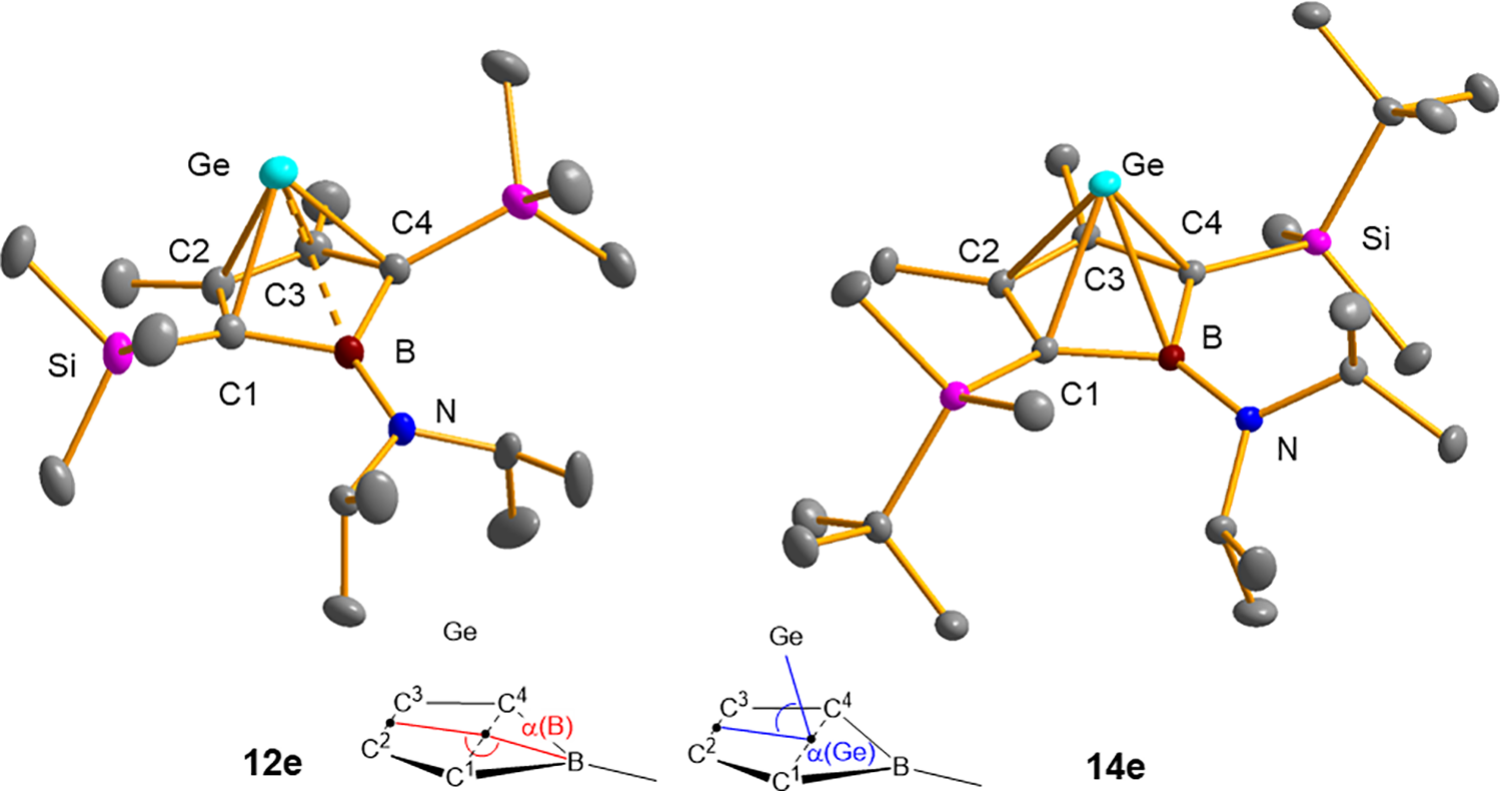
Molecular structures of boragerma[5]­pyramidanes **12e** and **14e** in the crystal. Thermal ellipsoids at 50% probability. Hydrogen atoms have been omitted for the sake of clarity. For selected atomic distances and angles, see Table 1.

**Table 1:** Structural Parameters of Borole Complexes of Germanium­(II) from sc-XRD (bond lengths in picometers and bond and dihedral angles in degrees)

parameter	**14a**	**12e**	**14e** ^a^	**14f**	**12g**	**14g**
Ge–B	230.7	253.4	235.3	233.2	231.4	234.4
Ge–C1/C4^b^	220.5	218.3	221.2	219.2	219.6	219.3
Ge–C2/C3^b^	220.6	217.8	218.2	219.1	221.0	220.3
C1–C2	145.9	147.3	146.4	146.4	146.1	146.8
C2–C3	142.3	142.9	143.0	142.5	142.6	142.8
C3–C4	146.4	147.2	146.3	146.4	146.6	146.8
B–C1/C4^b^	158.1	158.5	158.5	157.7	156.9	157.4
B–N/C	162.3	142.5	145.5	147.0	157.4	157.1
C1–B–C–C	54.9	–	–	–	35.2	6.7
C1–B–N–C		–2.9	43.8	70.5	–	–
α(Ge)^c^	80.7	79.5	79.0	80.2	81.0	80.8
α(B)^d^	171.5	152.6	171.1	169.6	169.9	167.2

a Crystal structures shows disorder (3%). Data given for the main component.

b Average of two equivalent values.

c Angle among the midpoints of C1/C4, C2/C3, and Ge (see Figure 2).

d Angle among the midpoints of C1/C4, C2/C3, and B (see Figure 2).

**Figure 3:**
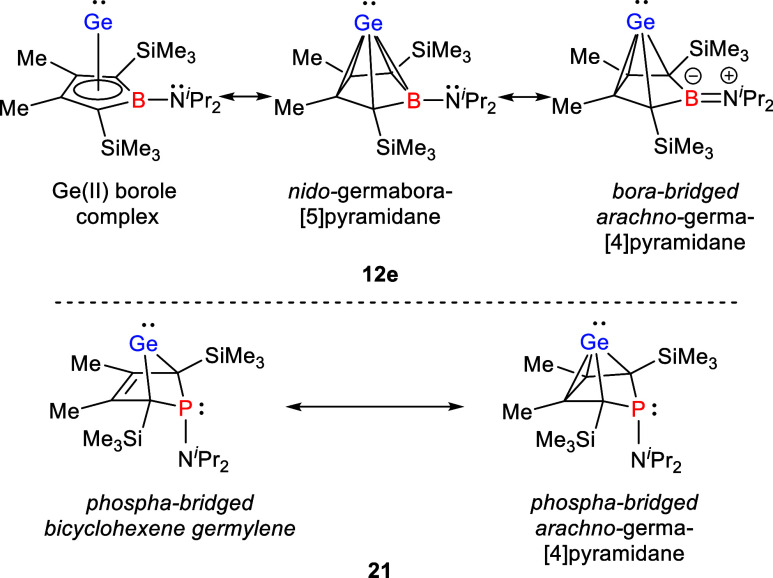
Different representations of compound **12e** as an example of germanium complexes of boroles and comparison with bicyclohexene germylene **21**.^25^

There is one structural feature in the structures of germanium aminoborole complexes **12c,e** and **14e**,**f** which deserves attention. When the boron atom is involved in the multicenter cluster bonding, the π-interaction between the nitrogen substituent and the boron center is canceled and the conformation around the exocyclic B–N bond is determined by steric effects. In the case of **12c**, **14e,f**, this leads to large dihedral angles between the borole ring and the plane of the amino-substituent (C1–B–N–C: 101.1° (**12c**), 43.8° (**14e**) 76.5° (**14f**)). In the case the germanium borole complex **12e** the steric interaction between the *iso*-propyl groups at the nitrogen atom and the trimethylsilyl groups at the borole ring is small enough to allow for B–N-π-interaction (C1–B–N–C: −2.9° (**12e**), see Figure 2 and Supporting Information for details). Consequently, the Ge/B distance widens by additional 8% (18 pm) and the bending of the borole ring increases as measured by the angle α­(B) (Table 1) compared to the ^*t*^BuMe_2_Si-substituted borole complex **14e**. These changes can be interpreted as the beginning exclusion of the boron atom from the cluster bonding and the inceptive transformation of the *nido*- to an *arachno*-type structure like already found for the phospholes derivate **21** (Figure 3).^25^ Related is also the case of C_4_B_2_H_6_ isomers. Substitution of the boron atoms with amino functionalities results in a destabilization of the *nido*-cluster compared to the classical structure.^9^

These findings are supported by the results of density functional calculations at the M06-2X/Def2-TZVP level^26^ for the model Ge­(II)­aminoborole complex **12M** in its two extreme conformations around the B–N bond, *perpendicular per*-**12M** and *planar pl*-**12M** (Figure 4). In agreement with the results obtained previously for borole complex **12c**,^13^ the HOMO of *per*-**12M** is dominated by contributions of the nitrogen lone pair and the empty 2p-orbital at the boron center is integrated in the multicenter cluster bonding (see Figures S72 and S73 for surface diagrams of the frontier orbitals of **12M**). In the absence of steric congestion, this conformation is the transition state of the rotation around the B–N bond. The minimum conformer is *pl*-**12M**, by 41 kJ mol^–1^ lower in energy. In this case, the nitrogen lone pair and the 2p-orbital at the boron atom are involved in B–N-π-bonding and the energy of the resulting MO is significantly lowered (HOMO–3, Figure 4). The most pronounced structural consequences of this electronic reorganization are a shortening of the B–N bond by 4 pm and a significant increase in the B/Ge distance by 10 pm (see Figure S75 for further details). This effect is demonstrated experimentally by the large B/Ge distance of compound **12e** compared to **14e** (see discussion above, Figure 2 and Table 1).

**Figure 4:**
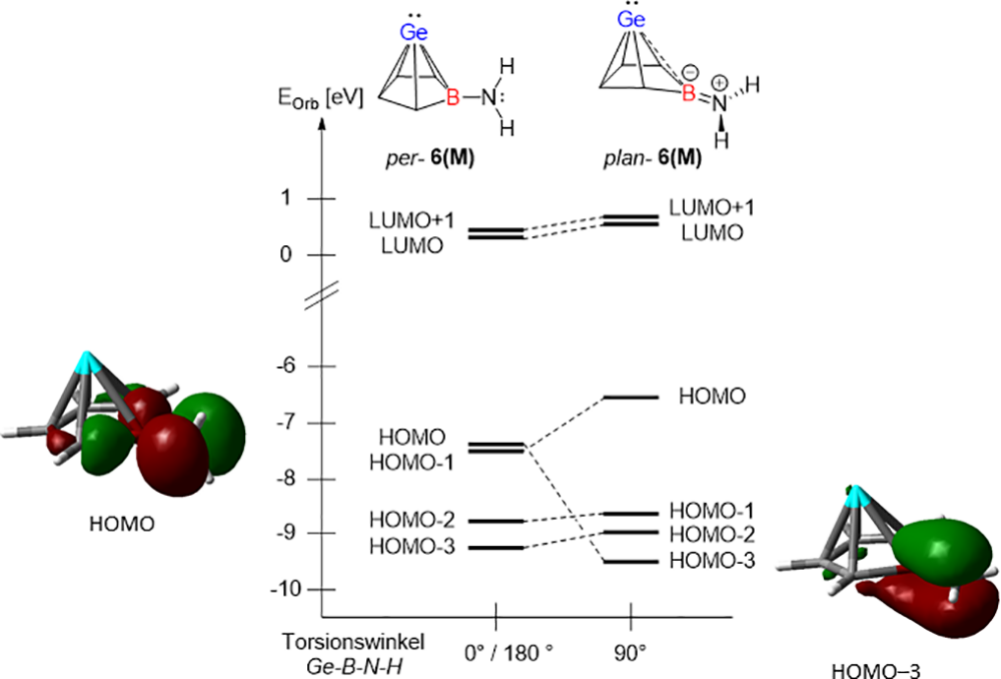
Walsh diagram for the rotation around the B–N bond in germanium borole complex **12M**. Surface diagrams of selected orbitals in both extreme conformations (calculated at the M06-2X/Def2-TZVP level, surface isodensity value of 0.05).

The molecular structure of the ferrocenyl-substituted germanium borole complexes **12g** and **14g** are very similar to their amino-substituted counterparts (Table 1). A structural characteristic of boryl- and borolyl-substituted ferrocenes is the bending of the substituents toward the iron atom due to multicenter bonding between the Lewis-acidic substituents and the ferrocene group (for detailed NBO analysis of these two compounds, which compares the B/Fe interaction, see Supporting Information, Figure S79).^30^ This leads to a significant shortening of the B/Fe separation which becomes smaller with increasing Lewis-acidity of the boron-substituent (see Figure 5). For the here investigated compounds **12g** and **14g**, the tilt angle α is slightly larger than for the ionic magnesium borole complex **24** but significantly smaller than for the ferrocenyl-substituted boroles **22** and **23**.^27–29^ In particular, the direct comparison between the molecular structures of the borole **25** (synthesized independently) and the germanium borole complex **14g** (Figure 5) indicates the strongly reduced Lewis-acidity of the boron center in germanium borole complexes **12g** and **14g**.^31^

**Figure 5:**
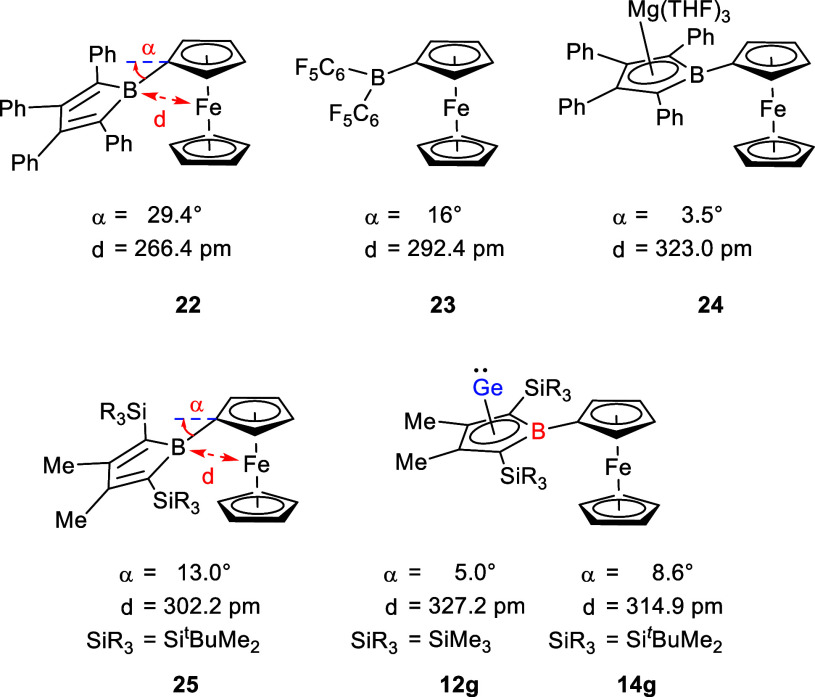
Selected structural parameters of boryl- and borolyl-substituted ferrocenes.^27–29^

Germanium­(II) borole complexes **12** and **14** are quite inert, they react only with strong Lewis-acids, strong donors and strong reductants. At first glance, this is surprising, since derivatives of the isolobal Cp half-sandwich complexes of Al­(I) **26**,^32–35^ Ge­(II) [**27**]^+^ ^36^ and P­(III) [**28**]^2+^ ^37^ exhibit diverse chemistry, albeit with very different characteristics. The reactivity of the respective Cp* complexes shifts from a predominantly nucleophilic behavior in the neutral aluminum­(I) complex to strongly electrophilic character in the dicationic phosphorus­(III) half-sandwich species. This behavior is mirrored by the computed energies of the frontier orbitals of the Cp half-sandwich complexes **26**, [**27**]^+^ and [**28**]^2+^ (see Figure 6). Based on this comparison the model Ge­(II)­borole complex *per*-**12M** (and likewise the investigated substituted congeners) should be less nucleophilic and similar weakly electrophilic as the aluminum­(I) complex **26**. Based on our frontier orbital analysis shown in Figure 4, we reasoned that the most promising compound for a reactivity study is the amino-substituted borole complex **12e**.

**Figure 6:**
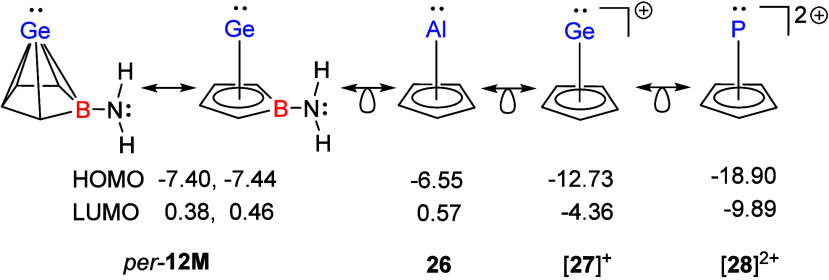
Calculated energies of frontier orbitals in *per*-**12M** and isolobal cyclopentadienyl half-sandwich complexes of aluminum **26**, germanium [**27**]^+^, and phosphorus [**28**]^2+^ (in electronvolts, calculated at the M06-2X/Def2-TZVP level).

Inspired by the results of the groups of Coburger,^38^ Berndt^39^ and Lee^20^ that demonstrated the clean conversion of *nido*-clusters into two-dimensional aromatic compounds upon two electron reduction,^40^ we tested the reactivity of the germanium borole complex **12e** versus strongly reducing reagents. Its reduction with KC_8_ and elemental potassium at different temperatures in different stoichiometric relations lead to nonseparable mixtures of at least three boron-containing compounds. In contrast, the reaction with excess lithium results in the formal elimination of germanium from the molecule and selective formation of the dilithium borolediides [Li­(solv)]_2_[**29**] in yields of 79% (solv = Et_2_O) and 74% (solv = THF) Scheme 4). The ion pairs [Li­(solv)_2_]­[**29**] were identified in solution by NMR spectroscopy. In addition, the lithium/THF solvate [Li­(THF)]_2_[**29**] was identified by high resolution mass spectrometry (HR-MS), which gave the correct mass for the solvated triple ion (*m*/*z* = 493.4104 Da for C_26_H_54_BLi_2_NO_2_Si_2_ (theor. 493.4106 Da)). ^7^Li NMR spectra of both solvates show only one resonance at very low frequence ([Li­(THF)]_2_[**29**] δ ^7^Li = −6.1 and [Li­(OEt_2_)]_2_[**29**] δ ^7^Li = −6.3) which indicates the formation of triple ion pairs with a η^5^-coordination of both lithium ions to the borole ring. The characteristic ^13^C and ^11^B NMR data of both solvates are very similar, therefore we refer here only to the THF solvated species. The ^11^B NMR chemical shift (δ ^11^B = 31.6) and the ^13^C NMR chemical shifts of the ring carbon atoms (δ ^13^C­(C^1/4^) = 98.9, δ ^13^C­(C^2/3^) = 117.8) are in the typical region for related borole dianions reported previously by Herberich, Lee and Sindlinger (see Figure 7).^6,10,20^

**Scheme 4:**
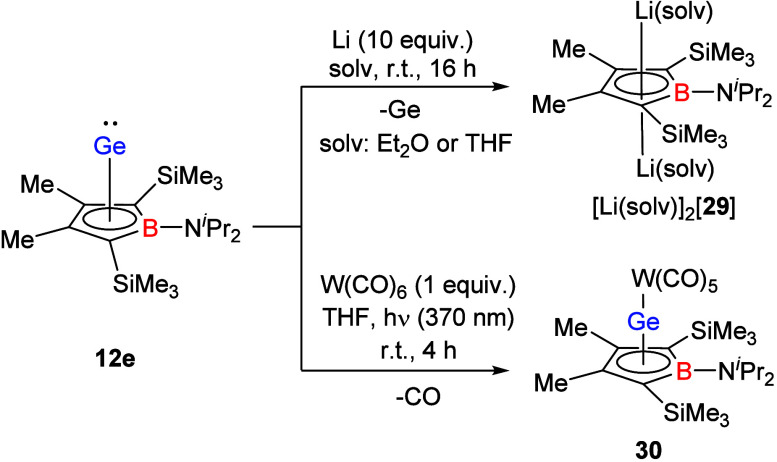
Selected Reactions of Ge­(II)­borole Complex **12e**

**Figure 7:**
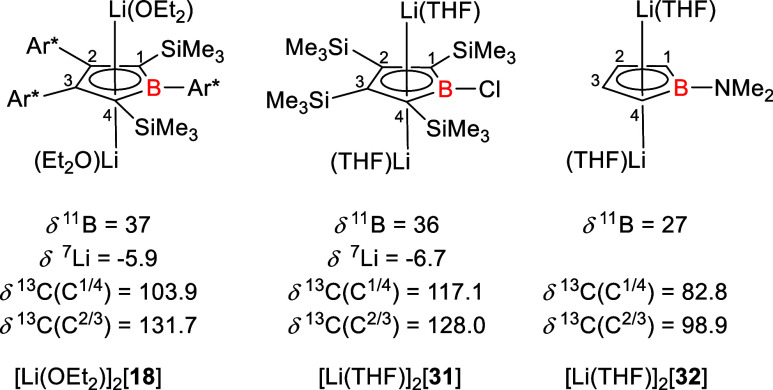
Relevant NMR data of different dilithium borolediides (Ar* = 3,5-di-^*t*^BuC_6_H_3_).

The nucleophilic properties of the borole complex **12e** were tested by reactions with several metal complexes. While we noticed no reactivity toward late transition metal complexes such as CpCo­(CO)_2_ and Ni­(COD)_2_, (COD: cyclooctadiene), we observed only incomplete reaction with Fe_2_(CO)_9_. In contrast, with the tungsten carbonyl complex W­(CO)_5_(THF), which was generated in situ by UV-irradiation of the corresponding hexacarbonyl complex, a smooth reaction occurred, providing the tungsten complex **30** in 80% yield (Scheme 4). This result is noteworthy as the related germapyramidanes **4** and **5** do not form stable tungsten carbonyl complexes when similar reaction conditions were applied.^11^ Complex **30** was characterized by NMR and IR spectroscopy. Orange crystals suitable for sc-XRD analysis were obtained by recrystallization from a *n*-pentane solution at −25 °C (Figure 8).

**Figure 8:**
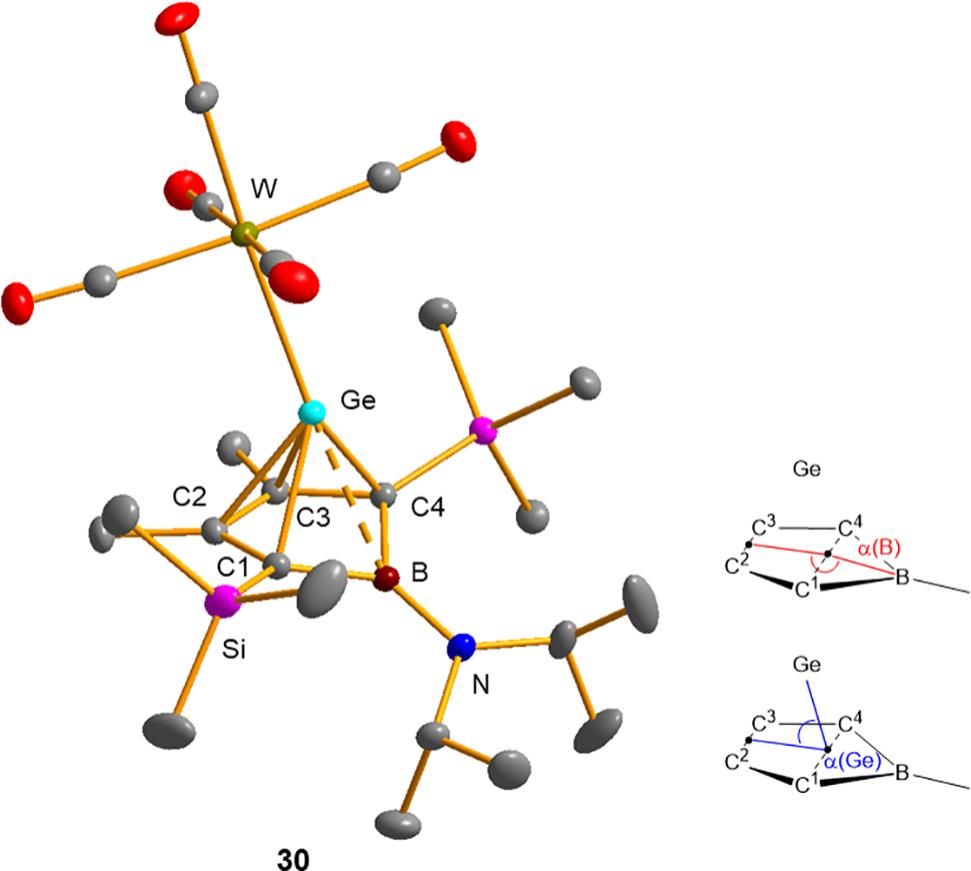
Molecular structure of tungsten carbonyl complex **30** in the crystal. Thermal ellipsoids at 50% probability. Hydrogen atoms have been omitted for the sake of clarity. Selected atomic distances (picometers) and angles (degrees): Ge–B, 244.8(1); Ge–W, 260.1(3); Ge–C1, 210.3(2); Ge–C2, 220.9(1); Ge–C3, 221.2(1); Ge–C4, 211.0(2); C1–C2, 148.6(2); C2–C3, 141.8(3); C3–C4, 147.7(2); B–C1, 160.3(2); B–C4, 160.0(2); B–N, 141.2(2); Ge–B–N, 146.6(1); W–Ge–B, 175.6(4); B–N–C16, 120.4(1); Ge–B–N–C16, 105.9; α­(B), 149.0.

The ^13^C NMR chemical shifts of the C^1^–C^4^ carbon atoms undergo only small changes upon complexation. There is a shift to lower frequencies for the resonances of the bridgehead carbon atoms C^1/4^ of Δ­(δ^13^C) = −14.3 (δ^13^C (C^1/4^) = 89.5 (**12e**) vs 75.2 (**30**)) and a concomitant shift to higher frequencies of the resonances corresponding to the carbon atoms C^2/3^ by Δ­(δ^13^C) = +4.7 (δ^13^C­(C^2/3^) = 128.0 (**12e**), 132.7 (**30**)). Finally, the ^11^B signal is slightly shifted to higher frequencies by Δ­(δ^11^B) = +3.6 (δ^11^B­(C^1/4^) = 33.3 (**12e**) vs 36.9 (**30**)). These small changes suggest an almost complete conservation of the electronic situation of the germapyramidane upon complexation. This assumption was further supported by the molecular structure obtained from sc-XRD analysis (Figure 8). All pertinent structural parameters of the not coordinated germapyramidane ligand **12e** are almost unchanged. The most significant differences are related to the Ge–C1/C4 bonds, which are shortened by approximately 8 pm (Ge–C1: 218.3 pm (**12e**), 210.3 pm (**30**), Ge–C4: 218.2 pm (**12e**), 211.0 pm (**30**)). The Ge–B separation in the tungsten complex **30** is shorter than in the starting germapyramidane **12e** (by 8.4 pm) but longer than in all other structurally verified Ge­(II)­borole complexes (by app. 10–14 pm, see Table 1). In view of the unusually large Ge/B separation in germapyramidane **12e** (see above), the latter comparison is more appropriate. The Ge–W bond length (260.1 pm) is in the normal range for Ge–W single bonds (exp. mean value 260 pm,^23^ theor. value: 258 pm),^22^ which suggests that there is no π-back-bonding from the transition metal to the germapyramidane ligand. This agrees with results from IR spectroscopy. The band of the total symmetric (^1^A) CO stretch vibrations appears at *ṽ* = 2072 cm^–1^ which translates into a Tolman electronic parameter (TEP) of 2068 cm^–1^.^41,42^ This value is typical for N-heterocyclic germylenes (TEP = 2051 – 2080 cm^–1^)^42^ and comparable to that of trialkylphosphanes (i.e., TEP­(^*t*^Bu_3_P) = 2056 cm^–1^).^41^ It suggests that the germapyramidanes **12**, **14** are almost pure σ-donors with only insignificant π-acceptor abilities.

Germapyramidane **12e** reacts with tetramethylimdazolylidene ^Me4^Im at room temperature to give one main product accompanied by several side products. Due to its high solubility in all tested organic solvents, the product could not be isolated as pure substance. By comparison with the data reported by the Sindlinger group for borole-NHC complex **34**,^43^ the acquired NMR spectroscopic data suggest formation of an NHC-coordinated borole **33** (see Scheme 5). This was supported further by an exact HR-MS of *m*/*z* = 459.3628 Da (theor. 459.3636 Da).

**Scheme 5:**
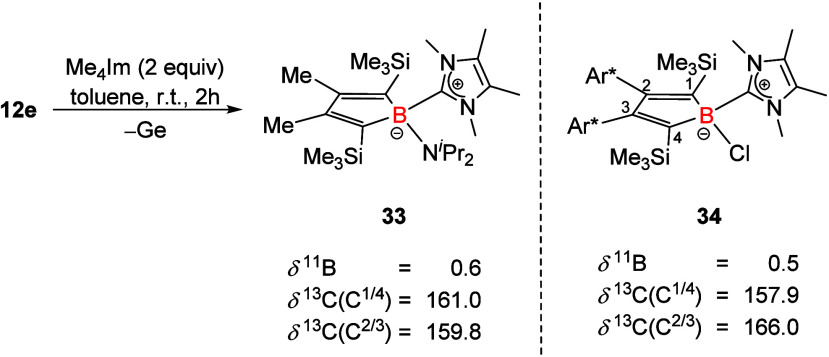
Reaction of Ge­(II) Borole Complex **12e** with Me_4_Im and NMR Data of NHC-borole Complex **34** (Ar* = 3,5-^*t*^Bu_2_(C_6_H_3_)^43^

## Conclusions

We report a one-step germole-to-borole transformation which allows the synthesis of a family of Ge­(II)­borole complexes **12** and **14**. The double salt metathesis reaction of dipotassium germolediides K_2_[**11**] and K_2_[**13**] with boron dihalides provides preparative access to cycloalkyl-, aryl- and aminosubstituted borole complexes in significant isolated yields and is a viable alternative to the salt metathesis reaction between borolediides and Ge­(II) sources. We demonstrated also the analogous silole-to-borole transformation for two selected aminosubstituted boron dihalides, although in this case the reaction is significantly less selective. The structural and electronic properties of the Ge­(II) borole complexes **12** and **14** allow their classification as *nido*-type cluster or [5]­pyramidanes although π-electron donating substituents at boron enforce more open structures in agreement with an incipient *nido* → *arachno* transformation. Our initial reactivity studies indicate that the germa[5]­pyramidanes are weak σ-donors forming metal complexes only with very strong Lewis acids. Reduction with alkali metals yields mixtures of several species or result in elimination of the germanium. Similarly, the reaction with strong σ-donors such as N-heterocyclic carbenes leads to decomposition presumably via germanium elimination.

## Experimental Section

### General

All manipulations of air- and moisture-sensitive compounds were carried out under an argon 5.0 or nitrogen 5.0 atmosphere using Schlenk techniques or a standard glovebox (Braun Unilab). Glassware was dried in an oven at T = 140 °C and evacuated three times prior to use. The solvents THF, diethyl ether, toluene, *n*-pentane and *n*-hexane were dried over Na/K alloy and distilled under an inert gas atmosphere. Benzene-d_6_ was dried over potassium and stored over molecular sieves (4 Å). HNCy_2_ and HN^*i*^Pr_2_ were dried over CaH_2_ and distilled under an inert gas atmosphere prior to use. Dichlorogermoles, dichlorosilole and the mesityl copper complex were prepared according to literature procedures.^16,44^ Ferrocene, boron trichloride, boron tribromide, elemental lithium and tungsten hexacarbonyl complex were purchased commercially and used without any further purification. NMR spectra were recorded on Bruker Avance DRX 500, Bruker Avance III 500 and JEOL JNM-ECZL 500 spectrometers. ^1^H NMR spectra were calibrated against the residual proton signal of the solvent as internal reference (benzene-d_6_: δ^1^H­(C_6_D_5_H) = 7.16; THF/D_2_O-capillary: δ^1^H­(C_4_H_8_O) = 3.58). ^13^C­{^1^H} NMR spectra were calibrated by using the central line of the solvent signal (benzene-d_6_: δ^13^C­(C_6_D_6_) = 128.0; THF/D_2_O-capillary: δ^13^C­(C_4_H_8_O) = 67.2)). The ^29^Si­{^1^H} NMR, ^11^B­{^1^H} NMR, and ^7^Li­{^1^H} NMR spectra were calibrated against an external standard (δ^29^Si­(Me_2_SiHCl) = 11.1 versus tetramethylsilane (TMS); δ^11^B­(BF_3_•Et_2_O) = 0.0; δ^7^Li­(LiCl) = 0.0). The ^29^Si­{^1^H} INEPT (*Insensitive Nuclei Enhancement by Polarization Transfer*) NMR spectra were recorded with a delay optimized for SiMe_3_-groups (*n* = 9 and ^2^ *J*_Si,H_ = 8 Hz). The ^29^Si­{^1^H} NMR *invers gated* spectra were recorded with a relaxation time of d = 10.0 s. For a clear assignment of the signals two-dimensional experiments, such as ^1^H^13^C heteronuclear multiple quantum coherence (HMQC) and ^1^H^13^C heteronuclear multiple bond coherence (HMBC) spectra were recorded. Mass Spectra were recorded with a ThermoScientific DFS – High Resolution Magnetic Sector MS (HV-Emitter: 8 kV, emitter heating current (EHC) Ramp: 21 mA/min, emitter heating current (EHC) max Ramp: 95 mA). Infrared spectra were measured and recorded on a Bruker Tensor 27 spectrometer. As the combustion analysis of these compounds is generally not satisfying due to carbide formation, we gave the complete set of NMR spectra and HR-MS data instead.

#### Boragerma­[5]­pyramidane **12d**

Dipotassium 2,5-bis­(trimethylsilyl)-3,4-dimethylgermolediide K_2_[**11**] (0.50 mmol, 1.00 equiv) was prepared as described before.^16^ The resulting suspension was transferred to a Schlenk flask. A solution of MesBBr_2_ **15d** (145 mg, 0.50 mmol, 1.00 equiv) in Et_2_O (30 mL) was added dropwise to the suspension at room temperature. The reaction mixture was stirred for 16 h. The solvent was removed under reduced pressure and the residue was dissolved in *n*-hexane. After filtration, the solvent was removed under reduced pressure. The residue was dissolved in benzene-d_6_ and analyzed by NMR spectroscopy. Complex **12d** could be isolated as a reddish oil (raw yield: 165 mg, 0.39 mmol, 77%). Several attempts to purify compound **12d** by recrystallization did not succeed.

^1^H NMR (499.9 MHz, 305.0 K, C_6_D_6_): δ = 6.88 (s, 2H, C_6_ H_2_(CH_3_)_3_), 2.50 (s, 3H, C_6_H_2_(CH_3_)_3_), 2.28 (s, 3H, C_6_H_2_(CH_3_)_3_), 2.24 (s, 3H, C_6_H_2_(CH_3_)_3_), 2.09 (s, 6H, C^2/3^-CH_3_), 0.00 (s, 18H, C^1/4^-Si­(CH_3_)_3_). ^13^C­{^1^H} NMR (125.7 MHz, 305.0 K, C_6_D_6_): δ = 140.9 (C_6_H_2_(CH_3_)_3_), 137.4 (C_6_H_2_(CH_3_)_3_), 135.4 (C_6_H_2_(CH_3_)_3_), 132.2 (C^2/3^), 127.7 (C_6_H_2_(CH_3_)_3_), 107.0 (C^1/4^), 28.2 (C_6_H_2_(CH_3_)_3_), 24.8 (C_6_H_2_(CH_3_)_3_), 21.4 (C_6_H_2_(CH_3_)_3_), 14.4 (C^2/3^-CH_3_), 1.3 (C^1/4^-Si­(CH_3_)_3_). ^11^B­{^1^H} NMR (160.4 MHz, 305.0 K, C_6_D_6_): δ = 29.4 (C_4_ B-C_6_H_2_(CH_3_)_3_). ^29^Si­{^1^H} INEPT NMR (99.3 MHz, 305.0 K, C_6_D_6_): δ = −8.2 (C^1/4^-Si­(CH_3_)_3_). HR-MS (30 eV, EI): *m*/*z* (C_21_H_35_^11^B^74^GeSi_2_): calc.:428.1582, exp.428.1577.

#### Boragerma­[5]­pyramidane **12e**

Dipotassium 2,5-bis­(trimethylsilyl)-3,4-dimethylgermolediide K_2_[**11**] (0.50 mmol, 1.00 equiv) was prepared as described before.^16^ The resulting suspension was transferred to a Schlenk flask. A solution of ^*i*^Pr_2_NBCl_2_ **15e** (91 mg, 0.50 mmol, 1.00 equiv) in Et_2_O (30 mL) was added dropwise to the suspension at room temperature. The reaction mixture was stirred for 16 h. The solvent was removed under reduced pressure and the residue was dissolved in *n*-hexane. After filtration, the solvent was removed under reduced pressure. The residue was dissolved in benzene-d_6_ and analyzed by NMR spectroscopy. Complex **12e** could be isolated as yellow crystals from a saturated *n*-pentane solution (yield: 63 mg, 0.15 mmol, 31%).

^1^H NMR (499.9 MHz, 305.0 K, C_6_D_6_): δ = 3.76 (sept, ^3^ *J*_H,H_ = 6.7 Hz, 2H, N-(CH­(CH_3_)_2_)_2_), 2.07 (s, 6H, C^2/3^-CH_3_), 1.25 (d, ^3^ *J*_H,H_ = 6.7 Hz, 2H, N-(CH­(CH_3_)_2_)_2_), 0.28 (s, 18H, C^1/4^-Si­(CH_3_)_3_). ^13^C­{^1^H} NMR (125.7 MHz, 305.0 K, C_6_D_6_): δ = 128.3 (C^2/3^), 89.3 (C^1/4^), 49.1 (N-(CH­(CH_3_)_2_)_2_), 25.5 (N-(CH­(CH_3_)_2_)_2_), 15.0 (C^2/3^-CH_3_), 1.3 (C^1/4^-Si­(CH_3_)_3_). ^11^B­{^1^H} NMR (160.4 MHz, 305.0 K, C_6_D_6_): δ = 34.3 (C_4_ B-N^*i*^Pr_2_). ^29^Si­{^1^H} INEPT NMR (99.3 MHz, 305.0 K, C_6_D_6_): δ = −10.1 (C^1/4^-Si­(CH_3_)_3_). HR-MS (30 eV, EI): *m*/*z* (C_18_H_38_^11^B^74^GeNSi_2_): calc.:409.1848, exp.: 409.1841

#### Boragerma­[5]­pyramidane **12f**

Dipotassium 2,5-bis­(trimethylsilyl)-3,4-dimethylgermolediide K_2_[**11**] (0.50 mmol, 1.00 equiv) was prepared as described before.^16^ The resulting suspension was transferred to a Schlenk flask. A solution of Cy_2_NBCl_2_ **15f** (131 mg, 0.50 mmol, 1.00 equiv) in Et_2_O (30 mL) was added dropwise to the suspension at room temperature. The reaction mixture was stirred for 16 h. The solvent was removed under reduced pressure and the residue was dissolved in *n*-hexane. After filtration, the solvent was removed under reduced pressure. The residue was dissolved in benzene-d_6_ and analyzed by NMR spectroscopy. Complex **12f** could be isolated as a dark-yellow oil (raw yield: 184 mg, 0.38 mmol, 75%). Several attempts to purify compound **12f** by recrystallization did not succeed.

^1^H NMR (499.9 MHz, 305.0 K, C_6_D_6_): δ = 3.12–3.07 (m, 2H, N­(CH-C_5_H_10_)_2_), 2.20–2.18 (m, 4H, N­(CH-C_5_ H_10_)_2_), 2.07 (s, 6H, C^2/3^-CH_3_), 1.85–0.98 (m, 16H, N­(CH-C_5_ H_10_)_2_), 0.31 (s, 18H, C^1/4^-Si­(CH_3_)_3_). ^13^C­{^1^H} NMR (125.7 MHz, 305.0 K, C_6_D_6_): δ = 128.6 (C^2/3^), 100.4 (C^1/4^), 57.3 (N­(CH–C_5_H_10_)_2_), 36.3 (N­(CH-C_5_H_10_)_2_), 27.1 (N­(CH-C_5_H_10_)_2_), 26.9 (N­(CH-C_5_H_10_)_2_), 14.8 (C^2/3^-CH_3_), 2.8 (C^1/4^-Si­(CH_3_)_3_). ^11^B­{^1^H} NMR (160.4 MHz, 305.0 K, C_6_D_6_): δ = 32.1 (C_4_ B-NCy_2_). ^29^Si­{^1^H} INEPT NMR (99.3 MHz, 305.0 K, C_6_D_6_): δ = −10.3 (C^1/4^-Si­(CH_3_)_3_). HR-MS (30 eV, EI): *m*/*z* (C_24_H_46_^11^B^74^GeNSi_2_): calc.: 489.2474, exp.: 489.2464.

#### Boragerma­[5]­pyramidane **12g**

Dipotassium 2,5-bis­(trimethylsilyl)-3,4-dimethylgermolediide K_2_[**11**] (0.50 mmol, 1.00 equiv) was prepared as described before.^16^ The resulting suspension was transferred to a Schlenk flask. A solution of FcBBr_2_ **15g** (178 mg, 0.50 mmol, 1.00 equiv) in Et_2_O (30 mL) was added dropwise to the suspension at room temperature. The reaction mixture was stirred for 16 h. The solvent was removed under reduced pressure and the residue was dissolved in *n*-hexane. After filtration, the solvent was removed under reduced pressure. Compound **12g** could be isolated as red crystals from a saturated *n*-pentane solution (yield: 87 mg, 0.18 mmol, 35%).

^1^H NMR (499.9 MHz, 305.0 K, C_6_D_6_): δ = 4.44–4.43 (m, 2H, C_4_B–C_5_ H_4_), 4.27–4.26 (m, 2H, C_4_B–C_5_ H_4_), 4.09 (s, 5H, Fe–C_5_ H_5_), 2.03 (s, 6H, C^2/3^-CH_3_), 0.34 (s, 18H, C^1/4^-Si­(CH_3_)_3_). ^13^C­{^1^H} NMR (125.7 MHz, 305.0 K, C_6_D_6_): δ = 133.5 (C^1/4^), 101.7 (C^1/4^), 76.8 (C_4_B-C_5_H_4_), 69.4 (C_4_B-C_5_H_4_), 69.2 (Fe-C_5_H_5_), 14.8 (C^2/3^-CH_3_), 3.4 (C^1/4^-Si­(CH_3_)_3_). The ^13^C NMR signal of the α-carbon atom bonded to the boron atom could not be determined. ^11^B­{^1^H} NMR (160.4 MHz, 305.0 K, C_6_D_6_): δ = 32.5 (C_4_ B-Fc). ^29^Si­{^1^H} INEPT NMR (99.3 MHz, 305.0 K, C_6_D_6_): δ = −9.1 (C^1/4^-Si­(CH_3_)_3_). HR-MS (30 eV, EI): *m*/*z* (C_22_H_33_^11^B^56^Fe^74^GeSi_2_): calc.:494.0775, exp.: 494.0765.

#### Boragerma­[5]­pyramidane **14a**

Dipotassium 2,5-bis­(*tert*-butyldimethylsilyl)-3,4-dimethylgermolediide K_2_[**13**] (0.50 mmol, 1.00 equiv) was prepared as described before.^16^ The resulting suspension was transferred to a Schlenk flask. A solution of Cp*BCl_2_ **15a** (108 mg, 0.50 mmol, 1.00 equiv) in Et_2_O (5 mL) was added to the suspension at – 80 °C. The reaction mixture was stirred at this temperature for 1.5 h. The cooling bath was removed and the mixture was stirred for additional 21 h. The solvent was removed under reduced pressure and the residue was dissolved in *n*-hexane and filtered over Celite. Then, the solvent was removed under reduced pressure. The residue was dissolved in benzene-d_6_ and analyzed by NMR spectroscopy. Complex **14a** was isolated as a brown oil (raw yield: 143 mg, 0.37 mmol, 73%). A small batch of crystals of **14a** which were suitable for sc-XRD was obtained from benzene at r.t..

^1^H NMR (499.9 MHz, 305.0 K, C_6_D_6_): δ = 2.14 (s, 3H, C^2/3^-CH_3_), 2.09 (s, 3H, C^2/3^-CH_3_), 2.02 1.89, 1.84, 1.82, 1.49 (5x s, 5x 3H, Cp*Me),f 1.14 (s, 9H, Si­(C­(CH_3_)_3_)­(CH_3_)_2_), 0.98 (s, 9H, Si­(C­(CH_3_)_3_)­(CH_3_)_2_), 0.51, 0.16, 0.03, 0.00 (4x s, 4x 3H, C^1/4^-Si­(C­(CH_3_)_3_)­(CH_3_)_2_). ^13^C­{^1^H} NMR (125.7 MHz, 305.0 K, C_6_D_6_): δ = 149.0 (Cp*), 145.4 (Cp*), 136.0 (C^2/3^), 135.4 (C^2/3^), 133.7 (Cp*), 131.5 (Cp*), 101.4 (C^1/4^), 100.7 (C^1/4^), 53.5 (Cp*), 30.2 (^t^BuMe), 29.4 (^t^BuMe), 28.5 (Cp*-Me), 18.9, 18.9 (2x ^t^BuC ^q^), 17.8 (C^2/3^-CH_3_), 17.5 (C^2/3^-CH_3_), 14.0 (Cp*-Me), 13.5 (Cp*-Me), 12.1 (Cp*-Me), 11.9 (Cp*-Me), 6.2 (Si­(CH_3_)_2_), 4.6 (Si­(CH_3_)_2_), 3.0 (Si­(CH_3_)_2_), 0.2 (Si­(CH_3_)_2_). ^11^B­{^1^H} NMR (160.4 MHz, 305.0 K, C_6_D_6_): δ = 35.6 (C_4_ B-C_6_H_2_(CH_3_)_3_). ^29^Si­{^1^H} INEPT NMR (99.3 MHz, 305.0 K, C_6_D_6_): δ = 0.13, −0.45 (C^1/4^-Si­(C­(CH_3_)_3_)­(CH_3_)_2_).

#### Boragerma­[5]­pyramidane **14d**

Dipotassium 2,5-bis­(*tert*-butyldimethylsilyl)-3,4-dimethylgermolediide K_2_[**13**] (0.22 mmol, 1.00 equiv) was prepared as described before.^16^ The resulting suspension was transferred to a Schlenk flask. A solution of MesBBr_2_ **15d** (64 mg, 0.22 mmol, 1.00 equiv) in Et_2_O (30 mL) was added dropwise to the suspension at room temperature. The reaction mixture was stirred for 16 h. The solvent was removed under reduced pressure and the residue was dissolved in *n*-hexane. After filtration, the solvent was removed under reduced pressure. The residue was dissolved in benzene-d_6_ and analyzed by NMR spectroscopy. Complex **14d** could be isolated as a reddish oil (raw yield: 79 mg, 0.15 mmol, 70%). Several attempts to purify compound **14d** by recrystallization did not succeed.

^1^H NMR (499.9 MHz, 305.0 K, C_6_D_6_): δ = 6.89 (s, 1H, C_6_ H_2_(CH_3_)_3_), 6.85 (s, 1H, C_6_ H_2_(CH_3_)_3_), 2.58 (s, 3H, C_6_H_2_(CH_3_)_3_), 2.26 (s, 3H, C_6_H_2_(CH_3_)_3_), 2.23 (s, 3H, C_6_H_2_(CH_3_)_3_), 2.15 (s, 6H, C^2/3^-CH_3_), 0.92 (s, 18H, C^1/4^-Si­(C­(CH_3_)_3_)­(CH_3_)_2_), 0.09 (s, 6H, C^1/4^-Si­(C­(CH_3_)_3_)­(CH_3_)_2_), −0.22 (s, 6H, C^1/4^-Si­(C­(CH_3_)_3_)­(CH_3_)_2_). ^13^C­{^1^H} NMR (125.7 MHz, 305.0 K, C_6_D_6_): δ = 141.3 (C_6_H_2_(CH_3_)_3_), 137.4 (C_6_H_2_(CH_3_)_3_), 135.6 (C_6_H_2_(CH_3_)_3_), 133.8 (C^2/3^), 127.7 (C_6_H_2_(CH_3_)_3_), 104.8 (C^1/4^), 28.3 (C^1/4^-Si­(C­(CH_3_)_3_)­(CH_3_)_2_), 27.5 (C_6_H_2_(CH_3_)_3_), 25.3 (C_6_H_2_(CH_3_)_3_), 21.4 (C_6_H_2_(CH_3_)_3_), 18.9 (C^1/4^-Si­(C­(CH_3_)_3_)­(CH_3_)_2_), 16.1 (C^2/3^-CH_3_), −2.3 (C^1/4^-Si­(C­(CH_3_)_3_)­(CH_3_)_2_), −2.4 (C^1/4^-Si­(C­(CH_3_)_3_)­(CH_3_)_2_). ^11^B­{^1^H} NMR (160.4 MHz, 305.0 K, C_6_D_6_): δ = 30.6 (C_4_ B-C_6_H_2_(CH_3_)_3_). ^29^Si­{^1^H} INEPT NMR (99.3 MHz, 305.0 K, C_6_D_6_): δ = −0.3 (C^1/4^-Si­(C­(CH_3_)_3_)­(CH_3_)_2_). HR-MS (30 eV, EI):*m*/*z* (C_27_H_47_^11^B^74^GeSi_2_): calc.: 512.2521; exp.: 512.2512.

#### Boragerma­[5]­pyramidane **14e**

Dipotassium 2,5-bis­(-*tert*-butyldimethylsilyl)-3,4-dimethylgermolediide K_2_[**13**] (0.50 mmol, 1.00 equiv) was prepared as described before.^16^ The resulting suspension was transferred to a Schlenk flask. A solution of ^*i*^Pr_2_NBCl_2_ **15e** (91 mg, 0.50 mmol, 1.00 equiv) in Et_2_O (30 mL) was added dropwise to the suspension at room temperature. The reaction mixture was stirred for 16 h. The solvent was removed under reduced pressure and the residue was dissolved in *n*-hexane. After filtration, the solvent was removed under reduced pressure. Complex **14e** could be isolated as yellow crystals from a saturated *n*-pentane solution (yield: 86 mg, 0.17 mmol, 35%).

^1^H NMR (499.9 MHz, 305.0 K, C_6_D_6_): δ = 3.92 (sept, ^3^ *J*_H,H_ = 6.8 Hz, 2H, N-(CH­(CH_3_)_2_)_2_), 2.15 (s, 6H, C^2/3^-CH_3_), 1.31 (d, ^3^ *J*_H,H_ = 6.8 Hz, 2H, N-(CH­(CH_3_)_2_)_2_), 1.04 (s, 18H, C^1/4^-Si­(C­(CH_3_)_3_)­(CH_3_)_2_), 0.35 (s, 6H, C^1/4^-Si­(C­(CH_3_)_3_)­(CH_3_)_2_), 0.25 (s, 6H, C^1/4^-Si­(C­(CH_3_)_3_)­(CH_3_)_2_). ^13^C­{^1^H} NMR (125.7 MHz, 305.0 K, C_6_D_6_): δ = 129.1 (C^2/3^), 89.8 (C^1/4^), 50.2 (N-(CH­(CH_3_)_2_)_2_), 29.1 (C^1/4^-Si­(C­(CH_3_)_3_)­(CH_3_)_2_), 25.7 (N-(CH­(CH_3_)_2_)_2_), 19.5 (C^1/4^-Si­(C­(CH_3_)_3_)­(CH_3_)_2_), 17.0 (C^2/3^-CH_3_), 0.8 (C^1/4^-Si­(C­(CH_3_)_3_)­(CH_3_)_2_), 0.7 (C^1/4^-Si­(C­(CH_3_)_3_)­(CH_3_)_2_). ^11^B­{^1^H} NMR (160.4 MHz, 305.0 K, C_6_D_6_): δ = 33.8 (C_4_ B-N^*i*^Pr_2_) ^29^Si­{^1^H} INEPT NMR (99.3 MHz, 305.0 K, C_6_D_6_): δ = −1.5 (C^1/4^-Si­(C­(CH_3_)_3_)­(CH_3_)_2_). HR-MS (30 eV, EI):*m*/*z* (C_24_H_50_^11^B^74^GeNSi_2_): calc.:493.2787, exp.: 493.2783.

#### Boragerma­[5]­pyramidane **14f**

Dipotassium 2,5-bis­(-*tert*-butyldimethylsilyl)-3,4-dimethylgermolediide K_2_[**13**] (0.50 mmol, 1.00 equiv) was prepared as described before.^16^ The resulting suspension was transferred to a Schlenk flask. A solution of Cy_2_NBCl_2_ **15f** (131 mg, 0.50 mmol, 1.00 equiv) in Et_2_O (30 mL) was added dropwise to the suspension at room temperature. The reaction mixture was stirred for 16 h. The solvent was removed under reduced pressure and the residue was dissolved in *n*-hexane. After filtration, the solvent was removed under reduced pressure. Complex **14f** could be isolated as yellow crystals from a saturated *n*-pentane solution (yield: 166 mg, 0.29 mmol, 58%).

^1^H NMR (499.9 MHz, 305.0 K, C_6_D_6_): δ = 3.23–3.17 (m, 2H, N­(CH-C_5_H_10_)_2_), 2.30–2.28 (m, 4H, N­(CH-C_5_ H_10_)_2_), 2.13 (s, 6H, C^2/3^-CH_3_), 1.78–1.75 (m, 4H, N­(CH-C_5_ H_10_)_2_), 1.63–1.60 (m, 2H, N­(CH-C_5_ H_10_)_2_), 1.46–1.28 (m, 8H, N­(CH-C_5_ H_10_)_2_), 1.19–1.10 (m, 2H, N­(CH-C_5_ H_10_)_2_), 1.05 (s, 18H, C^1/4^-Si­(C­(CH_3_)_3_)­(CH_3_)_2_), 0.40 (s, 6H, C^1/4^-Si­(C­(CH_3_)_3_)­(CH_3_)_2_), 0.37 (s, 6H, C^1/4^-Si­(C­(CH_3_)_3_)­(CH_3_)_2_). ^13^C­{^1^H} NMR (125.7 MHz, 305.0 K, C_6_D_6_): δ = 129.6 (C^2/3^), 102.4 (C^1/4^), 57.8 (N­(CH–C_5_H_10_)_2_), 36.2 (N­(CH-C_5_H_10_)_2_), 29.3 (C^1/4^-Si­(C­(CH_3_)_3_)­(CH_3_)_2_), 27.0 (N­(CH-C_5_H_10_)_2_), 27.0 (N­(CH-C_5_H_10_)_2_), 19.5 (C^1/4^-Si­(C­(CH_3_)_3_)­(CH_3_)_2_), 16.7 (C^2/3^-CH_3_), −0.4 (C^1/4^-Si­(C­(CH_3_)_3_)­(CH_3_)_2_), −0.5 (C^1/4^-Si­(C­(CH_3_)_3_)­(CH_3_)_2_). ^11^B­{^1^H} NMR (160.4 MHz, 305.0 K, C_6_D_6_): δ = 32.1 (C_4_ B-NCy_2_). ^29^Si­{^1^H} INEPT NMR (99.3 MHz, 305.0 K, C_6_D_6_): δ = −2.1 (C^1/4^-Si­(C­(CH_3_)_3_)­(CH_3_)_2_). HR-MS (30 eV, EI): *m*/*z* (C_30_H_58_^11^B^74^GeNSi_2_): calc.: 573.3413, exp.: 573.3407.

#### Boragerma­[5]­pyramidane **14g**

Dipotassium 2,5-bis­(*tert*-butyldimethylsilyl)-3,4-dimethylgermolediide K_2_[**13**] (0.22 mmol, 1.00 equiv) was prepared as described before.^16^ The resulting suspension was transferred to a Schlenk flask. A solution of FcBBr_2_ **15g** (79 mg, 0.22 mmol, 1.00 equiv) in Et_2_O (30 mL) was added dropwise to the suspension at room temperature. The reaction mixture was stirred for 16 h. The solvent was removed under reduced pressure and the residue was dissolved in *n*-hexane. After filtration, the solvent was removed under reduced pressure. Compound **14g** could be isolated as red crystals from a saturated *n*-pentane solution (yield: 69 mg, 0.11 mmol, 51%).

^1^H NMR (499.9 MHz, 305.0 K, C_6_D_6_): δ = 4.66–4.64 (m, 2H, C_4_B–C_5_ H_4_), 4.34–4.32 (m, 2H, C_4_B–C_5_ H_4_), 4.13 (s, 5H, Fe–C_5_ H_5_), 2.16 (s, 6H, C^2/3^-CH_3_), 1.15 (s, 18H, C^1/4^-Si­(C­(CH_3_)_3_)­(CH_3_)_2_), 0.43 (s, 6H, C^1/4^-Si­(C­(CH_3_)_3_)­(CH_3_)_2_), 0.26 (s, 6H, C^1/4^-Si­(C­(CH_3_)_3_)­(CH_3_)_2_). ^13^C­{^1^H} NMR (125.7 MHz, 305.0 K, C_6_D_6_): δ = 134.6 (C^2/3^), 101.1 (C^1/4^), 77.6 (C_4_B-C_5_H_4_), 69.4 (Fe-C_5_H_5_), 69.2 (C_4_B-C_5_H_4_), 29.3 (C^1/4^-Si­(C­(CH_3_)_3_)­(CH_3_)_2_), 19.2 (C^1/4^-Si­(C­(CH_3_)_3_)­(CH_3_)_2_), 16.9 (C^2/3^-CH_3_), 2.2 (C^1/4^-Si­(C­(CH_3_)_3_)­(CH_3_)_2_), 2.2 (C^1/4^-Si­(C­(CH_3_)_3_)­(CH_3_)_2_). The ^13^C NMR signal of the α-carbon atom bonded to the boron atom could not be determined. ^11^B­{^1^H} NMR (160.4 MHz, 305.0 K, C_6_D_6_): δ = 32.4 (C_4_ B-Fc). ^29^Si­{^1^H} INEPT NMR (99.3 MHz, 305.0 K, C_6_D_6_): δ = −0.4 (C^1/4^-Si­(C­(CH_3_)_3_)­(CH_3_)_2_). HR-MS (30 eV, EI): *m*/*z* (C_28_H_45_^11^B^56^Fe^74^GeSi_2_): calc.:578.1714, exp.: 578.1709.

#### Borasila­[5]­pyramidane **20f**

Dipotassium 2,5-bis­(trimethylsilyl)-3,4-diphenylsilolediide K_2_[**19**] (0.50 mmol, 1.00 equiv) was prepared as described before.^16^ The resulting suspension was transferred to a Schlenk flask. A solution of Cy_2_NBCl_2_ **15f** (131 mg, 0.50 mmol, 1.00 equiv) in Et_2_O (30 mL) was added dropwise to the suspension at room temperature. The reaction mixture was stirred for 16 h. The solvent was removed under reduced pressure and the residue was dissolved in *n*-hexane. After filtration, the solvent was removed under reduced pressure. The residue was dissolved in benzene-d_6_ and analyzed by NMR spectroscopy. Complex **20f** could be isolated as a dark-yellow oil (raw yield: 218 mg, 0.38 mmol, 77%). Several attempts to purify compound **20f** by recrystallization did not succeed.

^1^H NMR (499.9 MHz, 305.0 K, C_6_D_6_): δ = 7.21–7.19 (m, 4H, C_6_ H_5_), 6.86–6.84 (m, 6H, C_6_ H_5_), 3.58–3.52 (m, 2H, N­(CH-C_5_H_10_)_2_), 2.23–2.21 (m, 4H, N­(CH-C_5_H_10_)_2_), 1.91–1.08 (m, 16H, N­(CH-C_5_H_10_)_2_), 0.12 (s, 18H, C^1/4^-Si­(CH_3_)_3_). ^13^C­{^1^H} NMR (125.7 MHz, 305.0 K, C_6_D_6_): δ = 136.8 (C^2/3^), 135.9 (C^*ipso*^), 132.2 (C_6_H_5_), 127.7 (C_6_H_5_), 127.6 (C_6_H_5_), 88.7 (C^1/4^), 59.4 (N­(CH–C_5_H_10_)_2_), 36.8 (N­(CH-C_5_H_10_)_2_), 27.4 (N­(CH-C_5_H_10_)_2_), 26.7 (N­(CH-C_5_H_10_)_2_), 2.7 (C^1/4^-Si­(CH_3_)_3_). ^11^B­{^1^H} NMR (160.4 MHz, 305.0 K, C_6_D_6_): δ = 35.0 (C_4_ B-NCy_2_). ^29^Si­{^1^H} NMR (99.3 MHz, 305.0 K, C_6_D_6_): δ = −9.4 (C^1/4^-Si­(CH_3_)_3_), −337.5 (Si­(II)). Note: No satisfactory results could be obtained for Mass Spectrometry (MS) or Elemental Analysis (EA).

#### Ferrocenyl-Substituted Borole **25**

Dipotassium 2,5-bis­(-*tert*-butyldimethylsilyl)-3,4-dimethylstannolediide K_2_[**35**] (0.30 mmol, 1.00 equiv) was prepared following a literature procedure.^45^ The resulting suspension was transferred to a Schlenk flask. A solution of FcBBr_2_ 15g (107 mg, 0.30 mmol, 1.00 equiv) in Et_2_O (40 mL) was added slowly via a *Teflon* tube at −80 °C. The reaction mixture was stirred for 2 h while the cooling bath was allowed to warm to room temperature. Afterward, it was stirred at room temperature for 1 h. The solvent was removed under reduced pressure and the residue was dissolved in *n*-pentane. After filtration, the solvent was removed under reduced pressure and the crude product **25** was obtained as red oily substance (yield: 144 mg, 0.29 mmol, 95%). Red crystals of compound **25** could be isolated from a saturated *n*-pentane solution at −30 °C (yield: 84 mg, 0.17 mmol, 55%).

^1^H NMR (499.9 MHz, 298.1 K, C_6_D_6_): δ = 4.74–4.66 (m, 2H, C_4_B–C_5_ H_4_), 4.53–4.46 (m, 2H, C_4_B–C_5_ H_4_), 4.01 (s, 5H, Fe–C_5_ H_5_), 1.99 (s, 6H, C^2/3^-CH_3_), 1.11 (s, 18H, C^1/4^-Si­(C­(CH_3_)_3_)­(CH_3_)_2_), 0.35 (s, 12H, C^1/4^-Si­(C­(CH_3_)_3_)­(CH_3_)_2_). ^13^C­{^1^H} NMR (125.7 MHz, 298.1 K, C_6_D_6_): δ = 169.9 (C^2/3^), 147.9 (br, C^1/4^), 80.3 (C_4_B-C_5_H_4_), 75.1 (C_4_B-C_5_H_4_), 71.2 (Fe-C_5_H_5_), 28.8 (C^1/4^-Si­(C­(CH_3_)_3_)­(CH_3_)_2_), 20.6 (C^2/3^-CH_3_), 19.6 (C^1/4^-Si­(C­(CH_3_)_3_)­(CH_3_)_2_), −0.8 (C^1/4^-Si­(C­(CH_3_)_3_)­(CH_3_)_2_). The ^13^C NMR signal of the α-carbon atom bonded to the boron atom could not be determined. ^11^B­{^1^H} NMR (160.4 MHz, 298.1 K, C_6_D_6_): δ = 66.4 (C_4_ B-Fc). ^29^Si­{^1^H} INEPT NMR (99.3 MHz, 298.1 K, C_6_D_6_): δ = −3.7 (C^1/4^-Si­(C­(CH_3_)_3_)­(CH_3_)_2_). HR-MS (30 eV, EI): *m*/*z* (C_28_H_45_^11^B^56^FeSi_2_): calc.: 504.2497, exp.: 504.2507.

#### Tungsten Complex **30**

At room temperature, a mixture of boragerma[5]­pyramidane **12e** (204 mg, 0.50 mmol, 1.00 equiv) and tungsten hexacarbonyl (176 mg, 0.50 mmol, 1.00 equiv) in THF (15 mL) was irradiated with a LED (370 nm) for 4 h. After that, the solvent was removed under reduced pressure. After crystallization from *n*-pentane at T = −24 °C, the product **30** (293 mg, 0.40 mmol, 80%) was obtained as orange crystals suitable for sc-XRD analysis.

^1^H NMR (499.9 MHz, 298.0 K, C_6_D_6_): δ = 3.97 (sept, ^3^ *J*_H,H_ = 7.1 Hz, 2H, N­(CH­(CH_3_)_2_)_2_), 2.13 (s, 6 H, C^2/3^-CH_3_), 1.12 (d, ^3^ *J*_H,H_ = 7.1 Hz, 12 H, N­(CH­(CH_3_)_2_)_2_), 0.24 (s, 18 H, C^1/4^-Si­(CH_3_)).

^13^C­{^1^H} NMR (125.7 MHz, 298.0 K, C_6_D_6_): δ = 196.6 (CO^eq^, ^1^ *J*_C,W_ = 124.7 Hz), 196.5 (CO^ax^, ^1^ *J*_C,W_ = 161.8 Hz), 132.7 (C^2/3^), 75.1 (C^1/4^), 50.2 (N­(CH­(CH_3_)_2_)_2_), 25.7 (N-(CH­(CH_3_)_2_)_2_), 15.3 (C^2/3^-CH_3_), 2.3 (C^1/4^-Si­(CH_3_)_3_). ^11^B­{^1^H} NMR (160.4 MHz, 298.0 K, C_6_D_6_): δ = 36.9 (C_4_ B-N^*i*^Pr_2_). ^29^Si­{^1^H} INEPT NMR (99.3 MHz, 298.0 K, C_6_D_6_): δ = −8.2 (C^1/4^-Si­(CH_3_)_3_). IR (ATR, neat): 2072, 1977, 1915 cm^–1^.

#### Dilithium Boracyclopentadienediide Diethyl Ether Solvate [Li­(Et_2_O)]_2_[**29**]

Lithium (35 mg, 5.00 mmol, 10.00 equiv) was added to a solution of boragerma[5]­pyramidane **12e** (204 mg, 0.50 mmol, 1.00 equiv) in Et_2_O (10 mL). The reaction mixture was stirred for 16 h at room temperature. After that, the reaction mixture was filtered through a frit and the solvent was removed under reduced pressure. The product [Li­(Et_2_O)]_2_[**29**] (197 mg, 0.39 mmol, 79%) was obtained as a green oily substance. Several attempts to crystallize compound [Li­(Et_2_O)]_2_[**29**] did not succeed. Therefore, no satisfactory combustion analysis or HR-MS was obtained.

^1^H NMR (499.9 MHz, 298.0 K, C_6_D_6_): δ = 3.72 (sept, ^3^ *J*_H,H_ = 6.3 Hz, 2H, N­(CH­(CH_3_)_2_)_2_), 3.04 (q, ^3^ *J*_H,H_ = 7.0 Hz, 8H, 2 x O­(CH_2_CH_3_)_2_), 2.36 (s, 6H, C^2/3^-CH_3_), 1.34 (d, ^3^ *J*_H,H_ = 6.4 Hz, 12H, N­(CH­(CH_3_)_2_)_2_), 0.81 (t, ^3^ *J*_H,H_ = 7.0 Hz, 12H, 2 x O­(CH_2_CH_3_)_2_), 0.49 (s, 18H, C^1/4^-Si­(CH_3_)_3_).

^13^C­{^1^H} NMR (125.7 MHz, 298.0 K, C_6_D_6_): δ = 117.5 (C^2/3^), 98.1 (C^1/4^), 65.2 (O­(CH_2_CH_3_)_2_), 48.9 (N­(CH­(CH_3_)_2_)_2_), 26.1 (N­(CH­(CH_3_)_2_)_2_), 16.6 (C^2/3^-CH_3_), 14.5 (O­(CH_2_ CH_3_)_2_), 5.2 (C^1/4^-Si­(CH_3_)_3_). ^11^B­{^1^H} NMR (160.4 MHz, 298.0 K, C_6_D_6_): δ = 32.4 (C_4_ B-N^*i*^Pr_2_). ^29^Si­{^1^H} INEPT NMR (99.3 MHz, 298.0 K, C_6_D_6_): δ = −14.7 (C^1/4^-Si­(CH_3_)_3_). ^7^Li­{^1^H} NMR (194.3 MHz, 298.0 K, C_6_D_6_): δ = −6.3 (((H_3_CH_2_C)_2_O)Li).

#### Dilithium Boracyclopentadienediide Tetrahydrofuran Solvate [Li­(THF)]_2_[**29**]

Lithium (35 mg, 5.00 mmol, 10.00 equiv) was added to a solution of boragerma[5]­pyramidane **12e** (204 mg, 0.50 mmol, 1.00 equiv) in THF (10 mL). The reaction mixture was stirred for 16 h at room temperature. After that, the reaction mixture was filtered through a frit and the solvent was removed under reduced pressure. The product [Li­(THF)]_2_[**29**] (183 mg, 0.37 mmol, 74%) was obtained as a green oily substance. Several attempts to crystallize compound [Li­(THF)]_2_[**29**] did not succeed.

^1^H NMR (499.9 MHz, 298.0 K, C_6_D_6_): δ = 3.79 (sept, ^3^ *J*_H,H_ = 6.3 Hz, 2H, N­(CH­(CH_3_)_2_)_2_), 3.30–3.19 (m, 8H, 2 x C_4_ H_8_O), 2.45 (s, 6H, C^2/3^-CH_3_), 1.38 (d, ^3^ *J*_H,H_ = 6.4 Hz, 12H, N­(CH­(CH_3_)_2_)_2_), 1.18–1.13 (m, 8H, 2 x C_4_ H_8_O), 0.55 (s, 18H, C^1/4^-Si­(CH_3_)_3_). ^13^C­{^1^H} NMR (125.7 MHz, 298.0 K, C_6_D_6_): δ = 117.8 (C^2/3^), 98.8 (C^1/4^), 68.7 (C_4_H_8_O), 48.3 (N­(CH­(CH_3_)_2_)_2_), 25.9 (N­(CH­(CH_3_)_2_)_2_), 25.2 (C_4_H_8_O), 16.4 (C^2/3^-CH_3_), 5.0 (C^1/4^-Si­(CH_3_)_3_). ^11^B­{^1^H} NMR (160.4 MHz, 298.0 K, C_6_D_6_): δ = 31.5 (C_4_ B-N^*i*^Pr_2_). ^29^Si­{^1^H} INEPT NMR (99.3 MHz, 298.0 K, C_6_D_6_): δ = −14.3 (C^1/4^-Si­(CH_3_)_3_). ^7^Li­{^1^H} NMR (194.3 MHz, 298.0 K, C_6_D_6_): δ = −6.2 ((C_4_H_8_O)Li). HR-MS (30 eV, EI): *m*/*z* (C_26_H_54_^11^B^7^Li_2_NO_2_Si_2_): calc.:493.4106; exp.:493.4104.

### Computational Details

All quantum chemical calculations were performed using the Gaussian16 software package.^26^ For the Natural Bond Orbital (NBO) analyses,^46^ the NBO 7.0 program was used,^47^ and the Jmol16 program was used for graphical representation.^48^ The Quantum Theory of Atoms In Molecules (QTAIM) analyses were performed using the AIMALL program.^49^ The optimizations were performed using the hybrid functional M06-2X^50^ and the 6-311+G­(d,p) basis set or the Def2-TZVP basis set. The SCF energies and Gibbs free energies (at T = 298.15 K and p = 0.101 MPa (isolated molecule)) of all optimized molecular structures are listed in Tables S11 and S12. Subsequent frequency calculations determined the stationary points either as a minimum (number of imaginary frequencies NImag = 0) or as a transition state (NImag = 1). NMR chemical shift calculations were carried out using the GIAO method with the functional M06-L^51^ and the 6-311G­(2d,p) basis set for molecular structures optimized at the M06-2X/6-311+G­(d,p) level of theory.

## Supplementary Material







## Notes

Experimental data of synthesized starting compounds, analytical data of all characterized compounds, NMR spectra of all characterized compounds, summary of sc-XRD results, and tables of absolute electronic and free energies of all optimized structures (PDF)
DFT-optimized molecular structures (XYZ)
DFT-optimized molecular structures (XYZ)
