# Supplementary material for: Boragerma[5]pyramidanes via a Germole-to-Borole Rearrangement
Source: Inorg Chem. 2026 Mar 24;65(13):7322–32. doi: 10.1021/acs.inorgchem.6c00260 (PMC13058896; doi:10.1021/acs.inorgchem.6c00260)
Supplement: Supplementary file 1 [file ic6c00260_si_001.pdf]

Supporting Information

for

Boragerma[5]pyramidanes via a Germole-to-Borole Rearrangement

Lukas Bührmann,[a] Amrit Chandi,[a] Nadeschda Geibel,[a] Lena Albers,[a] Marc Schmidtmann[a] and Thomas Müller*[a]

Institute of Chemistry, Carl von Ossietzky University Oldenburg

Carl von Ossietzky-Straße 9-11, D-26129 Oldenburg, Federal Republic of Germany, European Union

* corresponding author:, e-mail: thomas.mueller@uni-oldenburg.de

Table of Contents.

Experimental Part	S-2
Crystallographic Data	S-66
Computational Details	S-84
References	S-98

Experimental Part.

General. All manipulations of air- and moisture-sensitive compounds were carried out under an argon 5.0 or nitrogen 5.0 atmosphere using Schlenk techniques or a standard glove box (Braun Unilab). Glassware was dried in an oven at T = 140 °C and evacuated three times prior to use. The solvents THF, diethyl ether, toluene, *n*-pentane and *n*-hexane were dried over Na/K alloy and distilled under an inert gas atmosphere. Benzene-d_6 was dried over potassium and stored over molecular sieves (4 Å). $HNCy_2$ and HN^iPr_2 were dried over CaH_2 and distilled under an inert gas atmosphere prior to use. Dichlorogermoles, dichlorosilole and the mesityl copper complex were prepared according to literature procedures.[1, 2] Ferrocene, boron trichloride, boron tribromide, elemental lithium and tungsten hexacarbonyl complex were purchased commercially and used without any further purification.

NMR spectroscopy. NMR spectra were recorded on Bruker Avance DRX 500, Bruker Avance III 500 and JEOL JNM-ECZL 500 spectrometers. ^1H NMR spectra were calibrated against the residual proton signal of the solvent as internal reference (benzene-d_6: $\delta^1H(C_6D_5H)$ = 7.16; THF/D_2O-capillary: $\delta^1H(C_4H_8O)$ = 3.58). $^{13}C\{^1H\}$ NMR spectra were calibrated by using the central line of the solvent signal (benzene-d_6: $\delta^{13}C(C_6D_6)$ = 128.0; THF/D_2O-capillary: $\delta^{13}C(C_4H_8O)$ = 67.2)). The $^{29}Si\{1H\}$ NMR, $^{11}B\{^1H\}$ NMR, and $^7Li\{^1H\}$ NMR spectra were calibrated against an external standard ($\delta^{29}Si(Me_2SiHCl)$ = 11.1 versus tetramethylsilane (TMS); $\delta^{11}B(BF_3{\cdot}Et_2O)$ = 0.0; $\delta^7Li(LiCl)$ = 0.0). The $^{29}Si\{^1H\}$ INEPT (*Insensitive Nuclei Enhancement by Polarization Transfer*) NMR spectra were recorded with a delay suitable for $SiMe_3$-groups ($n = 9$ and $^2J_{Si,H}$ = 8 Hz). The $^{29}Si\{^1H\}$ NMR spectra were recorded with a relaxation time of d = 10.0 s. For a clear assignment of the signals two-dimensional experiments, such as $^1H^{13}C$ heteronuclear multiple quantum coherence (HMQC) and $^1H^{13}C$ heteronuclear multiple bond coherence (HMBC) spectra were recorded.

Mass Spectrometry. Mass Spectra were recorded with a ThermoScientific DFS – High Resolution Magnetic Sector MS (HV-Emitter: 8 kV, emitter heating current (EHC) Ramp: 21 mA/min, emitter heating current (EHC) max Ramp: 95 mA).

IR Spectroscopy. Infrared spectra were measured and recorded on a Bruker Tensor 27 spectrometer.

Single X-ray diffraction. Single crystal X-ray data were measured on a Bruker AXS D8 Venture diffractometer (multilayer optics, Mo-Kα and Cu-Kα radiation with λ = 0.71073 Å and 1.54178 Å respectively, Kappa 4-circle goniometer, Photon III C14 CPAD detector). All crystals were measured at a temperature of 100 K. Absorption corrections using equivalent reflections were performed with the program SADABS.[3] All structures were solved with the program SHELXS[4] and refined with SHELXL[5] using the OLEX2 GUI.[6] All non-H-atoms were refined using anisotropic atomic displacement parameters (ADPs). H atoms bonded to C were located in the difference Fourier maps and placed on idealized geometric positions with idealized ADPs using the riding model. The crystallographic data can be obtained free of charge from https://www.ccdc.cam.ac.uk/structures/ quoting the CCDC numbers 2520807-2520814.

Synthesis of dipotassium germacyclopentadienediide K_2[11]

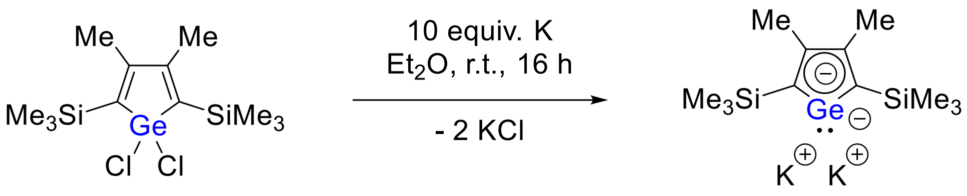

K$_2$[**11**]

The dipotassium 2,5-bis(trimethylsilyl)-3,4-dimethylgermolediide K$_2$[**11**] was synthesized according to a slightly modified literature procedure.[1] A mixture of the dichlorogermole (184 mg, 0.50 mmol, 1.00 equiv.) and potassium (195 mg, 5.00 mmol, 10.00 equiv.) was stirred in Et_2O (6 mL) at room temperature for 16 h. The obtained brown suspension was separated from excess potassium and was used for following reactions without any work-up. To record NMR spectroscopic data, the solvent was once removed and THF was added to the residue and a D_2O-capillary was used.

^{1}H NMR (499.9 MHz, 305.0 K, THF/D_2O-capillary): δ = 2.47 (s, 6H, C$^{2/3}$-C<u>H</u>$_3$), 0.35 (s, 18H, C$^{1/4}$-Si(C<u>H</u>$_3$)$_3$).

^{13}C{^{1}H} NMR (125.7 MHz, 305.0 K, THF/D_2O-capillary): δ = 156.2 (C$^{1/4}$), 130.8 (C$^{2/3}$), 20.9 (C$^{2/3}$-<u>C</u>H$_3$), 4.9 (C$^{1/4}$-Si(<u>C</u>H$_3$)$_3$).

^{29}Si{^{1}H} INEPT NMR (99.3 MHz, 305.0 K, THF/D_2O-capillary): δ = -15.9 (C$^{1/4}$-<u>Si</u>(CH$_3$)$_3$).

Synthesis of dipotassium germacyclopentadienediide K_2[13]

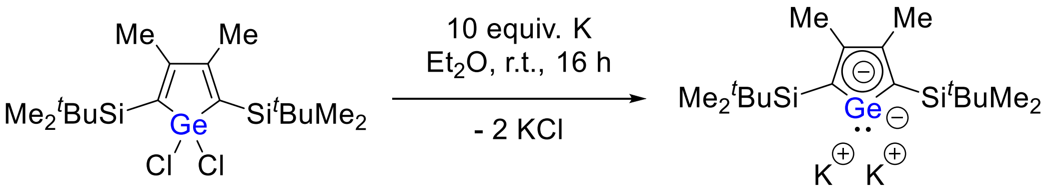

K$_2$[**13**]

The dipotassium 2,5-bis(*tert*-butyldimethylsilyl)-3,4-dimethylgermolediide K$_2$[**13**] was synthesized according to a slightly modified literature procedure.[1] A mixture of the dichlorogermole (226 mg, 0.50 mmol, 1.00 equiv.) and potassium (195 mg, 5.00 mmol, 10.00 equiv.) was stirred in Et_2O (6 mL) at room temperature for 16 h. The obtained brown

suspension was separated from excess potassium and was used for following reactions without any work-up. To record NMR spectroscopic data, the solvent was once removed and THF was added to the residue and a D_2O-capillary was used.

^{1}H NMR (499.9 MHz, 305.0 K, THF/D_2O-capillary): δ = 2.21 (s, 6H, $C^{2/3}$-C$\underline{H}_3$), 0.95 (s, 18H, $C^{1/4}$-Si(C(C$\underline{H}_3$)$_3$)(CH$_3$)$_2$), 0.15 (s, 12H, $C^{1/4}$-Si(C(CH$_3$)$_3$)(C$\underline{H}_3$)$_2$).

^{13}C{^{1}H} NMR (125.7 MHz, 305.0 K, THF/D_2O-capillary): δ = 150.8 ($C^{1/4}$), 129.6 ($C^{2/3}$), 28.3 ($C^{1/4}$-Si(C($\underline{C}$H$_3$)$_3$)(CH$_3$)$_2$), 21.6 ($C^{2/3}$-$\underline{C}$H$_3$), 17.5 ($C^{1/4}$-Si($\underline{C}$(CH$_3$)$_3$)(CH$_3$)$_2$), 0.0 ($C^{1/4}$-Si(C(CH$_3$)$_3$)($\underline{C}$H$_3$)$_2$).

^{29}Si{^{1}H} INEPT NMR (99.3 MHz, 305.0 K, THF/D_2O-capillary): δ = -5.9 ($C^{1/4}$-$\underline{Si}$(C(CH$_3$)$_3$)(CH$_3$)$_2$).

Synthesis of dipotassium silacyclopentadienediide K_2[19]

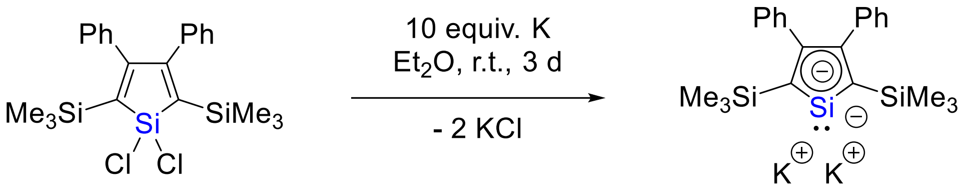

K_2[**19**]

The dipotassium 2,5-bis(trimethylsilyl)-3,4-diphenylsilolediide K_2[**19**] was synthesized according to a slightly modified literature procedure.[1] A mixture of the dichlorosilole (223 mg, 0.50 mmol, 1.00 equiv.) and potassium (195 mg, 5.00 mmol, 10.00 equiv.) was stirred in Et_2O (6 mL) at room temperature for 3 d. The obtained brown suspension was separated from excess potassium and was used for following reactions without any work-up. To record NMR spectroscopic data, the solvent was once removed and THF was added to the residue and a D_2O-capillary was used.

^{1}H NMR (499.9 MHz, 305.0 K, THF/D_2O-capillary): δ = 7.25-7.22 (m, 8H, $C^{2/3}$-C$_6\underline{H}_5$), 7.09-7.07 (m, 2H, $C^{2/3}$-C$_6\underline{H}_5$), 0.39 (s, 18H, $C^{1/4}$- Si(C$\underline{H}_3$)$_3$).

^{13}C{^{1}H} NMR (125.7 MHz, 305.0 K, THF/D_2O-capillary): δ = 149.9 ($C^{2/3}$-$\underline{C}_6$H$_5$), 145.8 ($C^{1/4}$), 140.5 ($C^{2/3}$), 130.4 ($C^{2/3}$-$\underline{C}_6$H$_5$), 126.1 ($C^{2/3}$-$\underline{C}_6$H$_5$), 121.1 ($C^{2/3}$-$\underline{C}_6$H$_5$), 5.6 ($C^{1/4}$- Si($\underline{C}$H$_3$)$_3$).

^{29}**Si{^{1}H} NMR** (99.3 MHz, 305.0 K, THF/D_2O-capillary): δ = 148.5 ($C_4\underline{Si}$), -15.8 ($C^{1/4}$- $\underline{Si}(CH_3)_3$).

Synthesis of $MesBBr_2$ 15d

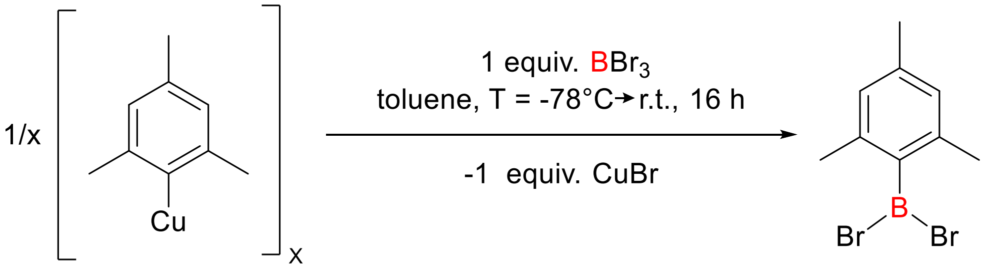

15d

Mesityl dibromoborane **15d** was synthesized according to a literature procedure.[2] At T = -78 °C, a solution of BBr_3 (2.88 g, 11.49 mmol, 1.05 equiv.) in toluene (10 mL) was added dropwise to a suspension of the mesityl copper complex (2.00 g, 10.95 mmol, 1.00 equiv.) in toluene (25 mL). While adding, the color of the suspension changed from yellow to orange-red. After 2 h of stirring of the reaction mixture at T = -78 °C, it was stirred for another 16 h while warming to room temperature. The precipitate was filtered off and the solvent of the filtrate was removed under reduced pressure. After distillation (p = 0.1 Pa, T = 130-135 °C), the product **15d** was obtained as colorless oil.

^{1}**H NMR** (499.9 MHz, 305.0 K, C_6D_6): δ = 6.54 (s, 2H, $Br_2B(C_6\underline{H}_2(CH_3)_3)$), 2.15 (s, 6H, $Br_2B(C_6H_2(C\underline{H}_3)_3)$), 2.04 (s, 3H, $Br_2B(C_6H_2(C\underline{H}_3)_3)$).

^{13}**C{^{1}H} NMR** (125.7 MHz, 305.0 K, C_6D_6): δ = 140.0 ($Br_2B(\underline{C}_6H_2(CH_3)_3)$), 136.1 ($Br_2B(\underline{C}_6H_2(CH_3)_3)$), 128.3 ($Br_2B(\underline{C}_6H_2(CH_3)_3)$), 21.8 ($Br_2B(C_6H_2(\underline{C}H_3)_3)$), 21.1 ($Br_2B(C_6H_2(\underline{C}H_3)_3)$).

The ^{13}C NMR signal of the *ipso*-carbon atom bonded to the boron atom could not be determined.

^{11}**B{^{1}H} NMR** (160.4 MHz, 305.0 K, C_6D_6): δ = 62.6 ($Br_2\underline{B}(C_6H_2(CH_3)_3)$).

Synthesis of $FcBBr_2$ 15g

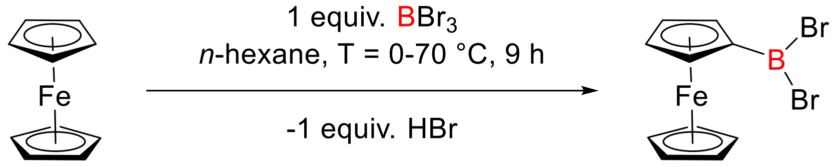

15g

Ferrocenyl dibromoborane **15g** was synthesized according to a modified literature procedure.[7] At T = 0 °C, a solution of BBr_3 (5.00 g, 19.96 mmol, 1.00 equiv.) in *n*-hexane (30 mL) was added dropwise to a suspension of ferrocene (3.71 g, 19.96 mmol, 1.00 equiv.) in *n*-hexane (80 mL). After addition, the reaction mixture was stirred and heated to T = 69 °C for 9 h. The resulting red suspension was cooled to room temperature, the precipitate was filtered off and the solvent was removed under reduced pressure. After crystallisation at T = -30 °C from *n*-hexane, the product **15g** (6.01 g, 16.90 mmol, 85%) was obtained as a red solid.

^{1}H NMR (499.9 MHz, 305.0 K, C_6D_6): δ = 4.38 (s, 4H, Fe($C_5\underline{H}_4BBr_2$)), 3.89 (s, 5H, Fe($C_5\underline{H}_5$)).

^{13}C{^{1}H} NMR (125.7 MHz, 305.0 K, C_6D_6): δ = 78.5 (Fe($\underline{C}_5H_4BBr_2$)), 77.6 (Fe($\underline{C}_5H_4BBr_2$)), 71.8 (Fe($\underline{C}_5H_5$)).
The ^{13}C NMR signal of the *α*-carbon atom bonded to the boron atom could not be determined.

^{11}B{^{1}H} NMR (160.4 MHz, 305.0 K, C_6D_6): δ = 46.2 (Fe($C_5H_4\underline{B}Br_2$)).

Synthesis of $^{i}Pr_2NBCl_2$ 15e

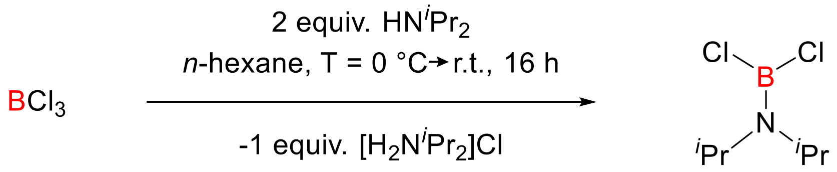

15e

Aminodichloroborane **15e** was synthesized according to a modified literature procedure.[8] At T = 0 °C, a solution of $HN^{i}Pr_2$ (3.16 g, 31.22 mmol, 2.00 equiv.) in n-hexane (60 mL) was added dropwise to a solution of BCl_3 (1.83 g, 15.61 mmol, 1.00 equiv.) in *n*-hexane (150 mL). Afterwards, the resulting colorless reaction mixture was stirred for 16 h while warming to room temperature. A colorless precipitate was filtered off and the solvent of the filtrate was removed

under reduced pressure. After distillation (p = 0.1 Pa, T = 30-35 °C), the product **15e** (2.44 g, 13.42 mmol, 86%) was obtained as colorless oil, that was stored at T = -24 °C.

^{1}H NMR (499.9 MHz, 305.0 K, C_6D_6): δ = 3.78-3.49 (m, 2H, $Cl_2BN(C\underline{H}(CH_3)_2)_2$), 1.08-0.84 (m, 12H, $Cl_2BN(CH(C\underline{H}_3)_2)_2$).

^{13}C{^{1}H} NMR (125.7 MHz, 305.0 K, C_6D_6): δ = 49.0 ($Cl_2BN(\underline{C}H(CH_3)_2)_2$), 21.8 ($Cl_2BN(CH(\underline{C}H_3)_2)_2$).

^{11}B{^{1}H} NMR (160.4 MHz, 305.0 K, C_6D_6): δ = 30.4 ($Cl_2\underline{B}N(CH(CH_3)_2)_2$).

Synthesis of Cy_2NBCl_2 15f

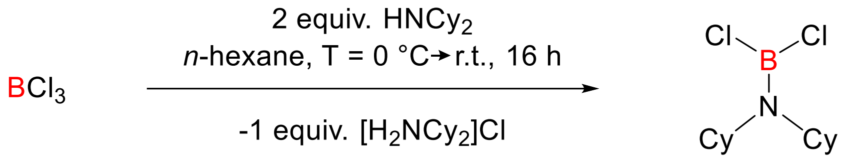

15f

Aminodichloroborane **15f** was synthesized according to a modified literature procedure.[9] At T = 0 °C, a solution of $HNCy_2$ (4.47 g, 23.88 mmol, 2.00 equiv.) in n-hexane (60 mL) was added dropwise to a solution of BCl_3 (1.40 g, 11.94 mmol, 1.00 equiv.) in *n*-hexane (150 mL). Afterwards, the resulting colorless reaction mixture was stirred for 16 h while warming to room temperature. A colorless precipitate was filtered off and the solvent of the filtrate was removed under reduced pressure. The product **15f** (2.44 g, 13.42 mmol, 86%) was obtained as yellowish solid.

^{1}H NMR (499.9 MHz, 305.0 K, C_6D_6): δ = 1.59-1.44 (m, 14H, $Cl_2B(N(C_6\underline{H}_{11})_2)$), 1.13-0.86 (m, 8H, $Cl_2B(N(C_6\underline{H}_{11})_2)$.

^{13}C{^{1}H} NMR (125.7 MHz, 305.0 K, C_6D_6): δ = 59.0 (br, $Cl_2B(N(\underline{C}_6H_{11})_2)$, 32.3 (br, $Cl_2B(N(\underline{C}_6H_{11})_2)$, 26.6 (br, $Cl_2B(N(\underline{C}_6H_{11})_2)$, 25.6 ($Cl_2B(N(\underline{C}_6H_{11})_2)$.

^{11}B{^{1}H} NMR (160.4 MHz, 305.0 K, C_6D_6): δ = 31.2 ($Cl_2\underline{B}(N(C_6H_{11})_2)$.

Synthesis of Mes-substituted boragerma[5]pyramidane 12d

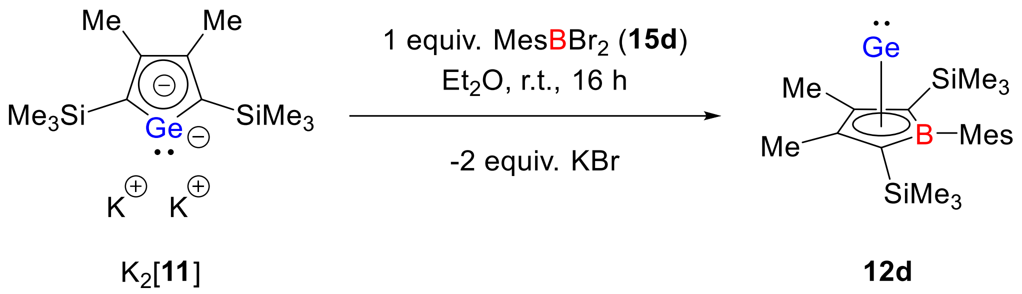

Dipotassium 2,5-bis(trimethylsilyl)-3,4-dimethylgermolediide K_2[**11**] (0.50 mmol, 1.00 equiv.) was prepared as described before. The resulting suspension was transferred to a Schlenk flask. A solution of $MesBBr_2$ **15d** (145 mg, 0.50 mmol, 1.00 equiv.) in Et_2O (30 mL) was added dropwise to the suspension at room temperature. The reaction mixture was stirred for 16 h. The solvent was removed under reduced pressure and the residue was dissolved in *n*-hexane. After filtration, the solvent was removed under reduced pressure. The residue was dissolved in benzene-d_6 and analysed by NMR spectroscopy. Complex **12d** could be isolated as a reddish oil (raw yield: 165 mg, 0.39 mmol, 77%). Several attempts to purify compound **12d** by recrystallisation did not succeed.

^{1}H NMR (499.9 MHz, 305.0 K, C_6D_6): δ = 6.88 (s, 2H, $C_6\underline{H}_2(CH_3)_3$), 2.50 (s, 3H, $C_6H_2(C\underline{H}_3)_3$), 2.28 (s, 3H, $C_6H_2(C\underline{H}_3)_3$), 2.24 (s, 3H, $C_6H_2(C\underline{H}_3)_3$), 2.09 (s, 6H, $C^{2/3}$-$C\underline{H}_3$), 0.00 (s, 18H, $C^{1/4}$-$Si(C\underline{H}_3)_3$).

^{13}C{^{1}H} NMR (125.7 MHz, 305.0 K, C_6D_6): δ = 140.9 ($\underline{C}_6H_2(CH_3)_3$), 137.4 ($\underline{C}_6H_2(CH_3)_3$), 135.4 ($\underline{C}_6H_2(CH_3)_3$), 132.2 ($C^{2/3}$), 127.7 ($\underline{C}_6H_2(CH_3)_3$), 107.0 ($C^{1/4}$), 28.2 ($C_6H_2(\underline{C}H_3)_3$), 24.8 ($C_6H_2(\underline{C}H_3)_3$), 21.4 ($C_6H_2(\underline{C}H_3)_3$), 14.4 ($C^{2/3}$-$\underline{C}H_3$), 1.3 ($C^{1/4}$-$Si(\underline{C}H_3)_3$).

^{11}B{^{1}H} NMR (160.4 MHz, 305.0 K, C_6D_6): δ = 29.4 ($C_4\underline{B}$-$C_6H_2(CH_3)_3$).

^{29}Si{^{1}H} INEPT NMR (99.3 MHz, 305.0 K, C_6D_6): δ = -8.2 ($C^{1/4}$-$\underline{Si}(CH_3)_3$).

HR-MS (30 eV, EI): m/z = calc.: 428.1582 $C_{21}H_{35}BGeSi_2$

exp.: 428.1577

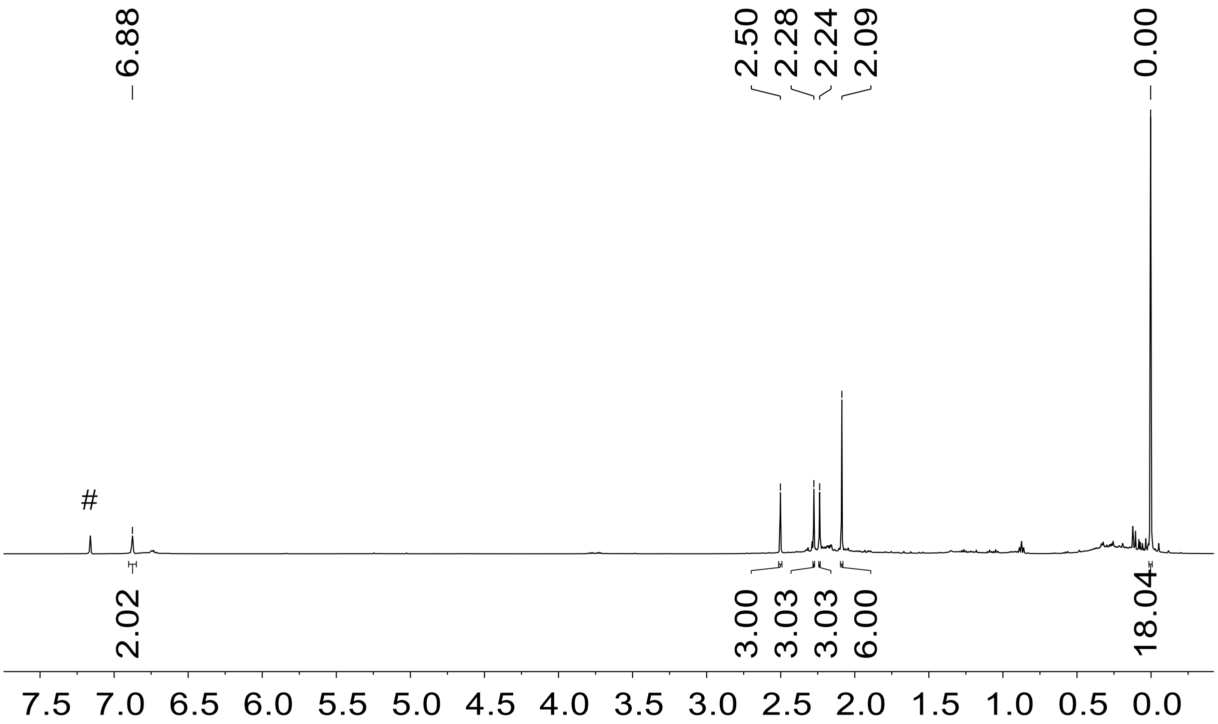

Figure S1. ^{1}H NMR spectrum (499.9 MHz, 305.0 K, C_6D_6) of Mes-substituted boragerma[5]pyramidane **12d**, # = C_6D_5H.

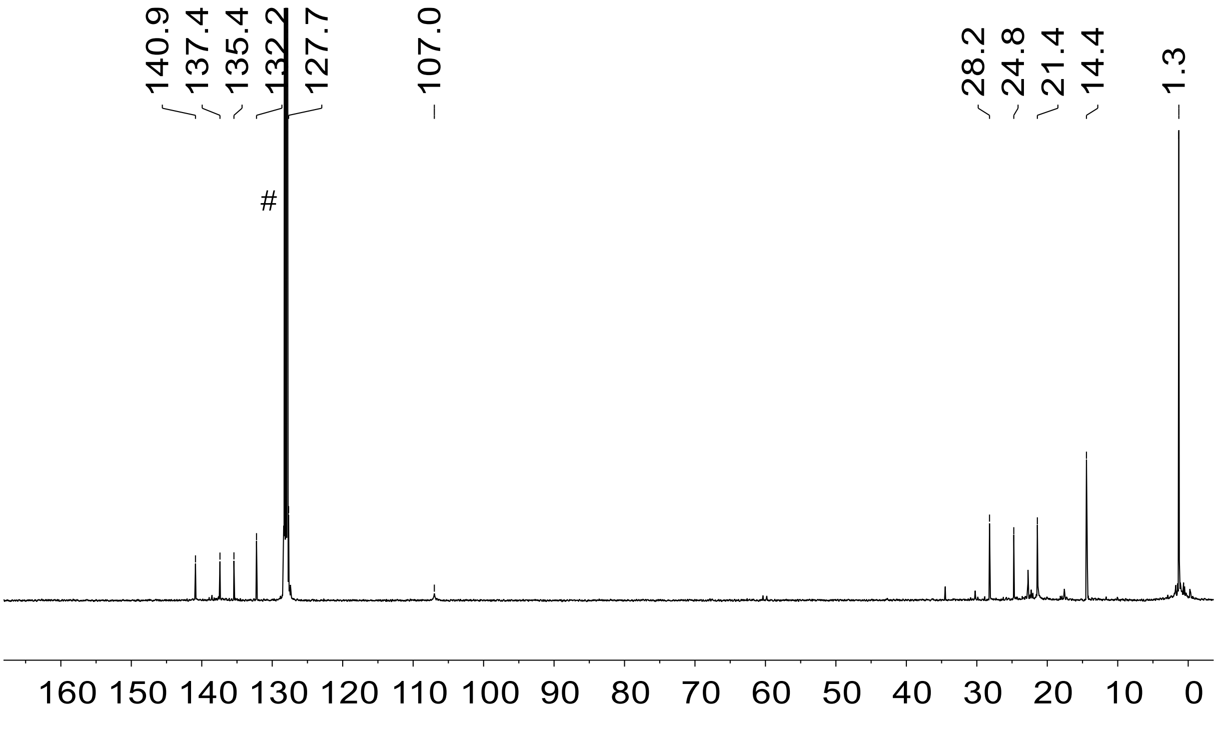

Figure S2. $^{13}C\{^{1}H\}$ NMR spectrum (125.7 MHz, 305.0 K, C_6D_6) of Mes-substituted boragerma[5]pyramidane **12d**, # = C_6D_6.

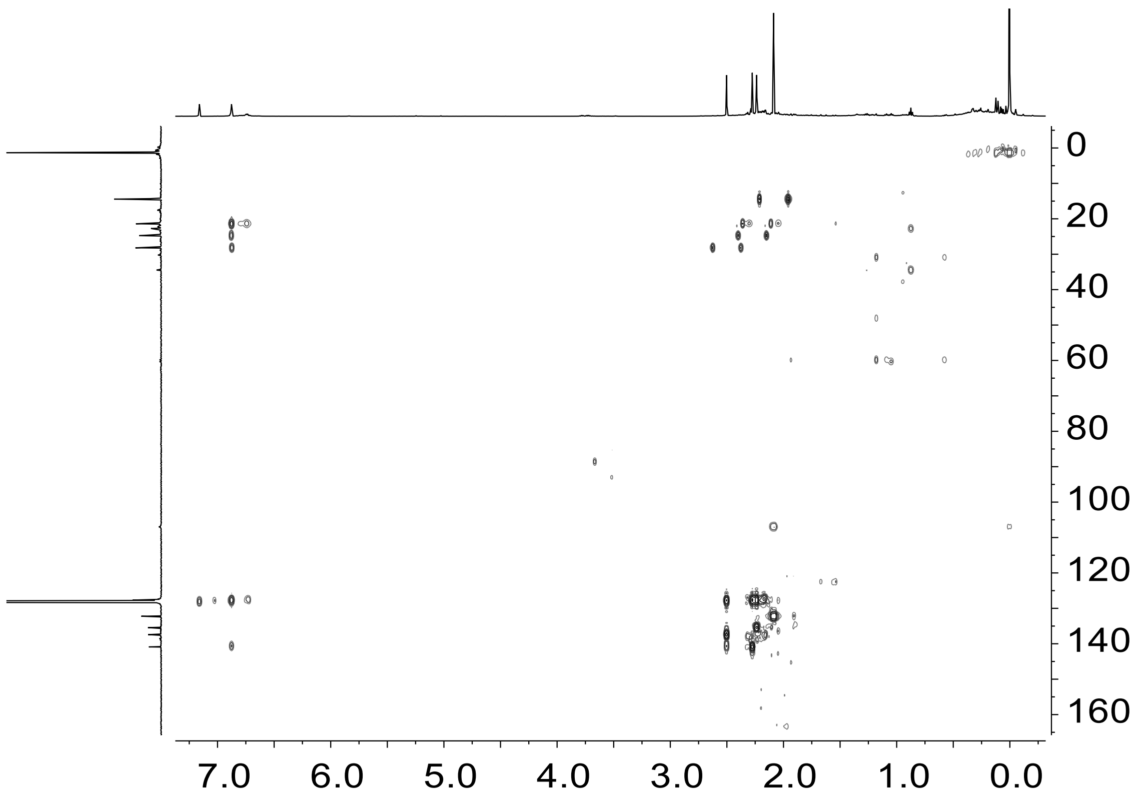

Figure S3. $^{1}H^{13}C$ HMBC NMR spectrum (499.9 MHz, 305.0 K, C_6D_6) of Mes-substituted boragerma[5]pyramidane **12d**.

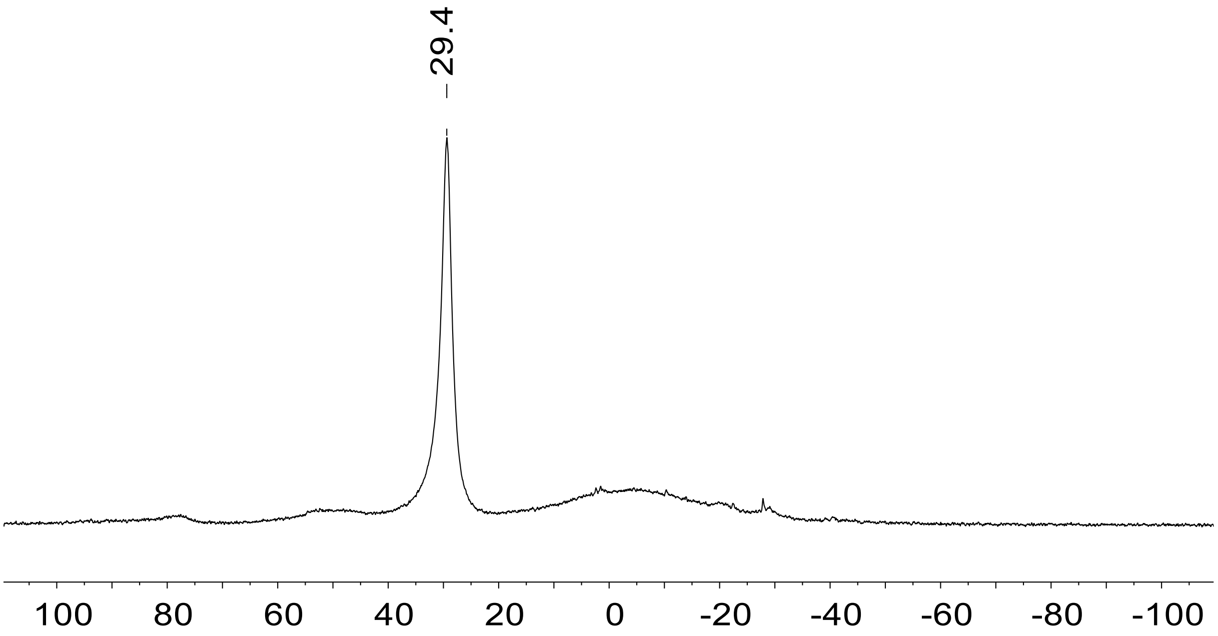

Figure S4. $^{11}B\{^{1}H\}$ NMR spectrum (160.4 MHz, 305.0 K, C_6D_6) of Mes-substituted boragerma[5]pyramidane **12d**.

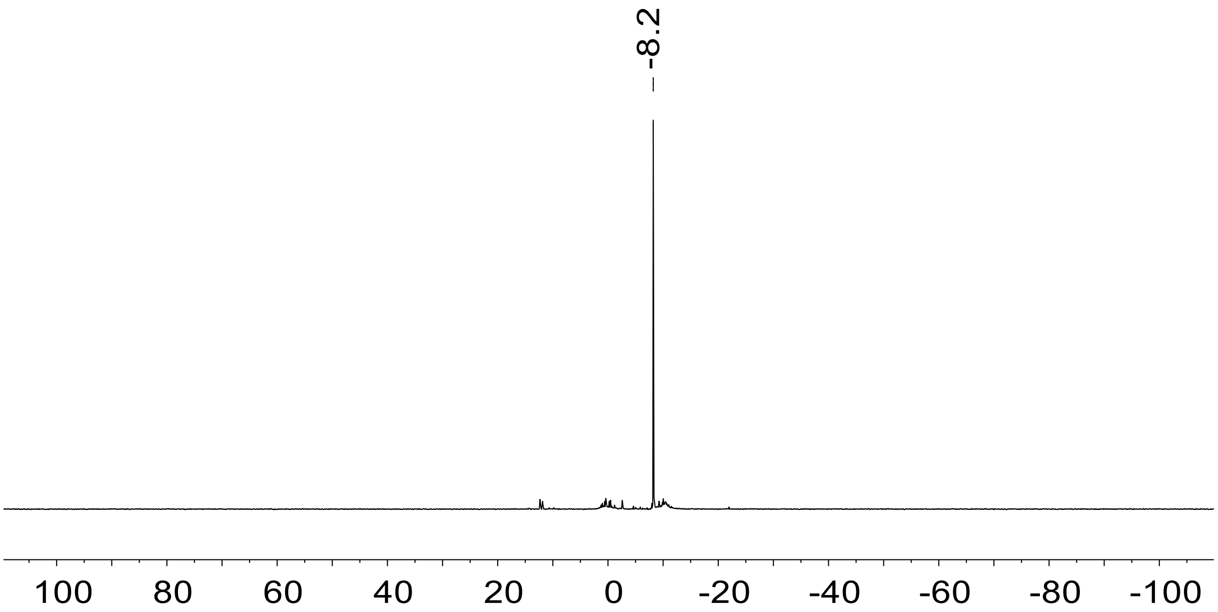

Figure S5. $^{29}Si\{^1H\}$ INEPT NMR spectrum (99.3 MHz, 305.0 K, C_6D_6) of Mes-substituted boragerma[5]pyramidane **12d**.

Synthesis of N^{i}Pr$_2$-substituted boragerma[5]pyramidane **12e**

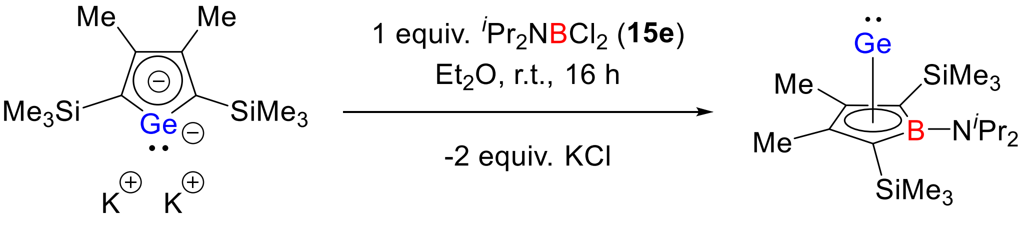

K$_2$[**11**] **12e**

Dipotassium 2,5-bis(trimethylsilyl)-3,4-dimethylgermolediide K$_2$[**11**] (0.50 mmol, 1.00 equiv.) was prepared as described before. The resulting suspension was transferred to a Schlenk flask. A solution of ^{i}Pr$_2$NBCl$_2$ **15e** (91 mg, 0.50 mmol, 1.00 equiv.) in Et$_2$O (30 mL) was added dropwise to the suspension at room temperature. The reaction mixture was stirred for 16 h. The solvent was removed under reduced pressure and the residue was dissolved in *n*-hexane. After filtration, the solvent was removed under reduced pressure. The residue was dissolved in benzene-d$_6$ and analysed by NMR spectroscopy. Complex **12e** could be isolated as yellow crystals from a saturated *n*-pentane solution (yield: 63 mg, 0.15 mmol, 31%).

^{1}H NMR (499.9 MHz, 305.0 K, C$_6$D$_6$): δ = 3.76 (sept, $^3J_{H,H}$ = 6.7 Hz, 2H, N-(C<u>H</u>(CH$_3$)$_2$)$_2$), 2.07 (s, 6H, C$^{2/3}$-C<u>H</u>$_3$), 1.25 (d, $^3J_{H,H}$ = 6.7 Hz, 2H, N-(C<u>H</u>(CH$_3$)$_2$)$_2$), 0.28 (s, 18H, C$^{1/4}$-Si(C<u>H</u>$_3$)$_3$).

^{13}C{^{1}H} NMR (125.7 MHz, 305.0 K, C$_6$D$_6$): δ = 128.3 (C$^{2/3}$), 89.3 (C$^{1/4}$), 49.1 (N-(<u>C</u>H(CH$_3$)$_2$)$_2$), 25.5 (N-(CH(<u>C</u>H$_3$)$_2$)$_2$), 15.0 (C$^{2/3}$-<u>C</u>H$_3$), 1.3 (C$^{1/4}$-Si(<u>C</u>H$_3$)$_3$).

^{11}B{^{1}H} NMR (160.4 MHz, 305.0 K, C$_6$D$_6$): δ = 34.3 (C$_4$<u>B</u>-N^{i}Pr$_2$)

^{29}Si{^{1}H} INEPT NMR (99.3 MHz, 305.0 K, C$_6$D$_6$): δ = -10.1 (C$^{1/4}$-<u>Si</u>(CH$_3$)$_3$).

HR-MS (30 eV, EI): m/z = calc.: 409.1848 $C_{18}H_{38}BGeNSi_2$
exp.: 409.1841

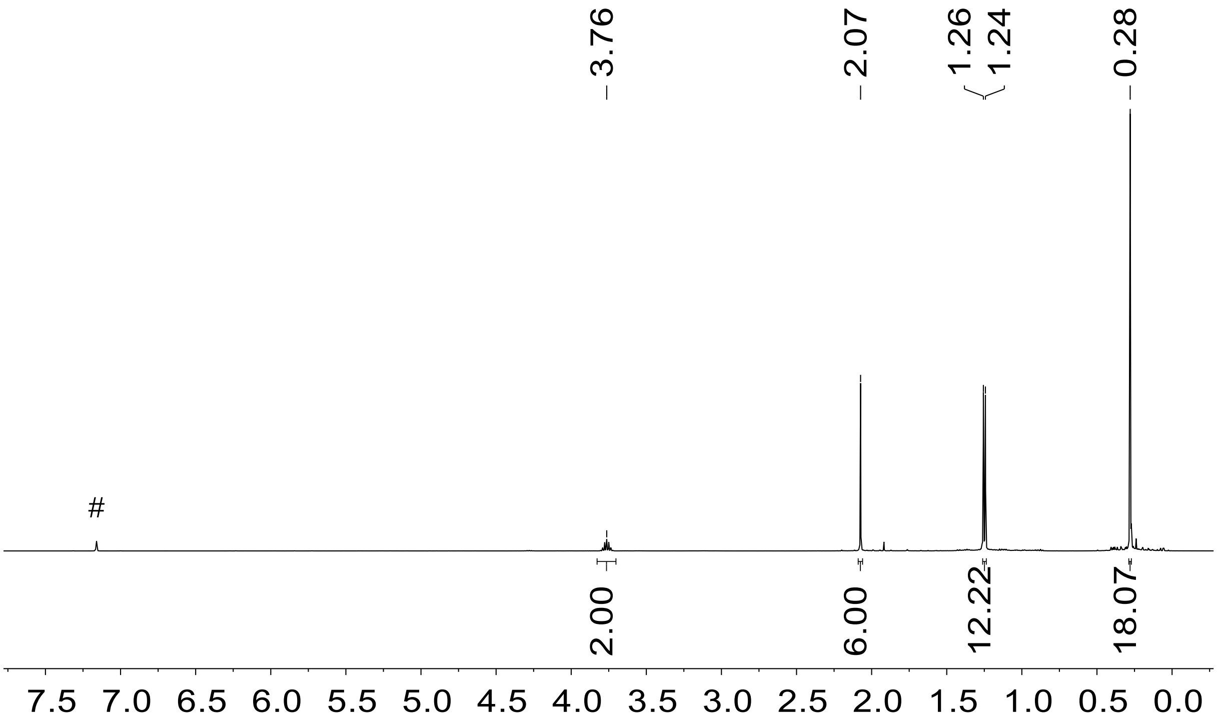

Figure S6. ^{1}H NMR spectrum (499.9 MHz, 305.0 K, C_6D_6) of N^{i}Pr$_2$-substituted boragerma[5]pyramidane **12e**, # = C_6D_5H.

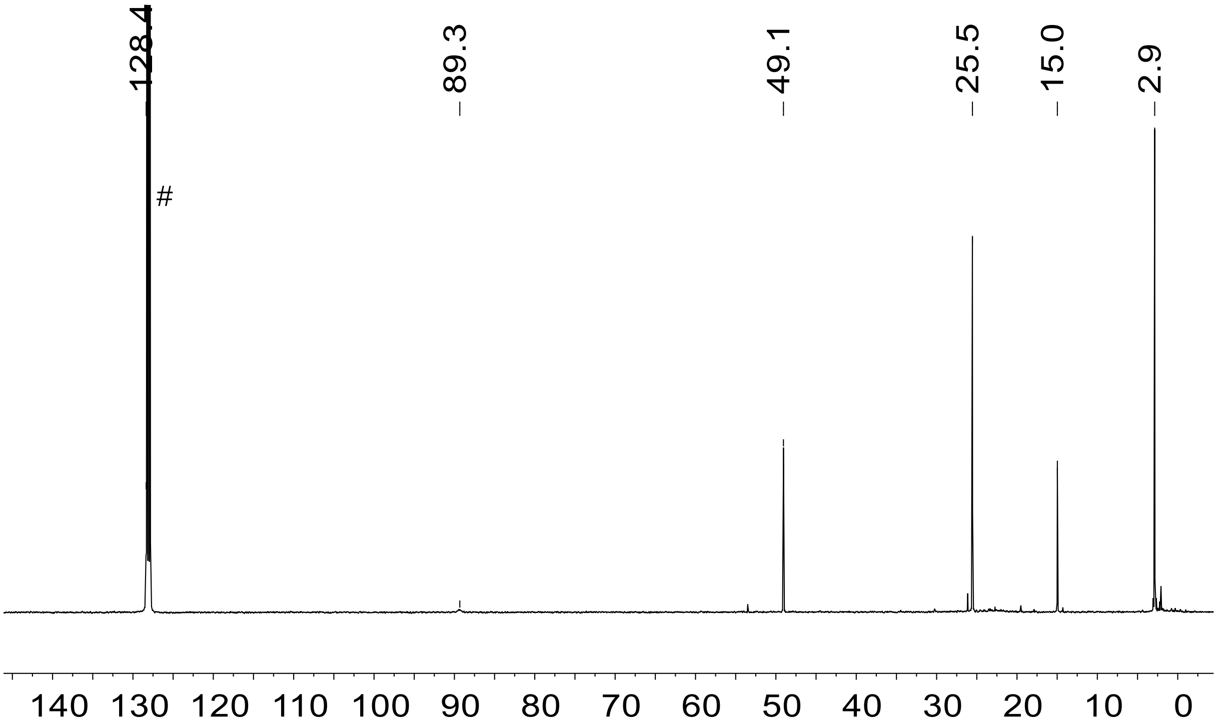

Figure S7. $^{13}C\{^{1}H\}$ NMR spectrum (125.7 MHz, 305.0 K, C_6D_6) of N^{i}Pr$_2$-substituted boragerma[5]pyramidane **12e**, # = C_6D_6.

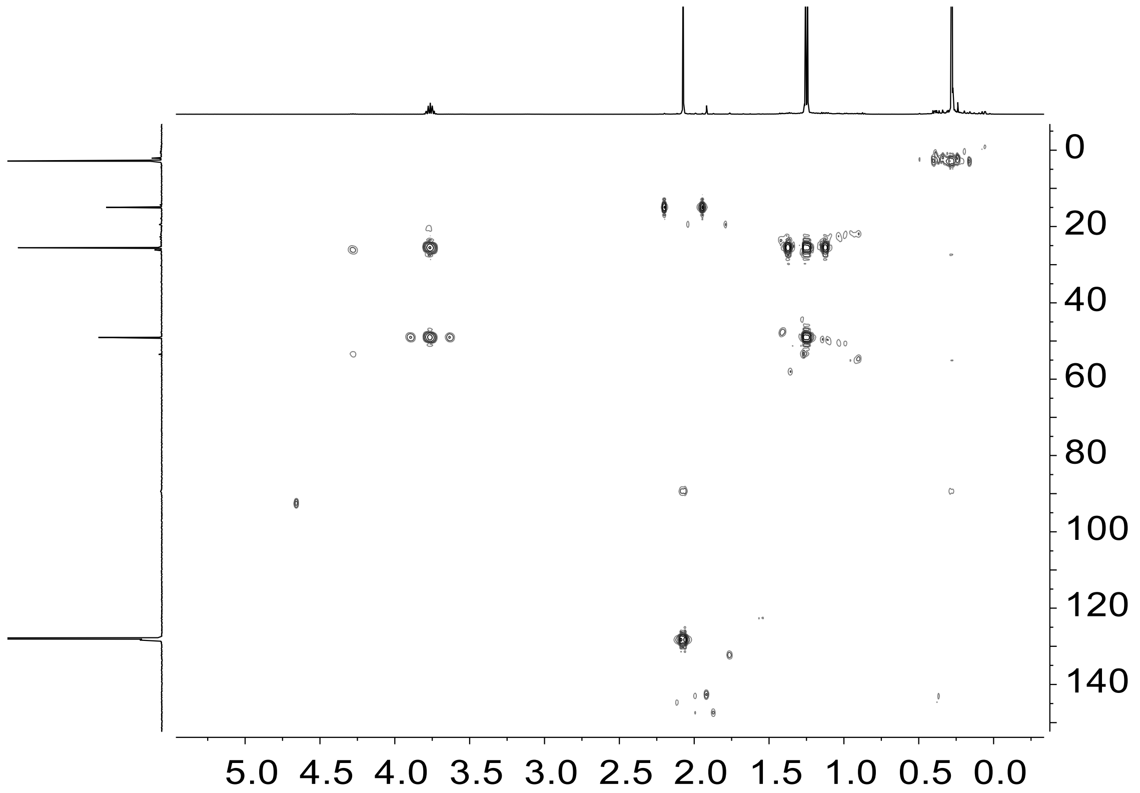

Figure S8. $^{1}H^{13}C$ HMBC NMR spectrum (499.9 MHz, 305.0 K, C_6D_6) of N^{i}Pr$_2$-substituted boragerma[5]pyramidane **12e**.

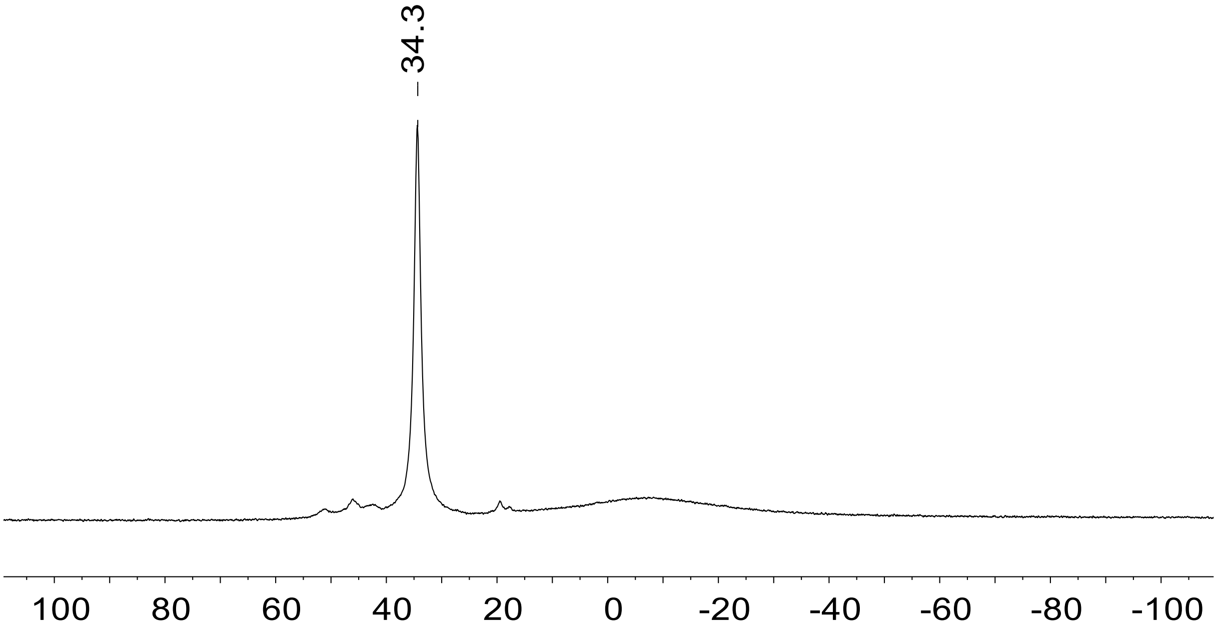

Figure S9. $^{11}B\{^{1}H\}$ NMR spectrum (160.4 MHz, 305.0 K, C_6D_6) of N^{i}Pr$_2$-substituted boragerma[5]pyramidane **12e**.

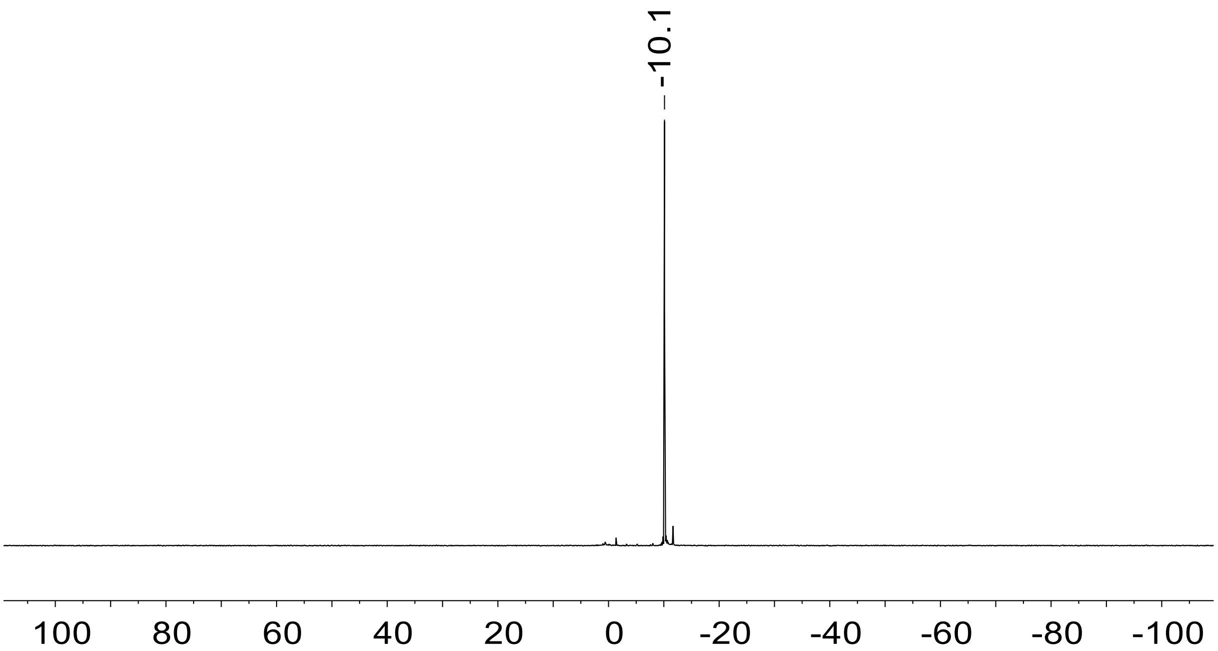

Figure S10. $^{29}Si\{^{1}H\}$ INEPT NMR spectrum (99.3 MHz, 305.0 K, C_6D_6) of N^{i}Pr$_2$-substituted boragerma[5]pyramidane **12e**.

Synthesis of NCy_2-substituted boragerma[5]pyramidane 12f

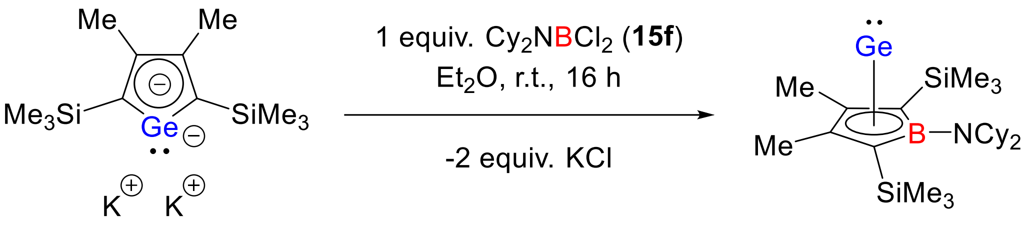

K_2[**11**] **12f**

Dipotassium 2,5-bis(trimethylsilyl)-3,4-dimethylgermolediide K_2[**11**] (0.50 mmol, 1.00 equiv.) was prepared as described before. The resulting suspension was transferred to a Schlenk flask. A solution of Cy_2NBCl_2 **15f** (131 mg, 0.50 mmol, 1.00 equiv.) in Et_2O (30 mL) was added dropwise to the suspension at room temperature. The reaction mixture was stirred for 16 h. The solvent was removed under reduced pressure and the residue was dissolved in *n*-hexane. After filtration, the solvent was removed under reduced pressure. The residue was dissolved in benzene-d_6 and analysed by NMR spectroscopy. Complex **12f** could be isolated as a dark-yellow oil (raw yield: 184 mg, 0.38 mmol, 75%). Several attempts to purify compound **12f** by recrystallisation did not succeed.

^{1}H NMR (499.9 MHz, 305.0 K, C_6D_6): δ = 3.12-3.07 (m, 2H, N(C<u>H</u>-C_5H_{10})$_2$), 2.20-2.18 (m, 4H, N(CH-C_5<u>H</u>$_{10}$)$_2$), 2.07 (s, 6H, $C^{2/3}$-C<u>H</u>$_3$), 1.85-0.98 (m, 16H, N(CH-C_5<u>H</u>$_{10}$)$_2$), 0.31 (s, 18H, $C^{1/4}$-Si(C<u>H</u>$_3$)$_3$).

^{13}C{^{1}H} NMR (125.7 MHz, 305.0 K, C_6D_6): δ = 128.6 ($C^{2/3}$), 100.4 ($C^{1/4}$), 57.3 (N(<u>C</u>H-C_5H_{10})$_2$), 36.3 (N(CH-<u>C</u>$_5H_{10}$)$_2$), 27.1 (N(CH-<u>C</u>$_5H_{10}$)$_2$), 26.9 (N(CH-<u>C</u>$_5H_{10}$)$_2$), 14.8 ($C^{2/3}$-<u>C</u>H$_3$), 2.8 ($C^{1/4}$-Si(<u>C</u>H$_3$)$_3$).

^{11}B{^{1}H} NMR (160.4 MHz, 305.0 K, C_6D_6): δ = 32.1 (C_4<u>B</u>-NCy_2).

^{29}Si{^{1}H} INEPT NMR (99.3 MHz, 305.0 K, C_6D_6): δ = -10.3 ($C^{1/4}$-<u>Si</u>(CH$_3$)$_3$).

HR-MS (30 eV, EI): m/z = calc.: 489.2474 $C_{24}H_{46}BGeNSi_2$
exp.: 489.2464

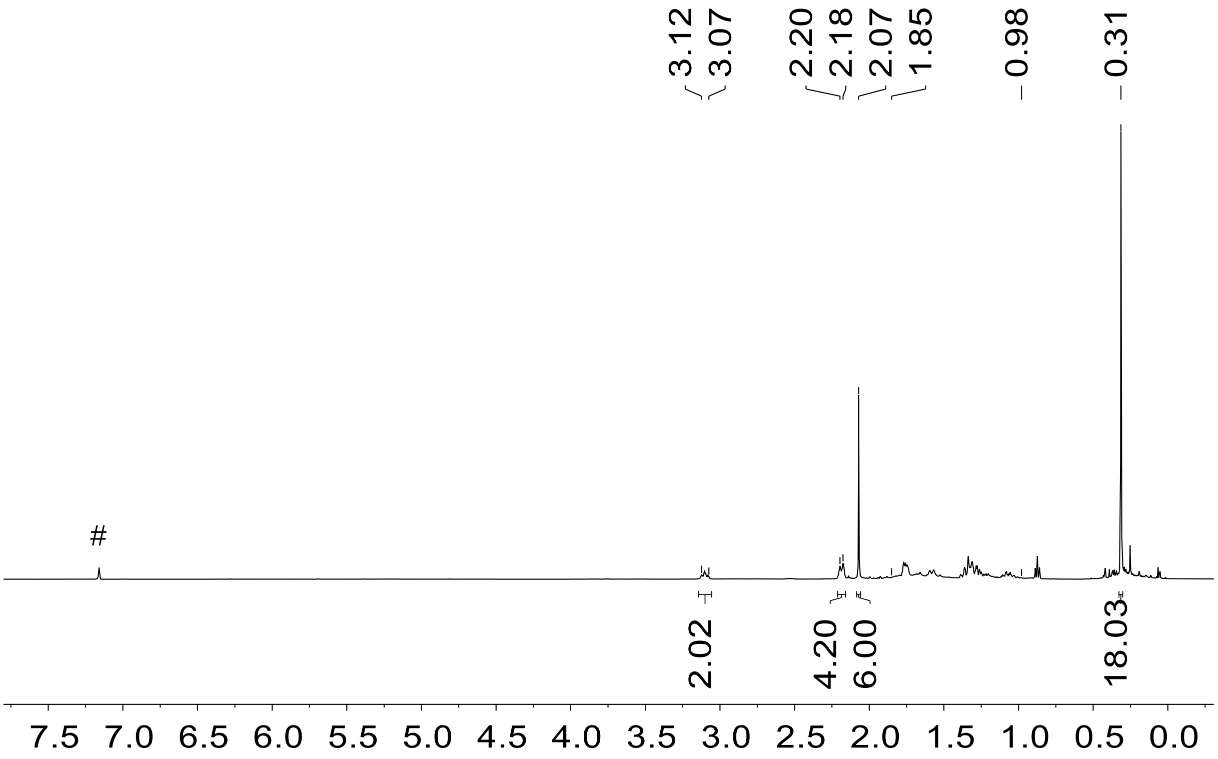

Figure S11. ^{1}H NMR spectrum (499.9 MHz, 305.0 K, C_6D_6) of NCy_2-substituted boragerma[5]pyramidane **12f**, # = C_6D_5H.

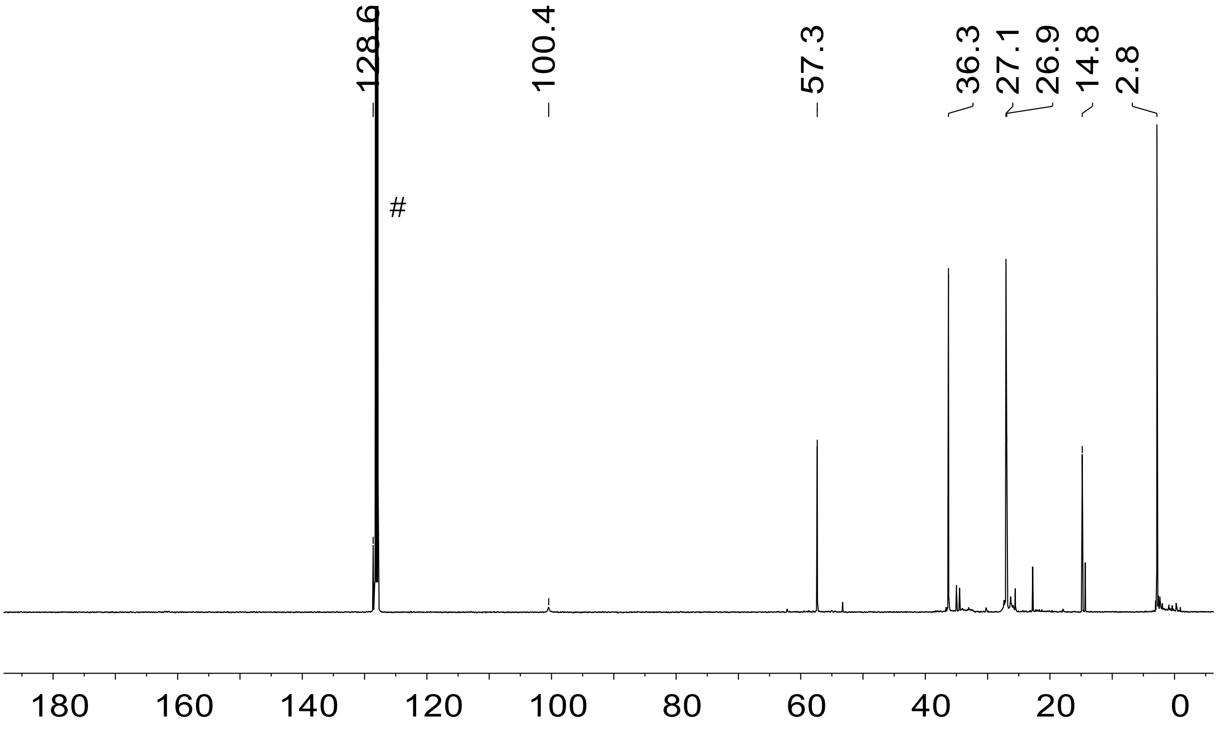

Figure S12. $^{13}C\{^{1}H\}$ NMR spectrum (125.7 MHz, 305.0 K, C_6D_6) of NCy_2-substituted boragerma[5]pyramidane **12f**, # = C_6D_6.

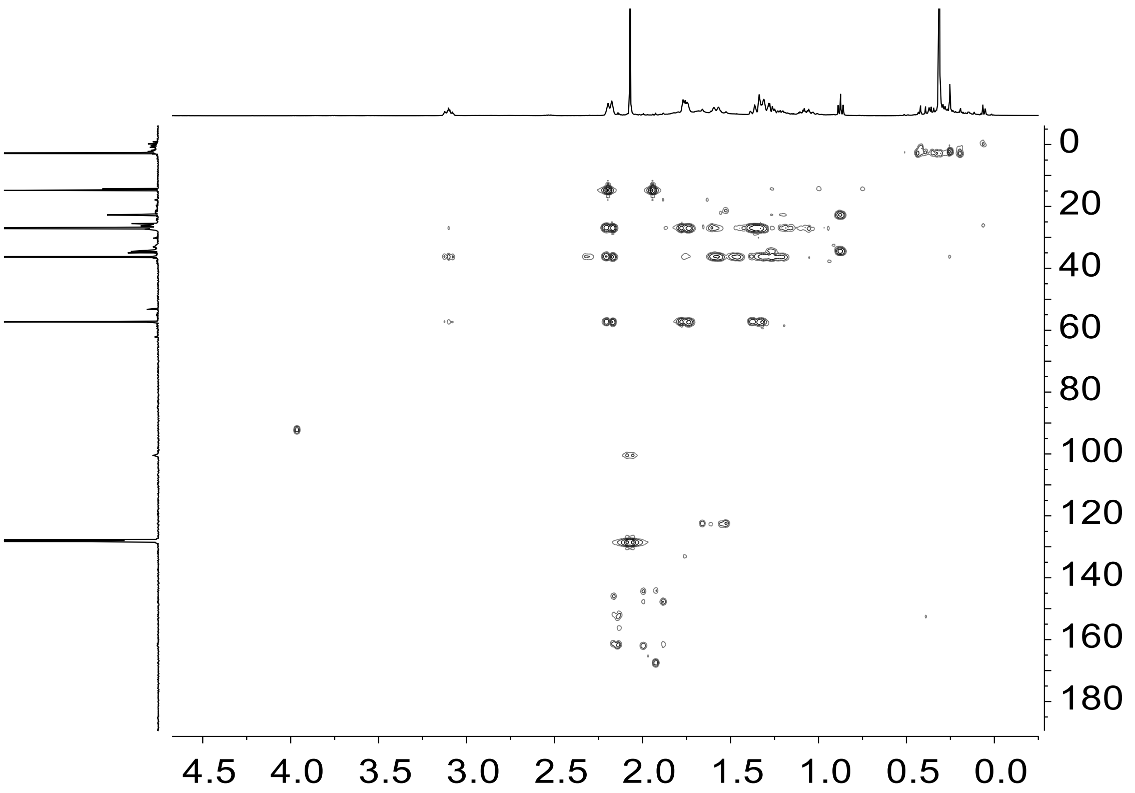

Figure S13. $^{1}H^{13}C$ HMBC NMR spectrum (499.9 MHz, 305.0 K, C_6D_6) of NCy_2-substituted boragerma[5]pyramidane **12f**.

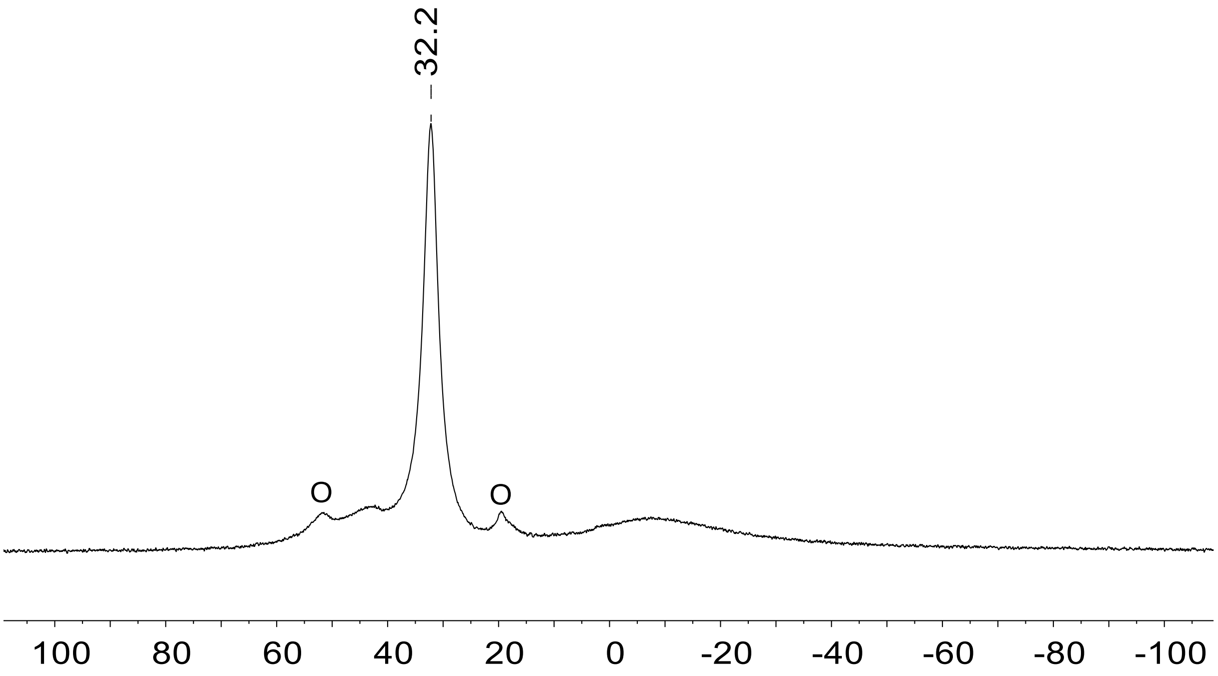

Figure S14. $^{11}B\{^{1}H\}$ NMR spectrum (160.4 MHz, 305.0 K, C_6D_6) of NCy_2-substituted boragerma[5]pyramidane **12f**, O = unidentified impurities.

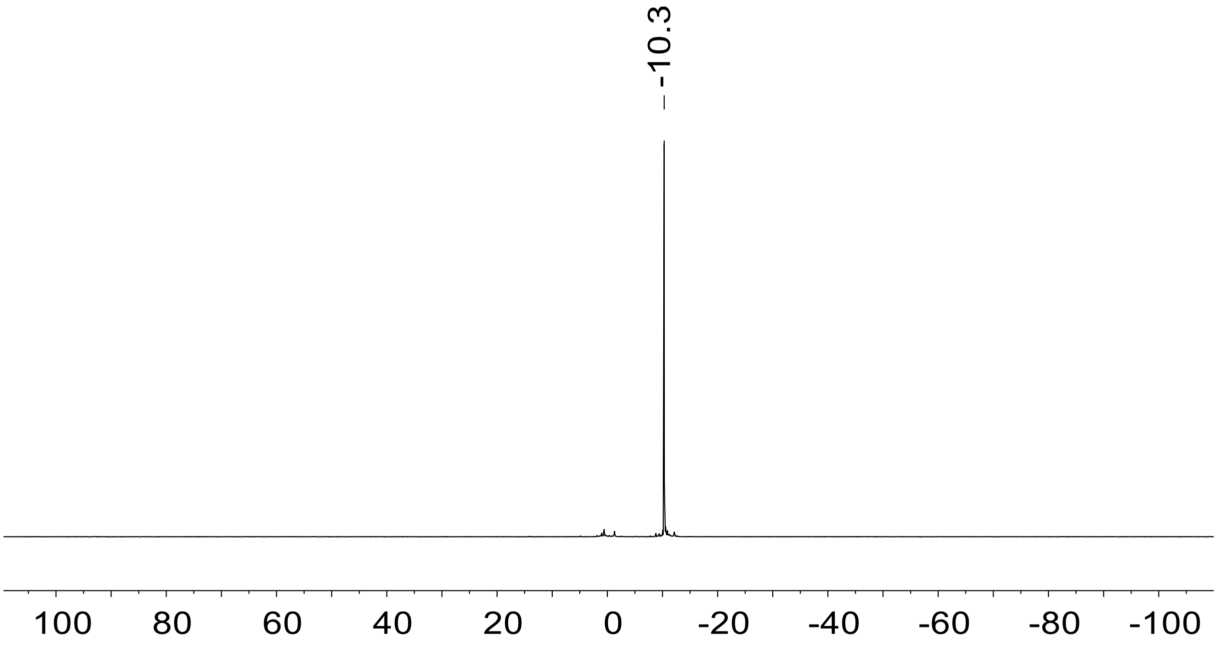

Figure S15. $^{29}Si\{^1H\}$ INEPT NMR spectrum (99.3 MHz, 305.0 K, C_6D_6) of NCy_2-substituted boragerma[5]pyramidane **12f**.

Synthesis of ferrocenyl-substituted boragerma[5]pyramidane 12g

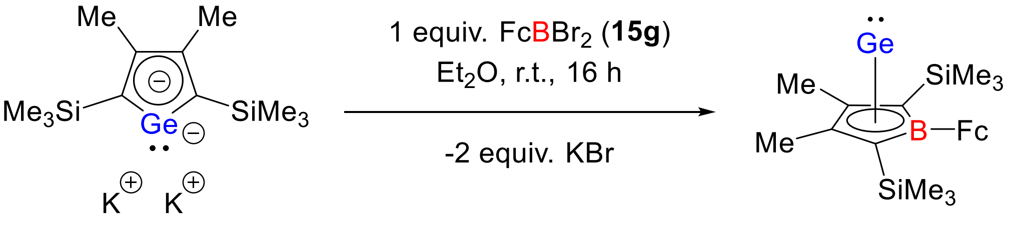

K$_2$[**11**] **12g**

Dipotassium 2,5-bis(trimethylsilyl)-3,4-dimethylgermolediide K$_2$[**11**] (0.50 mmol, 1.00 equiv.) was prepared as described before. The resulting suspension was transferred to a Schlenk flask. A solution of $FcBBr_2$ **15g** (178 mg, 0.50 mmol, 1.00 equiv.) in Et_2O (30 mL) was added dropwise to the suspension at room temperature. The reaction mixture was stirred for 16 h. The solvent was removed under reduced pressure and the residue was dissolved in *n*-hexane. After filtration, the solvent was removed under reduced pressure. Compound **12g** could be isolated as red crystals from a saturated *n*-pentane solution (yield: 87 mg, 0.18 mmol, 35%).

^{1}H NMR (499.9 MHz, 305.0 K, C_6D_6): δ = 4.44-4.43 (m, 2H, C_4B-$C_5\underline{H}_4$), 4.27-4.26 (m, 2H, C_4B-$C_5\underline{H}_4$), 4.09 (s, 5H, Fe-$C_5\underline{H}_5$), 2.03 (s, 6H, $C^{2/3}$-$C\underline{H}_3$), 0.34 (s, 18H, $C^{1/4}$-Si($C\underline{H}_3$)$_3$).

^{13}C{^{1}H} NMR (125.7 MHz, 305.0 K, C_6D_6): δ = 133.5 ($C^{1/4}$), 101.7 ($C^{1/4}$), 76.8 (C_4B-$\underline{C}_5H_4$), 69.4 (C_4B-$\underline{C}_5H_4$), 69.2 (Fe-$\underline{C}_5H_5$), 14.8 ($C^{2/3}$-$\underline{C}H_3$), 3.4 ($C^{1/4}$-Si($\underline{C}H_3$)$_3$).
The ^{13}C NMR signal of the *α*-carbon atom bonded to the boron atom could not be determined.

^{11}B{^{1}H} NMR (160.4 MHz, 305.0 K, C_6D_6): δ = 32.5 ($C_4\underline{B}$-Fc).

^{29}Si{^{1}H} INEPT NMR (99.3 MHz, 305.0 K, C_6D_6): δ = -9.1 ($C^{1/4}$-$\underline{Si}$(CH_3)$_3$).

HR-MS (30 eV, EI): m/z = calc.: 494.0775 $C_{22}H_{33}BFeGeSi_2$
exp.: 494.0765

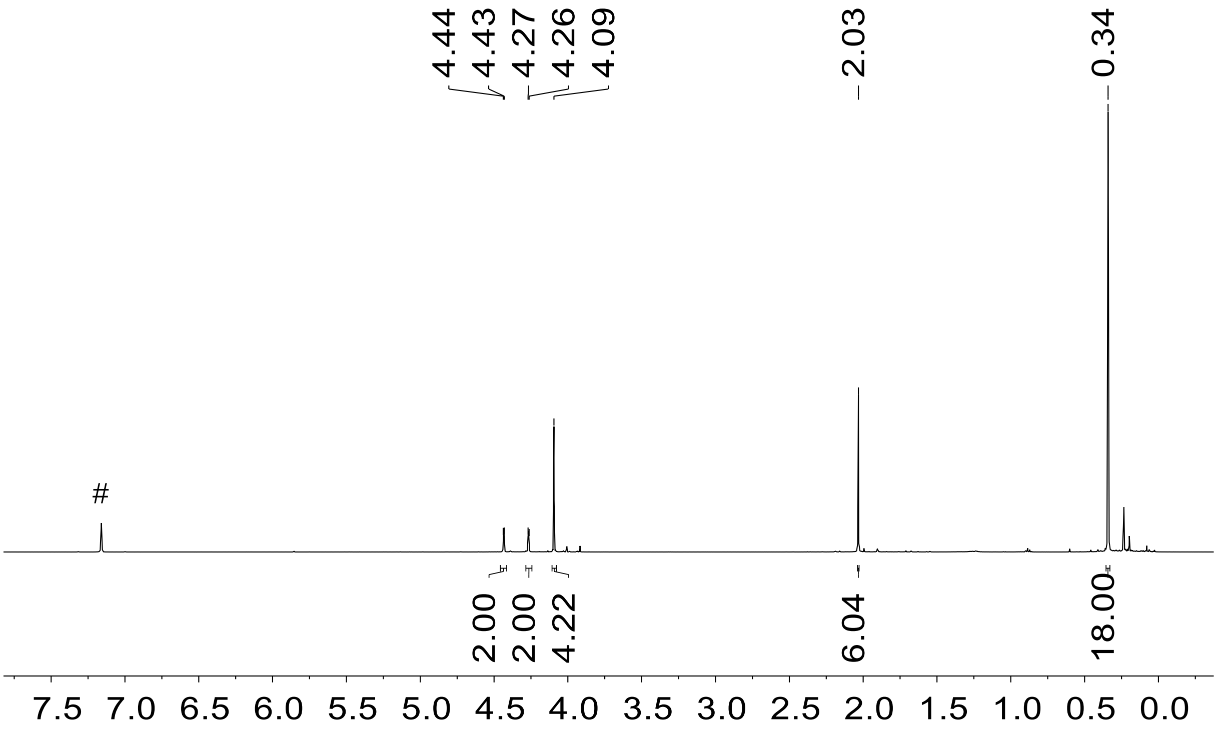

Figure S16. ^{1}H NMR spectrum (499.9 MHz, 305.0 K, C_6D_6) of Fc-substituted boragerma[5]pyramidane **12g**, # = C_6D_5H.

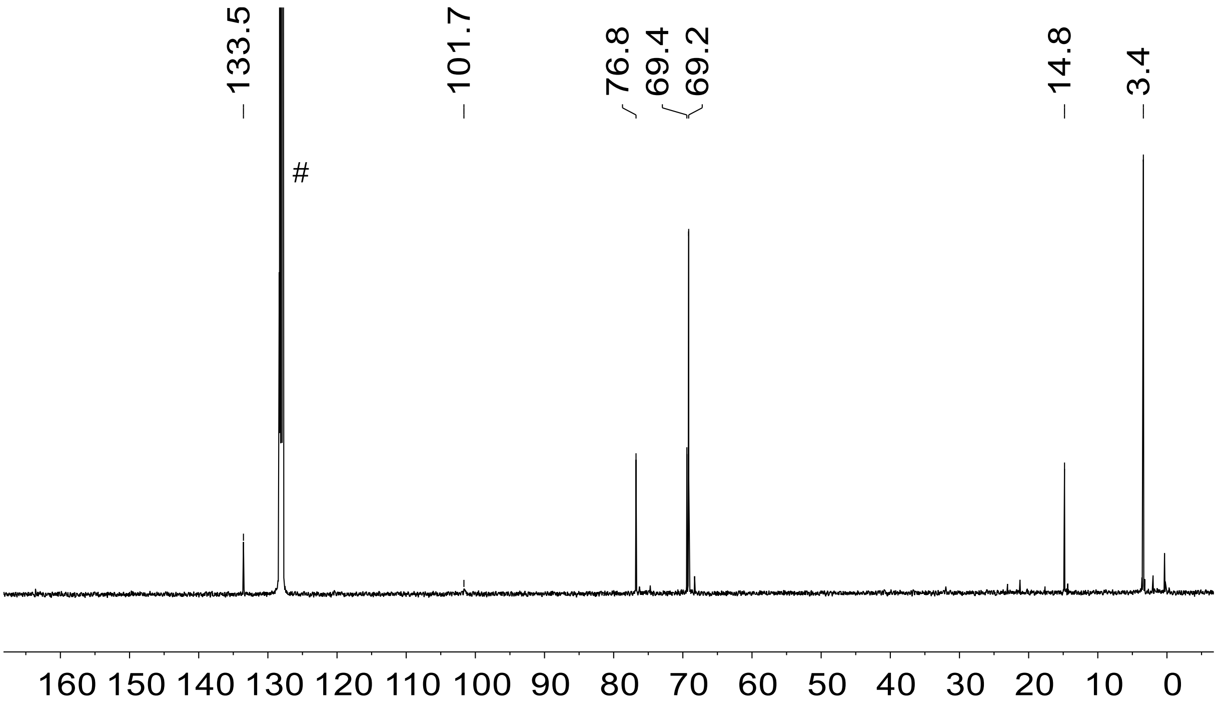

Figure S17. $^{13}C\{^{1}H\}$ NMR spectrum (125.7 MHz, 305.0 K, C_6D_6) of Fc-substituted boragerma[5]pyramidane **12g**, # = C_6D_6.

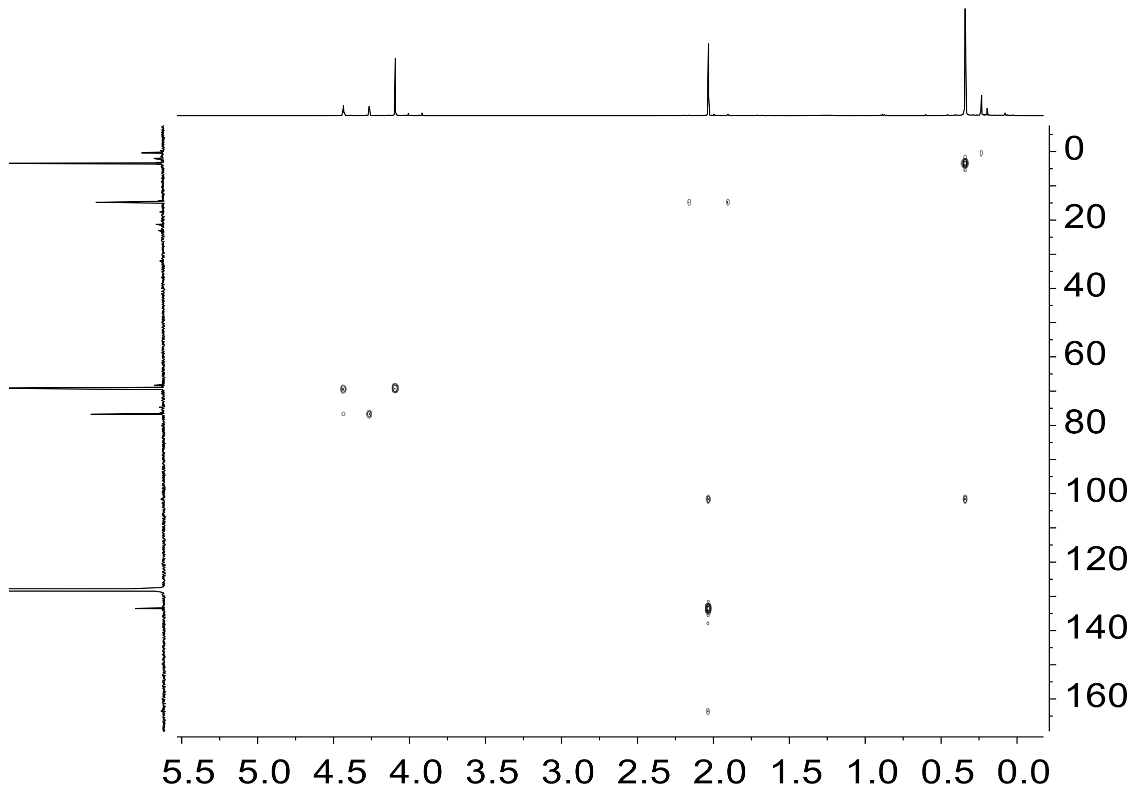

Figure S18. $^{1}H^{13}C$ HMBC NMR spectrum (499.9 MHz, 305.0 K, C_6D_6) of Fc-substituted boragerma[5]pyramidane **12g**.

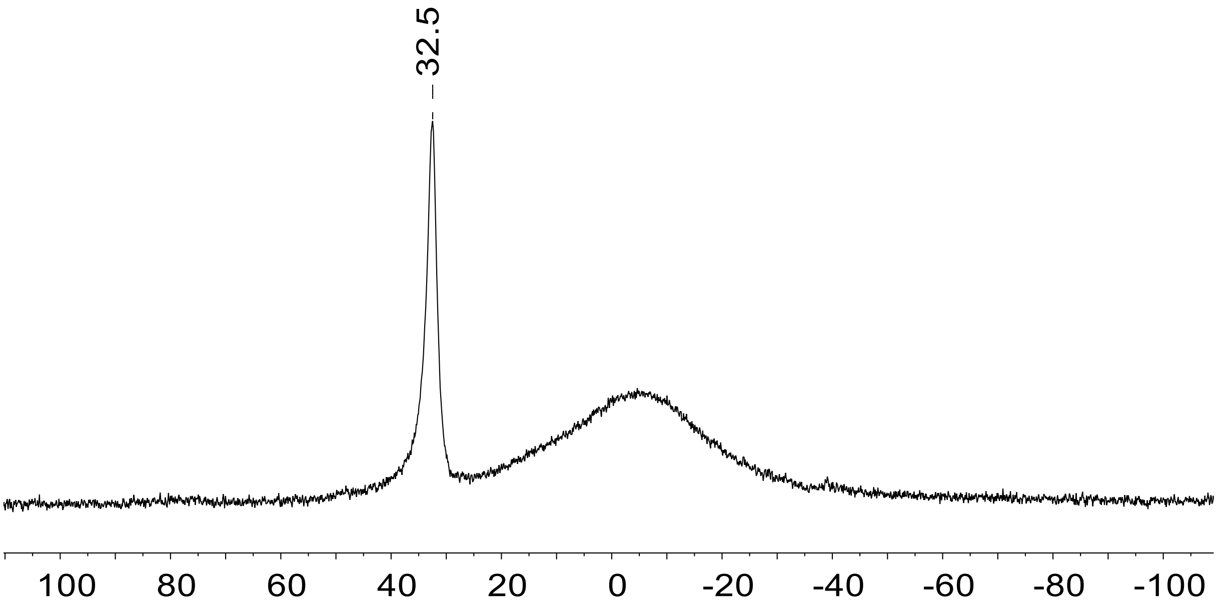

Figure S19. $^{11}B\{^{1}H\}$ NMR spectrum (160.4 MHz, 305.0 K, C_6D_6) of Fc-substituted boragerma[5]pyramidane **12g**.

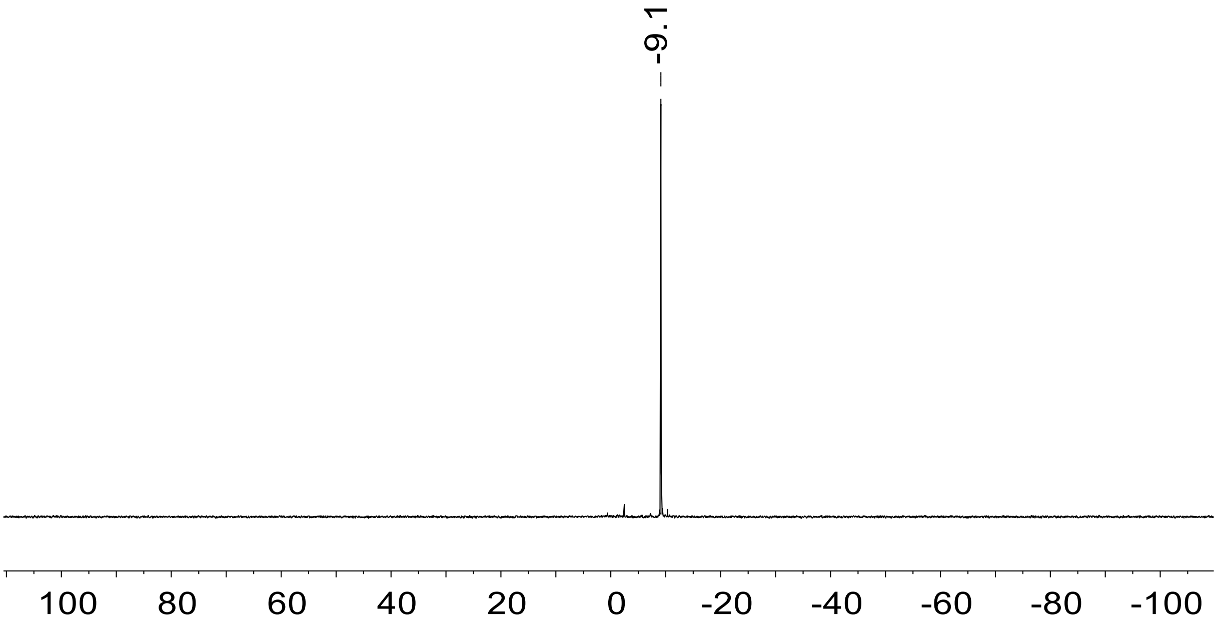

Figure S20. $^{29}Si\{^1H\}$ INEPT NMR spectrum (99.3 MHz, 305.0 K, C_6D_6) of Fc-substituted boragerma[5]pyramidane **12g**.

Synthesis of Cp*-substituted boragerma[5]pyramidane 14a

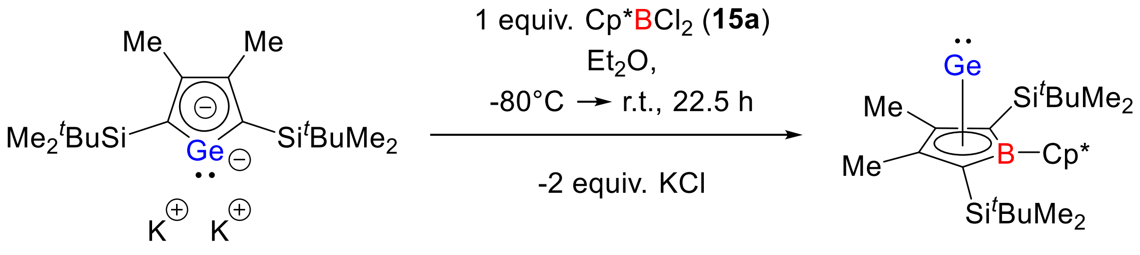

K$_2$[**13**] **14a**

Dipotassium 2,5-bis(*tert*-butyldimethylsilyl)-3,4-dimethylgermolediide K$_2$[**13**] (0.50 mmol, 1.00 equiv.) was prepared as described before. The resulting suspension was transferred to a Schlenk flask. A solution of Cp*BCl$_2$ **15a** (108 mg, 0.50 mmol, 1.00 equiv.) in Et$_2$O (5 mL) was added to the suspension at –80 °C. The reaction mixture was stirred at this temperature for 1.5 h. The cooling bath was removed and the mixture was stirred for additional 21 h. The solvent was removed under reduced pressure and the residue was dissolved in *n*-hexane and filtered over celite. Then, the solvent was removed under reduced pressure. The residue was dissolved in benzene-d$_6$ and analysed by NMR spectroscopy. Complex **14a** was isolated as a brown oil (raw yield: 143 mg, 0.37 mmol, 73%). A small batch of crystals of **14a** which were suitable for sc-XRD was obtained from benzene at r.t..

^{1}H NMR (499.9 MHz, 305.0 K, C$_6$D$_6$): δ = 2.14 (s, 3H, C$^{2/3}$-C<u>H</u>$_3$), 2.09 (s, 3H, C$^{2/3}$-C<u>H</u>$_3$), 2.02 1.89, 1.84, 1.82, 1.49 (5x s, 5x 3H, Cp*Me),f 1.14 (s, 9H, Si(C(C<u>H</u>$_3$)$_3$)(CH$_3$)$_2$), 0.98 (s, 9H, Si(C(C<u>H</u>$_3$)$_3$)(CH$_3$)$_2$), 0.51, 0.16, 0.03, 0.00 (4x s, 4x 3H, C$^{1/4}$-Si(C(CH$_3$)$_3$)(C<u>H</u>$_3$)$_2$).

^{13}C{^{1}H} NMR (125.7 MHz, 305.0 K, C$_6$D$_6$): δ = 149.0 (Cp*), 145.4 (Cp*), 136.0 (C$^{2/3}$), 135.4 (C$^{2/3}$), 133.7 (Cp*), 131.5 (Cp*), 101.4 (C$^{1/4}$), 100.7 (C$^{1/4}$), 53.5 (Cp*), 30.2 (^{t}Bu<u>Me</u>), 29.4 (^{t}Bu<u>Me</u>), 28.5 (Cp*-Me), 18.9, 18.9 (2x ^{t}Bu<u>C^{q}</u>), 17.8 (C$^{2/3}$-<u>C</u>H$_3$), 17.5 (C$^{2/3}$-<u>C</u>H$_3$), 14.0 (Cp*-Me), 13.5 (Cp*-Me), 12.1 (Cp*-Me), 11.9 (Cp*-Me), 6.2 (Si(<u>C</u>H$_3$)$_2$), 4.6 (Si(<u>C</u>H$_3$)$_2$), 3.0 (Si(<u>C</u>H$_3$)$_2$), 0.2 (Si(<u>C</u>H$_3$)$_2$).

^{11}B{^{1}H} NMR (160.4 MHz, 305.0 K, C$_6$D$_6$): δ = 35.6 (C$_4$<u>B</u>-C$_6$H$_2$(CH$_3$)$_3$).

^{29}Si{^{1}H} INEPT NMR (99.3 MHz, 305.0 K, C$_6$D$_6$): δ = 0.13, -0.45 (C$^{1/4}$-<u>Si</u>(C(CH$_3$)$_3$)(CH$_3$)$_2$).

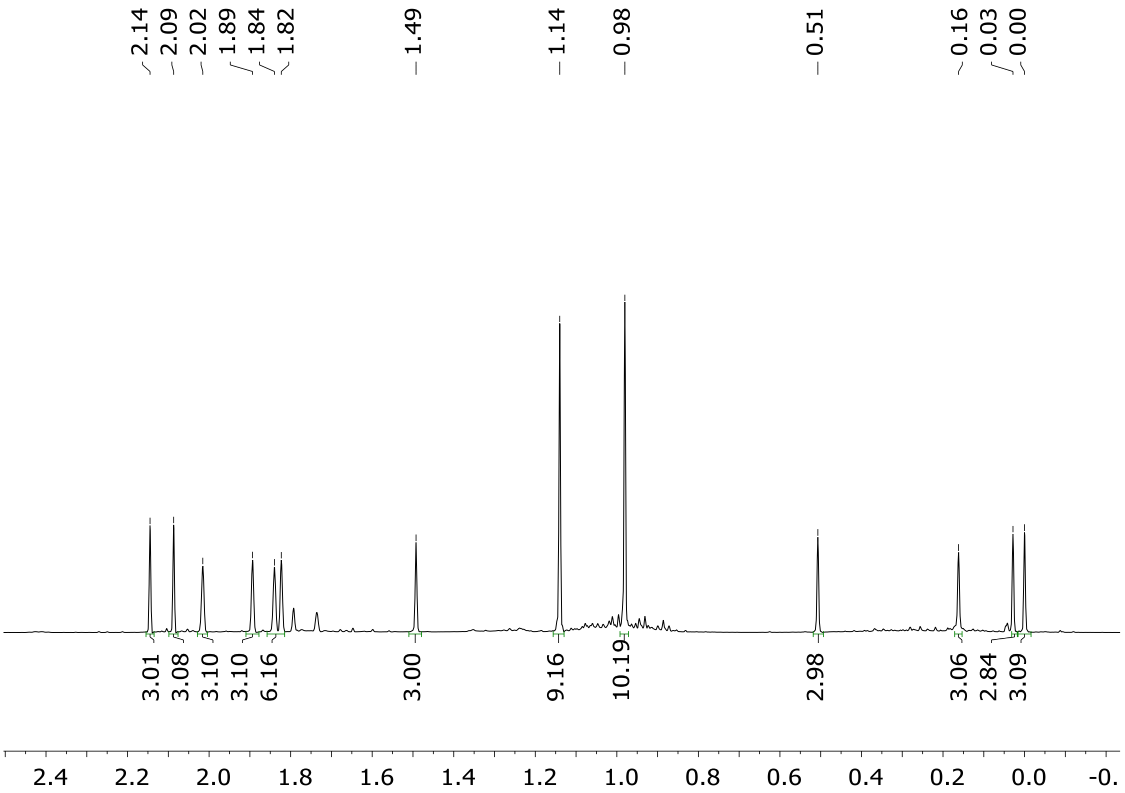

Figure S21. ^{1}H NMR spectrum (499.9 MHz, 305.0 K, C_6D_6) of Cp*-substituted boragerma[5]pyramidane **14a**.

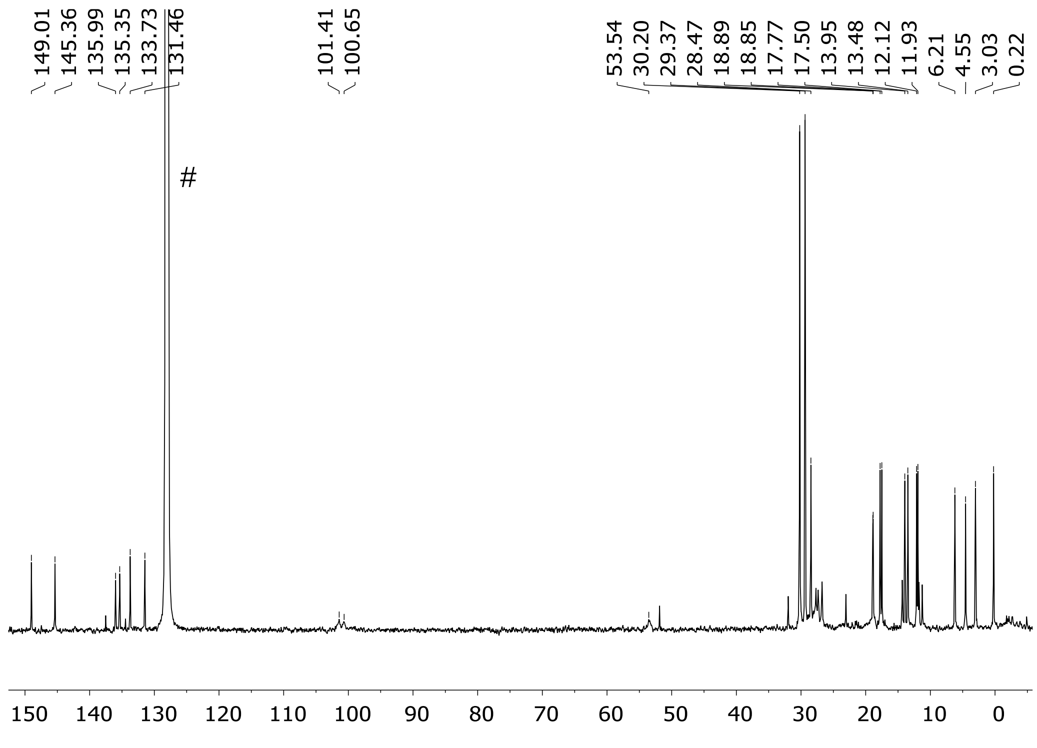

Figure S21. $^{13}C\{^{1}H\}$ NMR spectrum (125.7 MHz, 305.0 K, C_6D_6) of Cp*-substituted boragerma[5]pyramidane **14a**, # = C_6D_6.

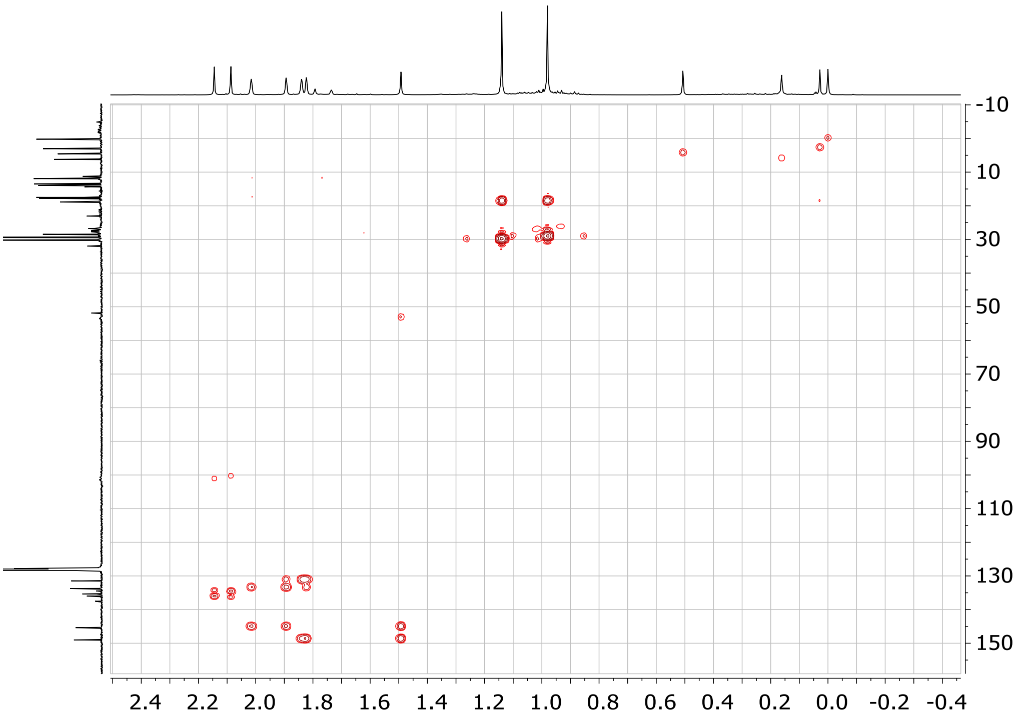

Figure S23. ^{1}H^{13}C HMBC NMR spectrum (499.9 MHz, 305.0 K, C_6D_6) of Cp*-substituted boragerma[5]pyramidane **14a**.

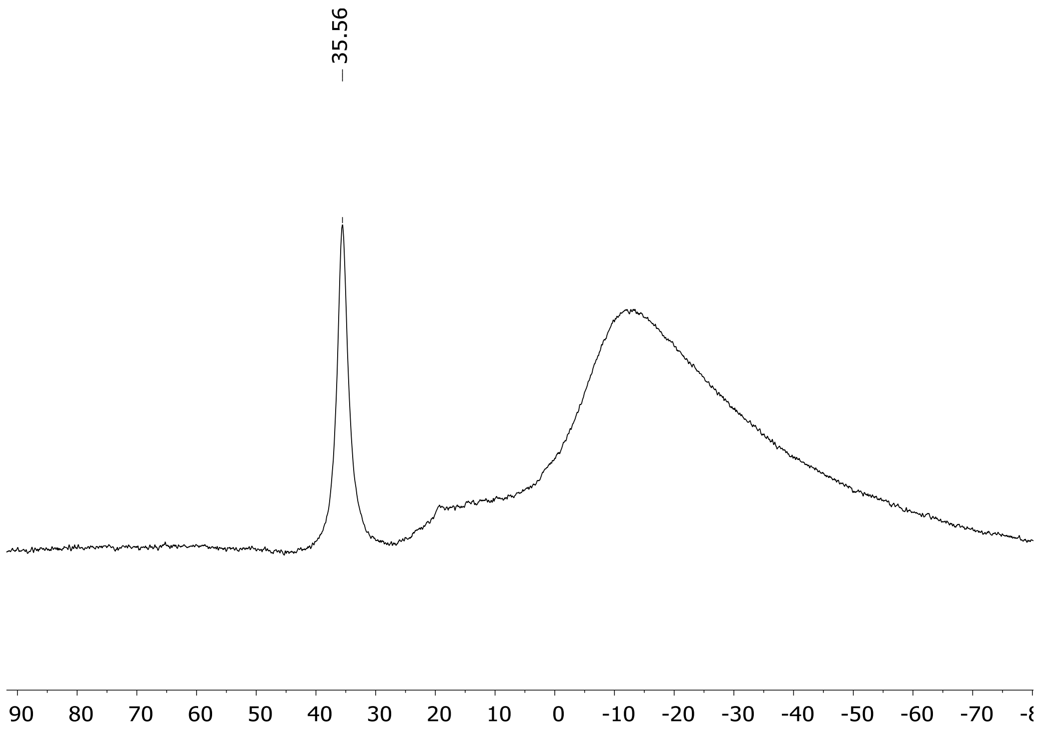

Figure S22. $^{11}B\{^1H\}$ NMR spectrum (160.4 MHz, 305.0 K, C_6D_6) of Cp*-substituted boragerma[5]pyramidane **14a**.

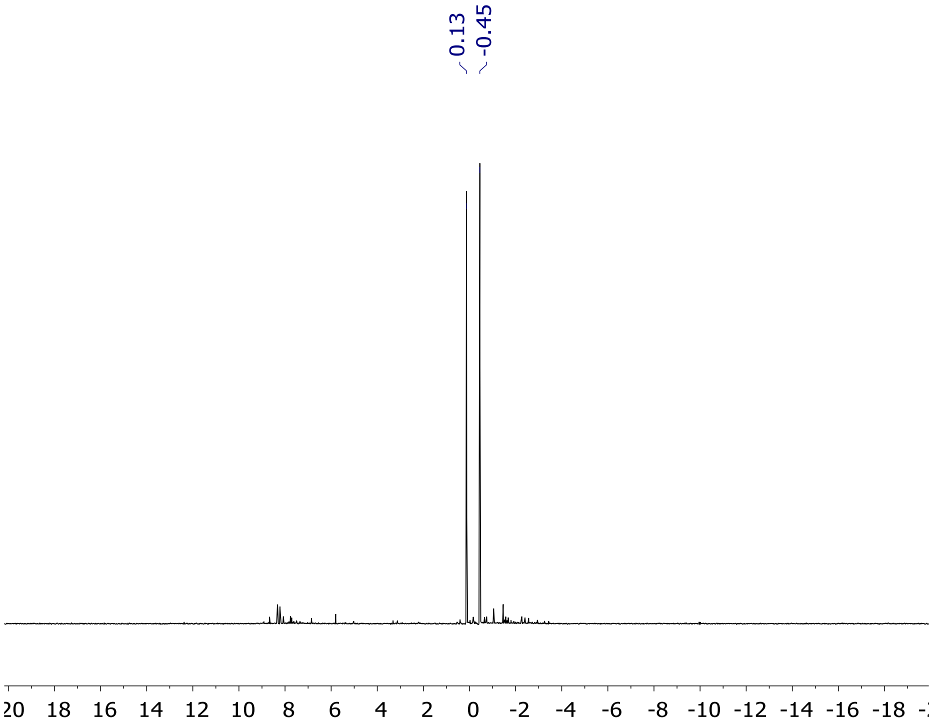

Figure S23. $^{29}Si\{^1H\}$ INEPT NMR spectrum (99.3 MHz, 305.0 K, C_6D_6) of Cp*-substituted boragerma[5]pyramidane **14a**.

Synthesis of Mes-substituted boragerma[5]pyramidane 14d

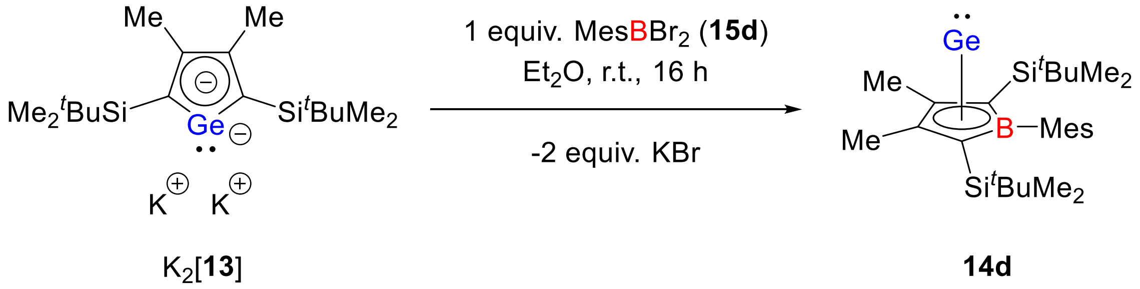

Dipotassium 2,5-bis(*tert*-butyldimethylsilyl)-3,4-dimethylgermolediide K_2[**13**] (0.22 mmol, 1.00 equiv.) was prepared as described before. The resulting suspension was transferred to a Schlenk flask. A solution of $MesBBr_2$ **15d** (64 mg, 0.22 mmol, 1.00 equiv.) in Et_2O (30 mL) was added dropwise to the suspension at room temperature. The reaction mixture was stirred for 16 h. The solvent was removed under reduced pressure and the residue was dissolved in *n*-hexane. After filtration, the solvent was removed under reduced pressure. The residue was dissolved in benzene-d_6 and analysed by NMR spectroscopy. Complex **14d** could be isolated as a reddish oil (raw yield: 79 mg, 0.15 mmol, 70%). Several attempts to purify compound **14d** by recrystallisation did not succeed.

^{1}H NMR (499.9 MHz, 305.0 K, C_6D_6): δ = 6.89 (s, 1H, $C_6\underline{H}_2(CH_3)_3$), 6.85 (s, 1H, $C_6\underline{H}_2(CH_3)_3$), 2.58 (s, 3H, $C_6H_2(C\underline{H}_3)_3$), 2.26 (s, 3H, $C_6H_2(C\underline{H}_3)_3$), 2.23 (s, 3H, $C_6H_2(C\underline{H}_3)_3$), 2.15 (s, 6H, $C^{2/3}$-$C\underline{H}_3$), 0.92 (s, 18H, $C^{1/4}$-$Si(C(C\underline{H}_3)_3)(CH_3)_2$), 0.09 (s, 6H, $C^{1/4}$-$Si(C(CH_3)_3)(C\underline{H}_3)_2$), -0.22 (s, 6H, $C^{1/4}$-$Si(C(CH_3)_3)(C\underline{H}_3)_2$).

^{13}C{^{1}H} NMR (125.7 MHz, 305.0 K, C_6D_6): δ = 141.3 ($\underline{C}_6H_2(CH_3)_3$), 137.4 ($\underline{C}_6H_2(CH_3)_3$), 135.6 ($\underline{C}_6H_2(CH_3)_3$), 133.8 ($C^{2/3}$), 127.7 ($\underline{C}_6H_2(CH_3)_3$), 104.8 ($C^{1/4}$), 28.3 ($C^{1/4}$-$Si(C(\underline{C}H_3)_3)(CH_3)_2$), 27.5 ($C_6H_2(\underline{C}H_3)_3$), 25.3 ($C_6H_2(\underline{C}H_3)_3$), 21.4 ($C_6H_2(\underline{C}H_3)_3$), 18.9 ($C^{1/4}$-$Si(\underline{C}(CH_3)_3)(CH_3)_2$), 16.1 ($C^{2/3}$-$\underline{C}H_3$), -2.3 ($C^{1/4}$-$Si(C(CH_3)_3)(\underline{C}H_3)_2$), -2.4 ($C^{1/4}$-$Si(C(CH_3)_3)(\underline{C}H_3)_2$).

^{11}B{^{1}H} NMR (160.4 MHz, 305.0 K, C_6D_6): δ = 30.6 ($C_4\underline{B}$-$C_6H_2(CH_3)_3$).

^{29}Si{^{1}H} INEPT NMR (99.3 MHz, 305.0 K, C_6D_6): δ = -0.3 ($C^{1/4}$-$\underline{Si}(C(CH_3)_3)(CH_3)_2$).

HR-MS (30 eV, EI): m/z = calc.: 512.2521 $C_{27}H_{47}BGeSi_2$
exp.: 512.2512

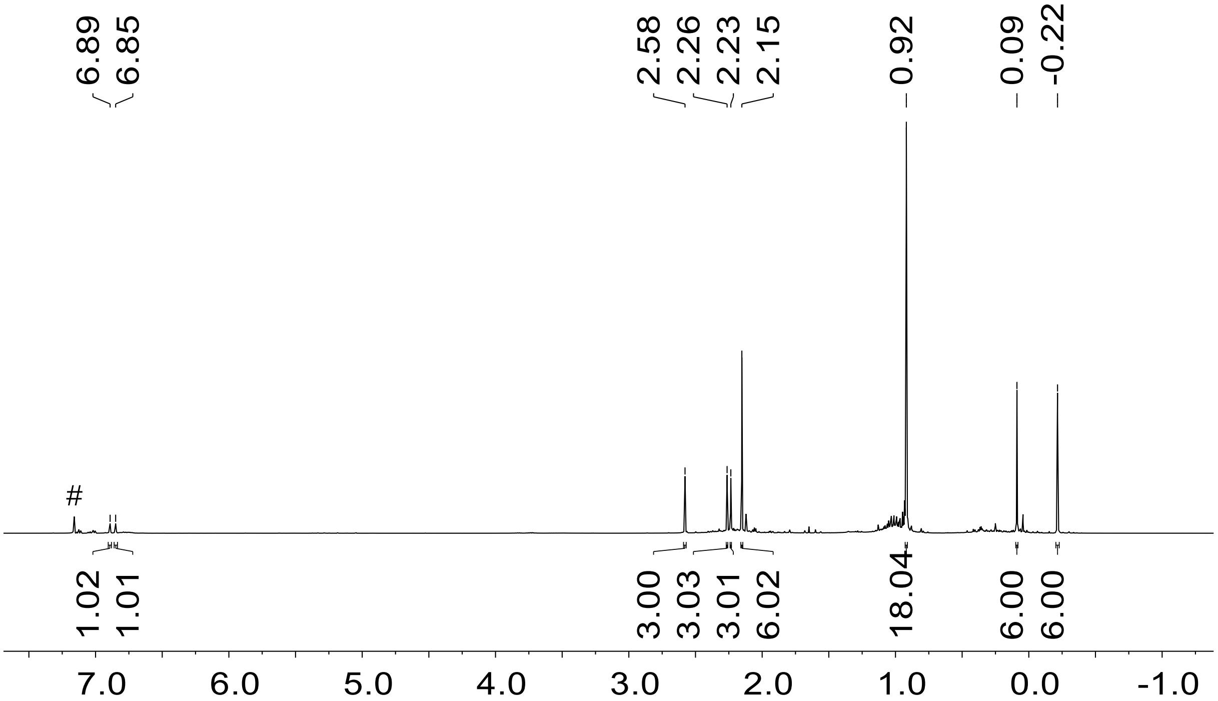

Figure S24. ^{1}H NMR spectrum (499.9 MHz, 305.0 K, C_6D_6) of Mes-substituted boragerma[5]pyramidane **14d**, # = C_6D_5H.

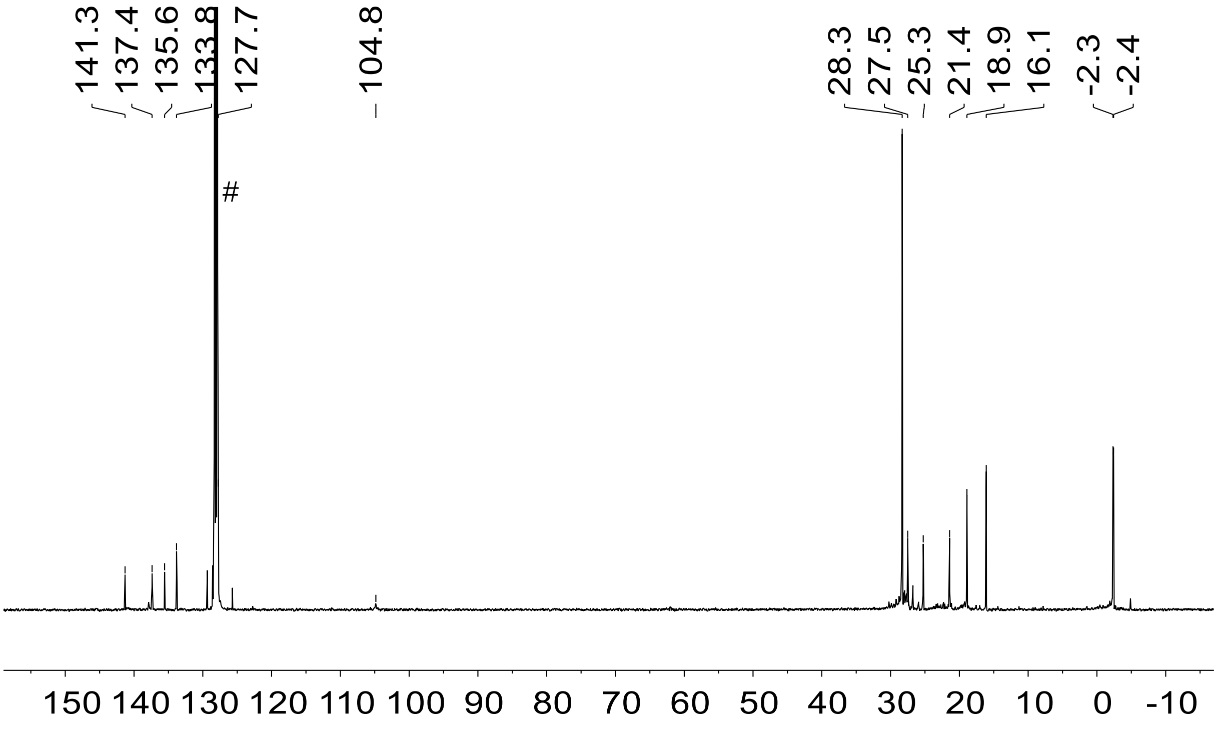

Figure S27. $^{13}C\{^{1}H\}$ NMR spectrum (125.7 MHz, 305.0 K, C_6D_6) of Mes-substituted boragerma[5]pyramidane **14d**, # = C_6D_6.

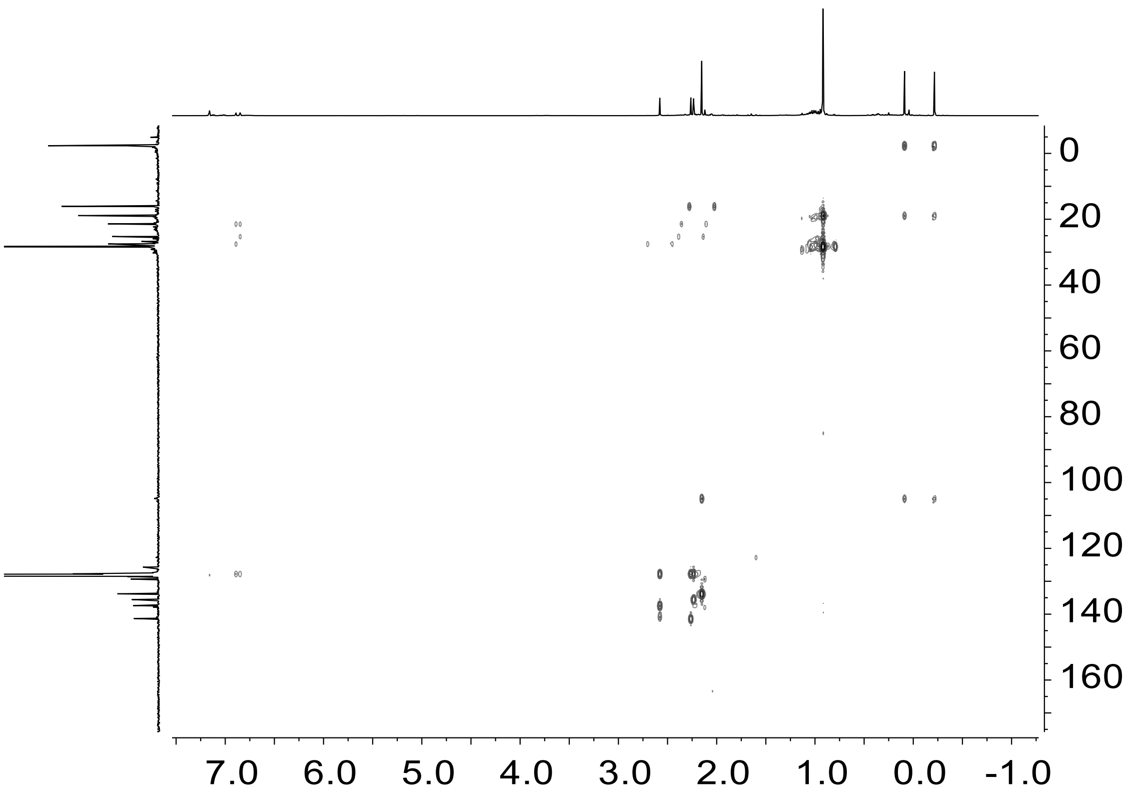

Figure S28. $^{1}H^{13}C$ HMBC NMR spectrum (499.9 MHz, 305.0 K, C_6D_6) of Mes-substituted boragerma[5]pyramidane **14d**.

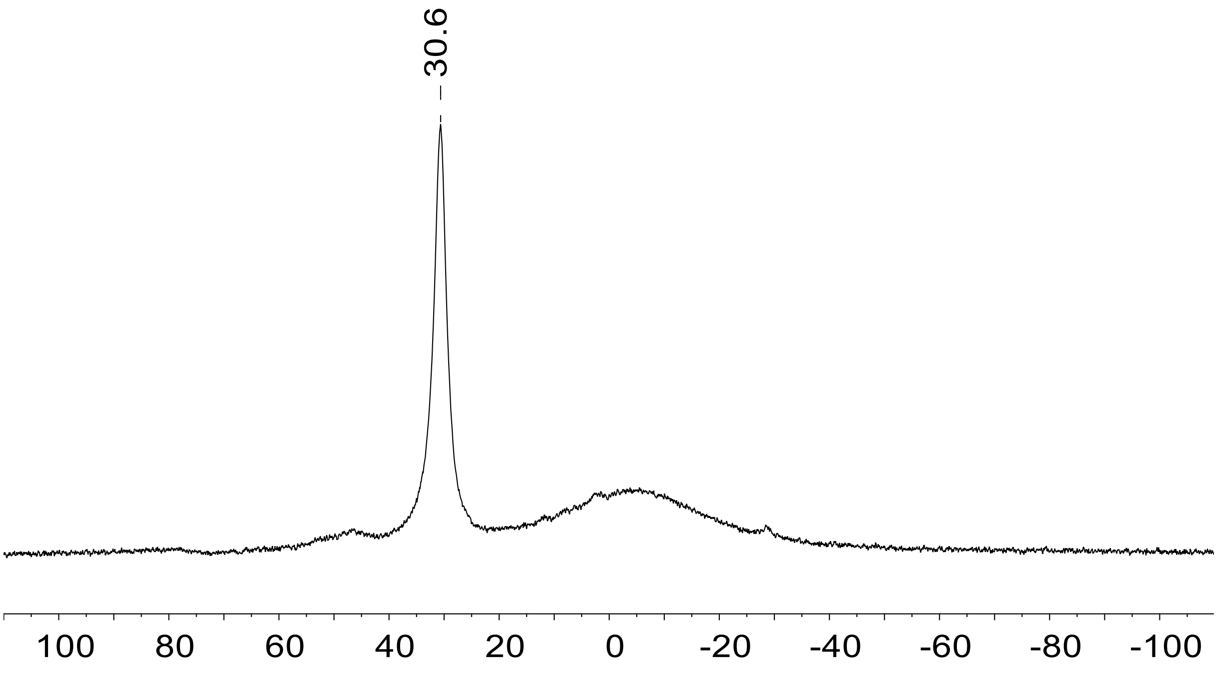

Figure S29. $^{11}B\{^{1}H\}$ NMR spectrum (160.4 MHz, 305.0 K, C_6D_6) of Mes-substituted boragerma[5]pyramidane **14d**.

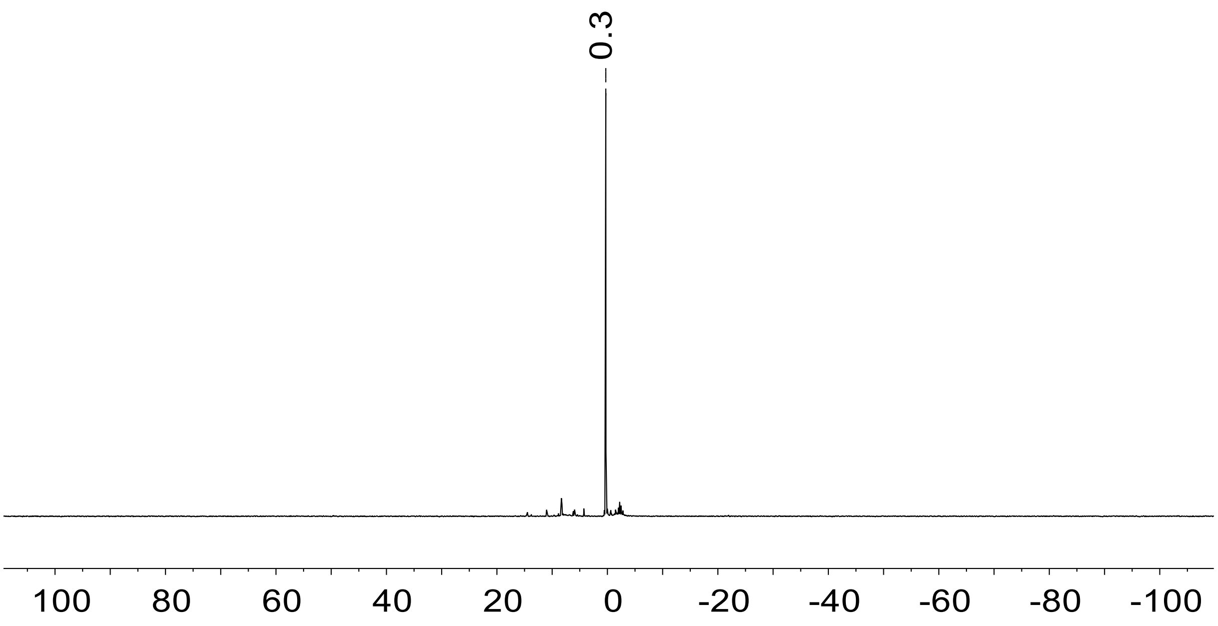

Figure S30. $^{29}Si\{^1H\}$ INEPT NMR spectrum (99.3 MHz, 305.0 K, C_6D_6) of Mes-substituted boragerma[5]pyramidane **14d**.

Synthesis of N^{i}Pr$_2$-substituted boragerma[5]pyramidane 14e

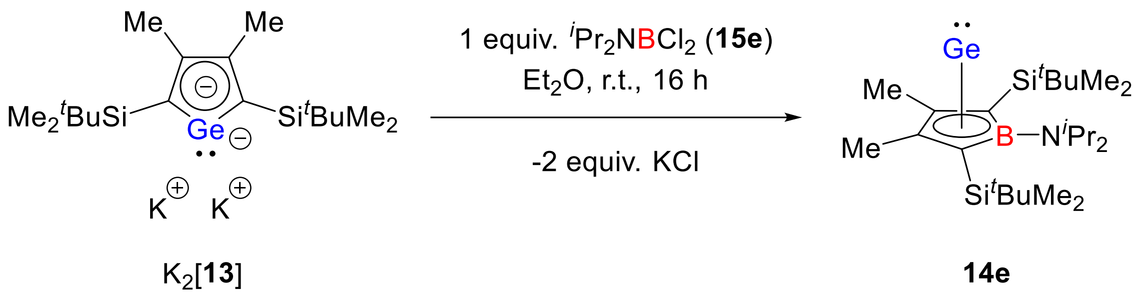

Dipotassium 2,5-bis(-*tert*-butyldimethylsilyl)-3,4-dimethylgermolediide K_2[**13**] (0.50 mmol, 1.00 equiv.) was prepared as described before. The resulting suspension was transferred to a Schlenk flask. A solution of ^{i}Pr$_2$NBCl$_2$ **15e** (91 mg, 0.50 mmol, 1.00 equiv.) in Et_2O (30 mL) was added dropwise to the suspension at room temperature. The reaction mixture was stirred for 16 h. The solvent was removed under reduced pressure and the residue was dissolved in *n*-hexane. After filtration, the solvent was removed under reduced pressure. Complex **14e** could be isolated as yellow crystals from a saturated *n*-pentane solution (yield: 86 mg, 0.17 mmol, 35%).

^{1}H NMR (499.9 MHz, 305.0 K, C_6D_6): δ = 3.92 (sept, $^3J_{H,H}$ = 6.8 Hz, 2H, N-(C$\underline{H}$(CH$_3$)$_2$)$_2$), 2.15 (s, 6H, C$^{2/3}$-C$\underline{H}_3$), 1.31 (d, $^3J_{H,H}$ = 6.8 Hz, 2H, N-(C$\underline{H}$(CH$_3$)$_2$)$_2$), 1.04 (s, 18H, C$^{1/4}$-Si(C(C$\underline{H}_3$)$_3$)(CH$_3$)$_2$), 0.35 (s, 6H, C$^{1/4}$-Si(C(CH$_3$)$_3$)(C$\underline{H}_3$)$_2$), 0.25 (s, 6H, C$^{1/4}$-Si(C(CH$_3$)$_3$)(C$\underline{H}_3$)$_2$).

^{13}C{^{1}H} NMR (125.7 MHz, 305.0 K, C_6D_6): δ = 129.1 (C$^{2/3}$), 89.8 (C$^{1/4}$), 50.2 (N-($\underline{C}$H(CH$_3$)$_2$)$_2$), 29.1 (C$^{1/4}$-Si(C($\underline{C}$H$_3$)$_3$)(CH$_3$)$_2$), 25.7 (N-(CH($\underline{C}$H$_3$)$_2$)$_2$), 19.5 (C$^{1/4}$-Si($\underline{C}$(CH$_3$)$_3$)(CH$_3$)$_2$), 17.0 (C$^{2/3}$-$\underline{C}$H$_3$), 0.8 (C$^{1/4}$-Si(C(CH$_3$)$_3$)($\underline{C}$H$_3$)$_2$), 0.7 (C$^{1/4}$-Si(C(CH$_3$)$_3$)($\underline{C}$H$_3$)$_2$).

^{11}B{^{1}H} NMR (160.4 MHz, 305.0 K, C_6D_6): δ = 33.8 (C$_4\underline{B}$-N^{i}Pr$_2$)

^{29}Si{^{1}H} INEPT NMR (99.3 MHz, 305.0 K, C_6D_6): δ = -1.5 (C$^{1/4}$-$\underline{Si}$(C(CH$_3$)$_3$)(CH$_3$)$_2$).

HR-MS (30 eV, EI): m/z = calc.: 493.2787 $C_{24}H_{50}BGeNSi_2$
exp.: 493.2783

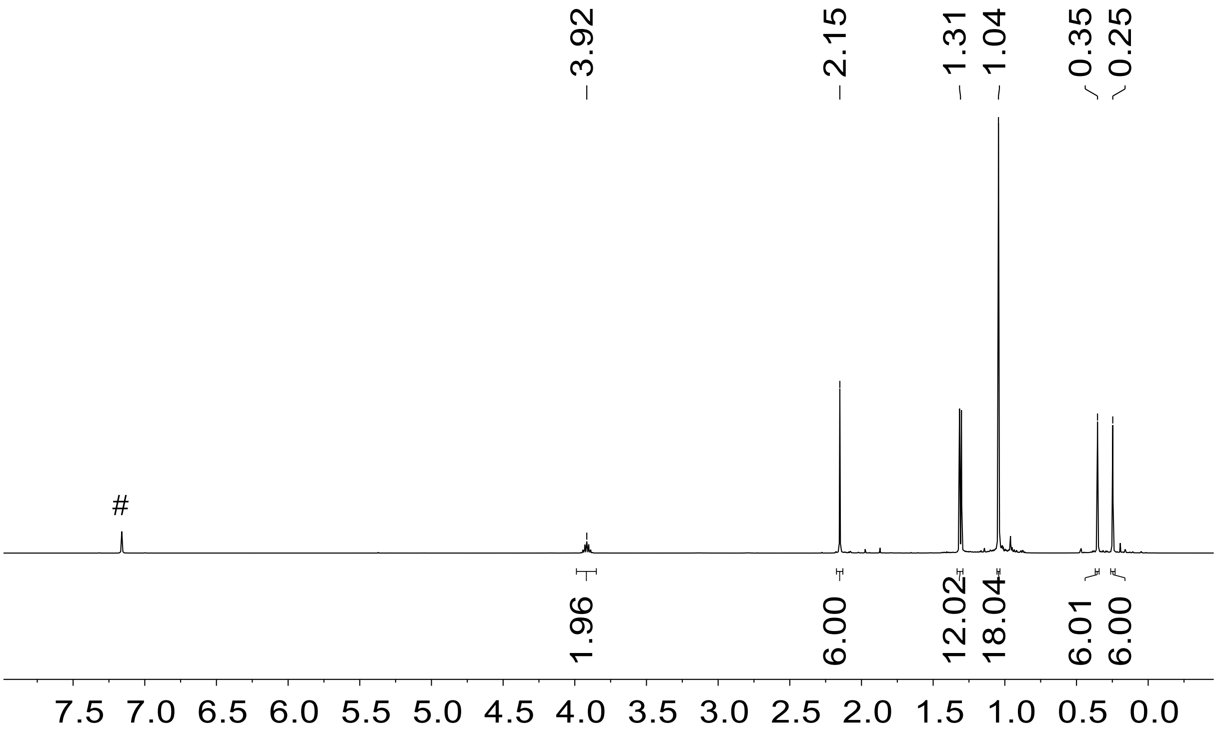

Figure S31. ^{1}H NMR spectrum (499.9 MHz, 305.0 K, C_6D_6) of N^{i}Pr$_2$-substituted boragerma[5]pyramidane **14e**, # = C_6D_5H.

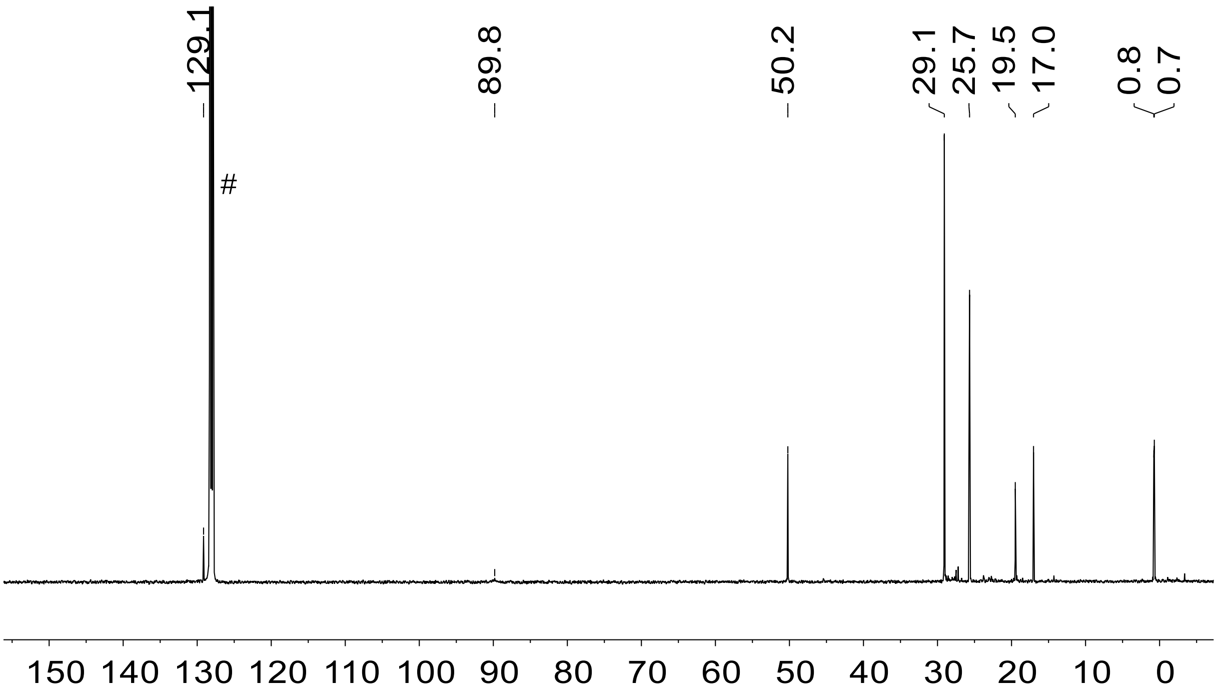

Figure S32. $^{13}C\{^{1}H\}$ NMR spectrum (125.7 MHz, 305.0 K, C_6D_6) of N^{i}Pr$_2$-substituted boragerma[5]pyramidane **14e**, # = C_6D_6.

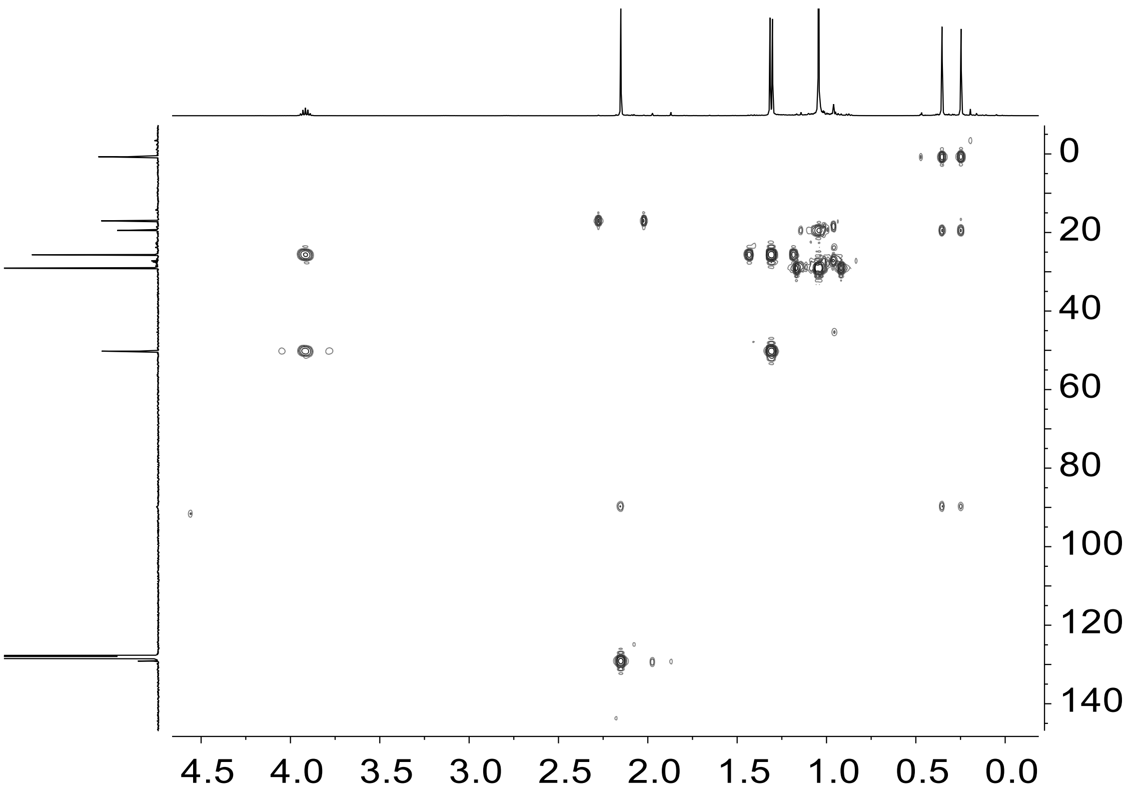

Figure S33. $^{1}H^{13}C$ HMBC NMR spectrum (499.9 MHz, 305.0 K, C_6D_6) of $N^{i}Pr_2$-substituted boragerma[5]pyramidane **14e**.

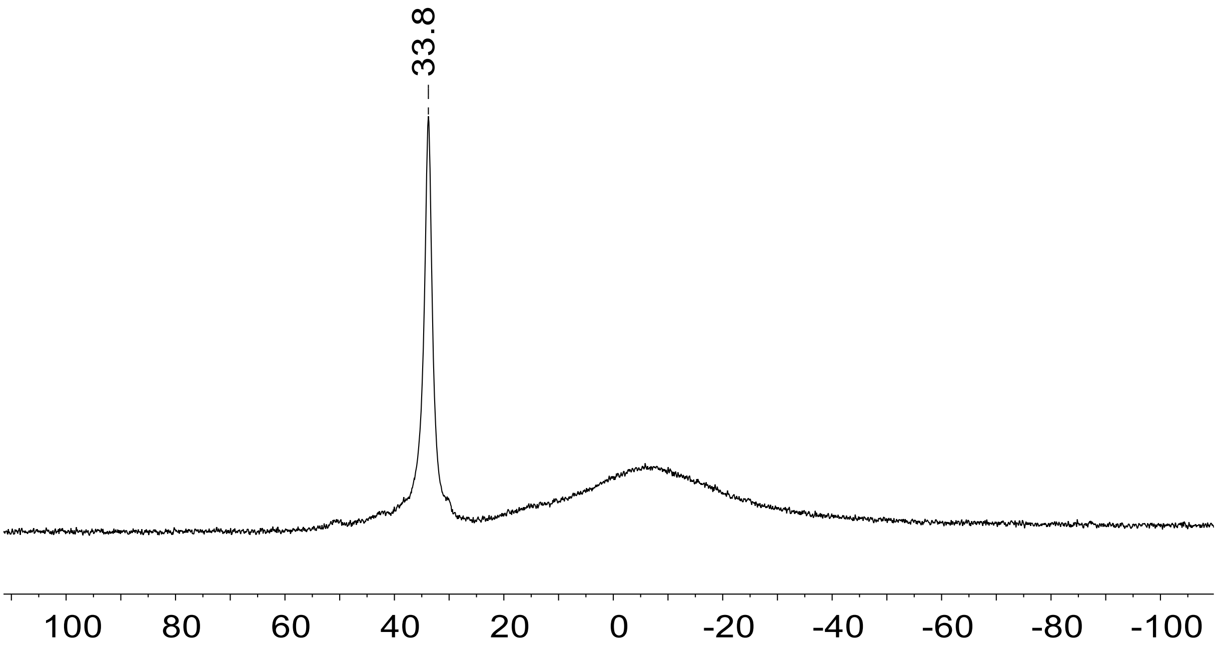

Figure S34. $^{11}B\{^{1}H\}$ NMR spectrum (160.4 MHz, 305.0 K, C_6D_6) of $N^{i}Pr_2$-substituted boragerma[5]pyramidane **14e**.

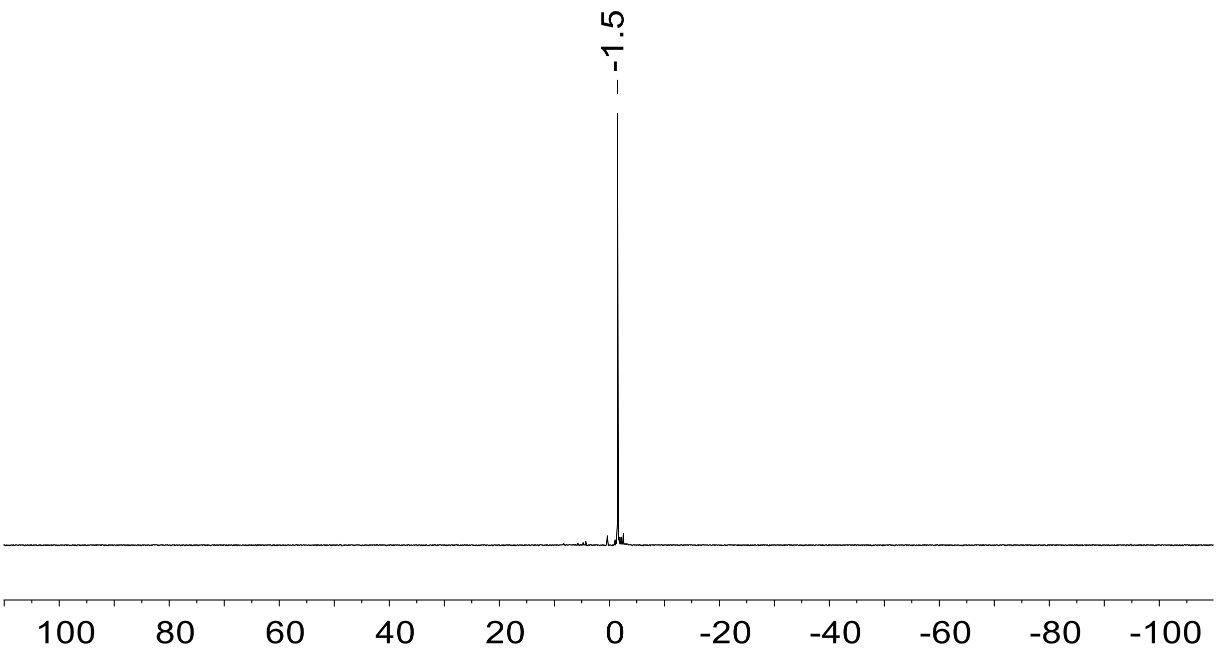

Figure S35. $^{29}Si\{^1H\}$ INEPT NMR spectrum (99.3 MHz, 305.0 K, C_6D_6) of N^{i}Pr$_2$-substituted boragerma[5]pyramidane **14e**.

Synthesis of NCy_2-substituted boragerma[5]pyramidane 14f

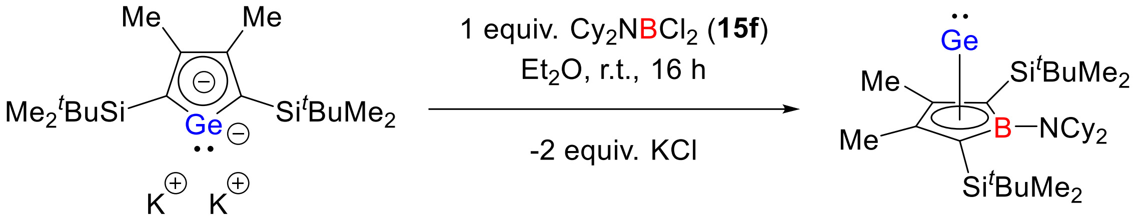

K_2[**13**] **14f**

Dipotassium 2,5-bis(-*tert*-butyldimethylsilyl)-3,4-dimethylgermolediide K_2[**13**] (0.50 mmol, 1.00 equiv.) was prepared as described before. The resulting suspension was transferred to a Schlenk flask. A solution of Cy_2NBCl_2 **15f** (131 mg, 0.50 mmol, 1.00 equiv.) in Et_2O (30 mL) was added dropwise to the suspension at room temperature. The reaction mixture was stirred for 16 h. The solvent was removed under reduced pressure and the residue was dissolved in *n*-hexane. After filtration, the solvent was removed under reduced pressure. Complex **14f** could be isolated as yellow crystals from a saturated *n*-pentane solution (yield: 166 mg, 0.29 mmol, 58%).

^{1}H NMR (499.9 MHz, 305.0 K, C_6D_6): δ = 3.23-3.17 (m, 2H, $N(C\underline{H}\text{-}C_5H_{10})_2$), 2.30-2.28 (m, 4H, $N(CH\text{-}C_5\underline{H}_{10})_2$), 2.13 (s, 6H, $C^{2/3}\text{-}C\underline{H}_3$), 1.78-1.75 (m, 4H, $N(CH\text{-}C_5\underline{H}_{10})_2$), 1.63-1.60 (m, 2H, $N(CH\text{-}C_5\underline{H}_{10})_2$), 1.46-1.28 (m, 8H, $N(CH\text{-}C_5\underline{H}_{10})_2$), 1.19-1.10 (m, 2H, $N(CH\text{-}C_5\underline{H}_{10})_2$), 1.05 (s, 18H, $C^{1/4}\text{-}Si(C(C\underline{H}_3)_3)(CH_3)_2$), 0.40 (s, 6H, $C^{1/4}\text{-}Si(C(CH_3)_3)(C\underline{H}_3)_2$), 0.37 (s, 6H, $C^{1/4}\text{-}Si(C(CH_3)_3)(C\underline{H}_3)_2$).

^{13}C{^{1}H} NMR (125.7 MHz, 305.0 K, C_6D_6): δ = 129.6 ($C^{2/3}$), 102.4 ($C^{1/4}$), 57.8 ($N(\underline{C}H\text{-}C_5H_{10})_2$), 36.2 ($N(CH\text{-}\underline{C}_5H_{10})_2$), 29.3 ($C^{1/4}\text{-}Si(C(\underline{C}H_3)_3)(CH_3)_2$), 27.0 ($N(CH\text{-}\underline{C}_5H_{10})_2$), 27.0 ($N(CH\text{-}\underline{C}_5H_{10})_2$), 19.5 ($C^{1/4}\text{-}Si(\underline{C}(CH_3)_3)(CH_3)_2$), 16.7 ($C^{2/3}\text{-}\underline{C}H_3$), -0.4 ($C^{1/4}\text{-}Si(C(CH_3)_3)(\underline{C}H_3)_2$), -0.5 ($C^{1/4}\text{-}Si(C(CH_3)_3)(\underline{C}H_3)_2$).

^{11}B{^{1}H} NMR (160.4 MHz, 305.0 K, C_6D_6): δ = 32.1 ($C_4\underline{B}\text{-}NCy_2$).

^{29}Si{^{1}H} INEPT NMR (99.3 MHz, 305.0 K, C_6D_6): δ = -2.1 ($C^{1/4}\text{-}\underline{Si}(C(CH_3)_3)(CH_3)_2$).

HR-MS (30 eV, EI): m/z = calc.: 573.3413 $C_{30}H_{58}BGeNSi_2$

exp.: 573.3407

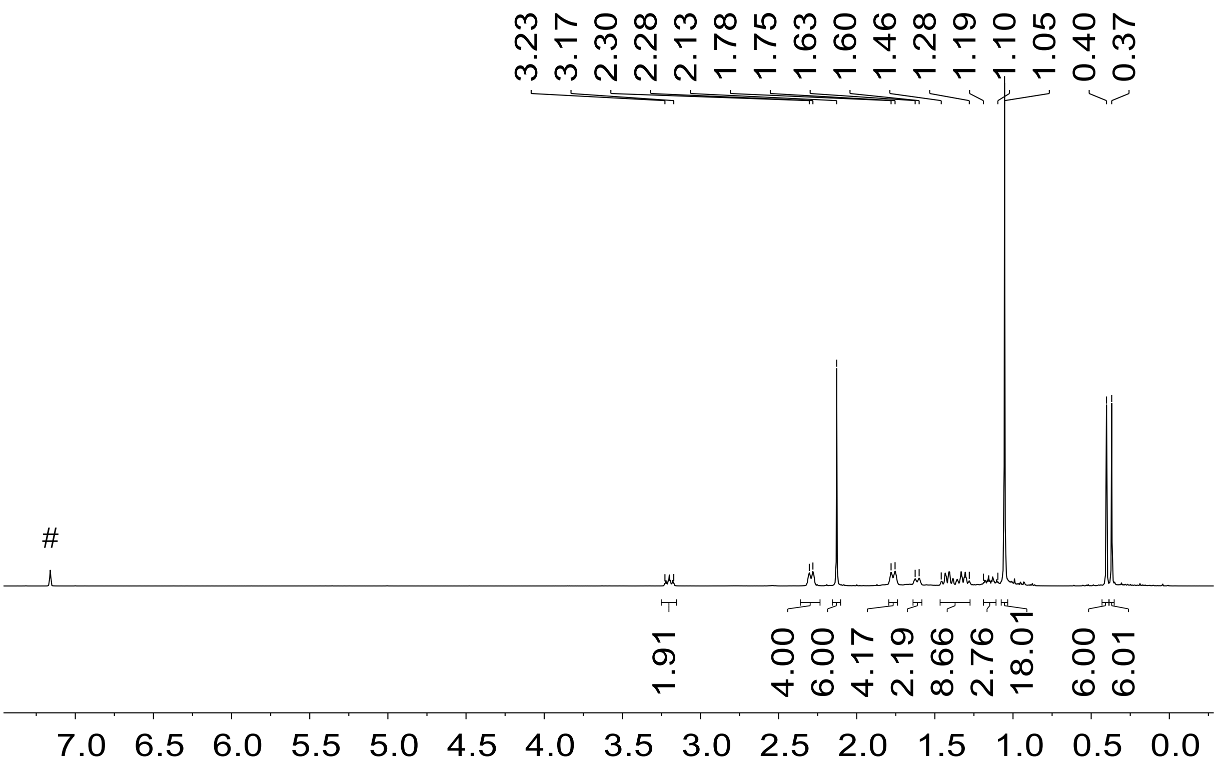

Figure S36. ^{1}H NMR spectrum (499.9 MHz, 305.0 K, C_6D_6) of NCy_2-substituted boragerma[5]pyramidane **14f**, # = C_6D_5H.

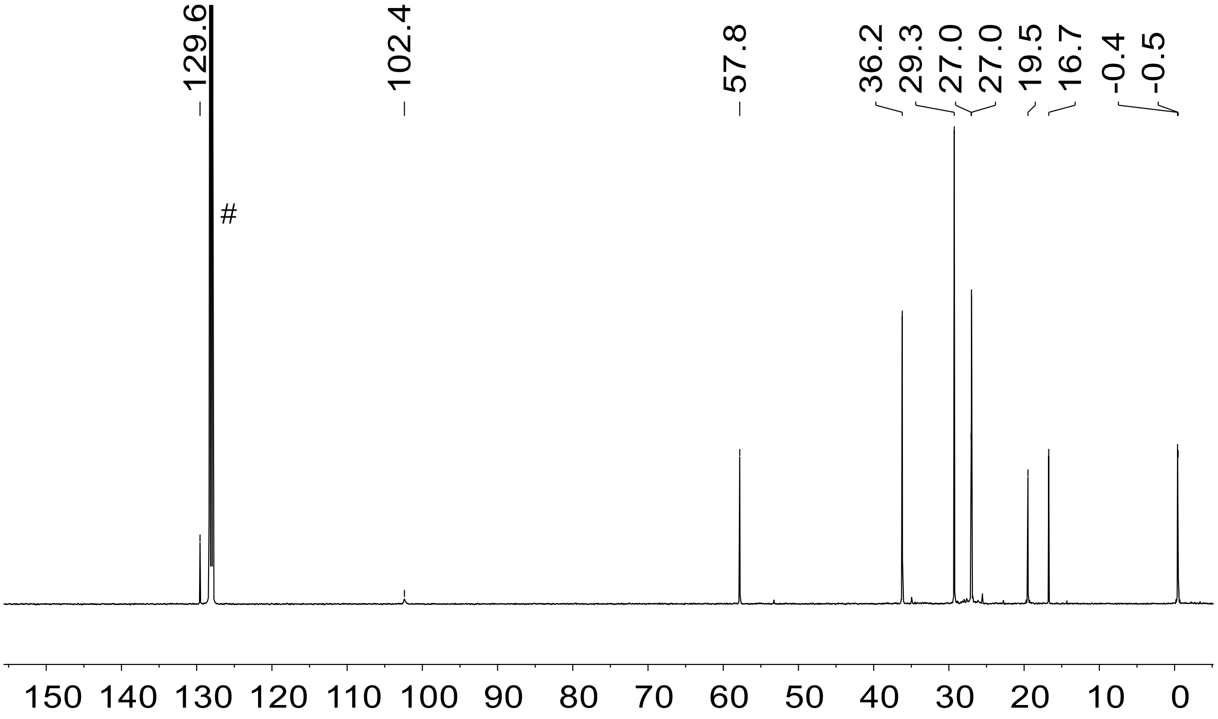

Figure S37. $^{13}C\{^{1}H\}$ NMR spectrum (125.7 MHz, 305.0 K, C_6D_6) of NCy_2-substituted boragerma[5]pyramidane **14e**, # = C_6D_6.

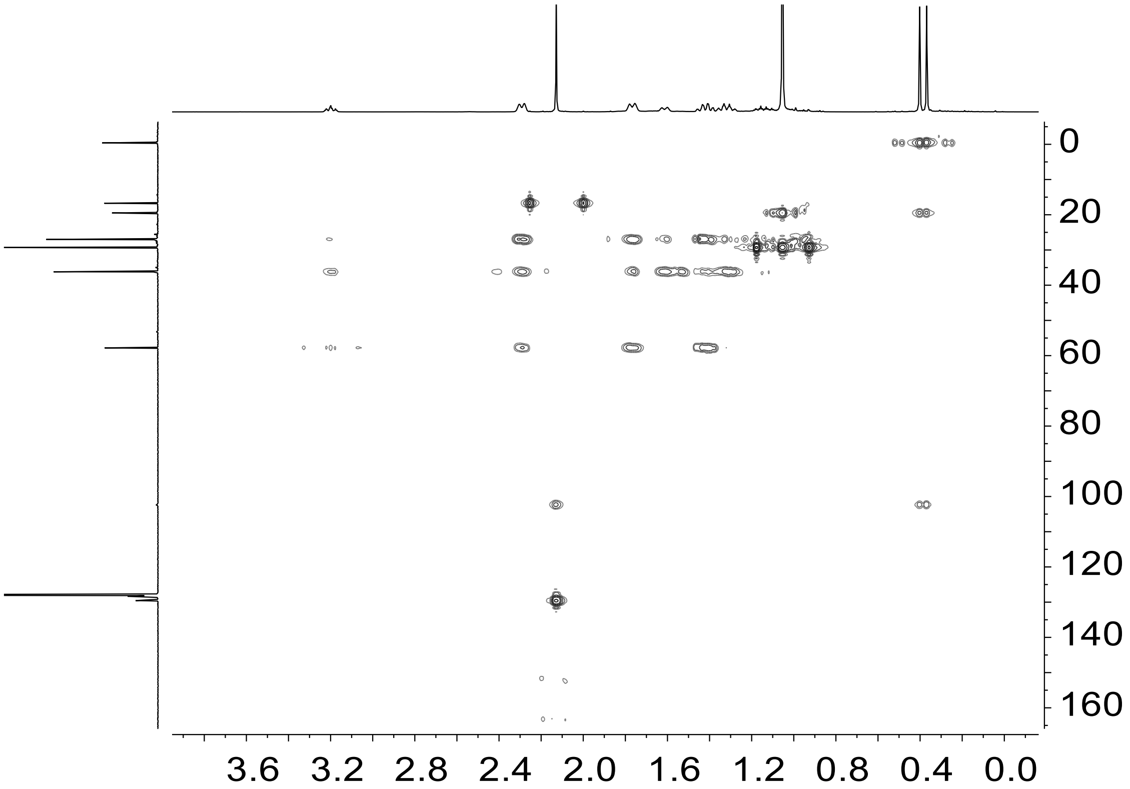

Figure S38. $^{1}H^{13}C$ HMBC NMR spectrum (499.9 MHz, 305.0 K, C_6D_6) of NCy_2-substituted boragerma[5]pyramidane **14f**.

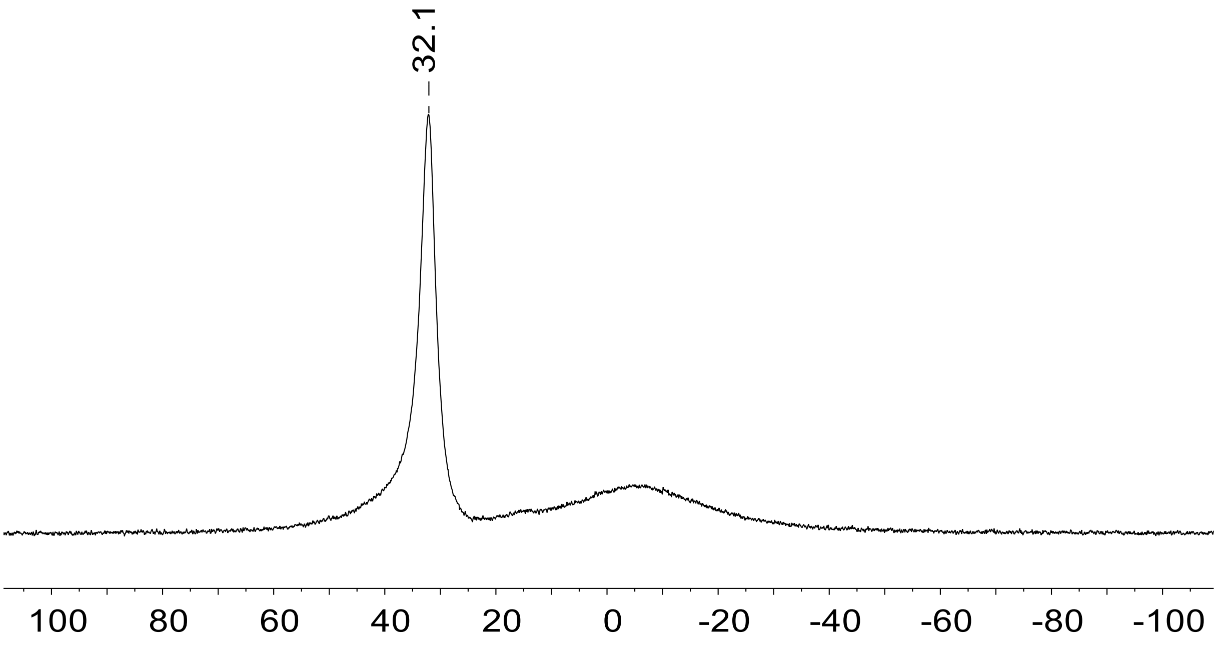

Figure S39. $^{11}B\{^{1}H\}$ NMR spectrum (160.4 MHz, 305.0 K, C_6D_6) of NCy_2-substituted boragerma[5]pyramidane **14f**.

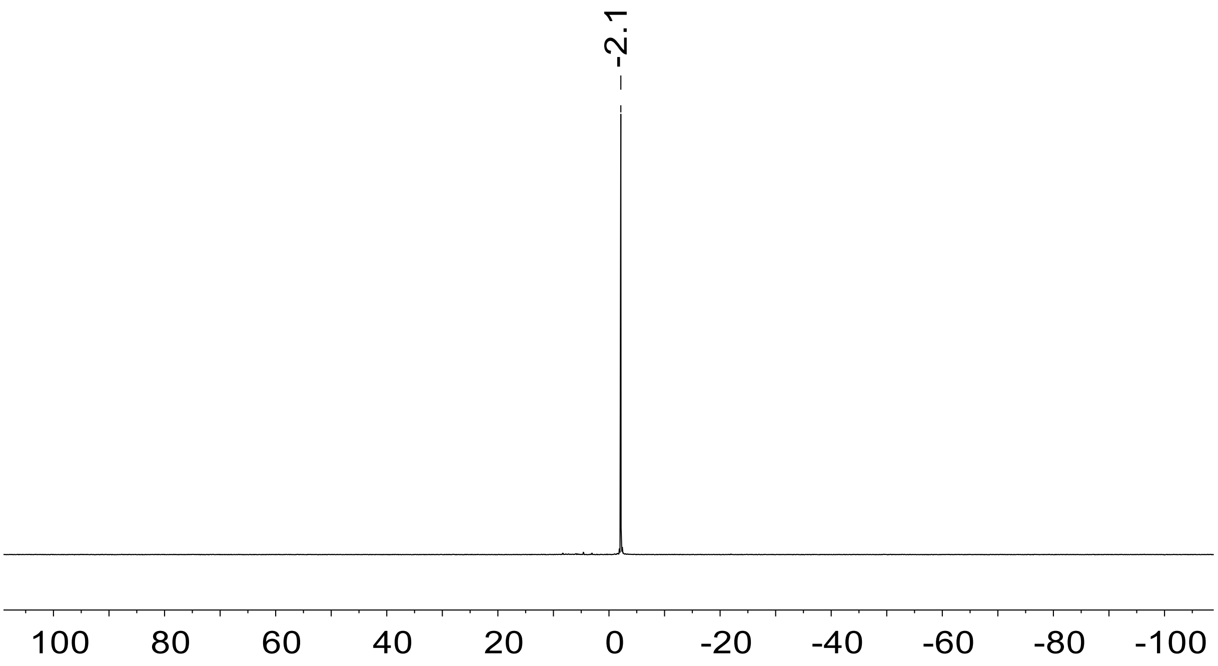

Figure S40. $^{29}Si\{^1H\}$ INEPT NMR spectrum (99.3 MHz, 305.0 K, C_6D_6) of NCy_2-substituted boragerma[5]pyramidane **14f**.

Synthesis of ferrocenyl-substituted boragerma[5]pyramidane 14g

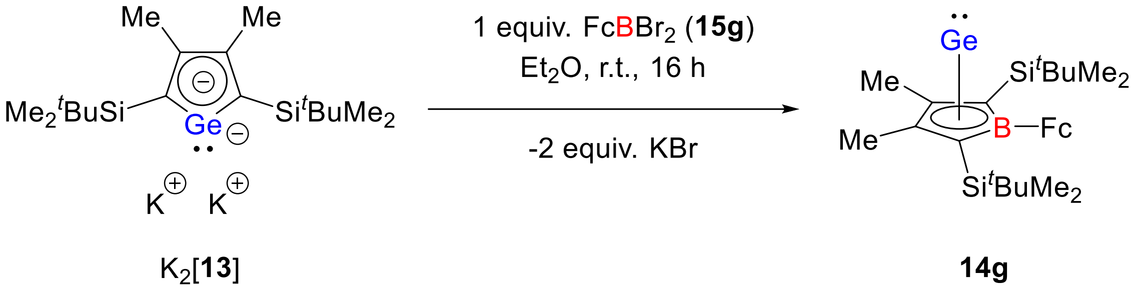

Dipotassium 2,5-bis(*tert*-butyldimethylsilyl)-3,4-dimethylgermolediide K_2[**13**] (0.22 mmol, 1.00 equiv.) was prepared as described before. The resulting suspension was transferred to a Schlenk flask. A solution of $FcBBr_2$ **15g** (79 mg, 0.22 mmol, 1.00 equiv.) in Et_2O (30 mL) was added dropwise to the suspension at room temperature. The reaction mixture was stirred for 16 h. The solvent was removed under reduced pressure and the residue was dissolved in *n*-hexane. After filtration, the solvent was removed under reduced pressure. Compound **14g** could be isolated as red crystals from a saturated *n*-pentane solution (yield: 69 mg, 0.11 mmol, 51%).

^{1}H NMR (499.9 MHz, 305.0 K, C_6D_6): δ = 4.66-4.64 (m, 2H, C_4B-$C_5\underline{H}_4$), 4.34-4.32 (m, 2H, C_4B-$C_5\underline{H}_4$), 4.13 (s, 5H, Fe-$C_5\underline{H}_5$), 2.16 (s, 6H, $C^{2/3}$-$C\underline{H}_3$), 1.15 (s, 18H, $C^{1/4}$-Si(C($C\underline{H}_3$)$_3$)(CH_3)$_2$), 0.43 (s, 6H, $C^{1/4}$-Si(C(CH_3)$_3$)($C\underline{H}_3$)$_2$), 0.26 (s, 6H, $C^{1/4}$-Si(C(CH_3)$_3$)($C\underline{H}_3$)$_2$).

^{13}C{^{1}H} NMR (125.7 MHz, 305.0 K, C_6D_6): δ = 134.6 ($C^{2/3}$), 101.1 ($C^{1/4}$), 77.6 (C_4B-$\underline{C}_5H_4$), 69.4 (Fe-$\underline{C}_5H_5$), 69.2 (C_4B-$\underline{C}_5H_4$), 29.3 ($C^{1/4}$-Si(C($\underline{C}H_3$)$_3$)(CH_3)$_2$), 19.2 ($C^{1/4}$-Si($\underline{C}$(CH_3)$_3$)(CH_3)$_2$), 16.9 ($C^{2/3}$-$\underline{C}H_3$), 2.2 ($C^{1/4}$-Si(C(CH_3)$_3$)($\underline{C}H_3$)$_2$), 2.2 ($C^{1/4}$-Si(C(CH_3)$_3$)($\underline{C}H_3$)$_2$).
The ^{13}C NMR signal of the *α*-carbon atom bonded to the boron atom could not be determined.

^{11}B{^{1}H} NMR (160.4 MHz, 305.0 K, C_6D_6): δ = 32.4 ($C_4\underline{B}$-Fc).

^{29}Si{^{1}H} INEPT NMR (99.3 MHz, 305.0 K, C_6D_6): δ = -0.4 ($C^{1/4}$-$\underline{Si}$(C(CH_3)$_3$)(CH_3)$_2$).

HR-MS (30 eV, EI): m/z = calc.: 578.1714 $C_{28}H_{45}BFeGeSi_2$
exp.: 578.1709

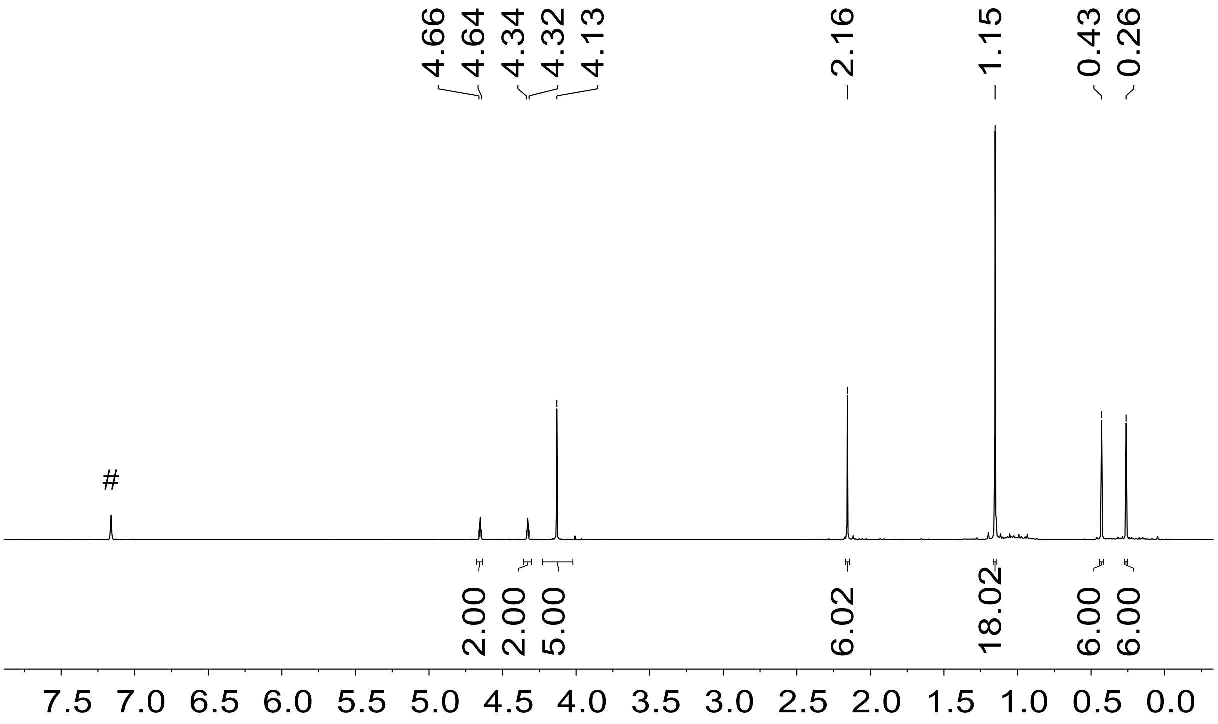

Figure S41. ^{1}H NMR spectrum (499.9 MHz, 305.0 K, C_6D_6) of Fc-substituted boragerma[5]pyramidane **14g**, # = C_6D_5H.

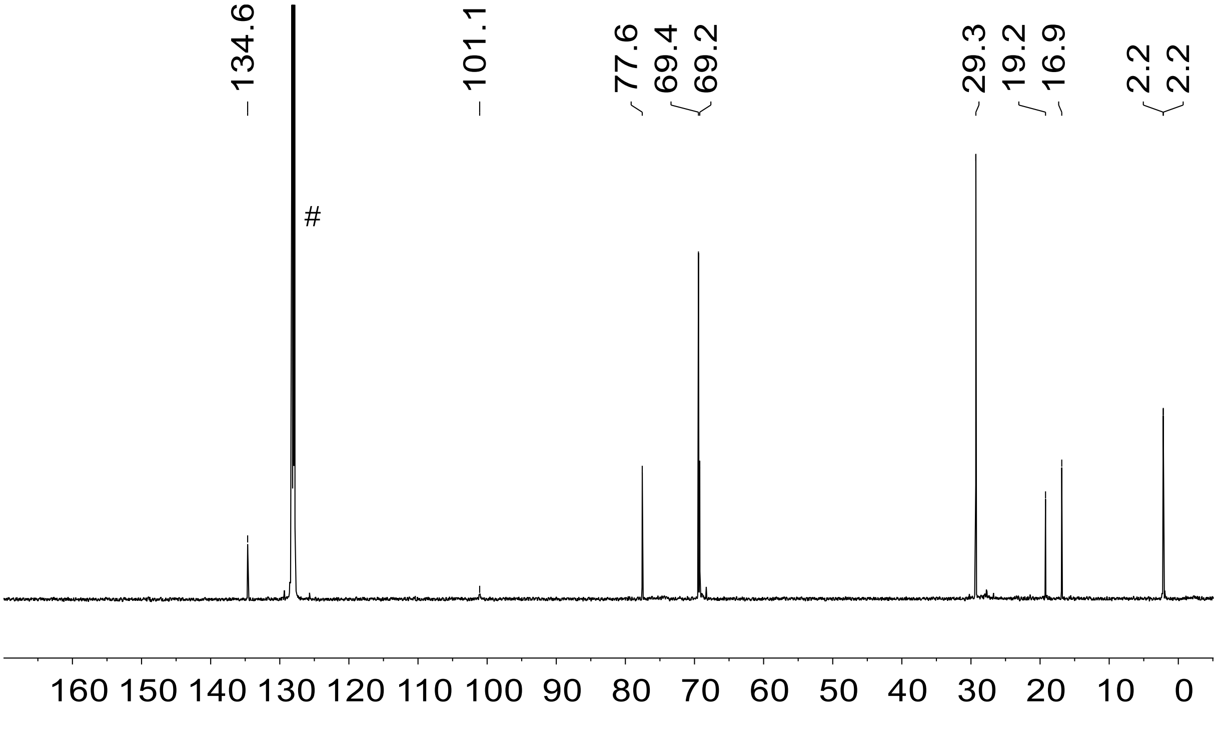

Figure S42. $^{13}C\{^{1}H\}$ NMR spectrum (125.7 MHz, 305.0 K, C_6D_6) of Fc-substituted boragerma[5]pyramidane **14g**, # = C_6D_6.

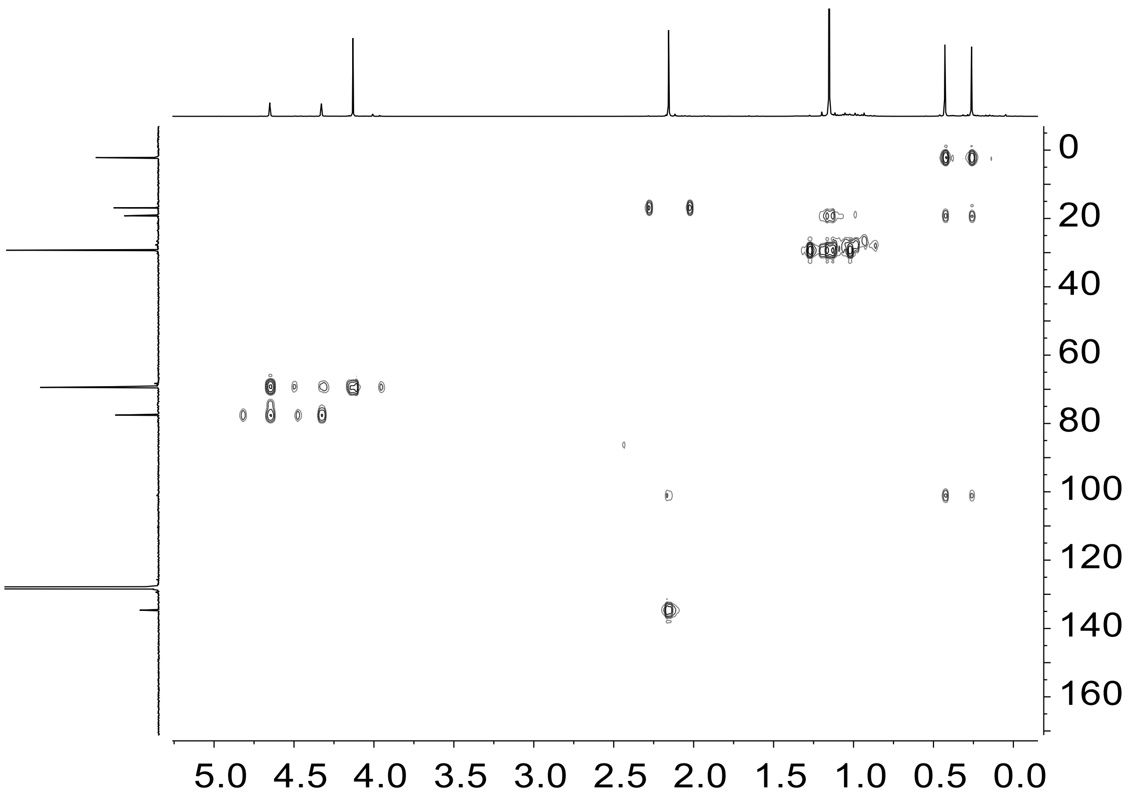

Figure S43. $^{1}H^{13}C$ HMBC NMR spectrum (499.9 MHz, 305.0 K, C_6D_6) of Fc-substituted boragerma[5]pyramidane **14g**.

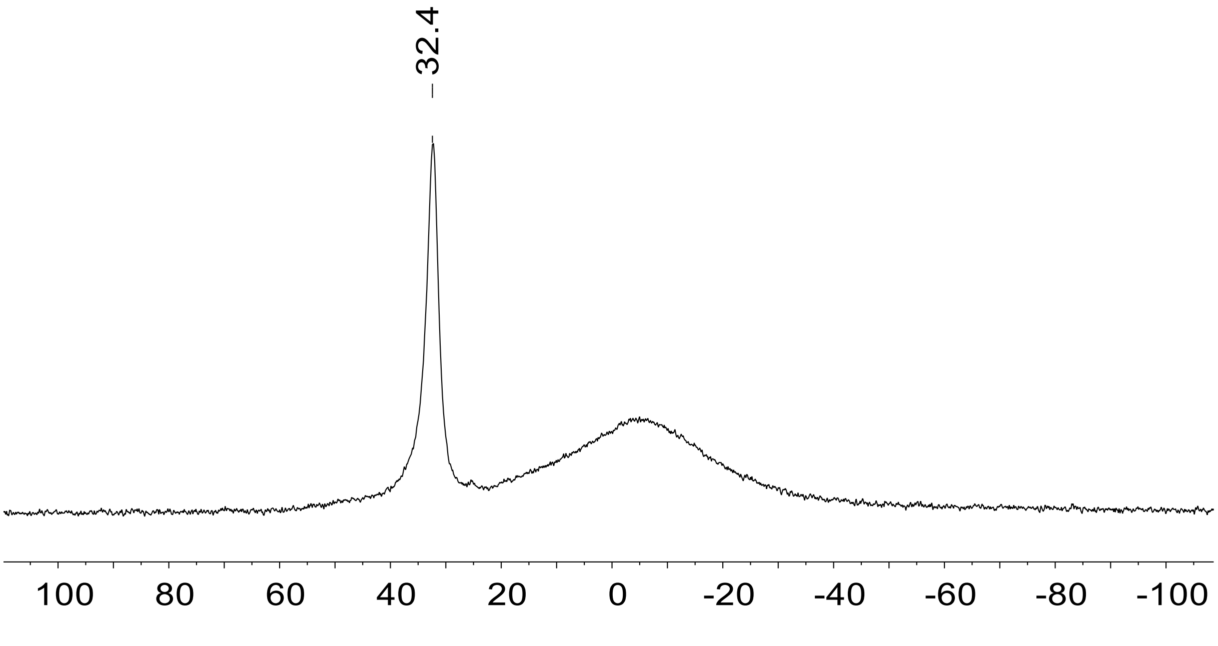

Figure S44. $^{11}B\{^{1}H\}$ NMR spectrum (160.4 MHz, 305.0 K, C_6D_6) of Fc-substituted boragerma[5]pyramidane **14g**.

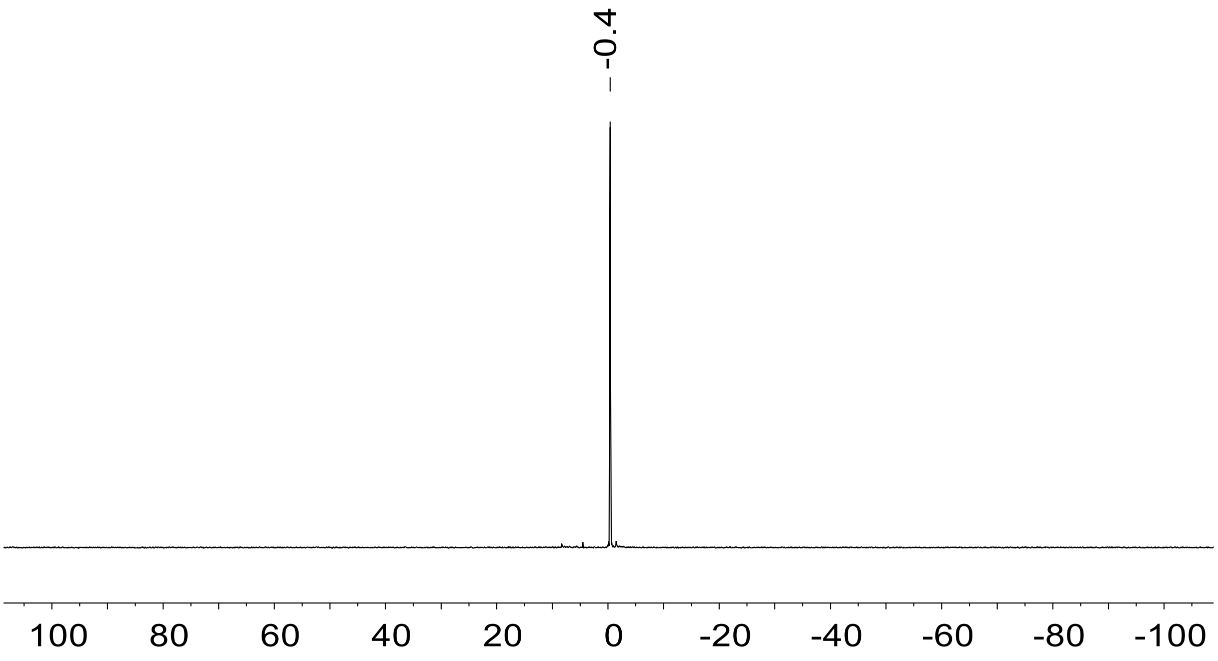

Figure S25. $^{29}Si\{^{1}H\}$ INEPT NMR spectrum (99.3 MHz, 305.0 K, C_6D_6) of Fc-substituted boragerma[5]pyramidane **14g**.

Synthesis of NCy_2-substituted borasila[5]pyramidane 20f

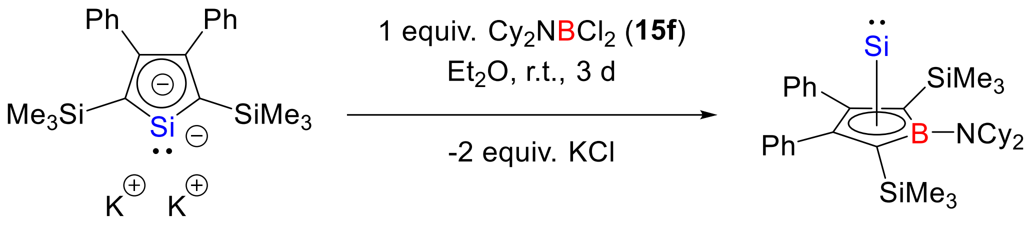

K_2[**19**] **20f**

Dipotassium 2,5-bis(trimethylsilyl)-3,4-diphenylsiloledIide K_2[**19**] (0.50 mmol, 1.00 equiv.) was prepared as described before. The resulting suspension was transferred to a Schlenk flask. A solution of Cy_2NBCl_2 **15f** (131 mg, 0.50 mmol, 1.00 equiv.) in Et_2O (30 mL) was added dropwise to the suspension at room temperature. The reaction mixture was stirred for 16 h. The solvent was removed under reduced pressure and the residue was dissolved in *n*-hexane. After filtration, the solvent was removed under reduced pressure. The residue was dissolved in benzene-d_6 and analysed by NMR spectroscopy. Complex **20f** could be isolated as a dark-yellow oil (raw yield: 218 mg, 0.38 mmol, 77%). Several attempts to purify compound **20f** by recrystallisation did not succeed, due to the high solubility of compound **20f** in all tested solvents.

^{1}H NMR (499.9 MHz, 305.0 K, C_6D_6): δ = 7.21-7.19 (m, 4H, $C_6\underline{H}_5$), 6.86-6.84 (m, 6H, $C_6\underline{H}_5$), 3.58-3.52 (m, 2H, N(C$\underline{H}$-C_5H_{10})$_2$), 2.23-2.21 (m, 4H, N(C$\underline{H}$-C_5H_{10})$_2$), 1.91-1.08 (m, 16H, N(C$\underline{H}$-C_5H_{10})$_2$), 0.12 (s, 18H, $C^{1/4}$-Si(C$\underline{H}_3$)$_3$).

^{13}C{^{1}H} NMR (125.7 MHz, 305.0 K, C_6D_6): δ = 136.8 ($C^{2/3}$), 135.9 (C^{ipso}), 132.2 ($\underline{C}_6H_5$), 127.7 ($\underline{C}_6H_5$), 127.6 ($\underline{C}_6H_5$), 88.7 ($C^{1/4}$), 59.4 (N($\underline{C}$H-C_5H_{10})$_2$), 36.8 (N(CH-$\underline{C}_5H_{10}$)$_2$), 27.4 (N(CH-$\underline{C}_5H_{10}$)$_2$), 26.7 (N(CH-$\underline{C}_5H_{10}$)$_2$), 2.7 ($C^{1/4}$-Si($\underline{C}H_3$)$_3$).

^{11}B{^{1}H} NMR (160.4 MHz, 305.0 K, C_6D_6): δ = 35.0 ($C_4\underline{B}$-NCy_2).

^{29}Si{^{1}H} NMR (99.3 MHz, 305.0 K, C_6D_6): δ = -9.4 ($C^{1/4}$-$\underline{Si}$(CH_3)$_3$), -337.5 (Si(II)).

Note: No satisfactory results could be obtained for Mass Spectrometry (**MS**) or Elemental Analysis (**EA**).

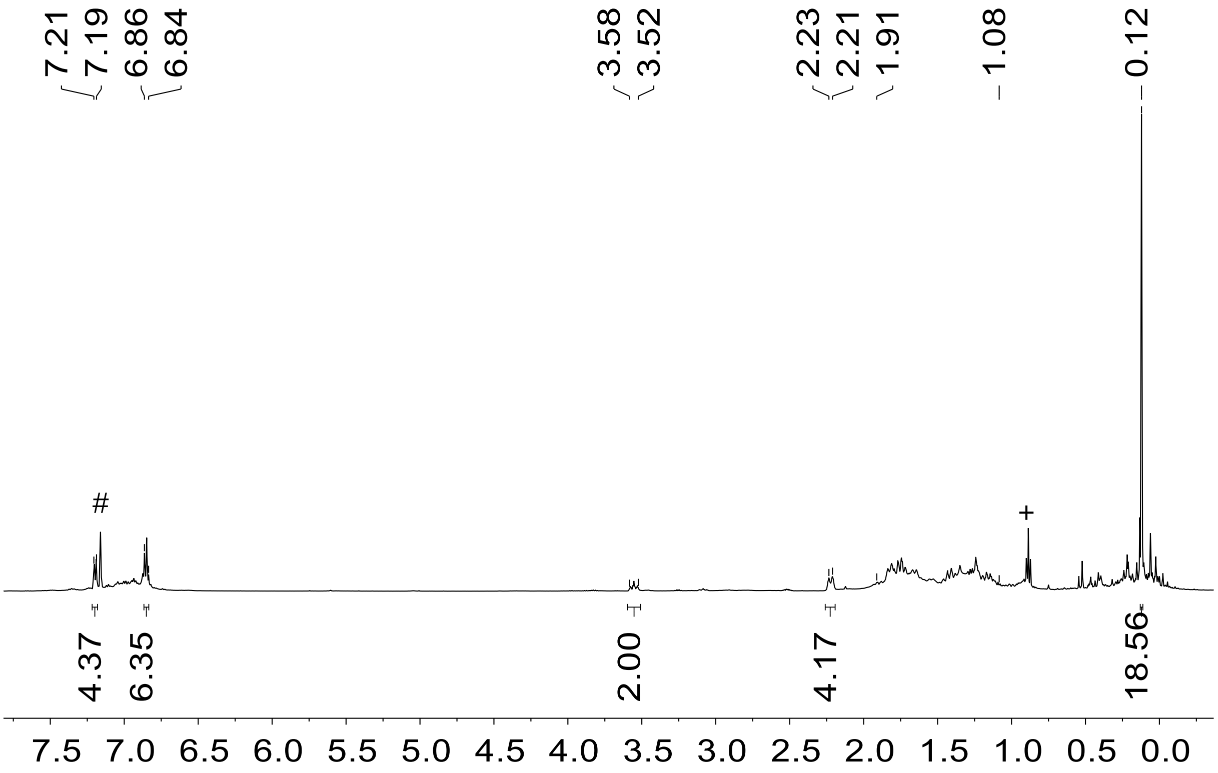

Figure S46. ^{1}H NMR spectrum (499.9 MHz, 305.0 K, C_6D_6) of NCy_2-substituted borasila[5]pyramidane **20f**, # = C_6D_5H, + = *n*-hexane.

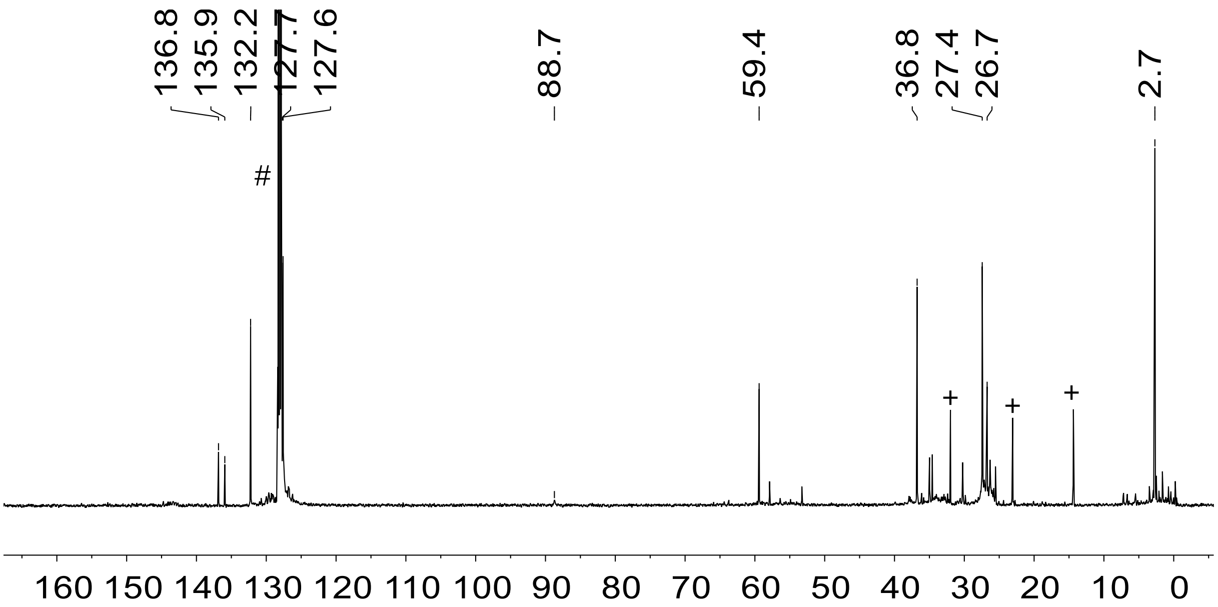

Figure S47. $^{13}C\{^{1}H\}$ NMR spectrum (125.7 MHz, 305.0 K, C_6D_6) of NCy_2-substituted borasila[5]pyramidane **20f**, # = C_6D_6, + = *n*-hexane.

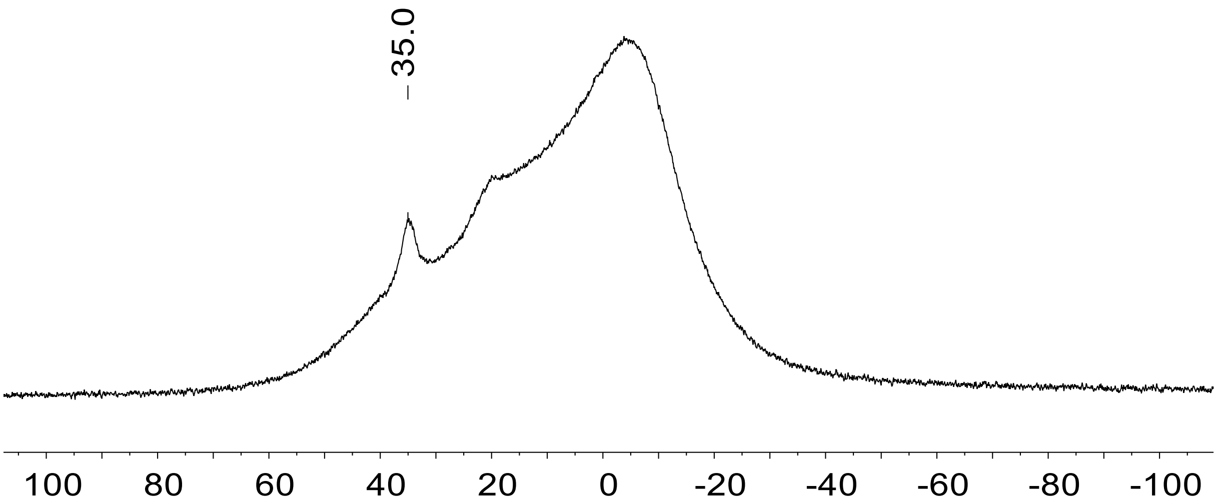

Figure S48. $^{11}B\{^1H\}$ NMR (160.4 MHz, 305.0 K, C_6D_6) of NCy_2-substituted borasila[5]pyramidane **20f**.

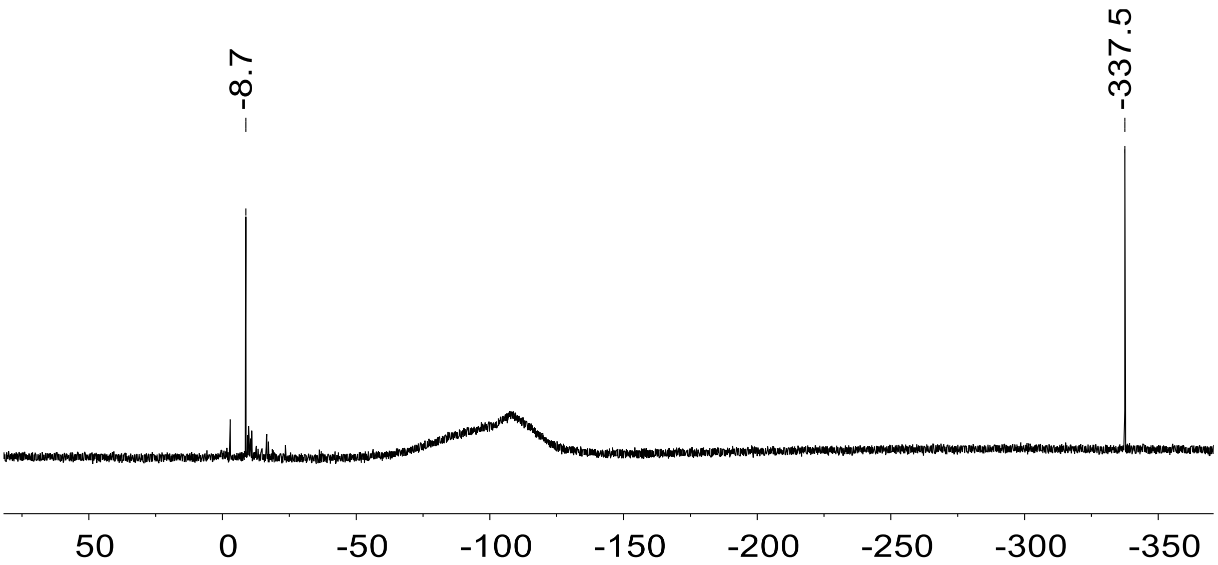

Figure S49. $^{29}Si\{^1H\}$ NMR spectrum (99.3 MHz, 305.0 K, C_6D_6) of NCy_2-substituted borasila[5]pyramidane **20f**.

Synthesis of ferrocenyl-substituted borole 25

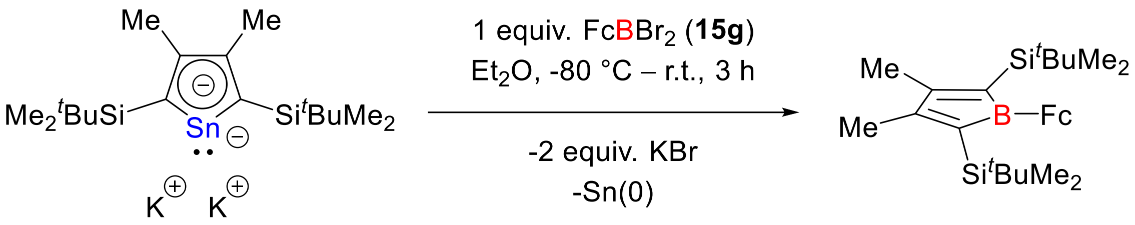

K_2[**35**] **25**

Dipotassium 2,5-bis(-*tert*-butyldimethylsilyl)-3,4-dimethylstannolediide K_2[**35**] (0.30 mmol, 1.00 equiv.) was prepared following a literature procedure.[10] The resulting suspension was transferred to a Schlenk flask. A solution of $FcBBr_2$ **15g** (107 mg, 0.30 mmol, 1.00 equiv.) in Et_2O (40 mL) was added slowly via a *Teflon* tube at -80 °C. The reaction mixture was stirred for 2 h while the cooling bath was allowed to warm to room temperature. Afterwards, it was stirred at room temperature for 1 h. The solvent was removed under reduced pressure and the residue was dissolved in *n*-pentane. After filtration, the solvent was removed under reduced pressure and the crude product **25** was obtained as red oily solid (yield: 144 mg, 0.29 mmol, 95%). Red crystals of compound **25** could be isolated from a saturated *n*-pentane solution at -30 °C (yield: 84 mg, 0.17 mmol, 55%).

^{1}H NMR (499.9 MHz, 298.1 K, C_6D_6): δ = 4.74-4.66 (m, 2H, C_4B-$C_5\underline{H}_4$), 4.53-4.46 (m, 2H, C_4B-$C_5\underline{H}_4$), 4.01 (s, 5H, Fe-$C_5\underline{H}_5$), 1.99 (s, 6H, $C^{2/3}$-$C\underline{H}_3$), 1.11 (s, 18H, $C^{1/4}$-Si(C($C\underline{H}_3$)$_3$)(CH_3)$_2$), 0.35 (s, 12H, $C^{1/4}$-Si(C(CH_3)$_3$)($C\underline{H}_3$)$_2$).

^{13}C{^{1}H} NMR (125.7 MHz, 298.1 K, C_6D_6): δ = 169.9 ($C^{2/3}$), 147.9 (br, $C^{1/4}$), 80.3 (C_4B-$\underline{C}_5H_4$), 75.1 (C_4B-$\underline{C}_5H_4$), 71.2 (Fe-$\underline{C}_5H_5$), 28.8 ($C^{1/4}$-Si(C($\underline{C}H_3$)$_3$)(CH_3)$_2$), 20.6 ($C^{2/3}$-$\underline{C}H_3$), 19.6 ($C^{1/4}$-Si($\underline{C}$(CH_3)$_3$)(CH_3)$_2$), -0.8 ($C^{1/4}$-Si(C(CH_3)$_3$)($\underline{C}H_3$)$_2$).
The ^{13}C NMR signal of the *α*-carbon atom bonded to the boron atom could not be determined.

^{11}B{^{1}H} NMR (160.4 MHz, 298.1 K, C_6D_6): δ = 66.4 ($C_4\underline{B}$-Fc).

^{29}Si{^{1}H} INEPT NMR (99.3 MHz, 298.1 K, C_6D_6): δ = -3.7 ($C^{1/4}$-$\underline{Si}$(C(CH_3)$_3$)(CH_3)$_2$).

HR-MS (30 eV, EI): m/z = calc.: 504.2497 $C_{28}H_{45}BFeSi_2$
exp.: 504.2507

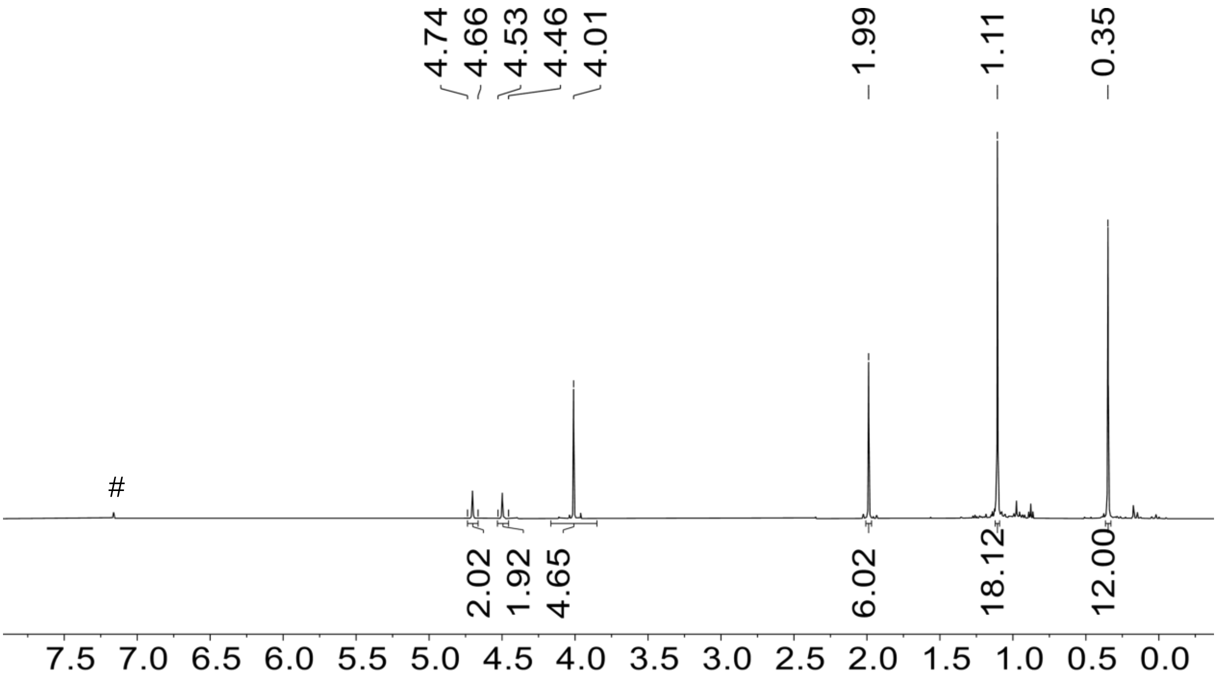

Figure S50. ^{1}H NMR spectrum (499.9 MHz, 298.1 K, C_6D_6) of Fc-substituted borole **25**, # = C_6D_5H.

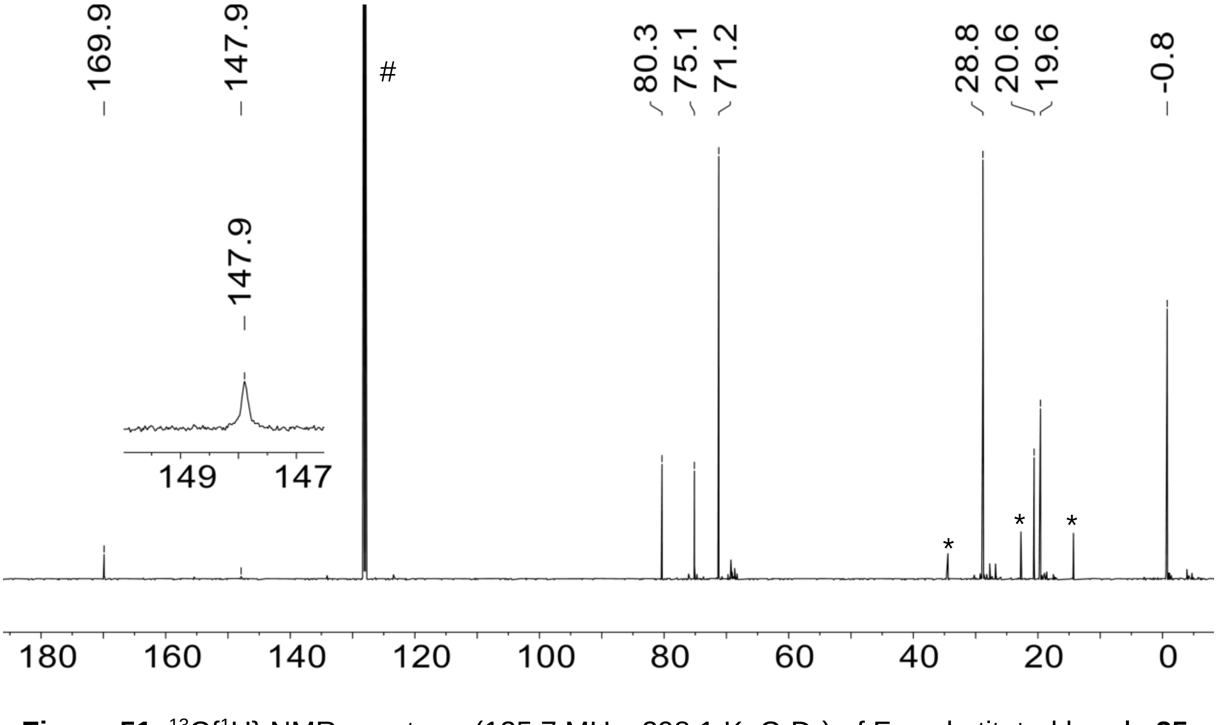

Figure 51. $^{13}C\{^{1}H\}$ NMR spectrum (125.7 MHz, 298.1 K, C_6D_6) of Fc-substituted borole **25**, * = *n*-pentane, # = C_6D_6.

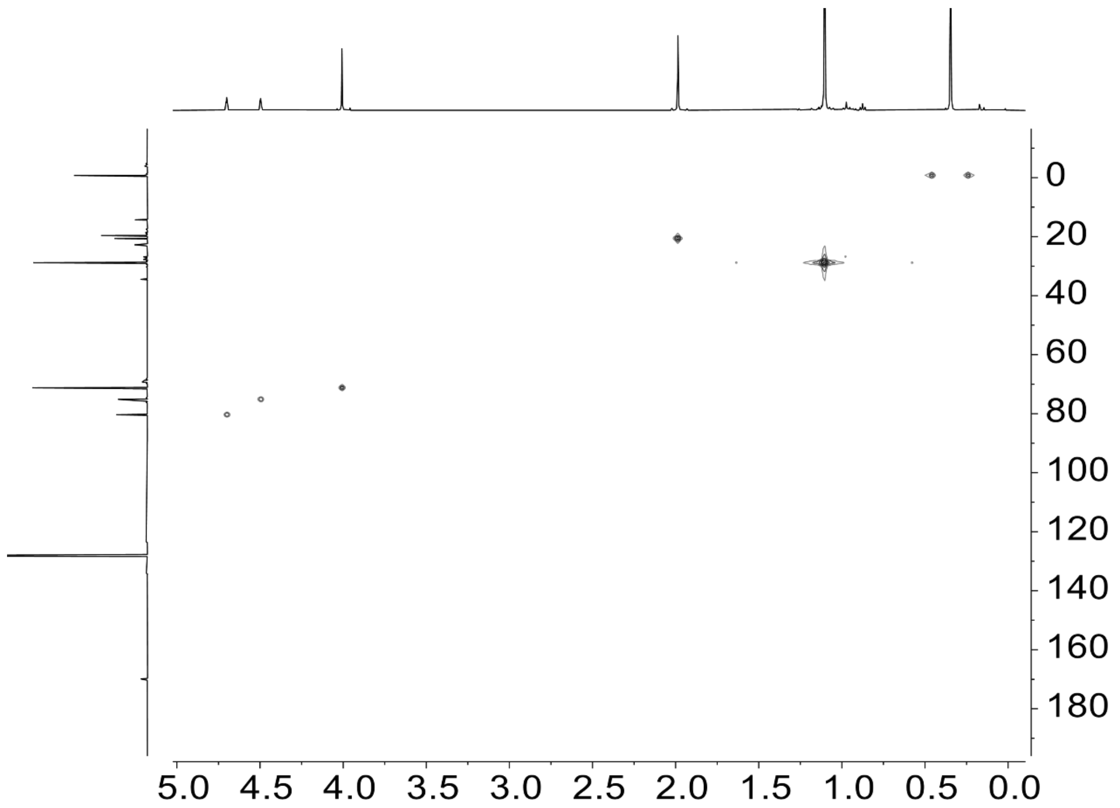

Figure 52. $^{1}H^{13}C$ HMQC NMR spectrum (499.9 MHz, 298.1 K, C_6D_6) of Fc-substituted borole **25**.

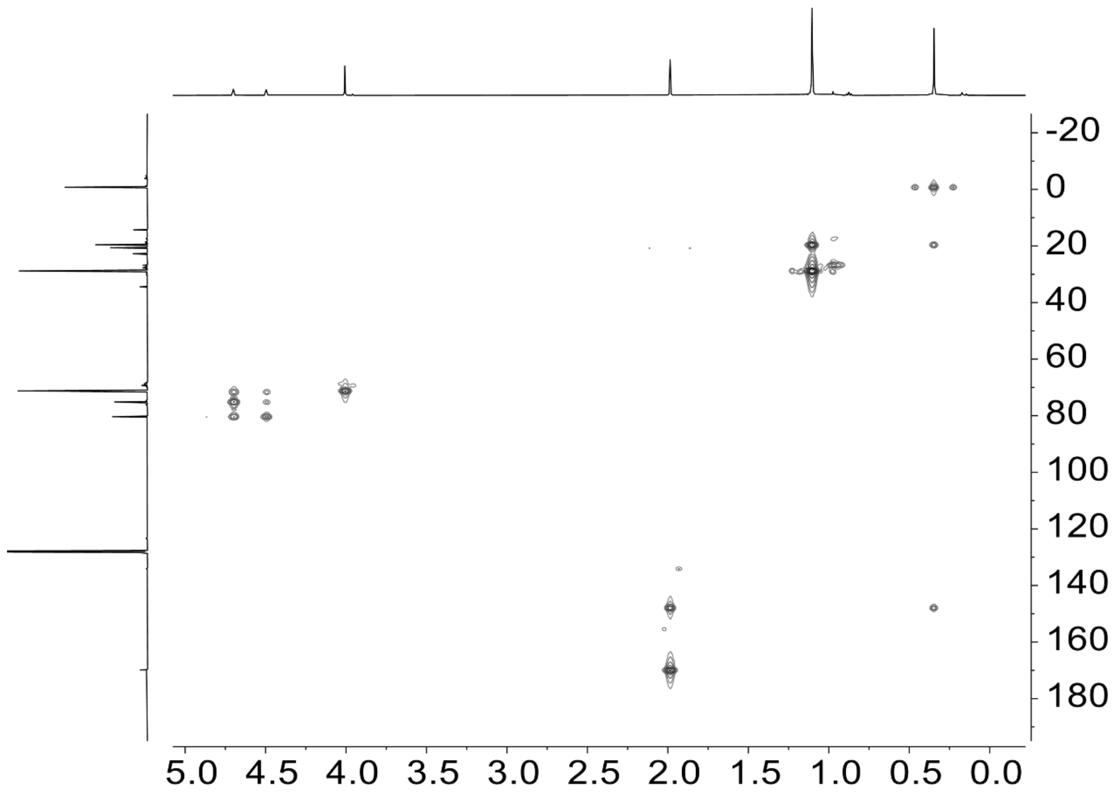

Figure 53. $^{1}H^{13}C$ HMBC NMR spectrum (499.9 MHz, 298.1 K, C_6D_6) of Fc-substituted borole **25**.

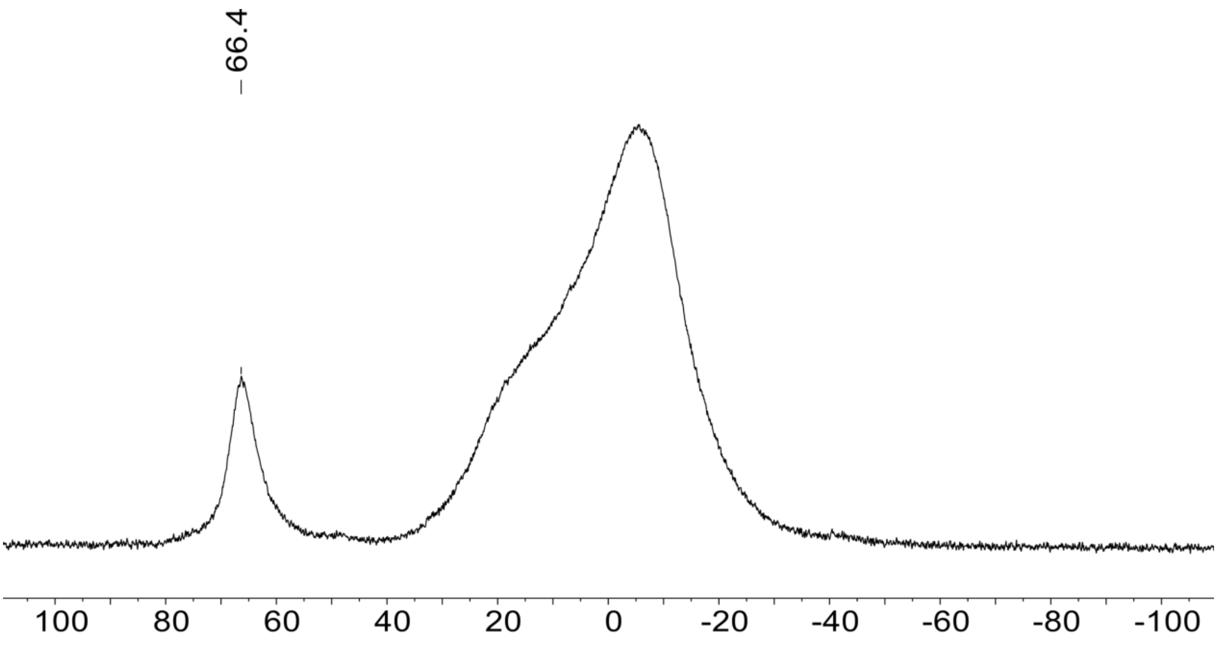

Figure S54. $^{11}B\{^{1}H\}$ NMR spectrum (160.4 MHz, 298.1 K, C_6D_6) of Fc-substituted borole **25**.

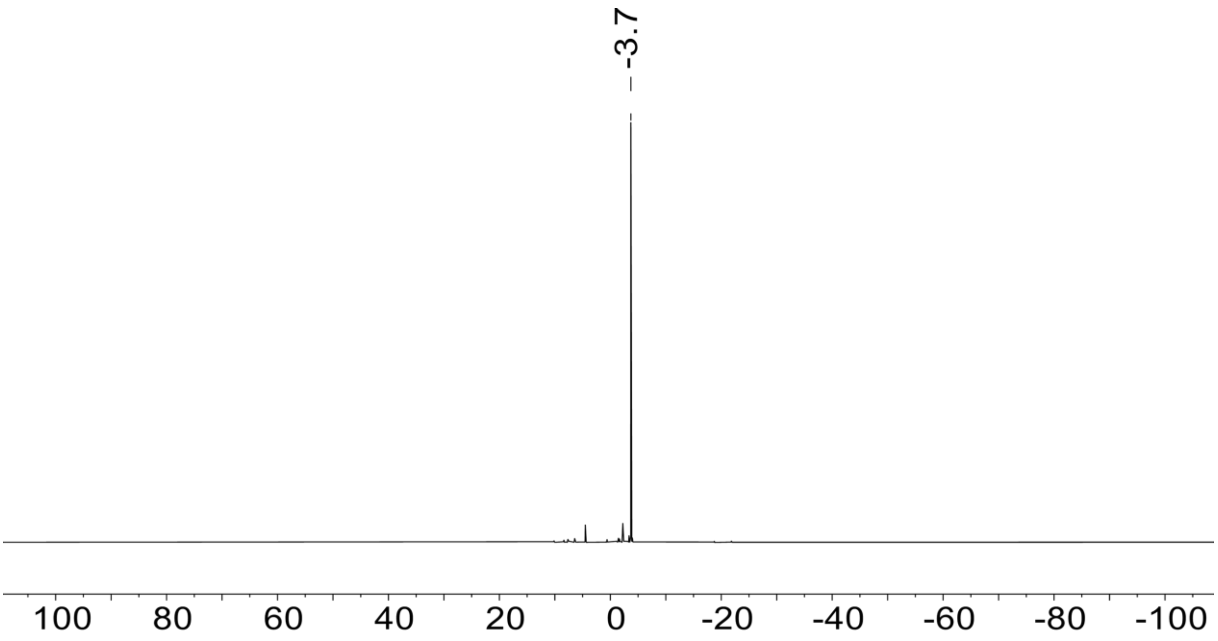

Figure S55. $^{29}Si\{^1H\}$ INEPT NMR spectrum (99.3 MHz, 298.1 K, C_6D_6) of Fc-substituted borole **25**.

Synthesis of tungsten complex 30

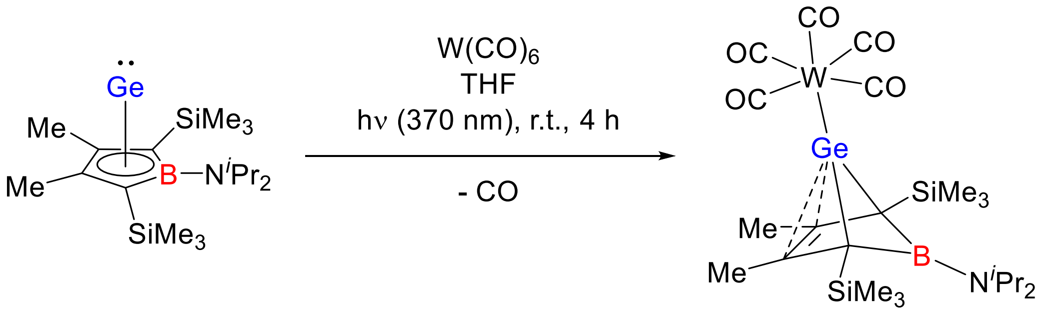

12e **30**

At room temperature, a mixture of boragerma[5]pyramidane **12e** (204 mg, 0.50 mmol, 1.00 equiv.) and tungsten hexacarbonyl (176 mg, 0.50 mmol, 1.00 equiv.) in THF (15 mL) was irradiated with a LED (370 nm) for 4 h. After that, the solvent was removed under reduced pressure. After crystallisation from *n*-pentane at T = -24 °C, the product **30** (293 mg, 0.40 mmol, 80%) was obtained as orange crystals suitable for sc-XRD analysis.

^{1}**H NMR** (499.9 MHz, 298.0 K, C_6D_6): δ = 3.97 (sept, $^3J_{H,H}$ = 7.1 Hz, 2H, N(C<u>H</u>(CH$_3$)$_2$)$_2$), 2.13 (s, 6 H, C$^{2/3}$-C<u>H</u>$_3$), 1.12 (d, $^3J_{H,H}$ = 7.1 Hz, 12 H, N(CH(C<u>H</u>$_3$)$_2$)$_2$), 0.24 (s, 18 H, C$^{1/4}$-Si(C<u>H</u>$_3$)).

^{13}**C{^{1}H} NMR** (125.7 MHz, 298.0 K, C_6D_6): δ = 196.6 (CO^{eq}, $^{1}J_{C,W}$ = 124.7 Hz), 196.5 (CO^{ax}, $^{1}J_{C,W}$ = 161.8 Hz), 132.7 (C$^{2/3}$), 75.1 (C$^{1/4}$), 50.2 (N(<u>C</u>H(CH$_3$)$_2$)$_2$), 25.7 (N-(CH(<u>C</u>H$_3$)$_2$)$_2$), 15.3 (C$^{2/3}$-<u>C</u>H$_3$), 2.3 (C$^{1/4}$-Si(<u>C</u>H$_3$)$_3$).

^{11}**B{^{1}H} NMR** (160.4 MHz, 298.0 K, C_6D_6): δ = 36.9 (C$_4$<u>B</u>-N^{i}Pr$_2$).

^{29}**Si{^{1}H} INEPT NMR** (99.3 MHz, 298.0 K, C_6D_6): δ = -8.2 (C$^{1/4}$-<u>Si</u>(CH$_3$)$_3$).

IR (ATR, neat): 2072, 1977, 1915 cm^{-1}.

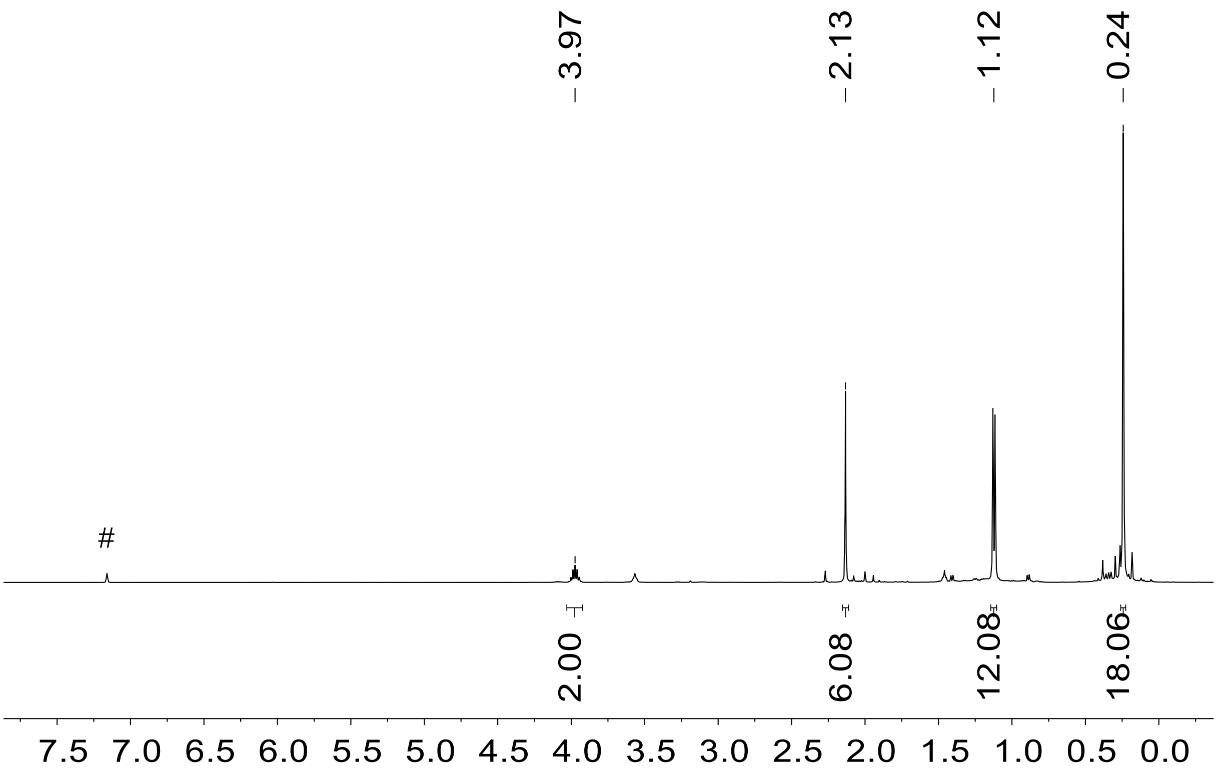

Figure S26. ^{1}H NMR spectrum (499.9 MHz, 298.0 K, C_6D_6) of tungsten complex **30**, # = C_6D_5H.

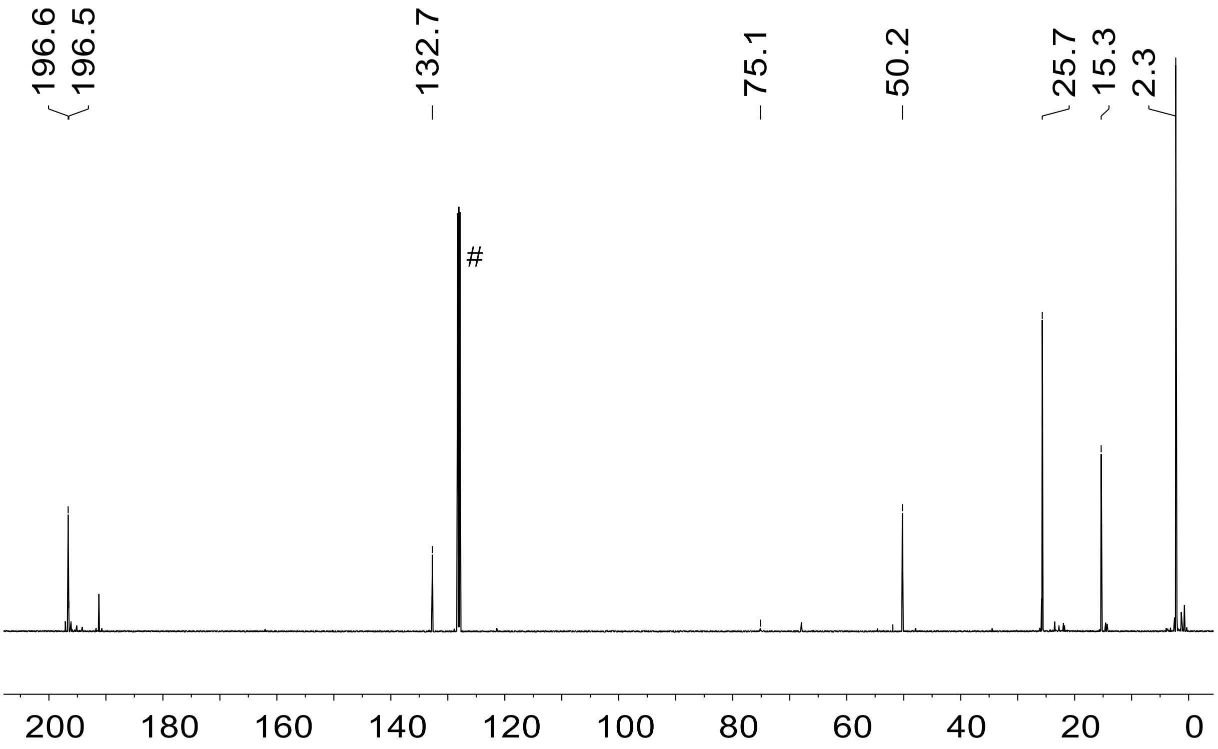

Figure S56. $^{13}C\{^{1}H\}$ NMR spectrum (125.7 MHz, 298.0 K, C_6D_6) of tungsten complex **30**, # = C_6D_6.

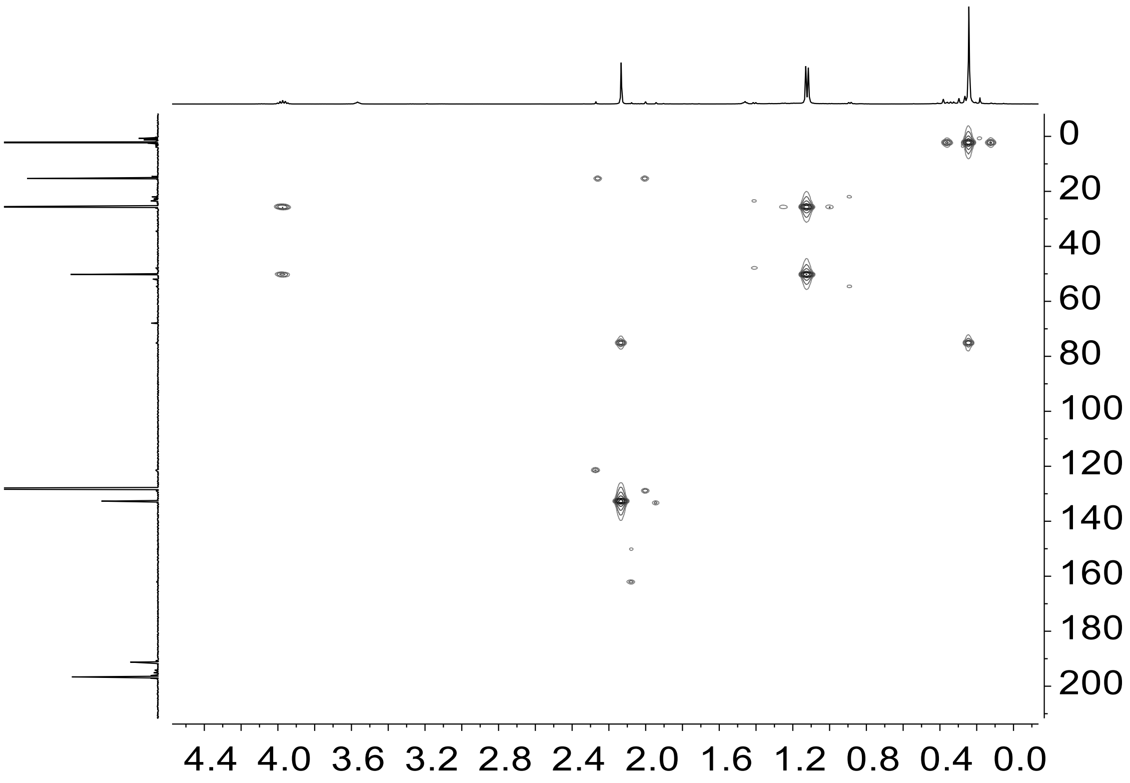

Figure S57. $^{1}H^{13}C$ HMBC NMR spectrum (499.9 MHz, 298.0 K, C_6D_6) of tungsten complex **30**.

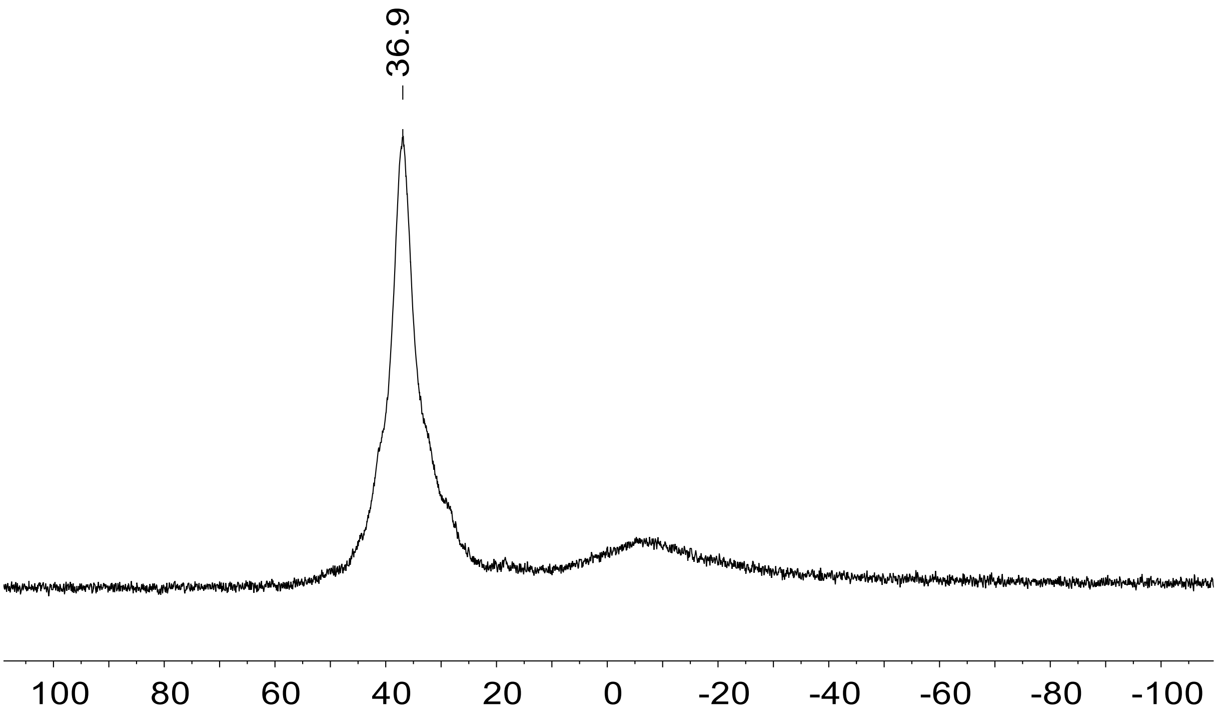

Figure S58. $^{11}B\{^1H\}$ NMR spectrum (160.4 MHz, 298.0 K, C_6D_6) of tungsten complex **30**.

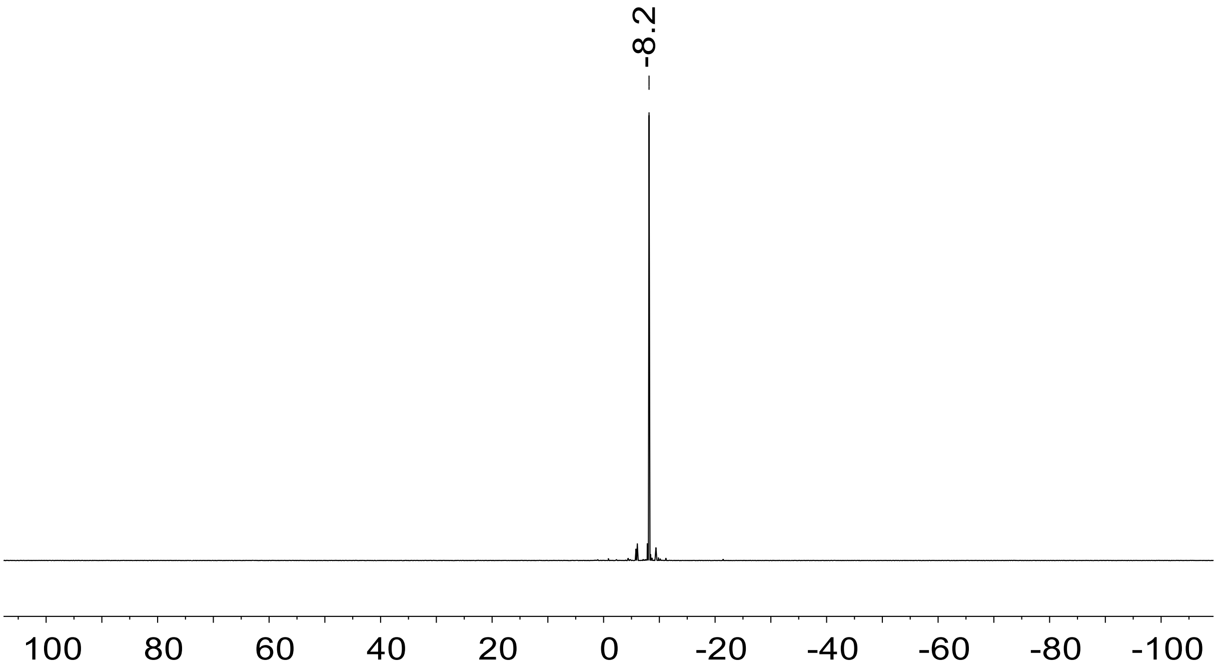

Figure 59. $^{29}Si\{^1H\}$ INEPT NMR spectrum (99.3 MHz, 298.0 K, C_6D_6) of tungsten complex **30**.

Synthesis of dilithium boracyclopentadienediide [Li(Et_2O)]$_2$[29]

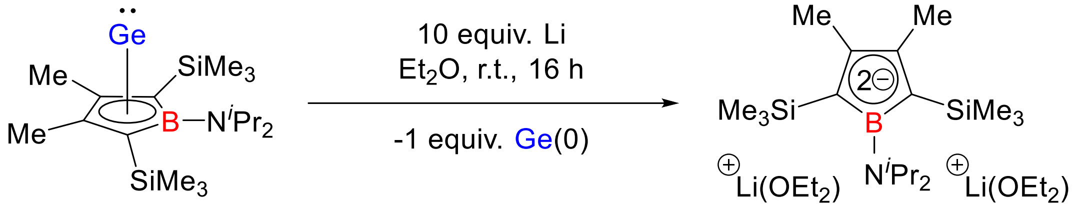

12e [Li(OEt_2)]$_2$[**29**]

Lithium (35 mg, 5.00 mmol, 10.00 equiv.) was added to a solution of boragerma[5]pyramidane **12e** (204 mg, 0.50 mmol, 1.00 equiv.) in Et_2O (10 mL). The reaction mixture was stirred for 16 h at room temperature. After that, the reaction mixture was filtered through a frit and the solvent was removed under reduced pressure. The product [Li(Et_2O)]$_2$[**29**] (197 mg, 0.39 mmol, 79%) was obtained as a green oily solid. Several attempts to crystallize compound [Li(Et_2O)]$_2$[**29**] did not succeed. Therefore, no satisfactory combustion analysis or HR-MS was obtained.

^{1}H NMR (499.9 MHz, 298.0 K, C_6D_6): δ = 3.72 (sept, $^3J_{H,H}$ = 6.3 Hz, 2H, $N(C\underline{H}(CH_3)_2)_2$), 3.04 (q, $^3J_{H,H}$ = 7.0 Hz, 8H, 2 x $O(C\underline{H}_2CH_3)_2$), 2.36 (s, 6H, $C^{2/3}$-$C\underline{H}_3$), 1.34 (d, $^3J_{H,H}$ = 6.4 Hz, 12H, $N(CH(C\underline{H}_3)_2)_2$), 0.81 (t, $^3J_{H,H}$ = 7.0 Hz, 12H, 2 x $O(CH_2C\underline{H}_3)_2$), 0.49 (s, 18H, $C^{1/4}$-$Si(C\underline{H}_3)_3$).

^{13}C{^{1}H} NMR (125.7 MHz, 298.0 K, C_6D_6): δ = 117.5 ($C^{2/3}$), 98.1 ($C^{1/4}$), 65.2 ($O(\underline{C}H_2CH_3)_2$), 48.9 ($N(\underline{C}H(CH_3)_2)_2$), 26.1 ($N(CH(\underline{C}H_3)_2)_2$), 16.6 ($C^{2/3}$-$\underline{C}H_3$), 14.5 ($O(CH_2\underline{C}H_3)_2$), 5.2 ($C^{1/4}$-$Si(\underline{C}H_3)_3$).

^{11}B{^{1}H} NMR (160.4 MHz, 298.0 K, C_6D_6): δ = 32.4 ($C_4\underline{B}$-N^iPr_2).

^{29}Si{^{1}H} INEPT NMR (99.3 MHz, 298.0 K, C_6D_6): δ = -14.7 ($C^{1/4}$-$\underline{Si}(CH_3)_3$).

^{7}Li{^{1}H} NMR (194.3 MHz, 298.0 K, C_6D_6): δ = -6.3 ($((H_3CH_2C)_2O)\underline{Li}$).

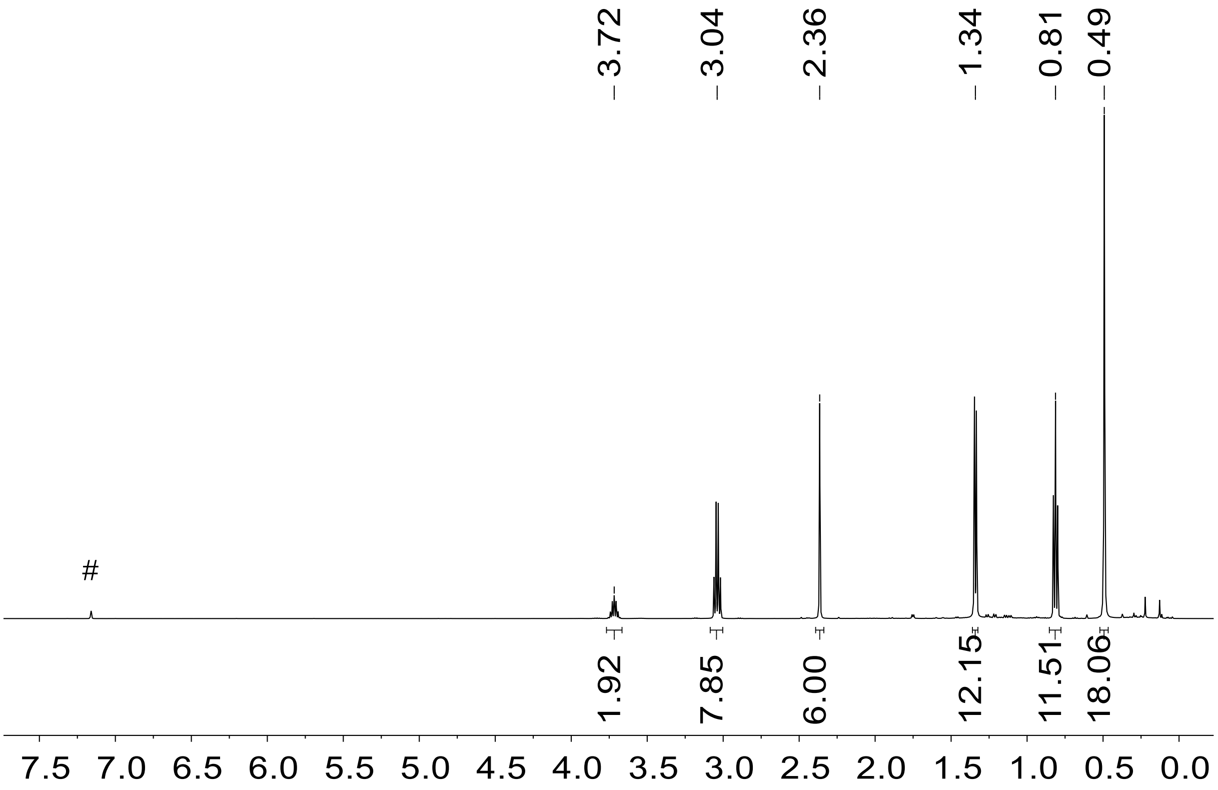

Figure S60. ^{1}H NMR spectrum (499.9 MHz, 298.0 K, C_6D_6) of dilithio boracyclopentadienediide $[Li(Et_2O)]_2$[**29**], # = C_6D_5H.

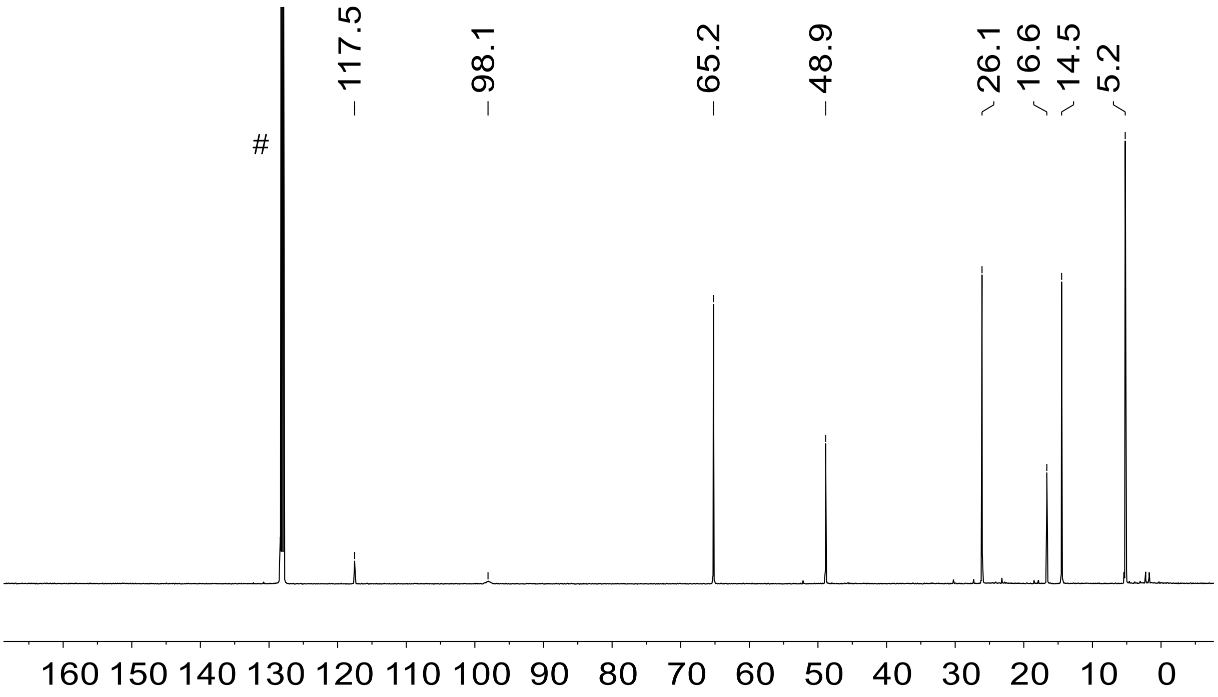

Figure S61. $^{13}C\{^{1}H\}$ NMR spectrum (125.7 MHz, 298.0 K, C_6D_6) of dilithio boracyclopentadienediide $[Li(Et_2O)]_2$[**29**], # = C_6D_6.

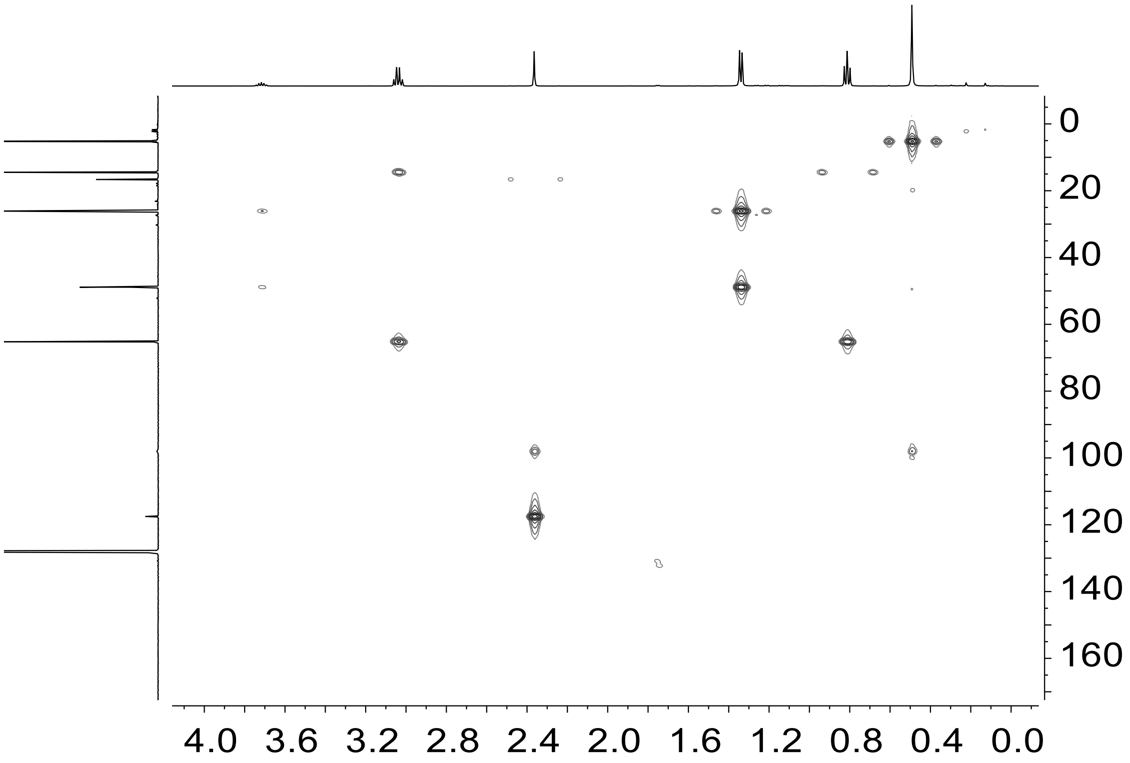

Figure S62. $^{1}H^{13}C$ HMBC NMR spectrum (499.9 MHz, 298.0 K, C_6D_6) of dilithio boracyclopentadienediide $[Li(Et_2O)]_2$[**29**].

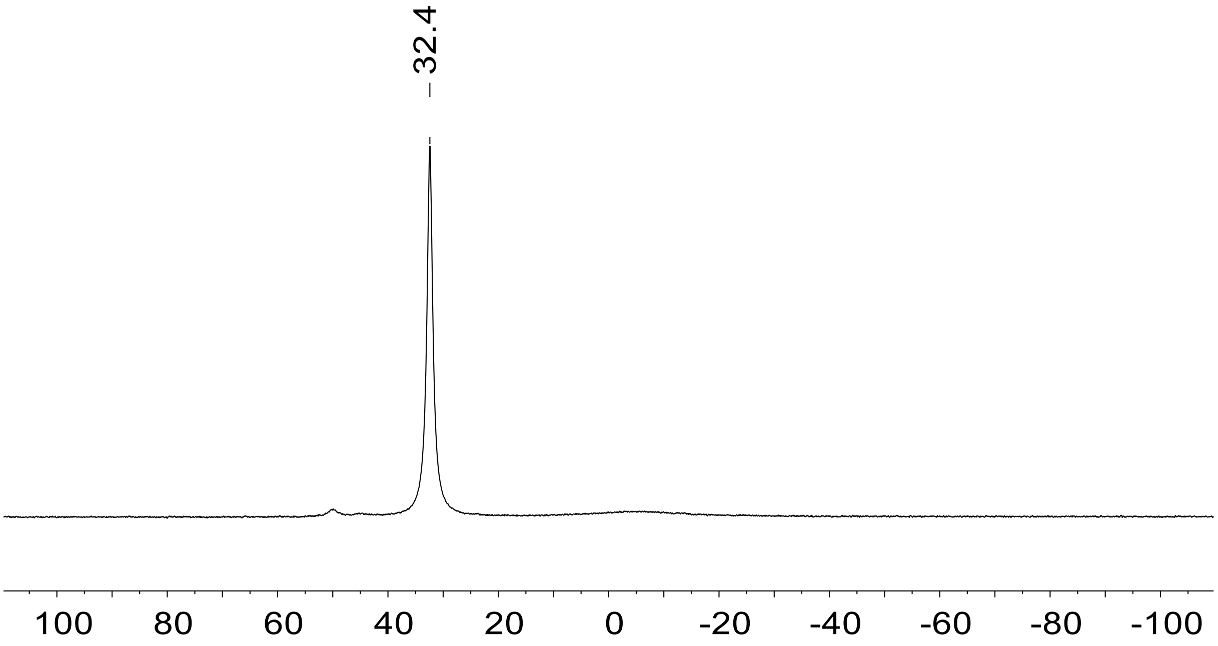

Figure S63. $^{11}B\{^{1}H\}$ NMR spectrum (160.4 MHz, 298.0 K, C_6D_6) of dilithio boracyclopentadienediide $[Li(Et_2O)]_2$[**29**].

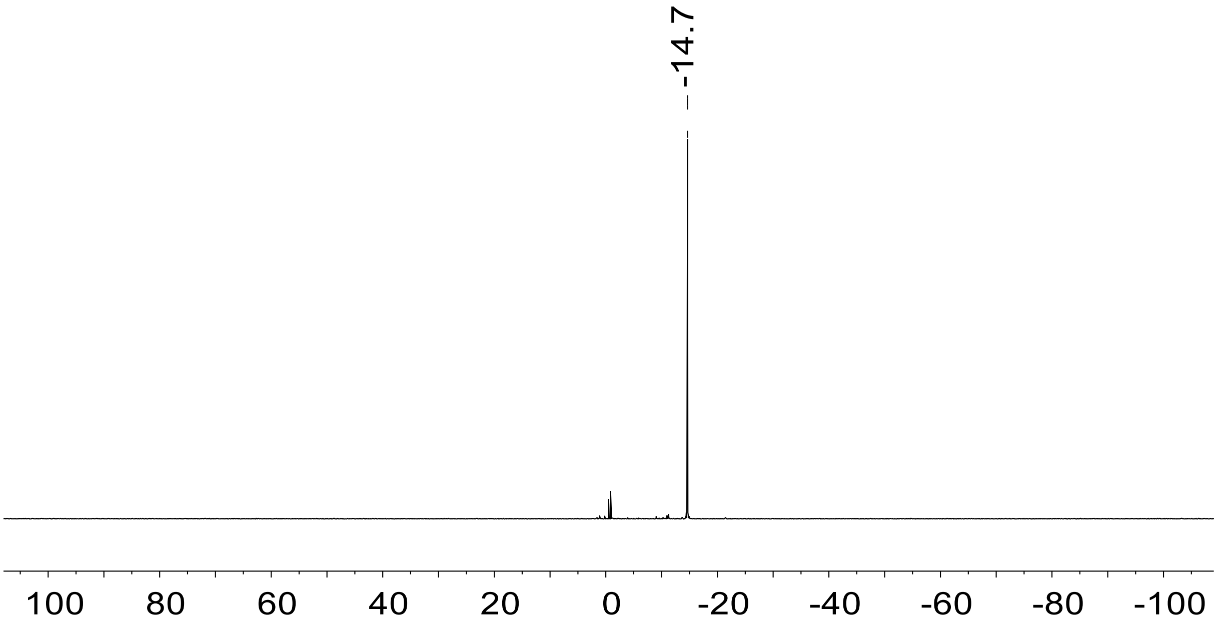

Figure S64. $^{29}Si\{^1H\}$ INEPT NMR spectrum (99.3 MHz, 298.0 K, C_6D_6) of dilithio boracyclopentadienediide $[Li(Et_2O)]_2$[**29**].

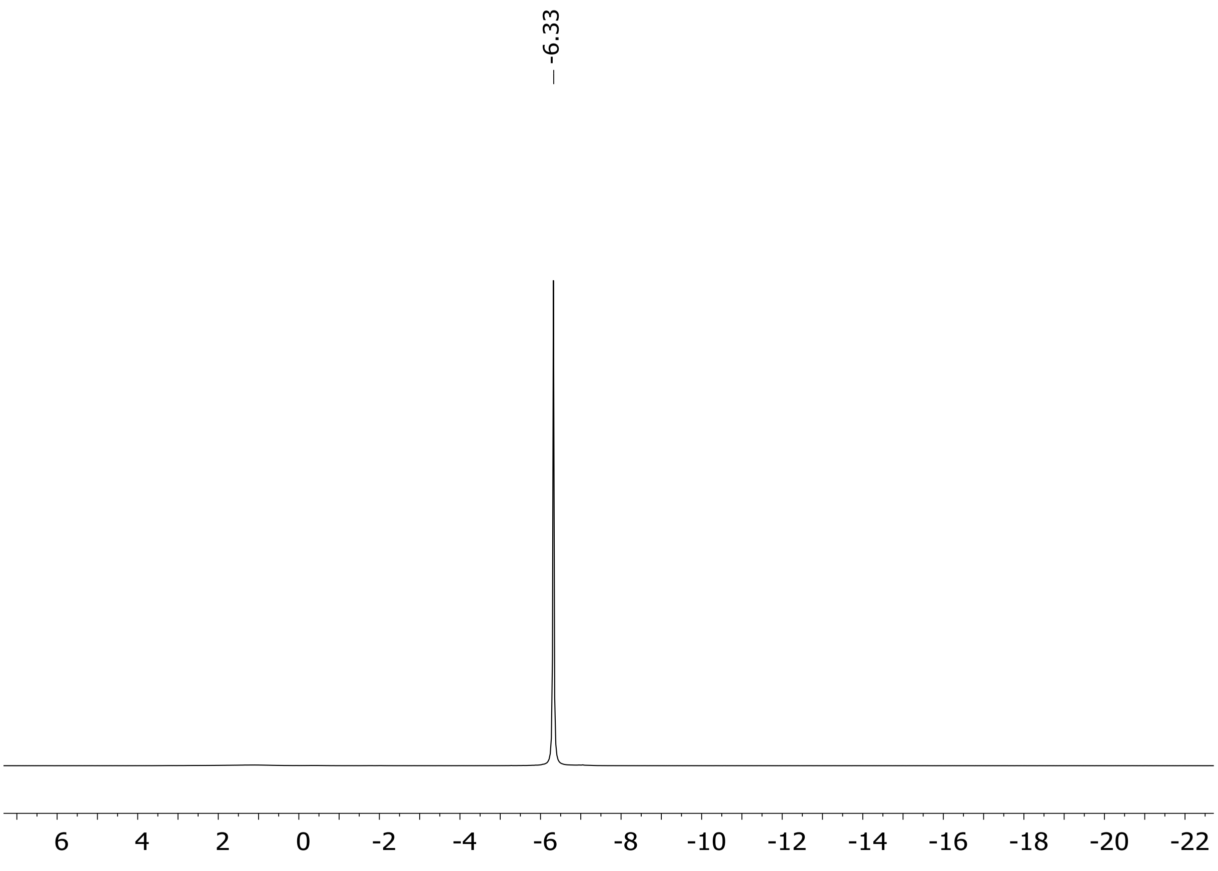

Figure S65. $^7Li\{^1H\}$ NMR spectrum (194.3 MHz, 298.0 K, C_6D_6) of dilithio boracyclopentadienediide $[Li(Et_2O)]_2$[**29**].

Synthesis of dilithium boracyclopentadienediide [Li(THF)]$_2$[29]

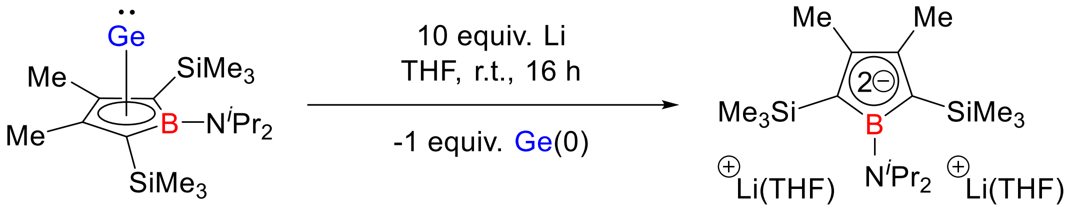

12e [Li(THF)]$_2$[**29**]

Lithium (35 mg, 5.00 mmol, 10.00 equiv.) was added to a solution of boragerma[5]pyramidane **12e** (204 mg, 0.50 mmol, 1.00 equiv.) in THF (10 mL). The reaction mixture was stirred for 16 h at room temperature. After that, the reaction mixture was filtered through a frit and the solvent was removed under reduced pressure. The product [Li(THF)]$_2$[**29**] (183 mg, 0.37 mmol, 74%) was obtained as a green oily solid. Several attempts to crystallize compound [Li(THF)]$_2$[**29**] did not succeed.

^{1}H NMR (499.9 MHz, 298.0 K, C_6D_6): δ = 3.79 (sept, $^3J_{H,H}$ = 6.3 Hz, 2H, N(C$\underline{H}$(CH$_3$)$_2$)$_2$), 3.30-3.19 (m, 8H, 2 x C$_4\underline{H}_8$O), 2.45 (s, 6H, C$^{2/3}$-C$\underline{H}_3$), 1.38 (d, $^3J_{H,H}$ = 6.4 Hz, 12H, N(CH(C$\underline{H}_3$)$_2$)$_2$), 1.18-1.13 (m, 8H, 2 x C$_4\underline{H}_8$O), 0.55 (s, 18H, C$^{1/4}$-Si(C$\underline{H}_3$)$_3$).

^{13}C{^{1}H} NMR (125.7 MHz, 298.0 K, C_6D_6): δ = 117.8 (C$^{2/3}$), 98.8 (C$^{1/4}$), 68.7 ($\underline{C}_4$H$_8$O), 48.3 (N($\underline{C}$H(CH$_3$)$_2$)$_2$), 25.9 (N(CH($\underline{C}$H$_3$)$_2$)$_2$), 25.2 ($\underline{C}_4$H$_8$O), 16.4 (C$^{2/3}$-$\underline{C}$H$_3$), 5.0 (C$^{1/4}$-Si($\underline{C}$H$_3$)$_3$).

^{11}B{^{1}H} NMR (160.4 MHz, 298.0 K, C_6D_6): δ = 31.5 (C$_4\underline{B}$-N^{i}Pr$_2$).

^{29}Si{^{1}H} INEPT NMR (99.3 MHz, 298.0 K, C_6D_6): δ = -14.3 (C$^{1/4}$-$\underline{Si}$(CH$_3$)$_3$).

^{7}Li{^{1}H} NMR (194.3 MHz, 298.0 K, C_6D_6): δ = -6.2 ((C$_4$H$_8$O)$\underline{Li}$).

**HR-MS** (30 eV, EI):	m/z =	calc.:	493.4106	$C_{26}H_{54}BLi_2NO_2Si_2$
		exp.:	493.4104	

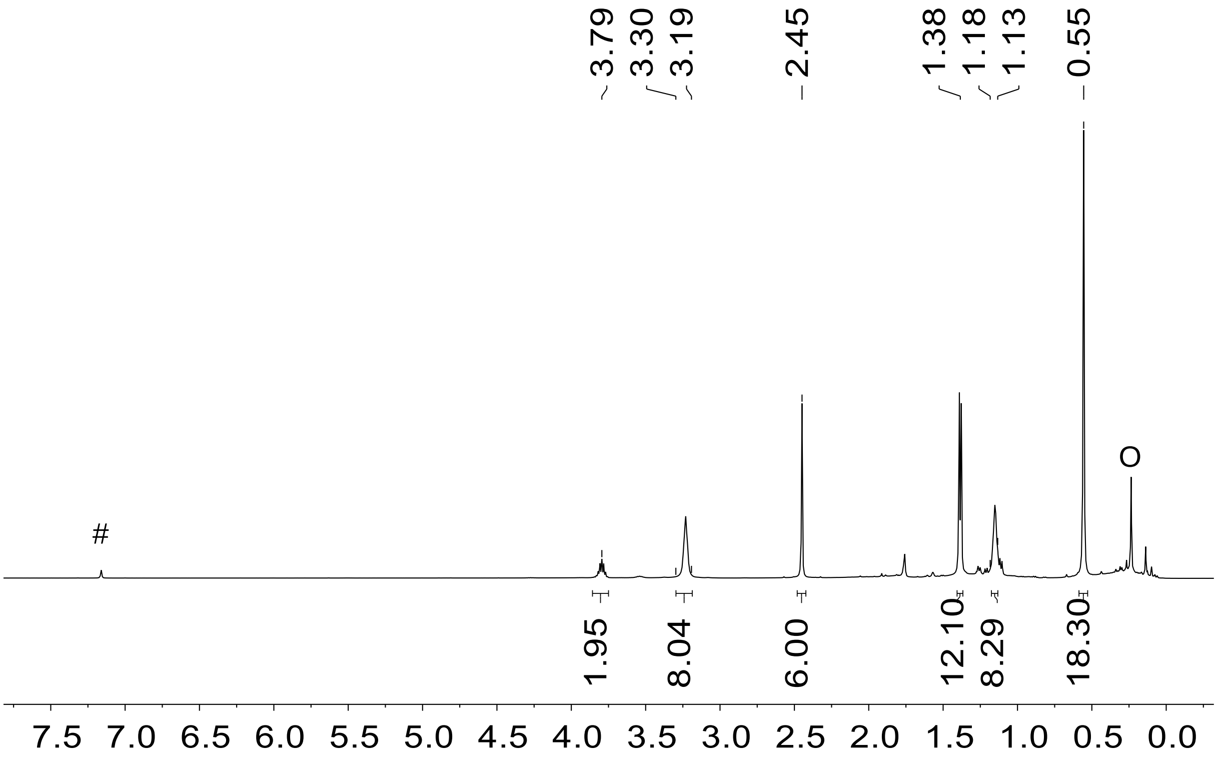

Figure S66. ^{1}H NMR spectrum (499.9 MHz, 298.0 K, C_6D_6) of dilithio boracyclopenta-dienediide [Li(THF)]$_2$[**29**], # = C_6D_5H, O = unidentified impurity.

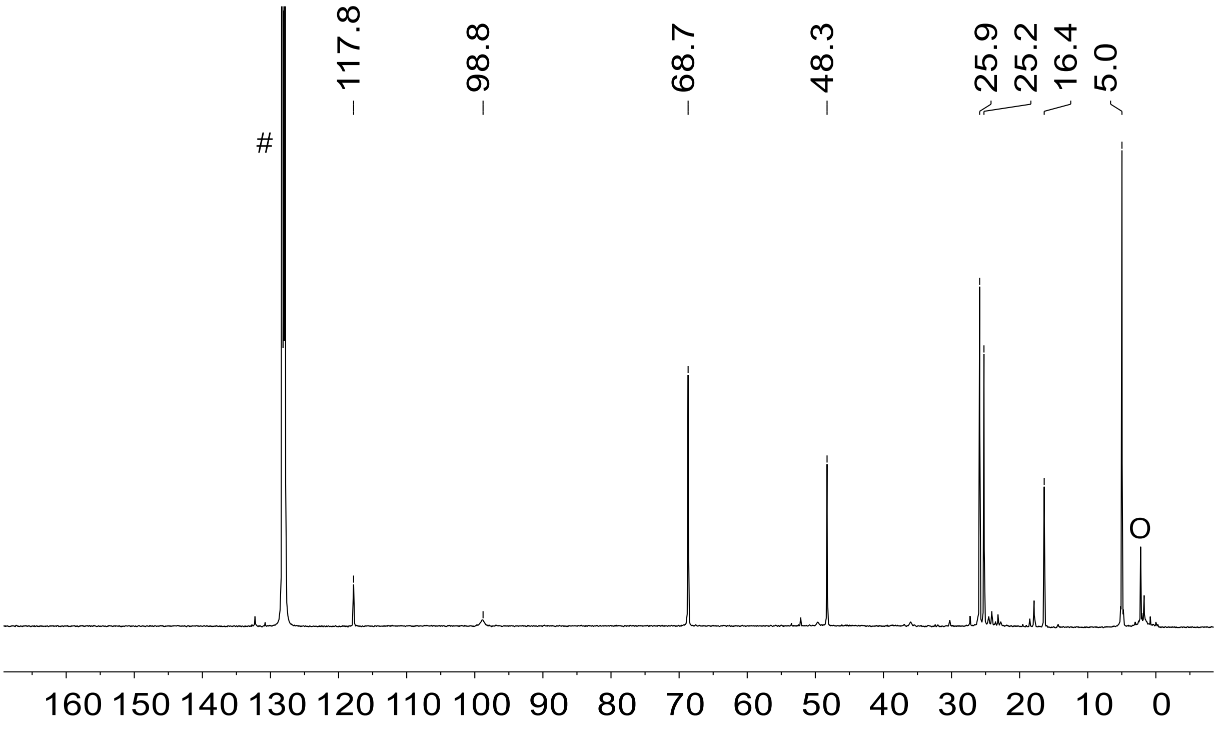

Figure S67. ^{13}C{^{1}H} NMR spectrum (125.7 MHz, 298.0 K, C_6D_6) of dilithio boracyclopenta-dienediide [Li(THF)]$_2$[**29**], # = C_6D_6, O = unidentified impurity.

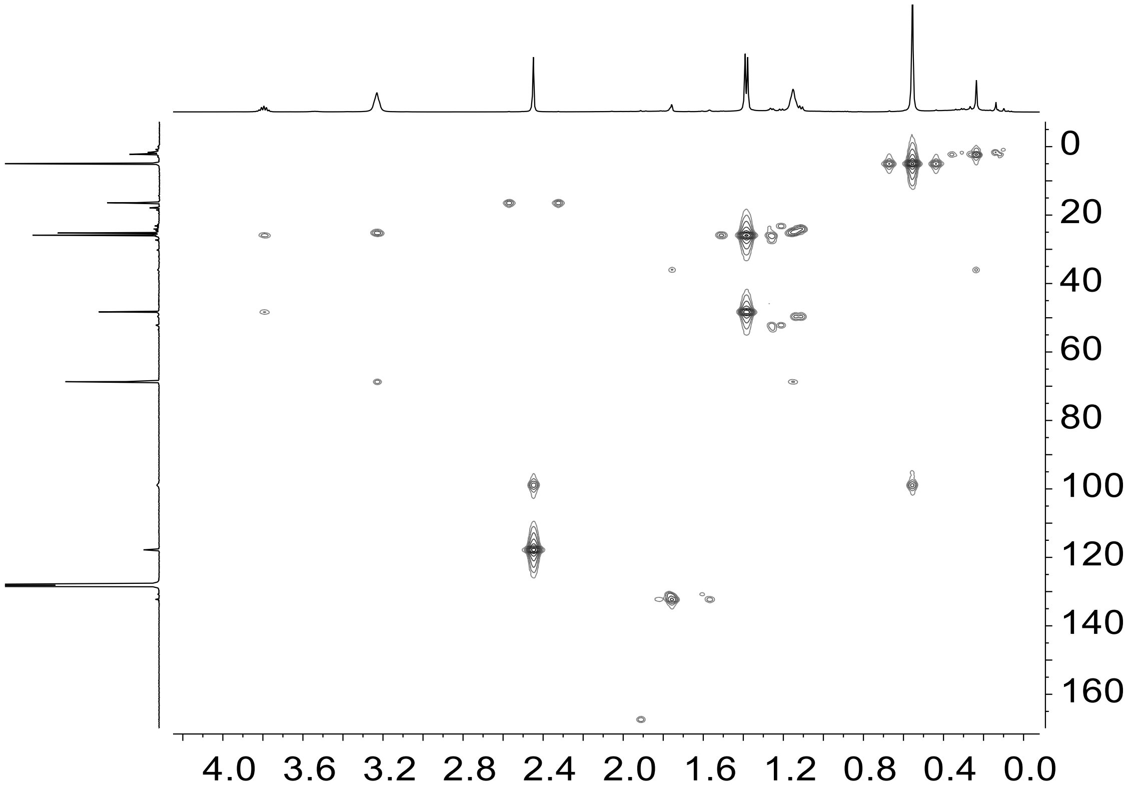

Figure S27. $^{1}H^{13}C$ HMBC NMR spectrum (499.9 MHz, 298.0 K, C_6D_6) of dilithio boracyclopentadienediide [Li(THF)]$_2$[**29**].

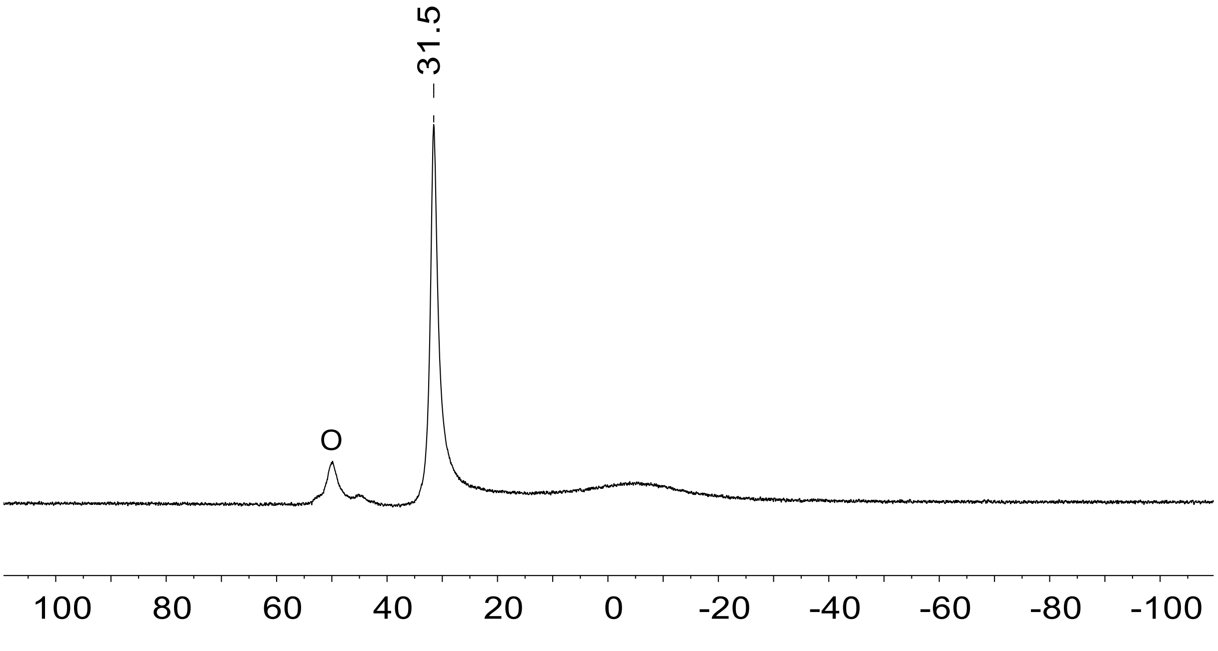

Figure S69. $^{11}B\{^{1}H\}$ NMR spectrum (160.4 MHz, 298.0 K, C_6D_6) of dilithio boracyclopentadienediide [Li(THF)]$_2$[**29**], O = unidentified impurity.

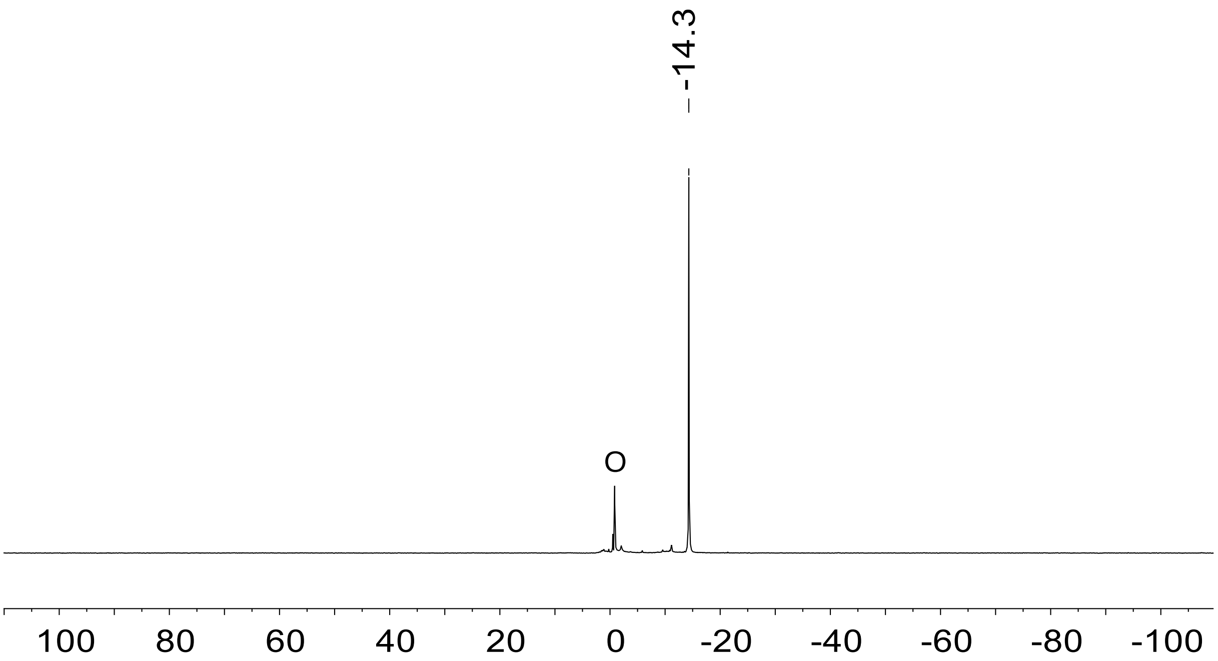

Figure S70. $^{29}Si\{^{1}H\}$ INEPT NMR spectrum (99.3 MHz, 298.0 K, C_6D_6) of dilithio boracyclopentadienediide $[Li(THF)]_2$[**29**], O = unidentified impurity.

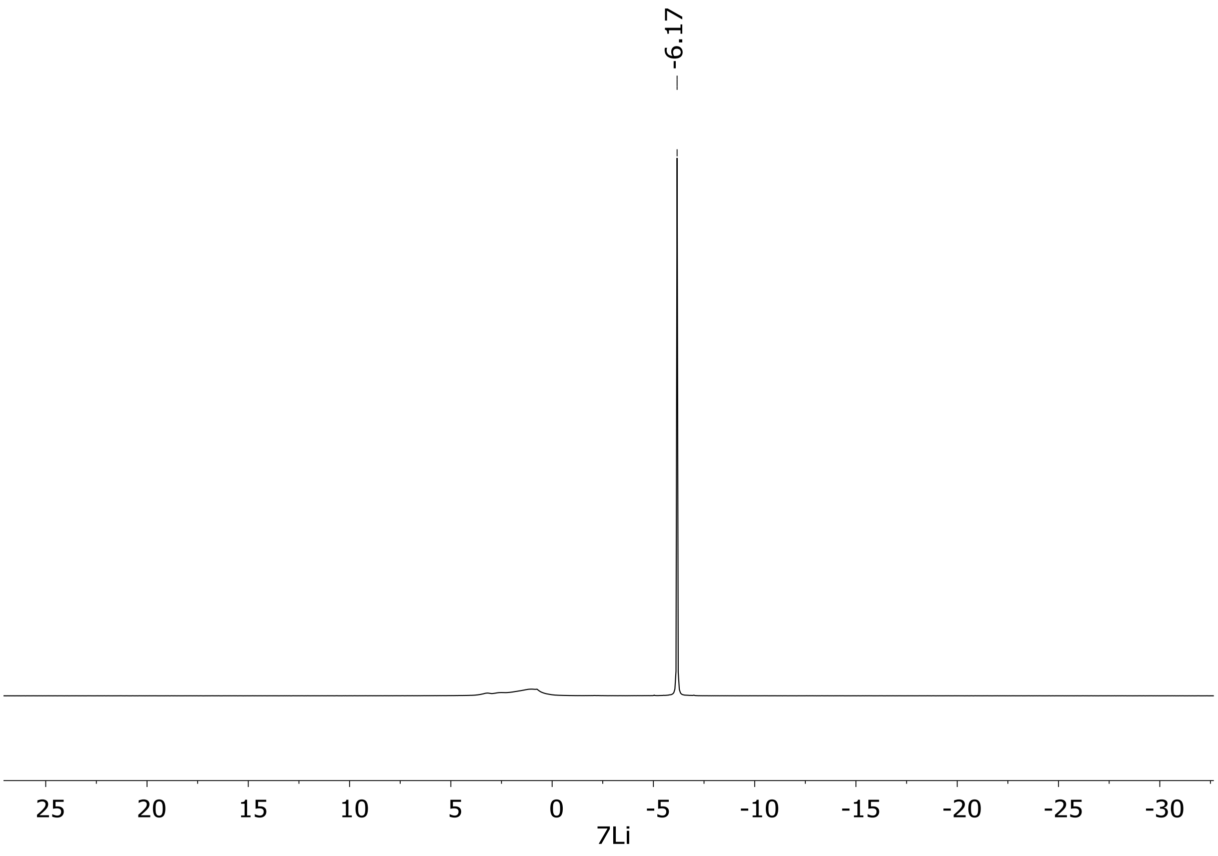

Figure S71. $^{7}Li\{^{1}H\}$ NMR spectrum (194.3 MHz, 298.0 K, C_6D_6) of dilithio boracyclopenta-dienediide $[Li(THF)]_2$[**29**].

Table S1. Characteristic NMR parameters of newly synthesized compounds in comparison with related compounds. NMR chemical shifts computed for optimized molecular structures are given in parentheses (M06-L/6-311G(2d,p)//M06-2X/6-311+G(d,p)).

**Verbindung**	**$\delta^{13}C(C^{1/4})$**	**$\delta^{13}C(C^{2/3})$**	**$\delta^{11}B(C_4B)$**	others
**12d**	107.0 (*98*)	132.2 (*125*)	29.4 (*25*)	
**12e**	89.3 (*69*)	128.3 (*122*)	34.3 (*34*)	
**12f**	100.4 (*86*)	128.6 (*122*)	32.1 (*28*)	
**12g**	101.7 (*96*)	133.5 (*127*)	32.5 (*26*)	
**14a**	101.4, 100.7	136.0, 135.4	35.6	
**14d**	104.8	133.8	30.6	
**14e**	89.8	129.1	33.8	
**14f**	102.4	129.6	32.1	
**14g**	101.1	134.6	32.4	
**20f**	136.8	88.7	35.0	$\delta^{29}Si(Si(II))$ = -337.5
**25**	147.9	169.9	66.4	
**30**	75.1	132.7	36.9	
$[Li(OEt_2)]_2$[**29**]	98.1	117.5	32.4	$\delta^{7}Li$ = -6.3
$[Li(THF)]_2$[**29**]	98.8	117.8	31.5	$\delta^{7}Li$ = -6.2
K_2[**11**][1]	156.1	130.9	-	
**12a**[11]	99.8, 99.3	134.8, 133.6	37.2	
**12b**[12]	103.2	127.7	30.7	
**12c**[12]	105.5	129.3	29.5	
**4**[13]	113.2	141.8	30.2	
**5**[13]	106.6	138.8	29.2	
$[Li(OEt_2)]_2$[**18**]	99.5	131.7	32	$\delta^{7}Li$ = -6.9
Li_2[**1**][14]	82.8	98.9	27.0	

Crystallographic Data.

Molecular structure of Cp*-substituted boragerma[5]pyramidane 14a from sc-XRD

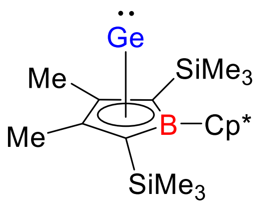

14a

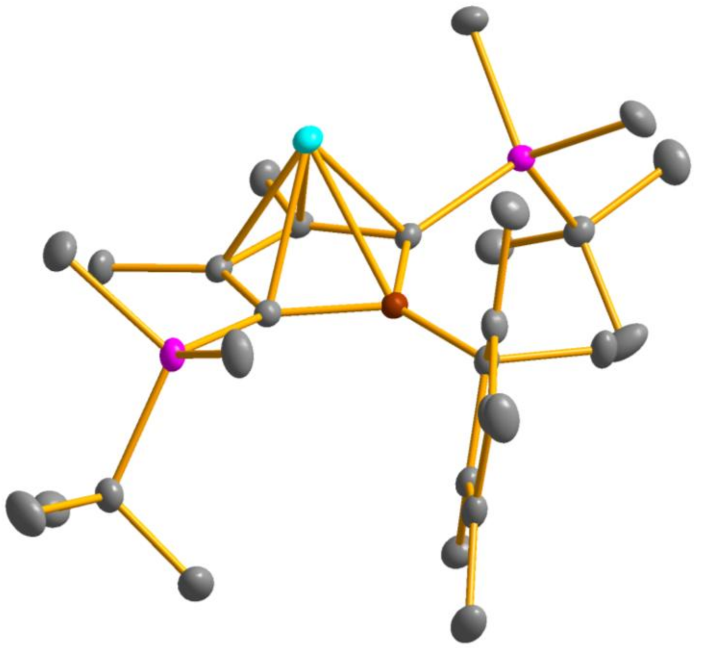

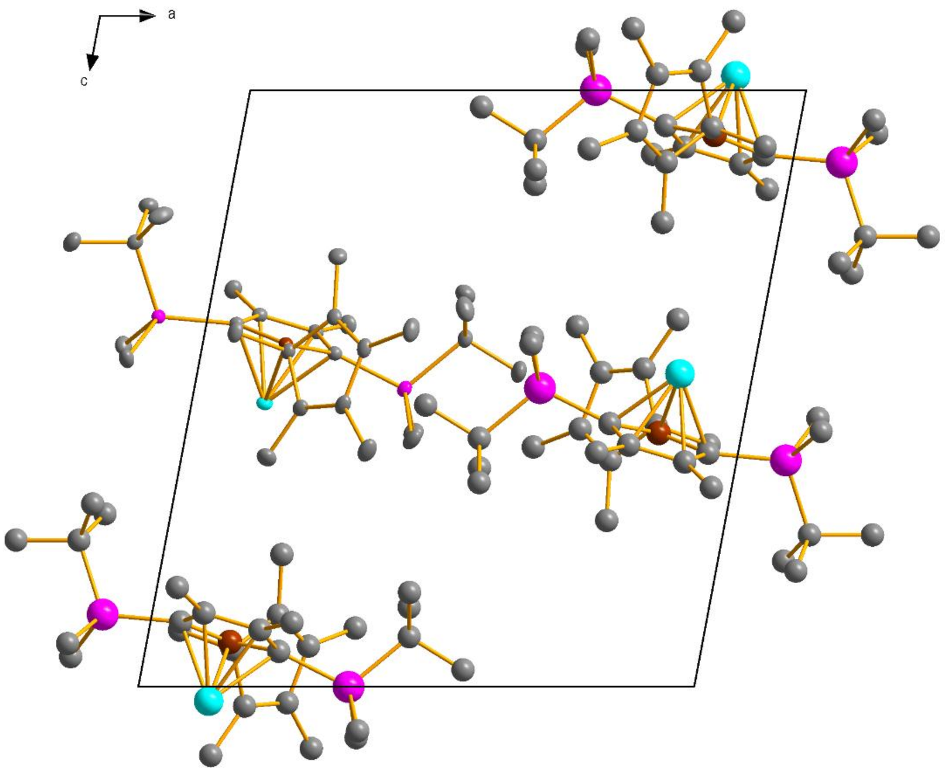

Unit cell (space group: $P2_1/c$, view along the b-axis, H-atoms omitted for clarity):

Table S2. Summary of crystallographic data of Cp*--substituted boragerma[5]pyramidane **14a** (CCSD 2520808).

Empirical formula	C28 H51 B Ge Si2	
Formula weight	527.26	
Temperature	100(2) K	
Wavelength	0.71073 Å	
Crystal system	Monoclinic	
Space group	$P2_1/c$	
Unit cell dimensions	a = 13.5064(5) Å	α = 90°.
	b = 15.1191(5) Å	β = 100.6251(13)°.
	c = 14.7398(5) Å	γ = 90°.
Volume	2958.33(18) $Å^3$	
Z	4	
Density (calculated)	1.184 Mg/m^3	
Absorption coefficient	1.130 mm^{-1}	
F(000)	1136	
Crystal size	0.140 x 0.070 x 0.020 mm^3	
Theta range for data collection	1.534 to 30.033°	
Index ranges	-19<=h<=19, -21<=k<=21, -20<=l<=16	
Reflections collected	105949	
Independent reflections	8665 (R(int) = 0.0482)	
Observed reflections (I > 2(I))	7660	
Completeness to theta = 30.033°	100.0 %	
Absorption correction	Semi-empirical from equivalents	
Max. and min. transmission	1.0000 and 0.9158	
Refinement method	Full-matrix least-squares on F^2	
Data / restraints / parameters	8665 / 0 / 306	
Goodness-of-fit on F^2	1.049	
Final R indices (I>2sigma(I))	R1 = 0.0301, wR2 = 0.0689	
R indices (all data)	R1 = 0.0369, wR2 = 0.0715	
Extinction coefficient	n/a	
Largest diff. peak and hole	0.453 and -0.323 e.$Å^{-3}$	

Molecular structure of N^{i}Pr$_2$-substituted boragerma[5]pyramidane 12e from sc-XRD

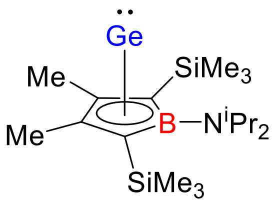

12e

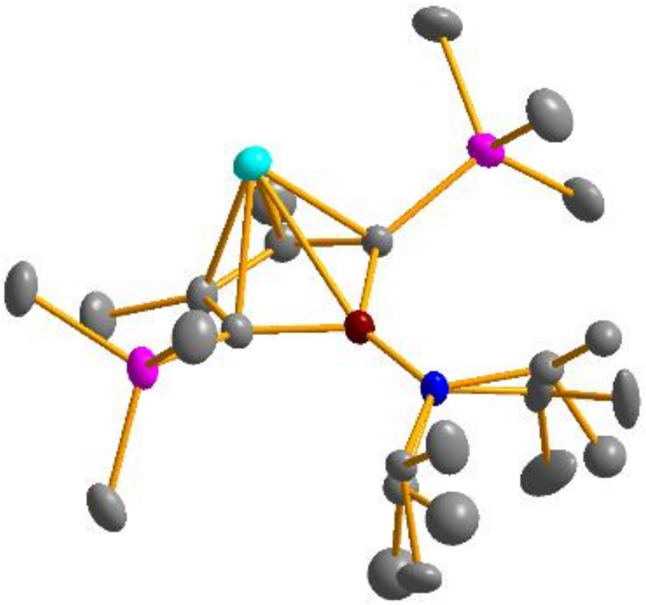

Disordered by 18%.

Unit cell (space group: Pna2$_1$, view along the b-axis, H-atoms omitted for clarity):

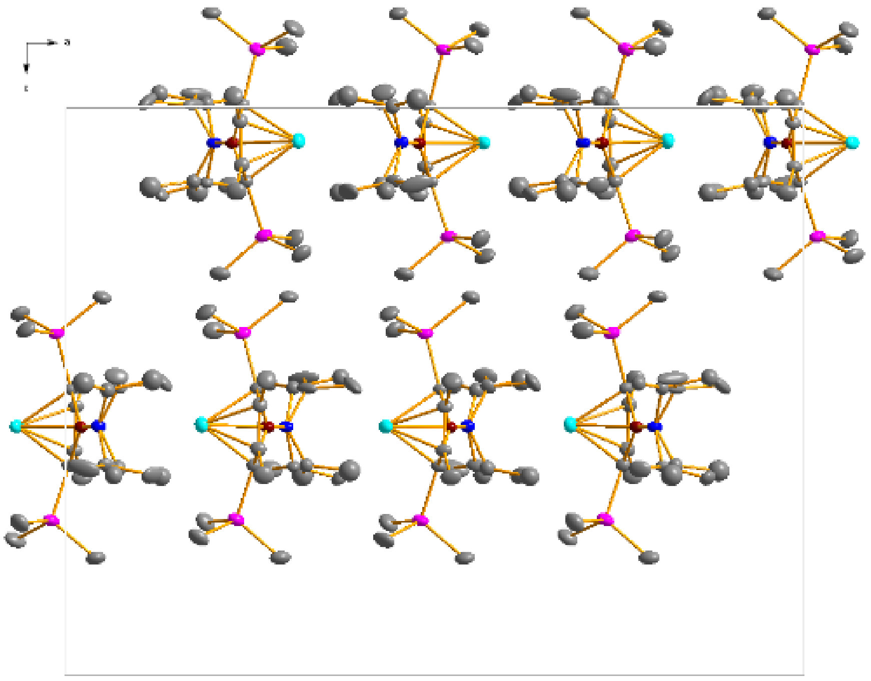

Table S3. Summary of crystallographic data of N^{i}Pr$_2$-substituted boragerma[5]pyramidane **12e** (CCSD 2520813).

Empirical formula	$C_{18}H_{38}BGeNSi_2$	
Formula weight	408.07	
Temperature	100(2) K	
Wavelength	0.71073 Å	
Crystal system	Orthorhombic	
Space group	$Pna2_1$	
Unit cell dimensions	a = 23.7185(8) Å	α = 90°.
	b = 10.5386(3) Å	β = 90°.
	c = 18.2546(6) Å	γ = 90°.
Volume	4562.9(3) $Å^3$	
Z	8	
Density (calculated)	1.188 Mg/m^3	
Absorption coefficient	1.448 mm^{-1}	
F(000)	1744	
Crystal size	0.170 x 0.140 x 0.130 mm^3	
Θ range for data collection	1.717 to 36.318°	
Index ranges	-39<=h<=39, -17<=k<=17, -30<=l<=30	
Reflections collected	348725	
Independent reflections	22129 (R(int) = 0.0544)	
Observed reflections (I > 2(I))	19142	
Completeness to Θ = 36.318°	100.0 %	
Absorption correction	Semi-empirical from equivalents	
Max. and min. transmission	1.0000 and 0.8916	
Refinement method	Full-matrix least-squares on F^2	
Data / restraints / parameters	22129 / 1 / 489	
Goodness-of-fit on F^2	1.063	
Final R indices (I>2σ(I))	R_1 = 0.0378, wR_2 = 0.1008	
R indices (all data)	R_1 = 0.0460, wR_2 = 0.1048	
Absolute structure parameter	0.381(8)	
Extinction coefficient	n/a	
Largest diff. peak and hole	0.950 and -0.921 e.$Å^{-3}$	

Molecular structure of N^{i}Pr$_2$-substituted boragerma[5]pyramidane 14e from sc-XRD

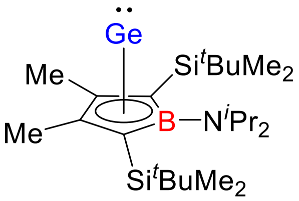

14e

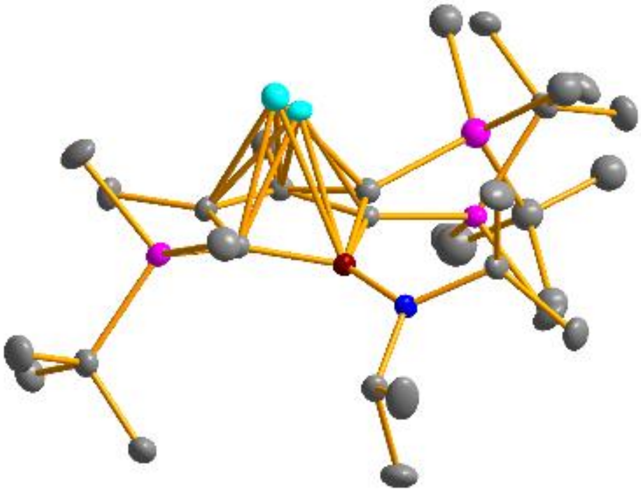

Disordered by 3%.

Unit cell (space group: C2/c, view along the b-axis, H-atoms omitted for clarity):

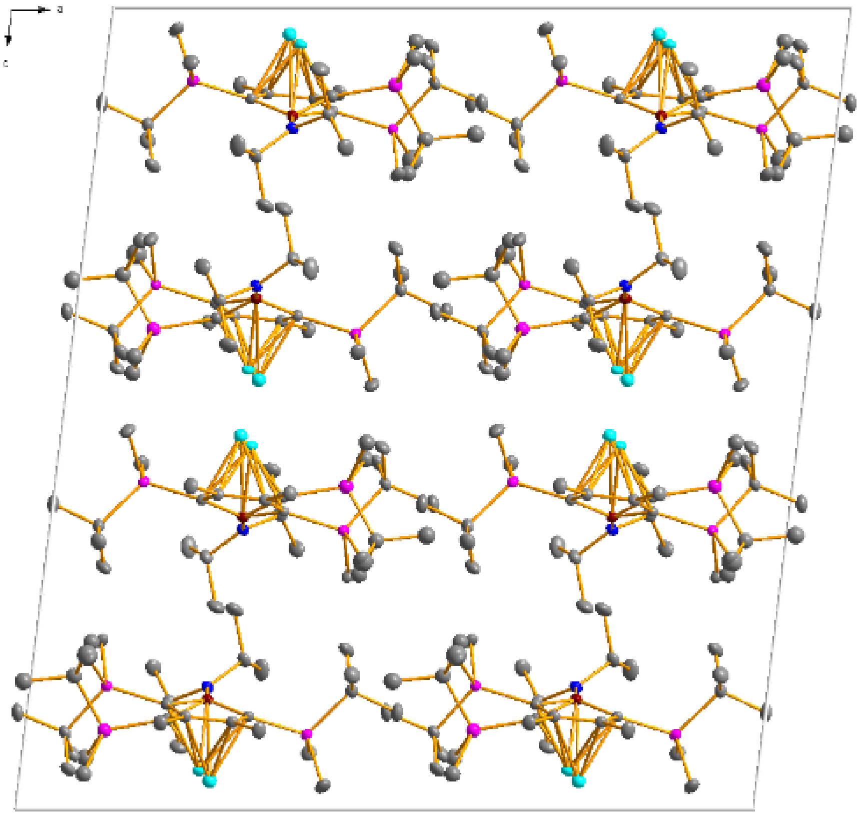

Table S4. Summary of crystallographic data of N^{i}Pr$_2$-substituted boragerma[5]pyramidane **14e** (CCSD 2520811).

Empirical formula	$C_{24}H_{50}BGeNSi_2$	
Formula weight	492.23	
Temperature	100(2) K	
Wavelength	0.71073 Å	
Crystal system	Monoclinic	
Space group	C2/c	
Unit cell dimensions	a = 22.1673(9) Å	α = 90°.
	b = 10.3882(4) Å	β = 96.9763(15)°.
	c = 24.3013(9) Å	γ = 90°.
Volume	5554.6(4) Å^3	
Z	8	
Density (calculated)	1.177 Mg/m^3	
Absorption coefficient	1.200 mm^{-1}	
F(000)	2128	
Crystal size	0.240 x 0.180 x 0.140 mm^3	
Θ range for data collection	1.688 to 40.249°	
Index ranges	-40<=h<=40,-18<=k<=18,-44<=l<=44	
Reflections collected	219899	
Independent reflections	17489 (R(int) = 0.0270)	
Observed reflections (I > 2(I))	16036	
Completeness to Θ = 40.249°	100.0 %	
Absorption correction	Semi-empirical from equivalents	
Max. and min. transmission	1.0000 and 0.9107	
Refinement method	Full-matrix least-squares on F^2	
Data / restraints / parameters	17489 / 15 / 315	
Goodness-of-fit on F^2	1.060	
Final R indices (I>2σ(I))	R_1 = 0.0191, wR_2 = 0.0564	
R indices (all data)	R_1 = 0.0221, wR_2 = 0.0577	
Extinction coefficient	n/a	
Largest diff. peak and hole	0.460 and -0.403 e.Å^{-3}	

Molecular structure of NCy_2-substituted boragerma[5]pyramidane 14f from sc-XRD

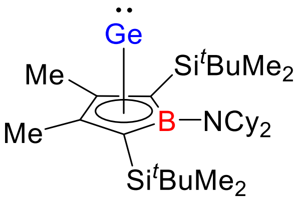

14f

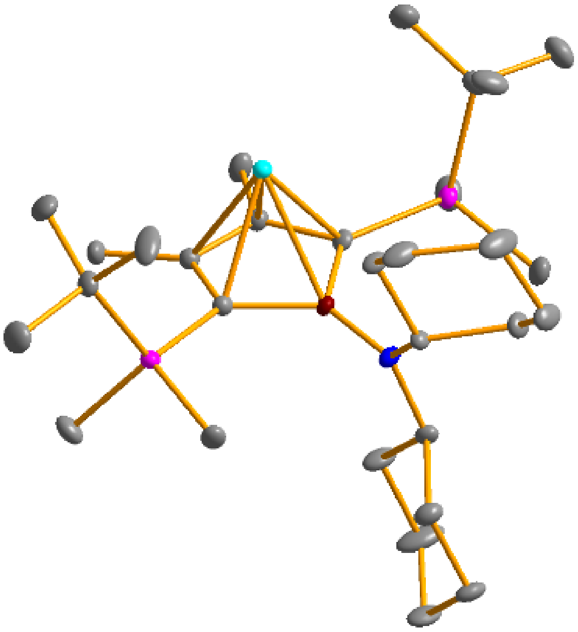

2 of 5 molecules in the asymmetric unit are disordered by 39% or 46%, respectively.

Unit cell (space group: $P\bar{1}$, view along the b-axis, H-atoms omitted for clarity):

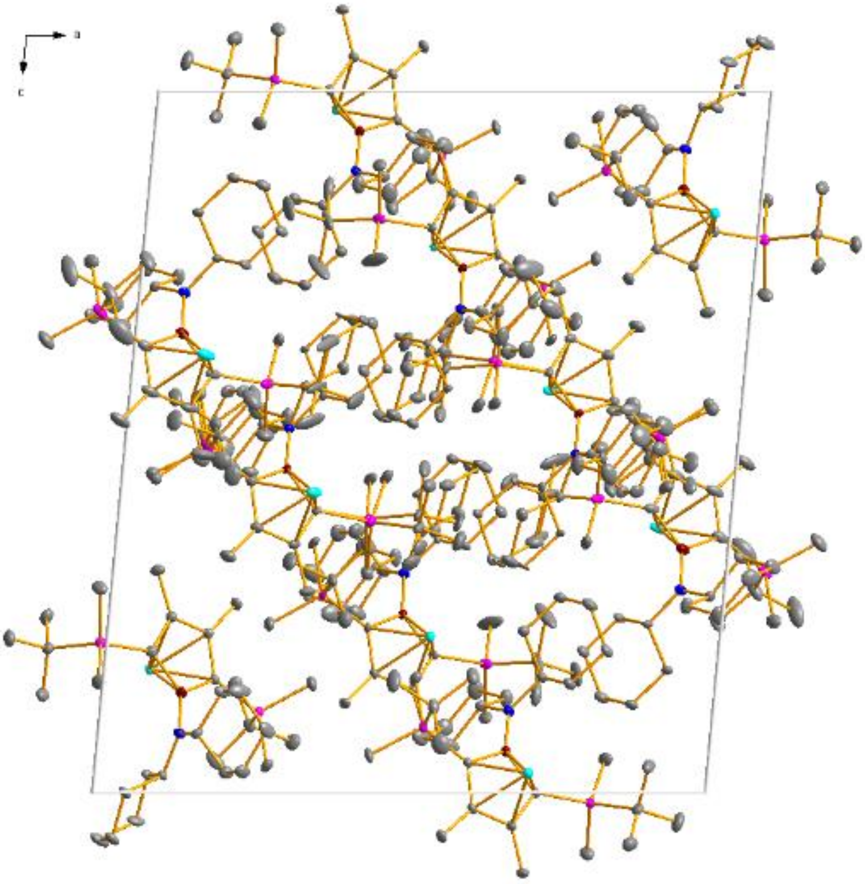

Table S5. Summary of crystallographic data of NCy_2-substituted boragerma[5]pyramidane **14f**. (CCSD 2520814)

Empirical formula	$C_{30}H_{58}BGeNSi_2$	
Formula weight	572.35	
Temperature	100(2) K	
Wavelength	0.71073 Å	
Crystal system	Triclinic	
Space group	$P\bar{1}$	
Unit cell dimensions	a = 10.9942(9) Å	α = 103.646(3)°.
	b = 11.0037(9) Å	β = 93.334(3)°.
	c = 15.6195(13) Å	γ = 116.464(2)°.
Volume	1614.6(2) $Å^3$	
Z	2	
Density (calculated)	1.177 Mg/m^3	
Absorption coefficient	1.042 mm^{-1}	
F(000)	620	
Crystal size	0.130 x 0.120 x 0.040 mm^3	
Θ range for data collection	1.235 to 30.034°	
Index ranges	-15<=h<=15, -15<=k<=15, -21<=l<=21	
Reflections collected	87228	
Independent reflections	9458 (R(int) = 0.0288)	
Observed reflections (I > 2(I))	8813	
Completeness to Θ = 30.034°	100.0 %	
Absorption correction	Semi-empirical from equivalents	
Max. and min. transmission	1.0000 and 0.9471	
Refinement method	Full-matrix least-squares on F^2	
Data / restraints / parameters	9458 / 0 / 564	
Goodness-of-fit on F^2	1.133	
Final R indices (I>2σ(I))	R_1 = 0.0346, wR_2 = 0.0756	
R indices (all data)	R_1 = 0.0383, wR_2 = 0.0784	
Extinction coefficient	n/a	
Largest diff. peak and hole	1.235 and -2.932 e.$Å^{-3}$	

Molecular structure of Fc-substituted boragerma[5]pyramidane 12g from sc-XRD

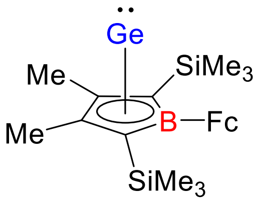

12g

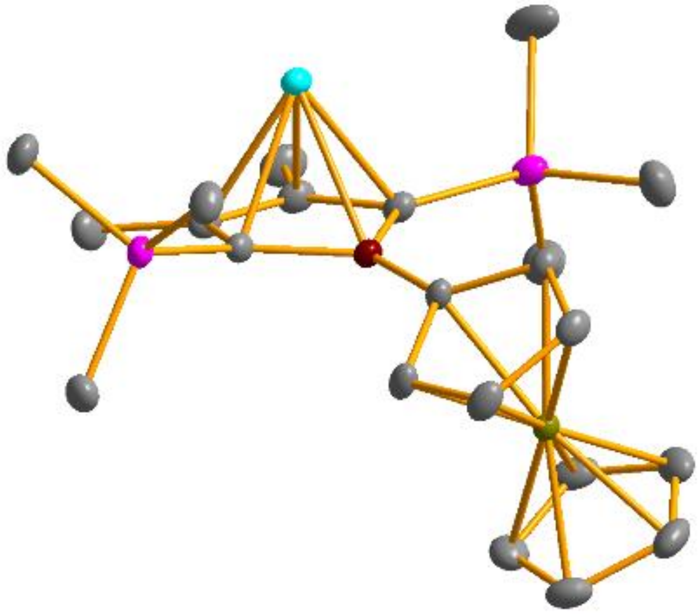

Unit cell (space group: $Pca2_1$, view along the b-axis, H-atoms omitted for clarity):

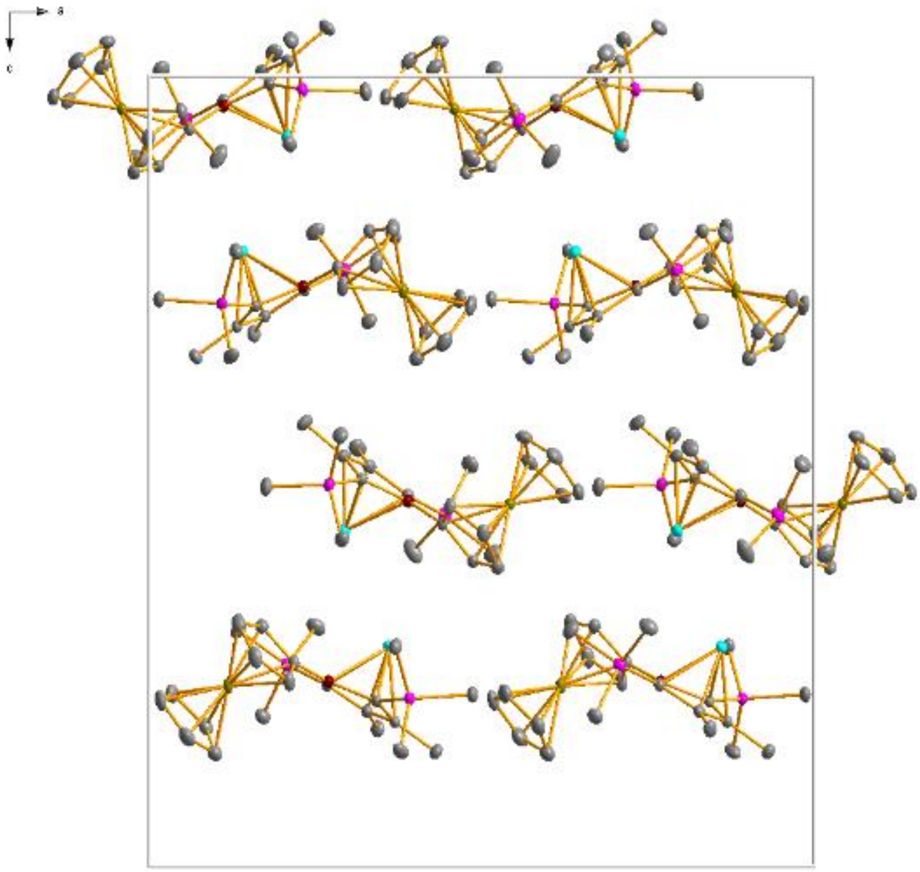

Table S6. Summary of crystallographic data of Fc-substituted boragerma[5]pyramidane **12g** (CCSD 2520812).

Empirical formula	$C_{22}H_{33}BFeGeSi_2$	
Formula weight	492.91	
Temperature	100(2) K	
Wavelength	0.71073 Å	
Crystal system	Orthorhombic	
Space group	$Pca2_1$	
Unit cell dimensions	a = 19.5813(7) Å	α = 90°.
	b = 10.3004(3) Å	β = 90°.
	c = 23.2825(8) Å	γ = 90°.
Volume	4696.0(3) $Å^3$	
Z	8	
Density (calculated)	1.394 Mg/m^3	
Absorption coefficient	2.005 mm^{-1}	
F(000)	2048	
Crystal size	0.170 x 0.140 x 0.070 mm^3	
Θ range for data collection	1.749 to 34.970°	
Index ranges	-31<=h<=31, -16<=k<=16, -37<=l<=37	
Reflections collected	235742	
Independent reflections	20630 (R(int) = 0.0394)	
Observed reflections (I > 2(I))	19349	
Completeness to Θ = 34.970°	100.0 %	
Absorption correction	Semi-empirical from equivalents	
Max. and min. transmission	1.0000 and 0.9064	
Refinement method	Full-matrix least-squares on F^2	
Data / restraints / parameters	20630 / 1 / 504	
Goodness-of-fit on F^2	1.035	
Final R indices (I>2σ(I))	R_1 = 0.0239, wR_2 = 0.0543	
R indices (all data)	R_1 = 0.0276, wR_2 = 0.0557	
Absolute structure parameter	0.328(5)	
Extinction coefficient	n/a	
Largest diff. peak and hole	0.482 and -0.411 e.$Å^{-3}$	

Molecular structure of Fc-substituted boragerma[5]pyramidane 14g from sc-XRD

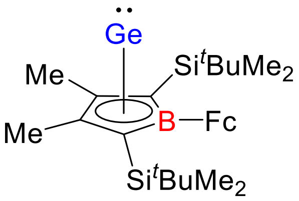

14g

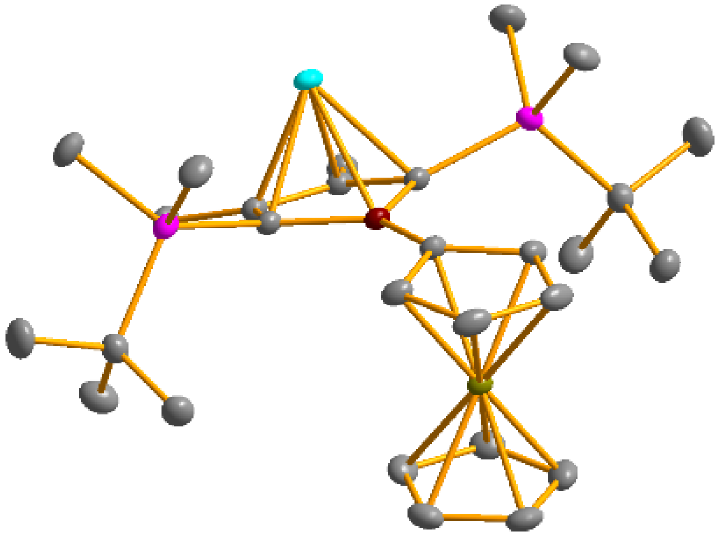

Unit cell (space group: C2/m, view along the b-axis, H-atoms omitted for clarity):

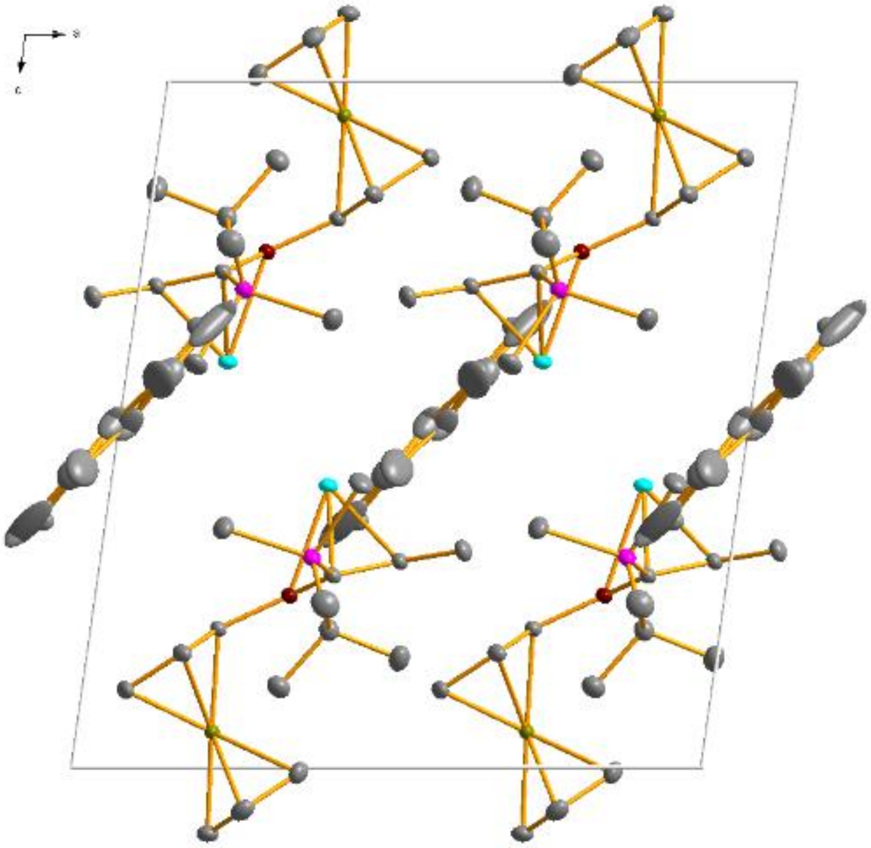

Table S7. Summary of crystallographic data of Fc-substituted boragerma[5]pyramidane **14g** (CCSD 2520810).

Empirical formula	$C_{30.50}H_{51}BFeGeSi_2$	
Formula weight	613.14	
Temperature	100(2) K	
Wavelength	0.71073 Å	
Crystal system	Monoclinic	
Space group	C2/m	
Unit cell dimensions	a = 12.6324(5) Å	α = 90°.
	b = 18.1055(7) Å	β = 98.0107(15)°.
	c = 13.9251(6) Å	γ = 90°.
Volume	3153.8(2) $Å^3$	
Z	4	
Density (calculated)	1.291 Mg/m^3	
Absorption coefficient	1.507 mm^{-1}	
F(000)	1300	
Crystal size	0.140 x 0.130 x 0.120 mm^3	
Θ range for data collection	1.477 to 40.249°	
Index ranges	-22<=h<=22,-32<=k<=32,-25<=l<=25	
Reflections collected	137067	
Independent reflections	10157 (R(int) = 0.0282)	
Observed reflections (I > 2(I))	9576	
Completeness to Θ = 40.249°	100.0 %	
Absorption correction	Semi-empirical from equivalents	
Max. and min. transmission	1.0000 and 0.9298	
Refinement method	Full-matrix least-squares on F^2	
Data / restraints / parameters	10157 / 25 / 210	
Goodness-of-fit on F^2	1.057	
Final R indices (I>2σ(I))	R_1 = 0.0195, wR_2 = 0.0569	
R indices (all data)	R_1 = 0.0210, wR_2 = 0.0576	
Extinction coefficient	n/a	
Largest diff. peak and hole	0.892 and -0.470 e.$Å^{-3}$	

Molecular structure of Fc-substituted borole 25 from sc-XRD

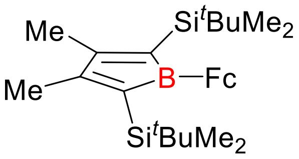

25

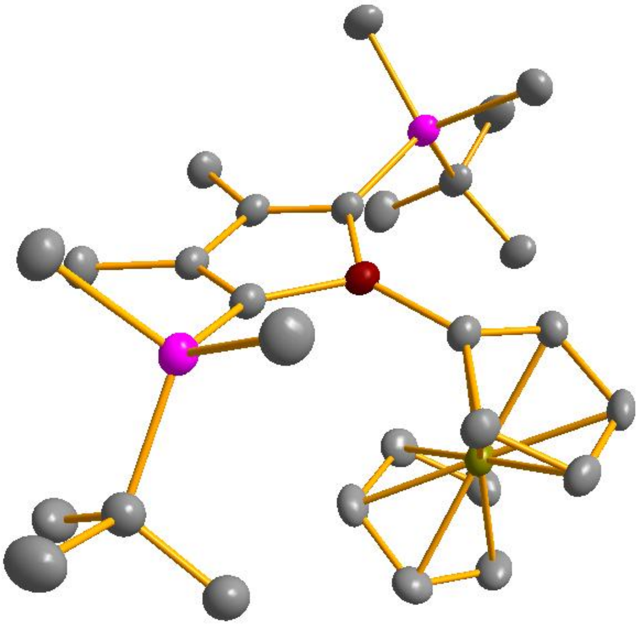

Unit cell (space group: P-1, view along the b-axis, H-atoms omitted for clarity, *n*-pentane disordered by 50%):

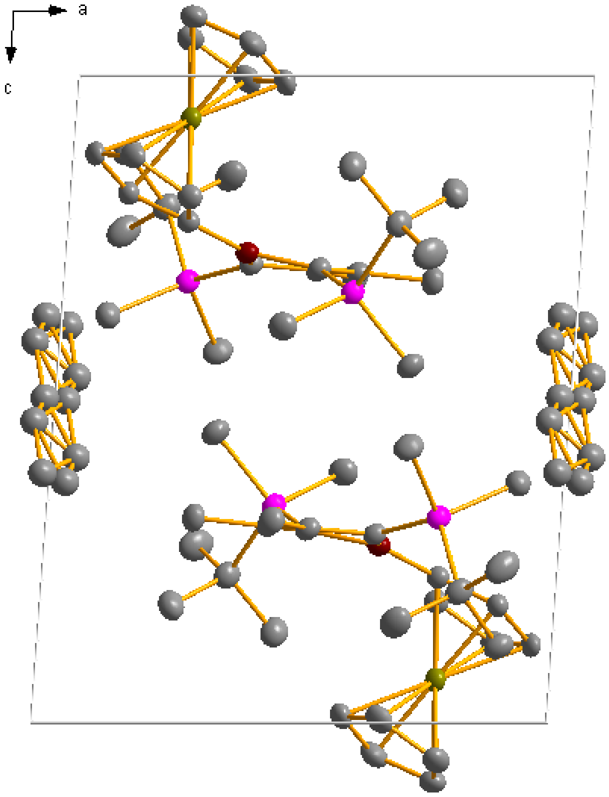

Table S8. Summary of crystallographic data of Fc-substituted borole **25** (CCSD 2520807).

Empirical formula	$C_{30.50}H_{51}BFeSi_2$	
Formula weight	540.55	
Temperature	100(2) K	
Wavelength	1.54178 Å	
Crystal system	Triclinic	
Space group	P-1	
Unit cell dimensions	a = 11.0726(4) Å	α = 71.597(2)°.
	b = 11.3541(4) Å	β = 87.232(2)°.
	c = 13.6969(5) Å	γ = 68.706(2)°.
Volume	1518.06(10) $Å^3$	
Z	2	
Density (calculated)	1.183 Mg/m^3	
Absorption coefficient	1.448 mm^{-1}	
F(000)	586	
Crystal size	0.100 x 0.060 x 0.020 mm^3	
Θ range for data collection	3.410 to 74.450°	
Index ranges	-13<=h<=13, -14<=k<=13, -17<=l<=16	
Reflections collected	27858	
Independent reflections	6164 (R(int) = 0.0618)	
Observed reflections (I > 2(I))	5287	
Completeness to Θ = 74.450°	99.3 %	
Absorption correction	Semi-empirical from equivalents	
Max. and min. transmission	1.0000 and 0.8385	
Refinement method	Full-matrix least-squares on F^2	
Data / restraints / parameters	6164 / 0 / 348	
Goodness-of-fit on F^2	1.011	
Final R indices (I>2σ(I))	R_1 = 0.0400, wR_2 = 0.0992	
R indices (all data)	R_1 = 0.0503, wR_2 = 0.1052	
Absolute structure parameter	0.381(8)	
Extinction coefficient	n/a	
Largest diff. peak and hole	0.829 and -0.251 e.$Å^{-3}$	

Molecular structure of tungsten complex 30 from sc-XRD

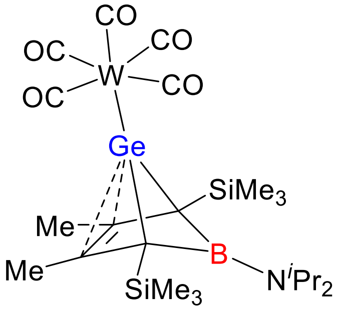

30

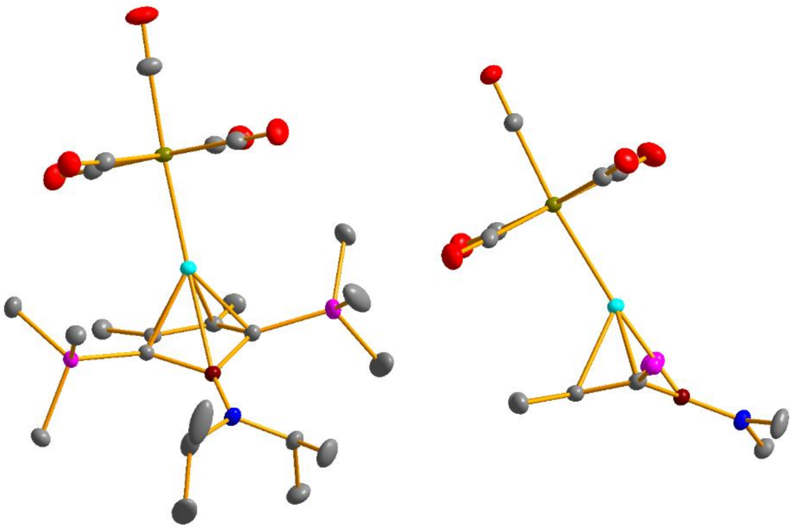

Unit cell (space group: $P\bar{1}$, view along the b-axis, H-atoms omitted for clarity):

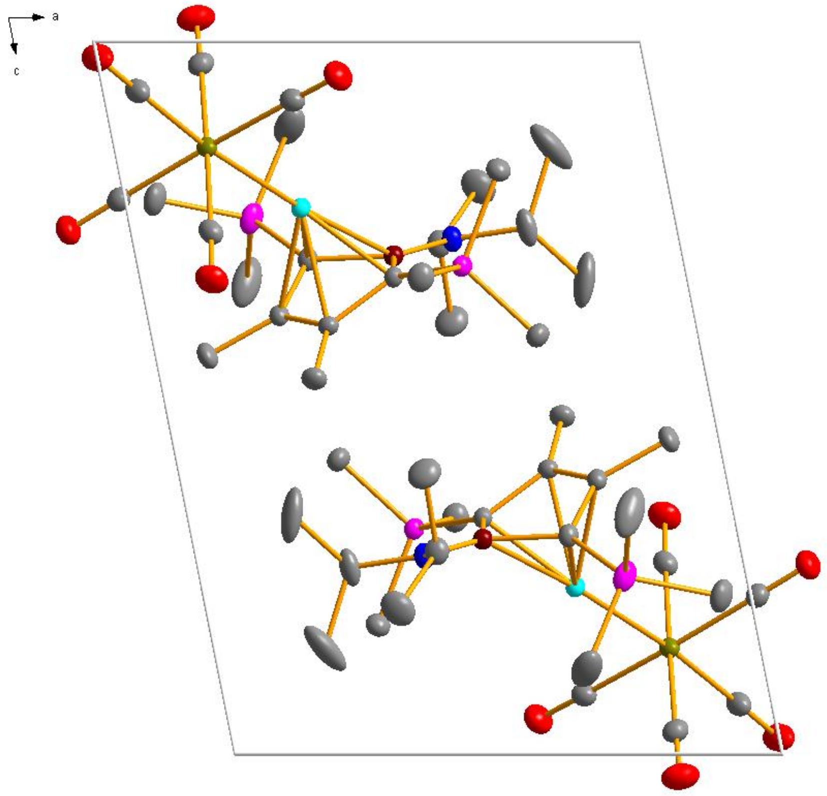

Table S9. Summary of crystallographic data of tungsten complex **30** (CCSD 2520809).

Empirical formula	$C_{23}H_{38}BGeNO_5Si_2W$	
Formula weight	731.97	
Temperature	100(2) K	
Wavelength	0.71073 Å	
Crystal system	Triclinic	
Space group	$P\bar{1}$	
Unit cell dimensions	a = 10.6810(8) Å	α = 80.179(3)°.
	b = 11.7790(8) Å	β = 76.057(3)°.
	c = 13.4175(9) Å	γ = 68.742(3)°.
Volume	1520.38(19) $Å^3$	
Z	2	
Density (calculated)	1.599 Mg/m^3	
Absorption coefficient	4.877 mm^{-1}	
F(000)	724	
Crystal size	0.100 x 0.090 x 0.040 mm^3	
Θ range for data collection	1.570 to 34.971°	
Index ranges	-17<=h<=17,-18<=k<=18,-21<=l<=21	
Reflections collected	116191	
Independent reflections	13347 (R(int) = 0.0437)	
Observed reflections (I > 2(I))	12324	
Completeness to Θ = 34.971°	100.0 %	
Absorption correction	Semi-empirical from equivalents	
Max. and min. transmission	1.0000 and 0.8425	
Refinement method	Full-matrix least-squares on F^2	
Data / restraints / parameters	13347 / 0 / 319	
Goodness-of-fit on F^2	1.115	
Final R indices (I>2σ(I))	R_1 = 0.0192, wR_2 = 0.0450	
R indices (all data)	R_1 = 0.0230, wR_2 = 0.0461	
Extinction coefficient	n/a	
Largest diff. peak and hole	2.459 and -0.902 e.$Å^{-3}$	

Table S10. Comparison of experimental structural data of all crystallized compounds (bond lengths in [pm], bond and dihedral angles [°]).

	**14a**	**12e**	**14e**	**14f**	**12g**	**14g**
Ge-W [pm]		-	-	-	-	-
Ge-B [pm]	230.7(2)	253.4(2)	235.26(5)	233.2(2)	231.35(19)	234.37(7)
Ge-C1 [pm]	220.5(2)	218.2(3)	220.26(4)	219.6(2)	219.55(19)	219.31(5)
Ge-C2 [pm]	220.8(2)	217.6(3)	219.80(4)	219.6(2)	222.7(2)	220.34(5)
Ge-C3 [pm]	220.4(1)	218.0(3)	216.62(4)	218.5(3)	219.2(2)	-
Ge-C4 [pm]	220.4(1)	218.3(3)	221.98(5)	218.7(2)	219.48(18)	-
C1-C2 [pm]	145.9(2)	147.4(4)	146.36(6)	146.4(3)	146.1(3)	146.75(7)
C2-C3 [pm]	142.3(2)	142.9(3)	142.97(6)	142.5(3)	142.6(3)	142.75 (10)
C3-C4 [pm]	146.4(2)	147.2(4)	146.34(6)	147.0(3)	146.6(3)	-
B-C1 [pm]	158.0(2)	158.7(4)	158.86(6)	157.6(3)	156.9(3)	157.38(6)
B-C4 [pm]	158.1(2)	158.3(4)	158.07(6)	157.8(3)	156.8(3)	-
B-N [pm]		142.5(2)	145.53(6)	147.9(3)	-	-
B-C^{α} [pm]	162.3(2)	-	-	-	157.4(3)	157.14(9)
Ge-B-N-C^{α} [°]	-	89.9(4)	128.8(4)	168.0(2)	-	-
Ge-B-C^{α}-C^{β} [°]	145.1(1)	-	-	-	120.1(2)	96.8(7)
α(Ge) [°]	80.7	79.5	79.0	80.2	81.0	80.8
α(B) [°]	171.5	152.6	171.1	169.6	169.9	167.2
∑β(B) [°]	360.0	360.0	359.7	359.0	360.0	360.0

	**30**			**25**
Ge-W [pm]	260.03(2)		B-Fe [pm]	302.2(2)
Ge-B [pm]	244.84(15)		Ge-B [pm]	
Ge-C1 [pm]	210.22(13)		Ge-C1 [pm]	
Ge-C2 [pm]	220.78(13)		Ge-C2 [pm]	
Ge-C3 [pm]	221.20(14)		Ge-C3 [pm]	
Ge-C4 [pm]	210.95(14)		Ge-C4 [pm]	
C1-C2 [pm]	148.55(19)		C1-C2 [pm]	136.29(26)
C2-C3 [pm]	141.9(2)		C2-C3 [pm]	151.56(35)
C3-C4 [pm]	147.7(2)		C3-C4 [pm]	135.79(27)
B-C1 [pm]	160.2(2)		B-C1 [pm]	160.38(32)

B-C4 [pm]	160.0(2)		B-C4 [pm]	159.85(33)
B-N [pm]	141.2(2)		B-N [pm]	
B-C^{α} [pm]	-		B-C^{α} [pm]	154.26(23)
Ge-B-N-C^{α} [°]	82.7(2)		Ge-B-N-C^{α} [°]	
Ge-B-C^{α}-C^{β} [°]	-		Ge-B-C^{α}-C^{β} [°]	
α(Ge) [°]	84.6		α(BFc) [°]	13.0
α(B) [°]	149.0		α(B) [°]	167.7
∑β(B) [°]	360.0		∑β(B) [°]	359.3

Computational Details.

All quantum chemical calculations were performed using the Gaussian16 software package.[15] For the Natural Bond Orbital (NBO) analyses,[16] the NBO 7.0 program was used,[17] and the Jmol16 program was used for graphical representation.[18] The Quantum Theory of Atoms In Molecules (QTAIM) analyses were performed using the AIMALL program.[19] The optimizations were performed using the hybrid functional M06-2X[20] and the 6-311+G(d,p) basis set or the Def2-TZVP basis set. The SCF energies and Gibbs free energies (at T = 298.15 K and p = 0.101 MPa (isolated molecule)) of all optimized molecular structures are listed in Tables S11, and S12. Subsequent frequency calculations determined the stationary points either as a minimum (number of imaginary frequencies NImag = 0) or as a transition state (NImag = 1). NMR chemical shift calculations were carried out using the GIAO method with the functional M06-L[21] and the 6-311G(2d,p) basis set for molecular structures optimized at the M06-2X/6-311+G(d,p) level of theory.

Table S11. Calculated absolute energies E(SCF) and Gibbs free energies G^{298} at T = 298 K, p = 0.101 MPa, number and size of imaginary frequencies (energies in a.u., at M06-2X/6-311+G(d,p).

Verbindung	E(SCF) [a.u.]	NImag; $\tilde{\nu}$ [cm^{-1}]; ZPVE [kJ/mol]	G^{298} [a.u.]
**12a**	-3542.52813	0 ; - ; 1463	-3542.03505
**12b**	-4025.81401	0 ; - ; 1481	-4025.31798
**12d**	-3502.06681	0 ; - ; 1328	-3501.62445
**12e**	-3444.29909	0 ; - ; 1391	-3443.82984
**12f**	-3677.73437	0 ; - ; 1743	-3677.13526
**12g**	-4802.56877	0 ; - ; 1297	-4802.13726
**12i**	-3880.33076	0 ; - ; 1003	-3880.01493
**20f**	-2273.65168	0 ; - ; 2024	-2272.95400
**30**[a]	-4078.22679	0; - ; 1503	-4077.73679

a) For tungsten the def2-TZVP basis set and effective core potential was used.

Table S12. Calculated absolute energies E(SCF) and Gibbs free energies G^{298} at T = 298 K, p = 0.101 MPa, number and size of imaginary frequencies (energies in a.u., at M06-2X/Def2-TZVP).

**Verbindung**	**E(SCF) [a.u.]**	**NImag; $\tilde{\nu}$ [cm^{-1}]; ZPVE [kJ/mol]**	**G^{298} [a.u.]**
per-**35**	-235.57925	2 ; -884, -541 ; 245	-235.51448
per-**35**	-235.58380	1 ; -599 ; 248	-235.51798
pl-**35**	-235.62710	0 ; - ; 255	-235.55834
per-**12M**	-2312.66341	2 ; -482, -415 ; 253	-2312.59749
per-**12M**	-2312.66586	1 ; -337 ; 256	-2312.59891
pl-**12M**	-2312.68146	0 ; - ; 258	-2312.61442
**36**	-734.94265	0 ; - ; 224	-734.88652
per-**37**	-411.24475	1 ; -79, 421	-411.11860
pl-**37**	-411.25655	0 ; - ; 423	-411.13049
per-**38**	-2488.33237	1 ; -40; 429	-2488.20464
pl-**38**	-2488.33677	0 ; - ; 428	-2488.21090
**14g**	-5038.57479	0 ; - ; 1749	-5037.97652
**39**	-525.03863	0 ; - ; 254	-524.97478
**TS(39/41)**	-525.03337	1 ; -74 ; 251	-524.96951
**41**	-525.09587	0 ; - ; 258	-525.02800
**TS(41/43)**	-525.06135	1 ; -414 ; 255	-524.99420
**43**	-525.13991	0 ; - ; 260	-525.07092

Table S13. Comparison of calculated structural data of boragerma[5]pyramidanes **12d-g** (all at M06-2X/6-311+G(d,p) level of theory), boragerma[5]pyramidane **14g** and tungsten complex **30** (both at M06-2X/Def2-TZVP level of theory).

	**12d**	**12e**	**12f**	**12g**	**14g**	**30**
Ge-W [pm]	-	-	-	-	-	*264.8*
Ge-B [pm]	*229.5*	*247.6*	*236.2*	*231.1*	*232.7*	*243.1*
Ge-C1 [pm]	*220.1*	*218.2*	*220.0*	*219.2*	*219.5*	*210.5*
Ge-C2 [pm]	*219.6*	*218.9*	*217.3*	*221.7*	*220.6*	*217.6*
Ge-C3 [pm]	*220.5*	*218.1*	*220.5*	*219.5*	-	*218.7*
Ge-C4 [pm]	*219.4*	*217.8*	*218.8*	*218.6*	-	*211.4*
C1-C2 [pm]	*145.4*	*147.3*	*146.4*	*144.9*	*145.3*	*148.0*
C2-C3 [pm]	*143.3*	*142.9*	*142.6*	*143.0*	*142.5*	*141.2*
C3-C4 [pm]	*145.1*	*146.2*	*145.3*	*146.1*		*147.0*
B-C1 [pm]	*154.6*	*158.5*	*157.6*	*156.1*	*156.8*	*159.6*
B-C4 [pm]	*155.3*	*158.0*	*157.7*	*155.8*	-	*159.1*
B-C^{ipso} [pm]	*158.1*	-	-	-	-	-
B-N [pm]	-	*143.5*	*145.5*	-	-	*142.0*
B-C^{α} [pm]	-	-	-	*157.1*	*156.5*	-
Ge-B-C^{ipso}-C^{ortho} [°]	*178.0*	-	-	-	-	-
Ge-B-N-C^{α} [°]	-	*84.5*	*127.1*	-	-	*84.4*
Ge-B-C^{α}-C^{β}[°]	-	-	-	*125.1*	*94.5*	-
α(Ge) [°]	*80.0*	*80.2*	*79.9*	*80.9*	*80.9*	*83.1*
α(B) [°]	*173.0*	*157.6*	*168.8*	*170.0*	*168.3*	*152.2*
∑β(B) [°]	*359.9*	*360.0*	*359.7*	*360.0*	*360.0*	*360.0*

Table S14. Comparison of experimental and calculated (at M06-2X/6-311+G(d,p) level of theory) structural data of N^{i}Pr$_2$-substituted boragerma[5]pyramidane **12e**.

	**12e** (exp.)	**12e** (*calc.*)	**deviation** (*abs.*)	**deviation** (*%*)
Ge-B [pm]	253.4(2)	*247.6*	*5.8*	*2.3*
Ge-C1 [pm]	218.2(3)	*218.2*	*0.0*	*0.0*
Ge-C2 [pm]	217.6(3)	*218.9*	*1.3*	*0.6*
Ge-C3 [pm]	218.0(3)	*218.1*	*0.1*	*0.0*
Ge-C4 [pm]	218.3(3)	*217.8*	*0.5*	*0.2*
C1-C2 [pm]	147.4(4)	*147.3*	*0.1*	*0.1*
C2-C3 [pm]	142.9(3)	*142.9*	*0.0*	*0.0*
C3-C4 [pm]	147.2(4)	*146.2*	*1.0*	*0.7*
B-C1 [pm]	158.7(4)	*158.5*	*0.2*	*0.1*
B-C4 [pm]	158.3(4)	*158.0*	*0.3*	*0.2*
B-N [pm]	142.5(2)	*143.5*	*1.0*	*0.7*
Ge-B-N-C16 [°]	89.852(355)	*84.5*	*5.4*	*6.0*
α(Ge) [°]	79.5	*80.2*	*0.7*	*0.9*
α(B) [°]	152.6	*157.6*	*5.0*	*3.3*
∑β(B) [°]	360.0	*360.0*	*0.0*	*0.0*

Table S15. Comparison of experimental and calculated (at M06-2X/6-311+G(d,p) level of theory) structural data of ferrocenyl-substituted boragerma[5]pyramidane **12g**.

	**6g** (exp.)	**6g** (*calc.*)	**deviation** (*abs.*)	**deviation** (*%*)
Ge-B [pm]	231.35(19)	*231.1*	*0.3*	*0.1*
Ge-C1 [pm]	219.55(19)	*219.2*	*0.4*	*0.2*
Ge-C2 [pm]	222.7(2)	*221.7*	*1.0*	*0.4*
Ge-C3 [pm]	219.2(2)	*219.5*	*0.3*	*0.1*
Ge-C4 [pm]	219.48(18)	*218.6*	*0.9*	*0.4*
C1-C2 [pm]	146.1(3)	*144.9*	*1.2*	*0.8*
C2-C3 [pm]	142.6(3)	*143.0*	*0.4*	*0.3*
C3-C4 [pm]	146.6(3)	*146.1*	*0.5*	*0.3*
B-C1 [pm]	156.9(3)	*156.1*	*0.8*	*0.5*
B-C4 [pm]	156.8(3)	*155.8*	*1.0*	*0.6*
B-C13 [pm]	157.4(3)	*157.1*	*0.3*	*0.2*
Ge-B-C13-C17 [°]	120.070(182)	*125.1*	*5.0*	*4.2*
α(Ge) [°]	81.0	*80.9*	*0.1*	*0.1*
α(B) [°]	169.9	*170.0*	*0.1*	*0.1*
∑β(B) [°]	360.0	*360.0*	*0.0*	*0.0*

Table S16. Comparison of experimental and calculated (at M06-2X/Def2-TZVP level of theory) structural data of ferrocenyl-substituted boragerma[5]pyramidane **14g**.

	**14g** (exp.)	**14g** (*calc.*)	**deviation** (*abs.*)	**deviation** (*%*)
Ge-B [pm]	234.37(7)	*232.7*	*1.7*	*0.7*
Ge-C1 [pm]	219.31(5)	*219.5*	*0.2*	*0.1*
Ge-C2 [pm]	220.34(5)	*220.6*	*0.3*	*0.1*
C1-C2 [pm]	146.75(7)	*145.3*	*1.4*	*1.0*
C2-C2‘ [pm]	142.75(10)	*142.5*	*0.3*	*0.2*
B-C1 [pm]	157.38(6)	*156.8*	*0.6*	*0.4*
B-C10 [pm]	157.14(9)	*156.5*	*0.6*	*0.4*
Ge-B-C10-C11 [°]	96.836(68)	*94.5*	*2.3*	*2.4*
α(Ge) [°]	80.8	*80.9*	*0.1*	*0.1*
α(B) [°]	167.2	*168.3*	*1.1*	*0.7*
∑β(B) [°]	360.0	*360.0*	*0.0*	*0.0*

Table S17. Comparison of experimental and calculated (at M06-2X/Def2-TZVP level of theory) structural data of tungsten carbonyl complex **30**.

	**30** (exp.)	**30** (*calc.*)	**deviation** (*abs.*)	**deviation** (*%*)
Ge-W [pm]	260.03(2)	*264.8*	*4.8*	*1.8*
Ge-B [pm]	244.84(15)	*243.1*	*1.7*	*0.7*
Ge-C1 [pm]	210.22(13)	*210.5*	*0.3*	*0.1*
Ge-C2 [pm]	220.78(13)	*217.6*	*3.2*	*1.4*
Ge-C3 [pm]	221.20(14)	*218.7*	*2.5*	*1.1*
Ge-C4 [pm]	210.95(14)	*211.4*	*0.5*	*0.2*
C1-C2 [pm]	148.55(19)	*148.0*	*0.6*	*0.4*
C2-C3 [pm]	141.9(2)	*141.2*	*0.7*	*0.5*
C3-C4 [pm]	147.7(2)	*147.0*	*0.7*	*0.5*
B-C1 [pm]	160.2(2)	*159.6*	*0.6*	*0.4*
B-C4 [pm]	160.0(2)	*159.1*	*0.9*	*0.6*
B-N [pm]	141.2(2)	*142.0*	*0.8*	*0.6*
Ge-B-N-C16 [°]	82.691(242)	*84.4*	*1.7*	*2.1*
α(Ge) [°]	84.6	*83.1*	*1.5*	*1.8*
α(B) [°]	149.0	*152.2*	*3.2*	*2.1*
∑β(B) [°]	360.0	*360.0*	*0.0*	*0.0*

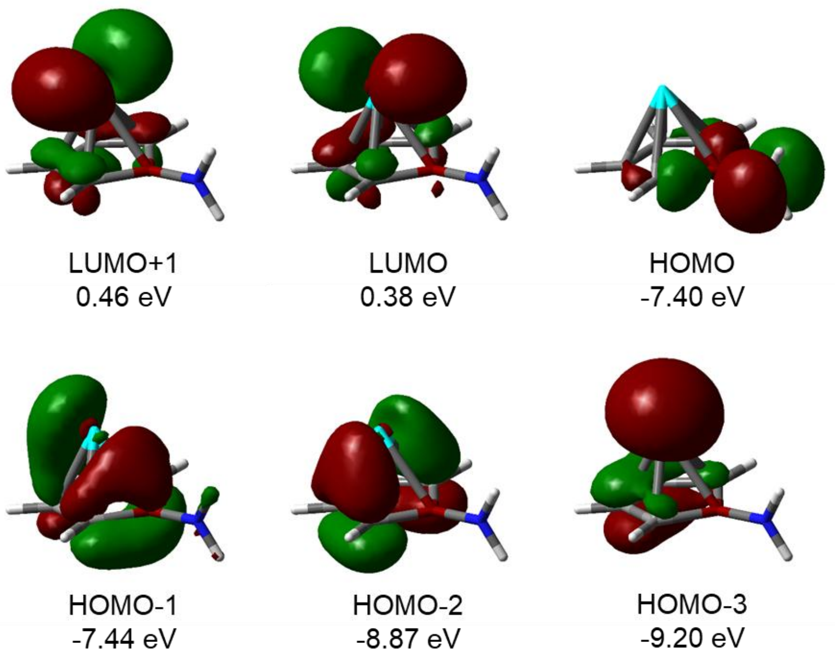

Figure S72. Surface diagrams of selected molecular orbitals of model boragerma[5]pyramidane *per*-**12M** (at M06-2X/Def2-TZVP level of theory, isodensity value 0.05).

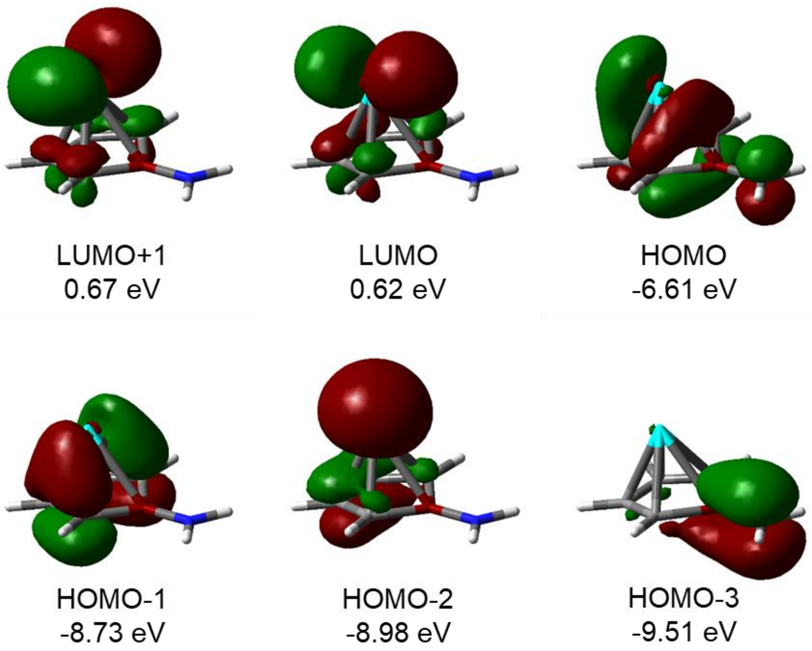

Figure S73. Surface diagrams of selected molecular orbitals of model boragerma[5]pyramidane *pl*-**12M** (at M06-2X/Def2-TZVP level of theory, isodensity value 0.05).

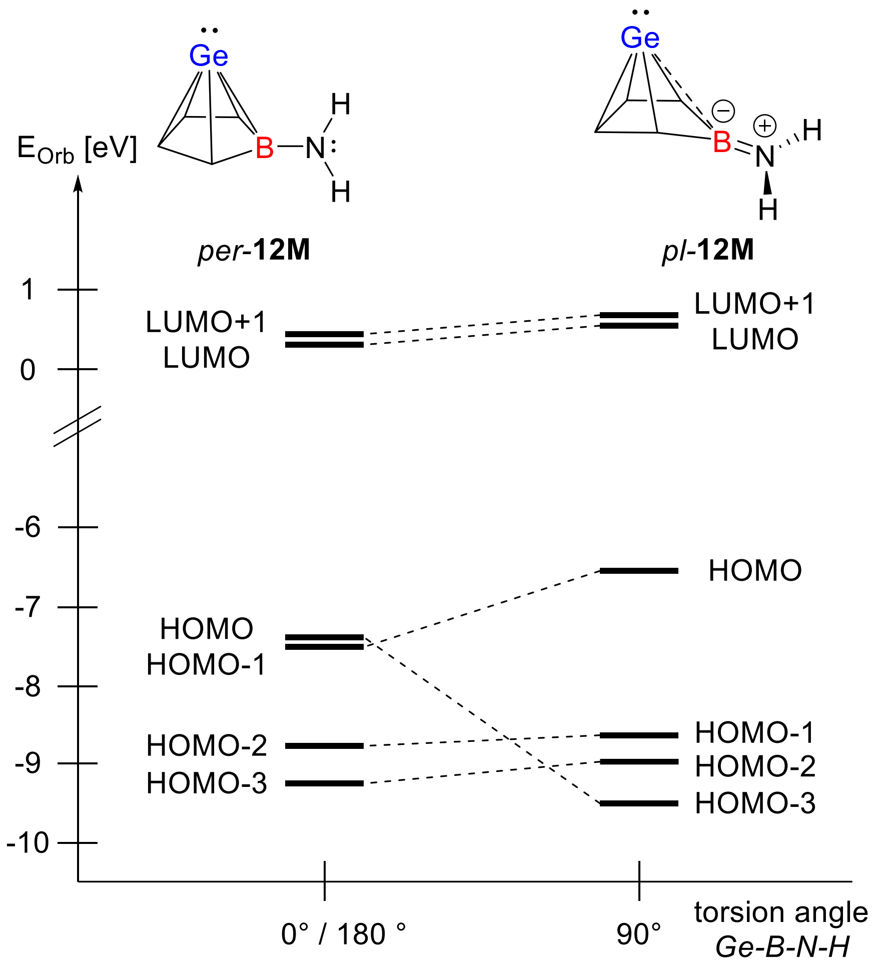

Figure S74. WALSH diagram for model aminoboragerma[5]pyramidane **12M**.

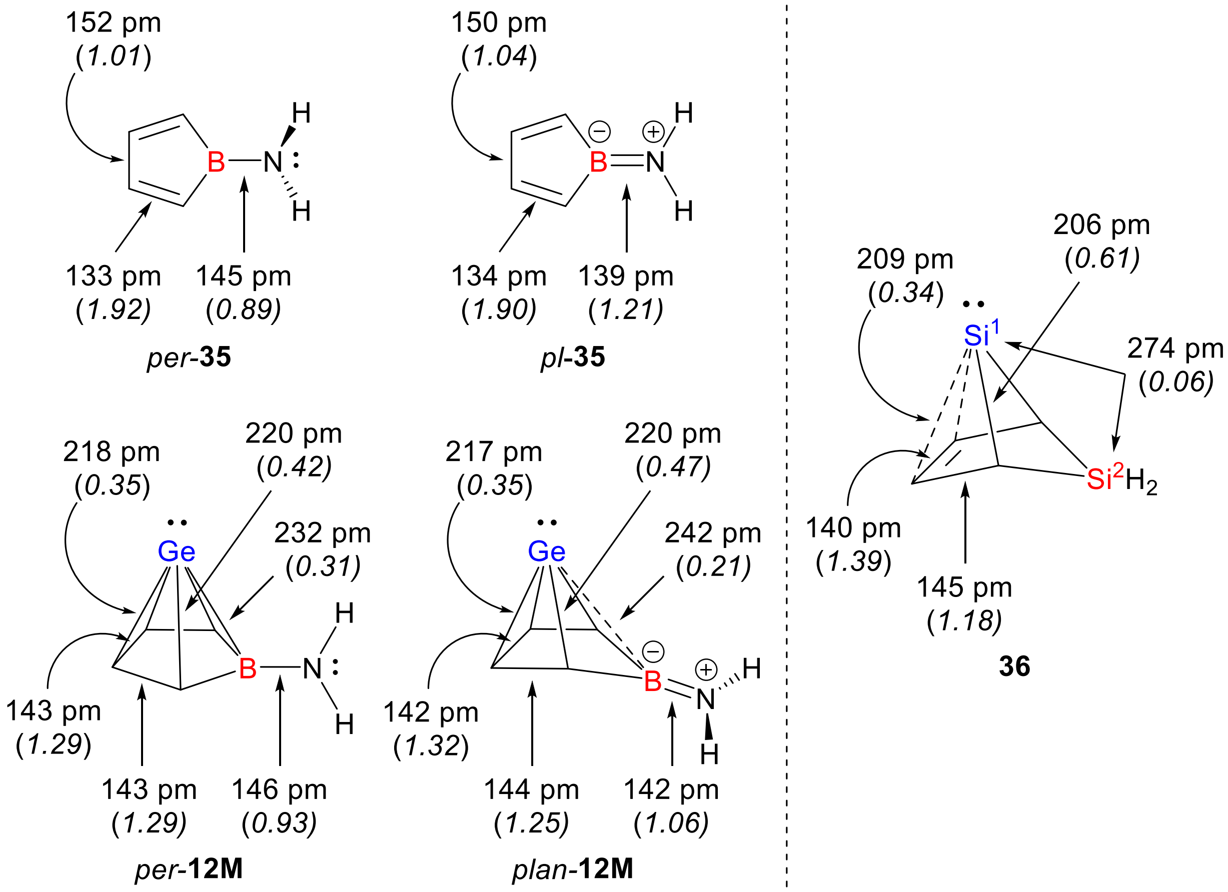

Figure S75. Calculated bond lengths and WIBERG bond indices (*WBIs*) of aminoborole **35** in its planar and perpendicular conformation, model aminoboragerma[5]pyramidane *pl*-**12M** and *per*-**12M** and model sila-bicyclohexene(BCH)-silylene **36** (at M06-2X/Def2-TZVP level of theory).

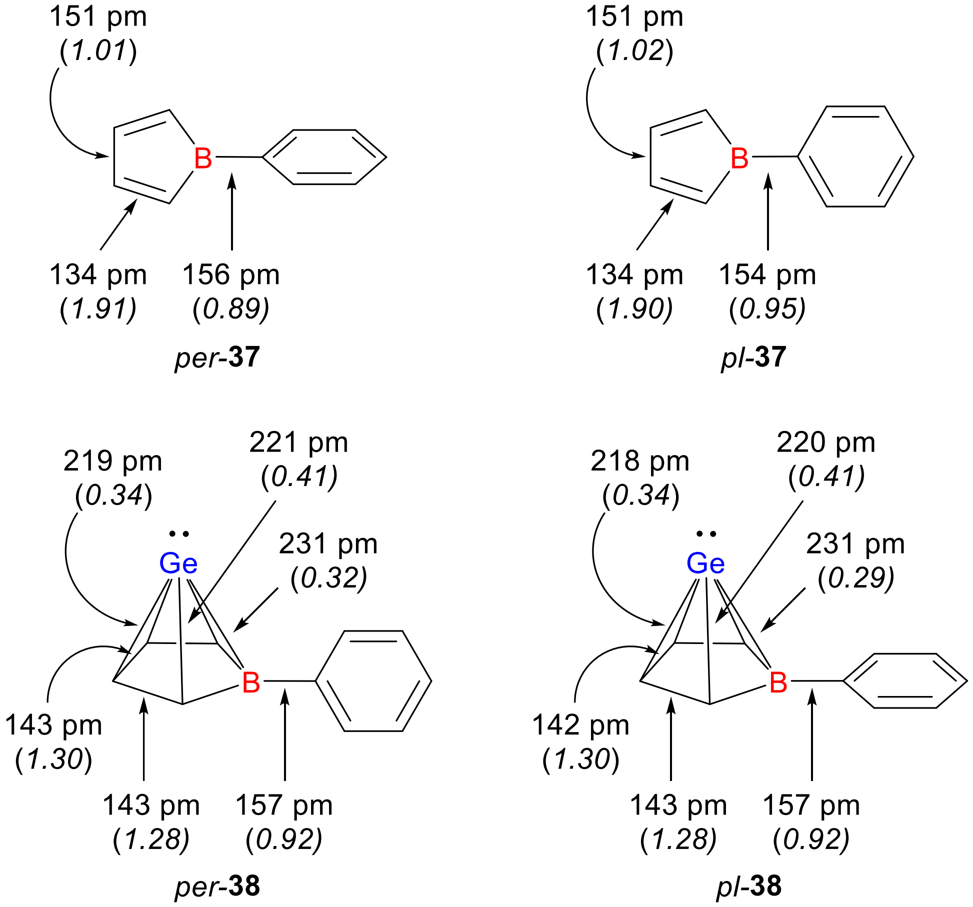

Figure S76. Calculated bond lengths and WIBERG bond indices (*WBIs*) of phenyl aminoborole *per- and pl*-**37** and phenyl aminoboragerma[5]pyramidane *per- and pl*-**38** (at M06-2X/Def2-TZVP level of theory).

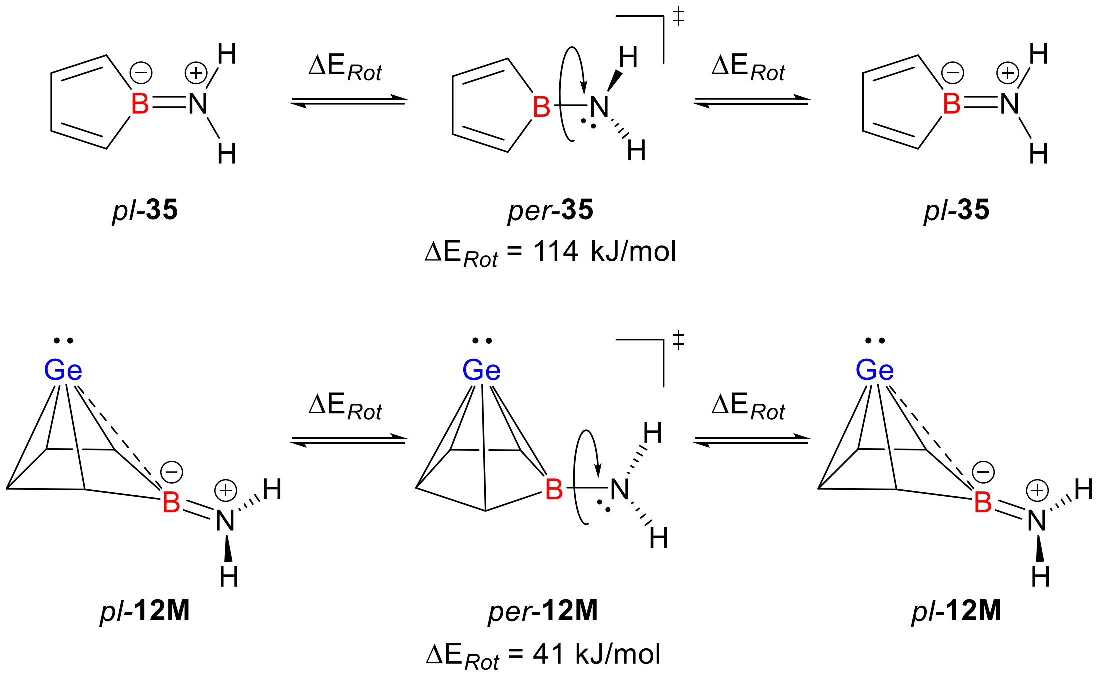

Figure S77. Calculated rotational barriers ΔE_{Rot} about the B-N bond in the model compounds **35** and **12M** (at M06-2X/Def2-TZVP level of theory).

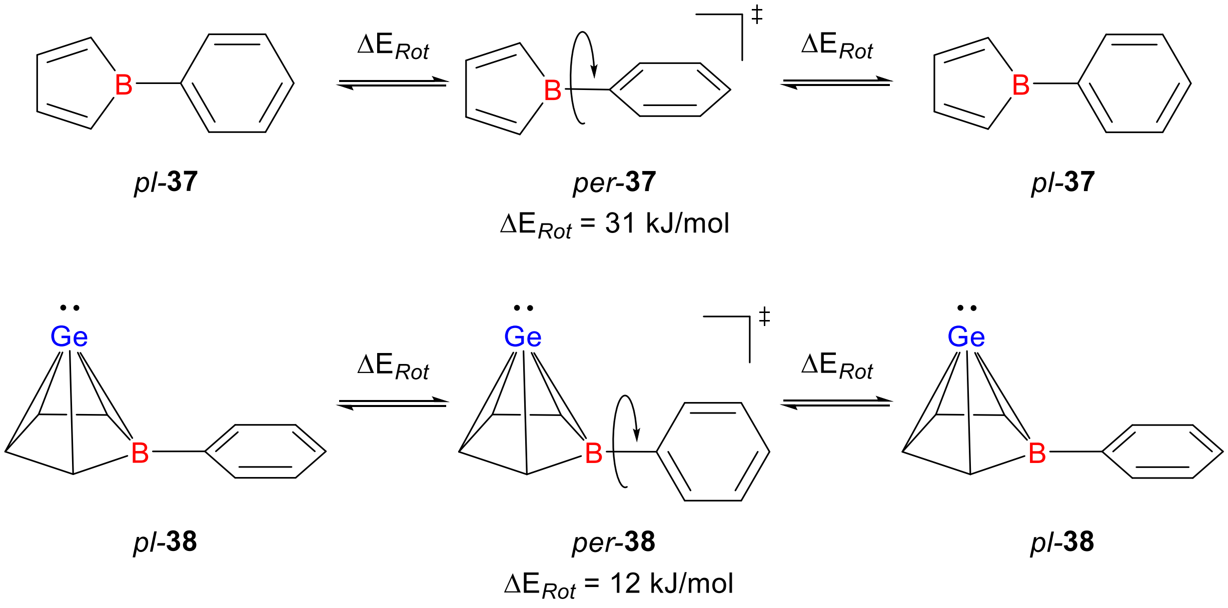

Figure S78. Calculated rotational barriers ΔE_{Rot} about the B-C^{ipso} bond in the model compounds **37** and **38** (at M06-2X/Def2-TZVP level of theory).

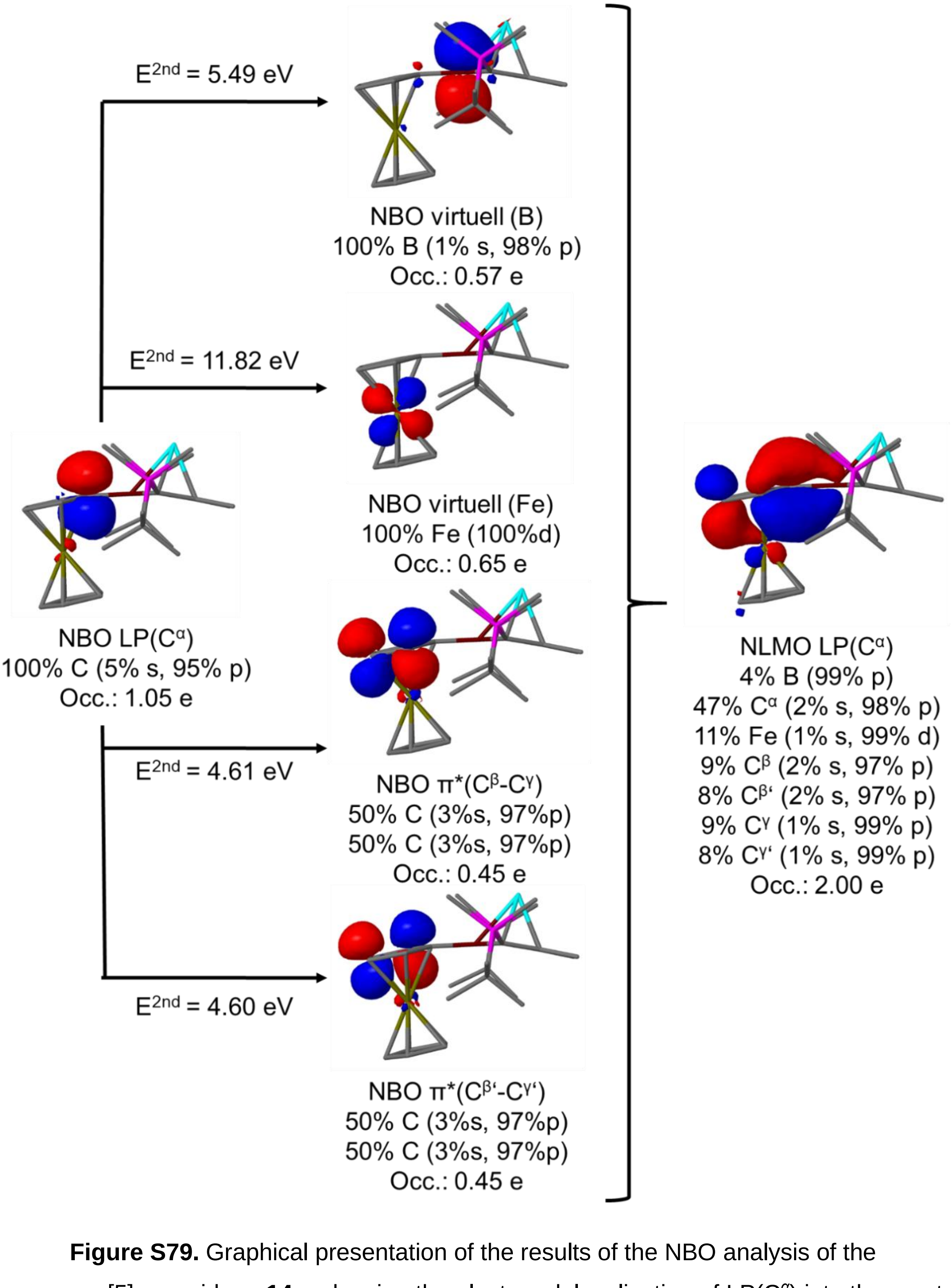

Figure S79. Graphical presentation of the results of the NBO analysis of the boragerma[5]pyramidane **14g**, showing the electron delocalisation of LP(C^{α}) into the empty 2p(B), the 3d(Fe), the π*(C^{β}-C^{γ}) and the π*($C^{\beta'}$-$C^{\gamma'}$) resulting in the NLMO LP(C^{α}).

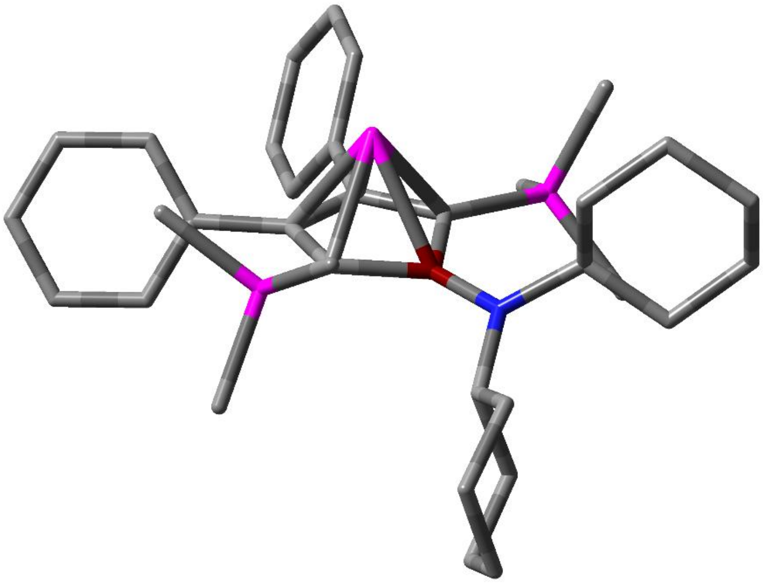

Figure S28. Calculated structure of NCy_2-substituted borasila[5]pyramidane **20f** (at M06-2X/6-311+G(d,p) level of theory, H-atoms are omitted for clarity, color code: violet Si, grey C, brown B, blue nitrogen). Calculated bond lengths [pm] and bond angles [°]: Si1-B 226.3, Si1-C1 208.4, Si1-C2 210.1, C1-C2 145.6, C2-C3 142.2, C3-C4 145.7, B-N 145.2, Si1-B-N-C^{α} 125.3, α(Si1) 79.1, α(B) 168.8.

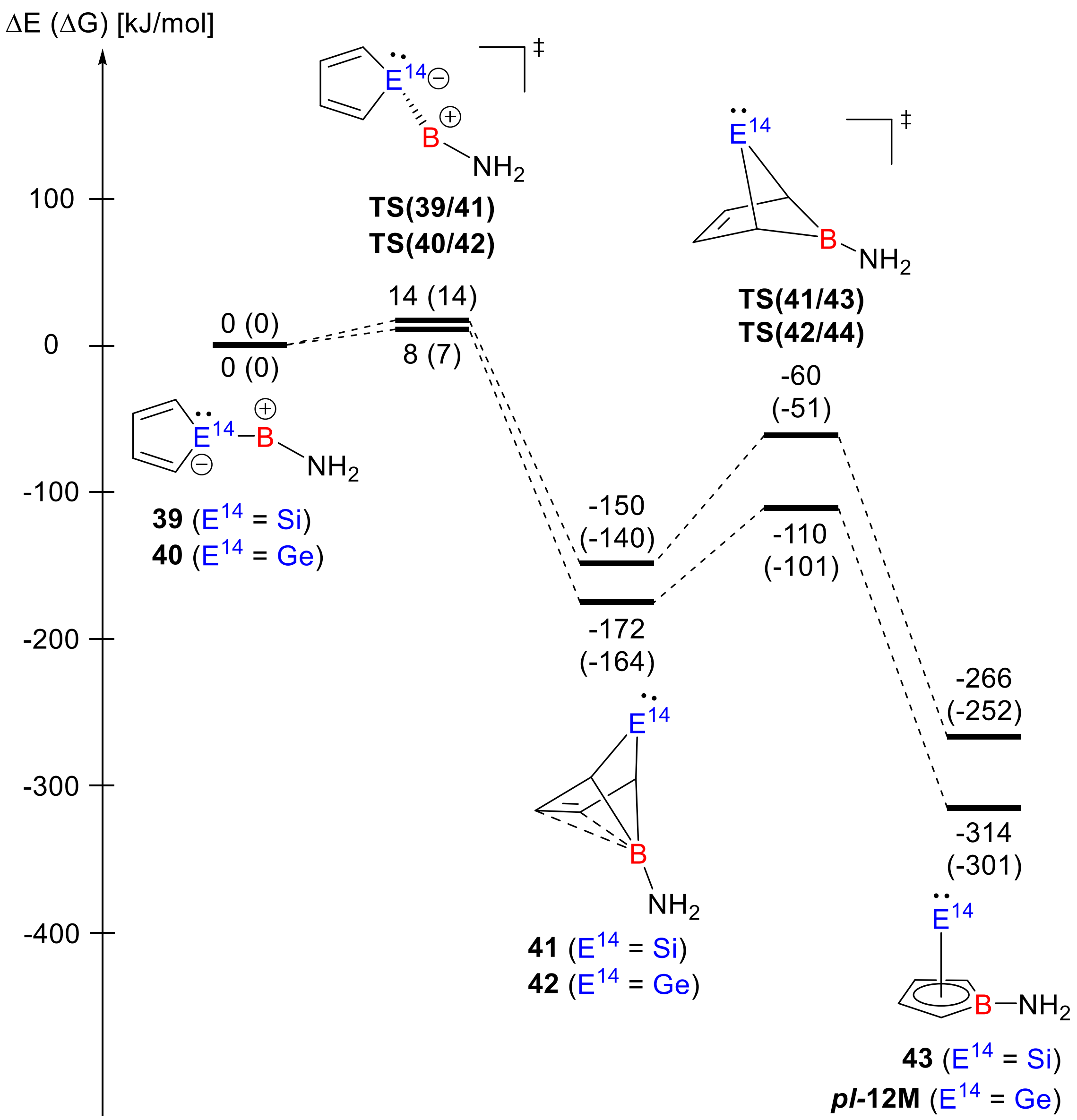

Figure S81. Reaction coordinate for the formation of the aminoborasila[5]pyramidane **43** compared to the formation of the aminoboragerma[5]pyramidane *pl*-**12M** (calculated at the M06-2X/Def2-TZVP level of theory, data for **12M** from reference [12]).

References

(1) Dong, Z.; Reinhold, C. R. W.; Schmidtmann, M.; Müller, T. *Trialkylsilyl-Substituted Silole and Germole Dianions*. *Organometallics* **2018**, *37* (24), 4736-4743. DOI: 10.1021/acs.organomet.8b00744.

(2) Ohashi, M.; Adachi, T.; Ishida, N.; Kikushima, K.; Ogoshi, S. *Synthesis and Reactivity of Fluoroalkyl Copper Complexes by the Oxycupration of Tetrafluoroethylene*. *Angew. Chem. Int. Ed.* **2017**, *56* (39), 11911-11915. DOI: https://doi.org/10.1002/anie.201703923.

(3) Krause, L.; Herbst-Irmer, R.; Sheldrick, G. M.; Stalke, D. *Comparison of silver and molybdenum microfocus X-ray sources for single-crystal structure determination*. *J. Appl. Crystallogr.* **2015**, *48* (1), 3-10. DOI: doi:10.1107/S1600576714022985.

(4) Sheldrick, G. *A short history of SHELX*. *Acta Crystallogr. A* **2008**, *64* (1), 112-122. DOI: doi:10.1107/S0108767307043930.

(5) Sheldrick, G. *Crystal structure refinement with SHELXL*. *Acta Crystallogr., Sect. C: Cryst. Struct. Commun.* **2015**, *71* (1), 3-8. DOI: doi:10.1107/S2053229614024218.

(6) Dolomanov, O. V.; Bourhis, L. J.; Gildea, R. J.; Howard, J. A. K.; Puschmann, H. *OLEX2: a complete structure solution, refinement and analysis program*. *J. Appl. Crystallogr.* **2009**, *42* (2), 339-341. DOI: doi:10.1107/S0021889808042726.

(7) Scheibitz, M.; Bolte, M.; Bats, J. W.; Lerner, H. W.; Nowik, I.; Herber, R. H.; Krapp, A.; Lein, M.; Holthausen, M. C.; Wagner, M. *C5H4□BR2 Bending in Ferrocenylboranes: A Delocalized Through-Space Interaction Between Iron and Boron*. *Chem. Eur. J.* **2005**, *11* (2), 584-603. DOI: https://doi.org/10.1002/chem.200400690.

(8) Gerrard, W.; Hudson, H. R.; Mooney, E. F. *995. Chemistry related to borazole. Part II. The reaction of secondary amines with boron trichloride*. *J. Chem. Soc.* **1960**, 5168-5172, 10.1039/JR9600005168. DOI: 10.1039/JR9600005168.

(9) Niedenzu, K.; Dawson, J. W. *Boron-Nitrogen Compounds. II.1,2 Aminoboranes, Part 1: The Preparation of Organic Substituted Aminoboranes through a Grignard Reaction*. *J. Am. Chem. Soc.* **1959**, *81* (21), 5553-5555. DOI: 10.1021/ja01530a010.

(10) De, S.; Mondal, A.; Chen, Y.-C.; Tong, M.-L.; Layfield, R. A. *Single-molecule Magnet Properties of Silole- and Stannole-ligated Erbium Cyclo-octatetraenyl Sandwich Complexes*. *Chem. Eur. J.* **2025**, *31* (17), e202500011. DOI: https://doi.org/10.1002/chem.202500011.

(11) Albers, L.; Tholen, P.; Schmidtmann, M.; Müller, T. *A germaaluminocene*. *Chem. Sci.* **2020**, *11* (11), 2982-2986, 10.1039/D0SC00401D. DOI: 10.1039/D0SC00401D.

(12) Tholen, P.; Dong, Z.; Schmidtmann, M.; Albers, L.; Müller, T. *A Neutral η5-Aminoborole Complex of Germanium(II)*. *Angew. Chem. Int. Ed.* **2018**, *57* (40), 13319-13324, https://doi.org/10.1002/anie.201808271. DOI: https://doi.org/10.1002/anie.201808271.

(13) Sarcevic, J.; Heitkemper, T.; Sindlinger, C. P. *Borole-based half-sandwich complexes of germanium and tin*. *Chem. Commun.* **2022**, *58* (2), 246-249, 10.1039/D1CC06227A. DOI: 10.1039/D1CC06227A.

(14) Herberich, G. E.; Hostalek, M.; Laven, R.; Boese, R. *Borole Dianions: Metalation of 1-(Dialkylamino)-2,5-dihydro-1H-boroles and the Structure of* $Li_2(C_4H_4BNEt_2)$*·TMEDA*. *Angew. Chem. Int. Ed. Engl.* **1990**, *29* (3), 317-318. DOI: https://doi.org/10.1002/anie.199003171.

(15) *Gaussian-16* Gaussian, Inc.: Wallingford CT, 2016.

(16) Glendening, E. D.; Weinhold, F. *Natural resonance theory: I. General formalism*. *J. Comput. Chem.* **1998**, *19* (6), 593-609. DOI: https://doi.org/10.1002/(SICI)1096-987X(19980430)19:6<593::AID-JCC3>3.0.CO;2-M.

(17) *NBO 7.0*; Theoretical Chemistry Institute, University of Wisconsin, Madison, 2018.

(18) *Jmol: an open-source Java viewer for chemical structures in 3D*; http://www.jmol.org/.

(19) *AIMALL (Version 19.10.12)*; TK Gristmill Software 2019. aim.tkgristmill.com.

(20) Zhao, Y.; Truhlar, D. G. *The M06 suite of density functionals for main group thermochemistry, thermochemical kinetics, noncovalent interactions, excited states, and transition elements: two new functionals and systematic testing of four M06-class functionals and 12 other functionals*. *Theor. Chem. Acc.* **2008**, *120* (1), 215-241. DOI: 10.1007/s00214-007-0310-x.

(21) Zhao, Y.; Truhlar, D. G. *Improved Description of Nuclear Magnetic Resonance Chemical Shielding Constants Using the M06-L Meta-Generalized-Gradient-Approximation Density Functional*. *J. Phys. Chem. A* **2008**, *112* (30), 6794-6799. DOI: 10.1021/jp804583d.
